# Over-expression of lncRNA TMEM161B-AS1 promotes the malignant biological behavior of glioma cells and the resistance to temozolomide via up-regulating the expression of multiple ferroptosis-related genes by sponging hsa-miR-27a-3p

**DOI:** 10.1038/s41420-021-00709-4

**Published:** 2021-10-23

**Authors:** Qiudan Chen, Weifeng Wang, Zhong Wu, Shuying Chen, Xiaotong Chen, Shihao Zhuang, Guanglei Song, Yuan Lv, Yong Lin

**Affiliations:** 1 Departments of Laboratory Medicine, Huashan Hospital, Fudan University, Shanghai, China; 2 Department of Clinical Laboratory, Central Laboratory, Jing’an District Center Hospital of Shanghai, Fudan University, Shanghai, China; 3 Department of Central Laboratory, Clinical Medicine Scientific and Technical Innovation Park, Shanghai Tenth People’s Hospital, Tongji University School of Medicine, Shanghai, China; 4 Department of Orthopedics, Shanghai Tenth People’s Hospital, Tongji University School of Medicine, Shanghai, China; 5 Fujian Children’s Hospital; Fujian Maternity and Child Health Hospital, Affiliated Hospital of Fujian Medical University, Fuzhou, China; 6 Department of Clinical Laboratory, Center of Naval Spectial Medicine, Naval Medical University, Shanghai, China

**Keywords:** Cancer, Oncogenesis

## Abstract

A growing body of evidence suggests that long-chain non-coding RNA (lncRNA) plays an important role in the malignant biological behavior and drug resistance of glioblastoma (GBM) cells. In this study, we analyzed the role and potential mechanism of lncRNA TMEM161B-AS1 in the malignant biological behavior of GBM cells and temozolomide (TMZ) resistance. Studies have found that FANCD2 and CD44 are significantly related to the occurrence of GBM, TMZ resistance and the survival of GBM patients. Knockdown of TMEM161B-AS1 down-regulated the expression of FANCD2 and CD44 by sponging hsa-miR-27a-3p, inhibited the proliferation, migration, invasion and promoted apoptosis, ferroptosis of U87 cells and U251 cells. Down-regulation of lncRNA TMEM161B-AS1 and/or over-expression of hsa-miR-27a-3p down-regulated the expression of FANCD2 and CD44, and inhibited the tumor growth in nude mice. These results demonstrated that the lncRNA TMEM161B-AS1-hsa-miR-27a-3p-FANCD2/CD44 signal axis regulated the malignant biological behavior of GBM and TMZ resistance. These findings were expected to provide promising therapeutic targets for the treatment of glioma.

## Introduction

Glioblastoma (GBM) is a primary central nervous system tumor with the highest incidence, high malignancy, and poor prognosis in adults [1–3]. Currently, standard treatment of GBM is neurosurgical resection, combined with radiotherapy and chemotherapy [4–8]. Due to the diffuse invasive growth characteristics of GBM, the tumors cannot be completely removed by surgical means [9]. GBM stem cells (GSC) in GBM give tumors the characteristics of genetic heterogeneity and abnormal microvascular proliferation, making them resistant to radiotherapy and chemotherapy [10–12]. Temozolomide (TMZ) is the first-line for GBM [13, 14], which can cross the blood-brain barrier and induce GBM cell apoptosis. Unfortunately, the clinical application of TMZ has been hampered by resistance of GBM cells [15, 16]. Therefore, it is important to elucidate mechanisms of TMZ resistance in GBM.

MicroRNAs (miRNAs) are a class of short-stranded, evolutionarily conserved, single-stranded, non-coding RNA molecules. The mature miRNA is incorporated into the RNA-induced silencing complex (RISC), and then negatively regulate gene expression by binding to target mRNA to prevent protein translation of mRNA [17]. Researchers identified in the blood of glioma patients and found that hsa-miR-27a-3p and its target genes were related to the progression of glioma [18].

The Fanconi anemia complementation group D2 (*FANCD2*) gene is located on the human chromosome 3p25.3. FANCD2 is the core link of the Fanconi anemia (FA) signaling pathway, and the FA signaling pathway is the main signaling pathway for DNA cross-linking damage repair, which participates in DNA damage repair processes such as nucleotide excision repair, homologous recombination repair, and cross-damage synthesis repair [19]. Studies have shown that FANCD2 re-expression was associated with glioma grade and chemical inhibition of the Fanconi Anaemia pathway sensitises gliomas to chemotherapeutic agents [20]. This research provides a strong basis for developing novel and effective FA signaling pathway inhibitors to improve the treatment of GBM. Although *FANCD2* was not up-regulated in TMZ-insensitive U251 cells according to the GSE100736 dataset, but FANCD2 was highly expressed in GBM tumor tissues, it was a ferroptosis-related gene [21], and FANCD2 overexpression improved the viability of TMZ-treated U87 cells [22]. Song et al. [23] and Fathima et al. [24] found that FANCD2 played an active role in the negative regulation of ferroptosis.

The *CD44* gene is located on the human chromosome 11p13, and the CD44 protein is related to cell-cell interaction, cell adhesion and migration [25]. High expression of CD44 is associated with poor survival of GBM patients [26], and CD44 GBM is closely related to histopathological grade and cell migration in human [27]. These genes that were differentially expressed during the occurrence of GBM and related to TMZ resistance have attracted our attention. In particular, the *FANCD2* gene and *CD44* gene (Supplementary materials) [21, 28] related to ferroptosis are particularly worthy of our focus on research.

TMZ resistance in GBM is related to different cellular pathways. For example, Wick et al. [29] found that high expression of O-6-methylguanine-DNA methyltransferase (MGMT) is related to TMZ resistance. The results of Sun et al. [30] showed that the sensitivity of GBM cells to TMZ increased after prolyl 4-hydroxylase, β polypeptide (P4HB) was inhibited. Schäfer et al. [31] found that GBM resistance to TMZ was mediated by ALDH1A1, which was expected to become a potential target to improve treatment of GBM.

In this study, we analyzed the endogenous expression of lncRNA TMEM161B-AS1, hsa-miR-27a-3p, FANCD2, CD44 and the influence on the malignant biological behavior of GBM cells. Further, we also studied whether lncRNA TMEM161B-AS1 regulated the expression of FANCD2 and CD44 by regulating the expression of hsa-miR-27a-3p. This study aimed to study the possible function of lncRNA TMEM161B-AS1- hsa-miR-27a-3p- FANCD2/CD44 crosstalk in the malignant biological behavior of GBM and TMZ resistance.

## Results

### Identification of glioma and TMZ resistance related genes and non-coding RNAs

**Fig. 1:**
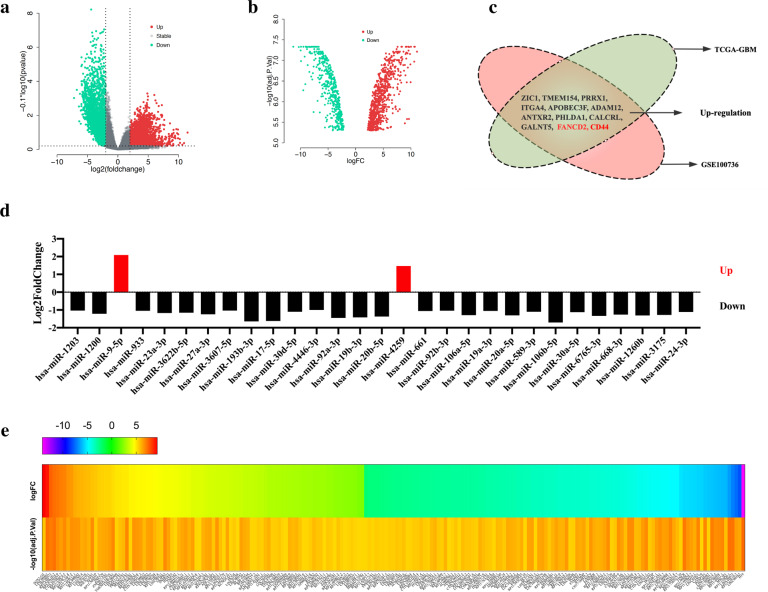
Differential expression analysis results of genes and non-coding RNAs related to glioma and temozolomide resistance. **a** Volcano plot analysis of differentially expressed gene in the TCGA-GBM dataset. It contains data on 5 normal tissue samples and 156 tumor tissue samples. **b** Volcano plot analysis of differentially expressed genes in TMZ-sensitive and TMZ-resistant U251 glioma cell lines in the GSE100736 dataset. **c** Venn diagram of up-regulated gene distribution in TCGA-GBM dataset and GSE100736 dataset. **d** Analysis results of differentially expressed microRNAs in TMZ-resistant GBM cell line and TMZ-sensitive GBM cell line in the GSE100775 dataset. **e** The analysis results of differentially expressed lncRNAs in TMZ-sensitive and TMZ-resistant U251 glioma cell lines in GSE100736 dataset.

According to the TCGA database, a total of 2913 gene were up-regulated (log2FoldChange > 2, *p* < 0.001), and 2443 genes were down-regulated (log2FoldChange < −2, *p* < 0.001) (Supplementary materials, Fig. 1a). According to the GSE100736 dataset, 1263 gene expression was up-regulated (log2FoldChange > 2, *p* < 0.001), and 653 gene expression was down-regulated (log2FoldChange < −2, *p* < 0.001) (Fig. 1b). We found that 11 genes were both up-regulated in glioma cells and TMZ-insensitive U251 cells, namely *ZIC1*, *TMEM154*, *PRRX1*, *ITGA4*, *APOBEC3F*, *ADAM12*, *ANTXR2*, *PHLDA*1, *CALCRL*, *GALNT5* and *CD44* (Fig. 1c). According to the GSE100775 dataset, 2 miRNAs up-regulated (log2FoldChange > 1, *p* < 0.001) and 27 miRNAs down-regulated (log2FoldChange < −1, *p* < 0.001) (Fig. 1d). According to the GSE100736 dataset, a total of 93 lncRNAs up-regulated (log2FoldChange > 2, *p* < 0.001) and 110 down-regulated (log2FoldChange < −2, *p* < 0.001) (Fig. 1e).

### Ferroptosis-related genes were related to the survival of GBM patients

**Fig. 2:**
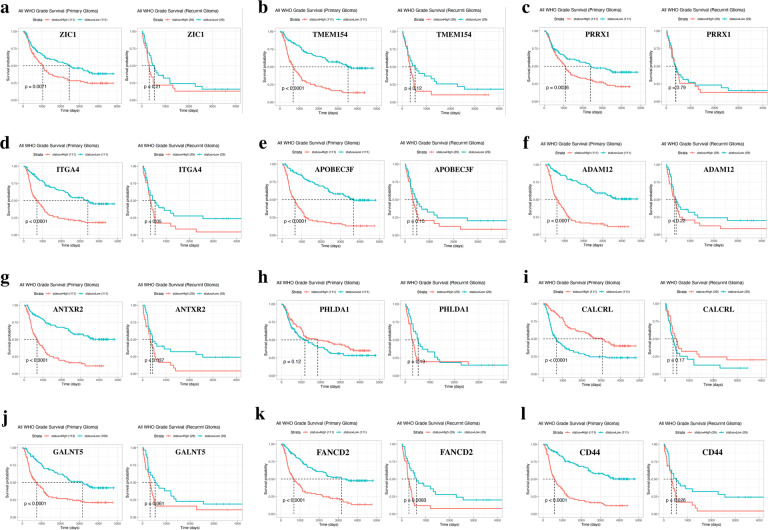
Kaplan–Meier survival analysis for glioma patients with low and high gene expression in CGGA datasets. **a**–**l** Comparison of Survival of Primary Glioma and Recurrent Glioma patients with high and low ZIC1, TMEM154, PRRX14, ITGA4, APOBEC3F, ADAM12, ANTXR2, PHLDA1, CALCRL, GALNT5, FANCD2, CD44 expression.

According to the CGGA database, the correlation between the expression levels of the above-upregulated genes and the survival of GBM patients was analyzed. The results showed that the survival time of primary GBM and recurrent GBM patients with high expression level of ferroptosis-related genes *FANCD2* and *CD44* were significantly lower than that of patients with low expression level (*p* < 0.05, Fig. 2).

### Down-regulation of FANCD2 and CD44 expression is associated with ferroptosis in U87 and U251 cells

**Fig. 3:**
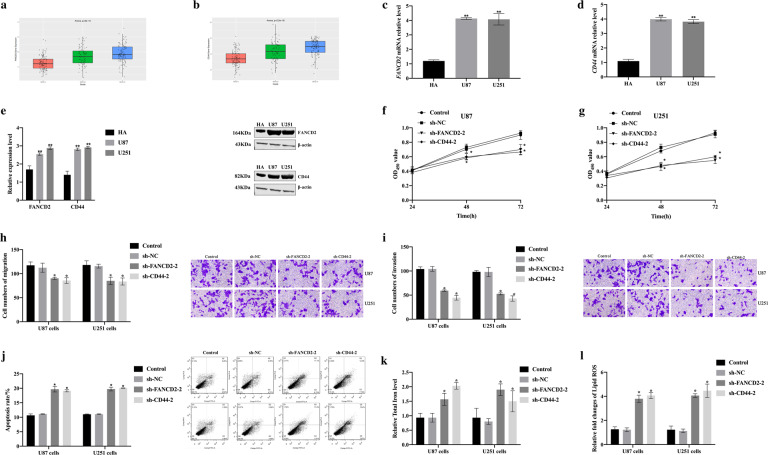
Down-regulation of FANCD2 and CD44 promoted ferroptosis of U87 cells and U251 cells. **a, b** Comparison of *FANCD2* and *CD44* gene expression level in patients with different WHO Grade gliomas. **c, d** The relative expression level of *FANCD2* mRNA and *CD44* mRNA in human astrocytes (HA), U87 and U251 cells were detected by qRT-PCR. **e** The relative expression levels of FANCD2 and CD44 in HA, U87 and U251 cells were detected by western blot. **f, g** The viability of U87 and U251 cells after sh-NC, sh-FANCD2-2, sh-CD44-2 transfection were detected by Cell Counting Kit-8 assay. **h, i** The migration and invasion ability of U87 and U251 cells after sh-NC, sh-FANCD2-2, sh-CD44-2 transfection were detected by Transwell assay. **j** The apoptosis rate of U87 and U251 cells after sh-NC, sh-FANCD2-2, sh-CD44-2 transfection were detected by Flow cytometry. **k, l** Comparison of the total iron levels and lipid ROS levels in U87 and U251 cells after sh-NC, sh-FANCD2-2, and sh-CD44-2 transfection. *n* = 3 per group.

In order to evaluate the effects of *FANCD2* and *CD44* gene on the proliferation, migration and apoptosis of U87 cells and U251 cells, we successfully constructed two kinds of short hairpin RNA (shRNA) for each gene, namely sh-FANCD2-1, sh-FANCD2-2, sh-CD44-1, sh-CD44-2 (Figure S1), and the shRNAs with high knockout efficiency (sh-FANCD2-2, sh-CD44-2) were selected for subsequent experiments. According to the CGGA database, the expression levels of FANCD2 and CD44 were positively correlated with the grade of the tumor (Fig. 3a, b) [32, 33]. The results showed that the relative expression levels of *FANCD2* and *CD44* genes in U87 and U251 cells were significantly higher than that in human astrocytes (HA) (Fig. 3c–e). Compared with the sh-NC group, the viability of U87 cells and U251 cells transfected with sh-FANCD2-2 and sh-CD44-2 were significantly decreased (Fig. 3f, g). The migration and invasion capabilities of U87 cells and U251 cells transfected with sh-FANCD2-2 and sh-CD44-2 were significantly decreased (Fig. 3h, i). The apoptosis rate, the concentration of iron and lipid ROS of U87 cells and U251 cells transfected with sh-FANCD2-2 and sh-CD44-2 increased significantly (Fig. 3j–l).

The above research results indicated that *FANCD2* and *CD44* genes acted as the oncogenes in U87 cells and U251 cells, and silencing of FANCD2 and CD44 related to ferroptosis in U87 cells and U251 cells.

### Silencing of FANCD2 and CD44 were related to the TMZ sensitivity in U87 cells and U251 cells

**Fig. 4:**
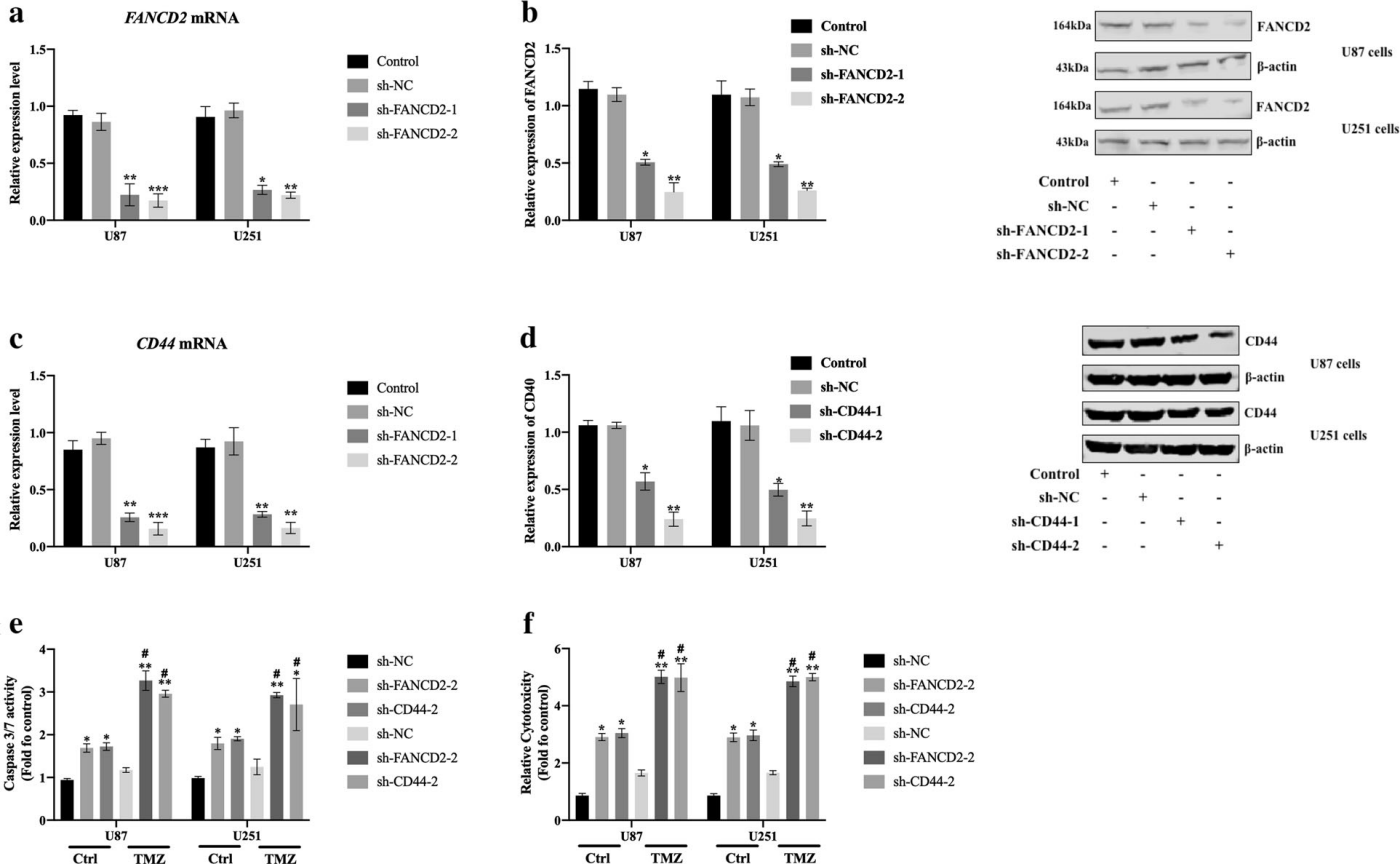
FANCD2 and CD44 silenced U87 cells and U251 cells were sensitive to TMZ. **a** The relative expression levels of FANCD2 protein in the non-transfected group (Control), blank control (sh-NC), sh-FANCD2-1, sh-FANCD2-2 transfected U87 and U251 cells were detected by western blot. **b** The relative expression levels of CD44 protein in Control, sh-NC, sh-CD44-1, sh-CD44-2 transfected U87 and U251 cells were detected by western blot. **c** Comparison of Caspase 3/7 activities in TMZ treated or without TMZ (Ctrl) treated U87 and U251 cells after transfected with sh-NC, sh-FANCD2, and sh-CD44. **d** Comparison of LDH release in TMZ treated or Ctrl treated U87 and U251 cells after transfected with sh-NC, sh-FANCD2, and sh-CD44. *p < 0.05, compared with sh-NC group; **p < 0.01, compared with sh-NC group; #*p* < 0.05, compared with Ctrl group. *n* = 3 per group.

Next, we studied the correlation between the silencing of FANCD2 and CD44 and the TMZ sensitivity in U87 cells and U251 cells. The results of qRT-PCR and Western blot proved that we had successfully silenced *FANCD2* and *CD44* gene in U87 cells and U251 cells (Fig. 4a, b, c, d). The caspase 3/7 activities and LDH release were significantly increased after FANCD2 and CD44 were silenced, and TMZ treated significantly promoted the caspase 3/7 activities and LDH release (Fig. 4e, f). The above results indicated that silencing of FANCD2 and CD44 were related to the TMZ sensitivity in U87 cells and U251 cells.

### Knockdown of lncRNA TMEM161B-AS1 inhibited cell proliferation, migration and invasion, while promoted glioma cell apoptosis

**Fig. 5:**
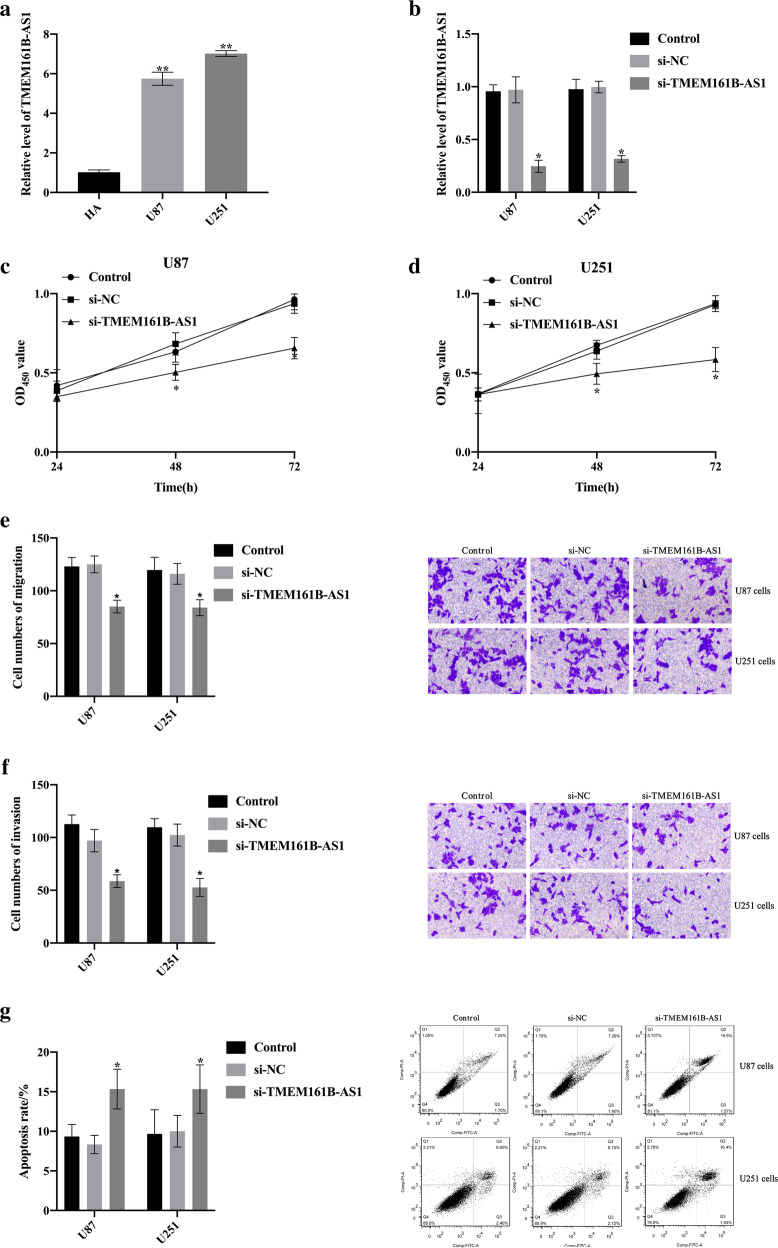
Knockdown TMEM161B-AS1 inhibited the proliferation, migration, invasion of glioma cells and promoted apoptosis. **a** The TMEM161B-AS1 levels in U87, U251 and human astrocytes (HA) cells were detected by qRT-PCR. **b** The TMEM161B-AS1 levels in the Control, si-NC, si-TMEM161B-AS1 transfected U87 and U251 cells. **c, d** The cell viability of U87, U251 cells transfected with Control, si-NC and si-TMEM161B-AS1 were analyzed by Cell Counting Kit-8 assay. **e, f** The cell migration and invasion ability of Control, si-NC, si-TMEM161B-AS1 transfected U87 and U251 cells were detected by Transwell assay. **g** The apoptosis rate of Control, si-NC, si-TMEM161B-AS1 transfected U87 and U251 cells were detected by Flow cytometry. *n* = 3 per group.

Combined with The Encyclopedia of RNA Interactomes (ENCORI) tool [34], we have a keen interest in lncRNA TMEM161B-AS1. According to the results of the GSE100736 dataset, lncRNA TMEM161B-AS1 was significantly up-regulated in TMZ-resistant U251 cells (Supplementary materials). The expression level of lncRNA TMEM161B-AS1 in U87 cells and U251 cells were significantly higher than that of human astrocytes (HA) (Fig. 5a). The results of qRT-PCR proved that we had successfully knocked down lncRNA TMEM161B-AS1 in U87 cells and U251 cells (Fig. 5b). Compared with the si-NC group, the proliferation ability of U87 cells and U251 cells transfected with si-TMEM161B-AS1 were significantly decreased (Fig. 5c, d). The migration ability (Fig. 5e) and invasion ability (Fig. 5f) of U87 cells and U251 cells transfected with si-TMEM161B-AS1 were inhibited, but the apoptosis rate increased (Fig. 5g).

The above results indicated that knockdown of lncRNA TMEM161B-AS1 inhibited cell proliferation, migration and invasion, while promoted glioma cell apoptosis.

### lncRNA TMEM161B-AS1 functioned as a sponge of hsa-miR-27a-3p

**Fig. 6:**
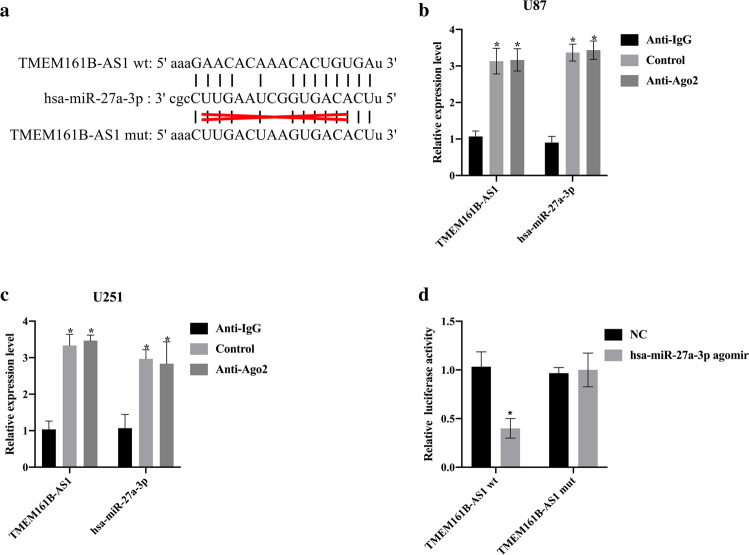
TMEM161B-AS1 acted as a sponge of hsa-miR-27a-3p. **a** Prediction of binding sites between TMEM161B-AS1 and hsa-miR-27a-3p by StarBase v2.0. **b** Compared of TMEM161B-AS1 and hsa-miR-27a-3p levels between RIP assay with antibody Ago2, IgG, or input from U87 cell extracts. *p < 0.05, compared with NC. **c** Compared of TMEM161B-AS1 and hsa-miR-27a-3p levels between RIP assay with antibody Ago2, IgG, or input from U251 cell extracts. *p < 0.05, compared with NC. **d** Luciferase reporter assay was performed to detect luciferase activity in U87 and U251 cells co-transfected with the constructed luciferase reporter plasmids (TMEM161B-AS1 wt or TMEM161B-AS1 mut) and hsa-miR-27a-3p angomir or no temple control (NC). *p < 0.05, compared with NC. *n* = 3 per group.

By using StarBase v2.0 (http://starbase.sysu.edu.cn/index.php) [34], we found that lncRNA TMEM161B-AS1 contained a binding sequence complementary to the seed region of hsa-miR-27a-3p (Fig. 6a). We used Ago2 antibody to perform RNA immunoprecipitation (RIP) analysis in U87 cells and U251 cells to detect whether lncRNA TMEM161B-AS1 and hsa-miR-27a-3p interacted with Ago2. The results showed that compared with the control group (NC), lncRNA TMEM161B-AS1 and hsa-miR-27a-3p were significantly enriched on the miRNA ribonucleoprotein complex of Ago2 (*p* < 0.05, Fig. 6b, c). This result indicated that lncRNA TMEM161B-AS1 and hsa-miR-27a-3p were both present in the same RISC complex. In order to further study whether lncRNA TMEM161B-AS1 and hsa-miR-27a-3p interact at the predicted binding site, we constructed a luciferase reporter plasmid containing wild-type (wt) with the predicted binding site sequence of hsa-miR-27a-3p and a mutant (mut) with the binding site sequence (Fig. 6a). When hsa-miR-27a-3p angomir and TMEM161B-AS1 wt were co-transfected into HEK-293T cells, a significant decrease in luciferase activity was detected, however no significant change in luciferase activity was detected after hsa-miR-27a-3p angomir and TMEM161B-AS1 mut were co-transfected (Fig. 6d). This indicated that lncRNA TMEM161B-AS1 functioned as a sponge of hsa-miR-27a-3p.

### The inhibitory effect of lncRNA TMEM161B-AS1 silencing on GBM cells was mediated by hsa-miR-27a-3p

**Fig. 7:**
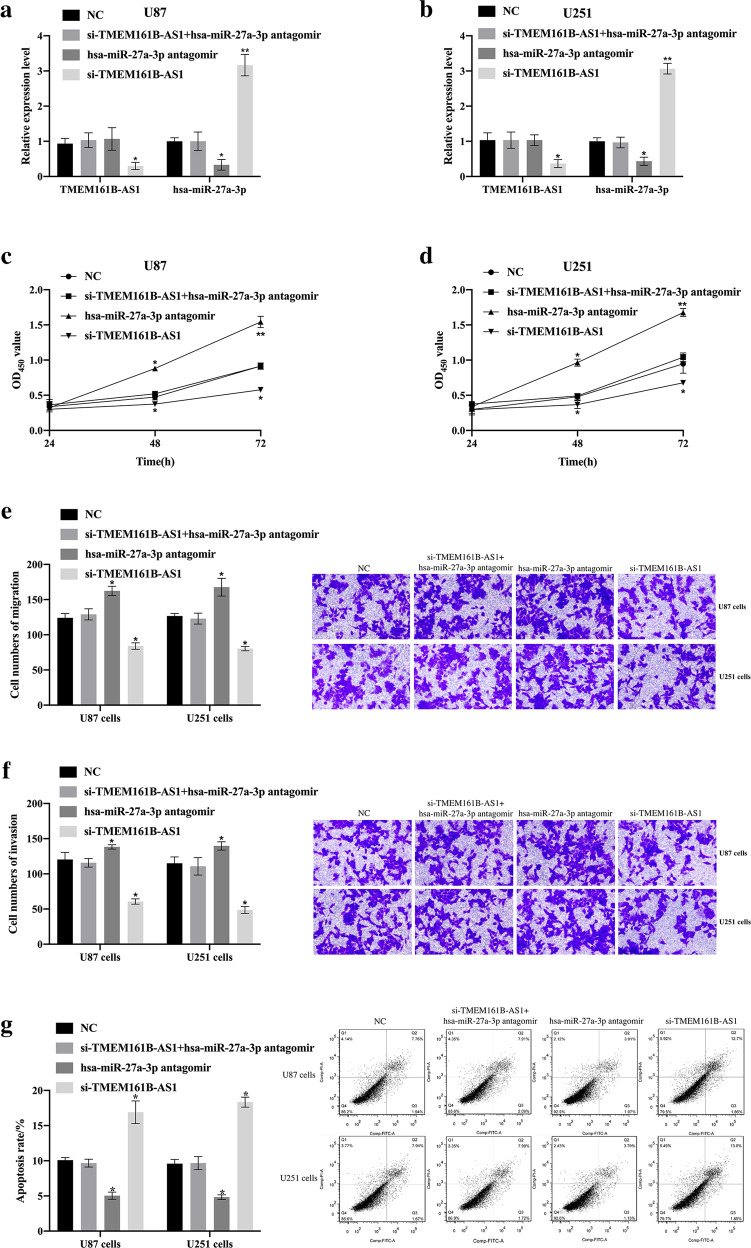
The tumor suppressor effect caused by TMEM161B-AS1 knockout was mediated by hsa-miR-27a-3p. **a, b** The hsa-miR-27a-3p, TMEM161B-AS1 expression level in U87 cells and U251 cells transfected by no template control (NC), si-TMEM161B-AS1 + hsa-miR-27a-3p antagomir, hsa-miR-27a-3p antagomir, si-TMEM161B-AS1 were detected by qRT-PCR. **c, d** Comparison of cell viability of U87 cells and U251 cells after transfected with NC, si-TMEM161B-AS1 + hsa-miR-27a-3p antagomir, hsa-miR-27a-3p antagomir, and si-TMEM161B-AS1. **e**, **f** Comparison of cell migration and invasion ability of U87 cells and U251 cells after transfected with NC, si-TMEM161B-AS1 + hsa-miR-27a-3p antagomir, hsa-miR-27a-3p antagomir, si-TMEM161B-AS1. g. Comparison of apoptosis rate of U87 cells and U251 cells after transfection of NC, si-TMEM161B-AS1 + hsa-miR-27a-3p antagomir, hsa-miR-27a-3p antagomir, si-TMEM161B-AS1. *p < 0.05, compared with NC, **p < 0.01, compared with NC. *n* = 3 per group.

No template control (NC), si-TMEM161B-AS1 + hsa-miR-27a-3p antagomir, hsa-miR-27a-3p antagomir and si-TMEM161B-AS1 were transfected into U87 cells and U251 cells respectively and qRT-PCR was used to detect transfection efficiency (Fig. 7a, b). Compared with the NC group, the cell proliferation activity increased in the hsa-miR-27a-3p antagomir group, but decreased in si-TMEM161B-AS1 group (*p* < 0.05, Fig. 7c, d). Compared with the NC group, the cell migration and invasion abilities were enhanced in hsa-miR-27a-3p antagomir group, but inhibited in si-TMEM161B-AS1 group, but no significant change found in si-TMEM161B-AS1 + hsa-miR-27a-3p antagomir group (Fig. 7e, f). The cell apoptotic rate decreased significantly in hsa-miR-27a-3p antagomir group, and increased significantly in si-TMEM161B-AS1 group, but no significant change found in si-TMEM161B-AS1 + hsa-miR-27a-3p antagomir group (Fig. 7g).

The results showed that the inhibitory effect of lncRNA TMEM161B-AS1 silencing on GBM cells was mediated by hsa-miR-27a-3p.

### Hsa-miR-27a-3p inhibited the proliferation, migration and invasion of glioma cells and promoted apoptosis and ferroptosis by down-regulating the expression of FANCD2 and CD44

**Fig. 8:**
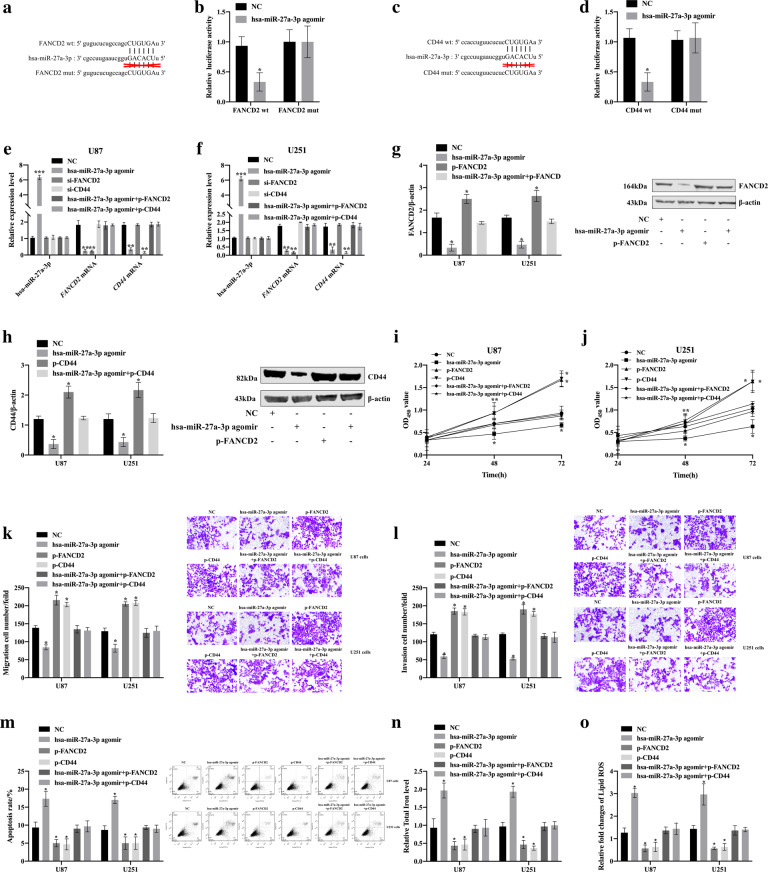
hsa-miR-27a-3p down-regulated the expression of FANCD2 and CD44, inhibited the proliferation, migration, invasion of glioma cells and promoted apoptosis and ferroptosis. **a, c** The predicted wild-type or mutated hsa-miR-27a-3p binding sites in *FANCD2* or *CD44*. **b, d** The regulatory relationship between hsa-miR-27a-3p and FANCD2 or CD44 was validated in luciferase reporter assay. **e, f** The transfection efficiency of U87 and U251 cells were estimated by qRT-PCR. **g, h** FANCD2 and CD44 expression level of U87 cells and U251 cells was estimated by Western Blot. **i**, **j** Influence of hsa-miR-27a-3p/FANCD2 axis and hsa-miR-27a-3p/CD44 axis on the proliferation of U87 and U251 cells was detected by Cell Counting Kit-8. **k**, **l** The migration and invasion ability of U87 and U251 cells were detected by Transwell assay. **m** Effects of hsa-miR-27a-3p/FANCD2 axis and hsa-miR-27a-3p/CD44 axis on apoptosis of U87 cells and U251 cells. **n**. Relative Total Iron level in U87 and U251 cells detected by Iron Assay Kit (Sigma Aldrich, Missouri, USA). **o** Lipid ROS was measured by C11-BODIPY staining coupled to flow cytometry in U87 and U251 cells. *p < 0.05, **p < 0.01, ***p < 0.001, compared with NC. *n* = 3 per group.

According to StarBase v2.0 [34] prediction, we found that hsa-miR-27a-3p contains complementary binding with the seed region of *FANCD2* gene and *CD44* gene 3’ UTR sequence (Fig. 8a, c). Next, we constructed the luciferase reporter plasmids contained *FANCD2* gene and *CD44* gene wild-type (wt) and mutant (mut) sequences, respectively (Fig. 8a, c). When hsa-miR-27a-3p angomir and FANCD2 wt or CD44 wt were co-transfected into HEK-293T cells, a significant decrease in luciferase activity was detected, however, after co-transfected hsa-miR-27a-3p angomir with FANCD2 mut or CD44 mut into HEK-293T cells, no significant changes in luciferase activity were detected (Fig. 8b, d). This indicated that hsa-miR-27a-3p bound to *FANCD2* and *CD44* 3’UTR sequence.

In addition, NC, hsa-miR-27a-3p angomir, si-FANCD2, si-CD44, hsa-miR-27a-3p angomir+p-FANCD2, hsa-miR-27a-3p angomir+p-CD44 were transfected into U87 cells and U251 cells, respectively, and the transfection efficiency were detected by qRT-PCR (Fig. 8e, f). The results showed that hsa-miR-27a-3p down-regulated the FANCD2 and CD44 expression in U87 cells and U251 cells (Fig. 8g, h).

The cell proliferation activity was significantly decreased in hsa-miR-27a-3p angomir group and increased in p-FANCD2 group and p-CD44 group, but no significant change in hsa-miR-27a-3p angomir+p-FANCD2 group or hsa-miR-27a-3p angomir+p-CD44 group (Fig. 8i, j). Cell migration and invasion capabilities reduced in hsa-miR-27a-3p angomir group, and increased in p-FANCD2 group and p-CD44 group, but no significant change in hsa-miR-27a-3p angomir+p-FANCD2 group and hsa-miR-27a-3p angomir+p-CD44 group (Fig. 8k, l).

Cell apoptosis increased in hsa-miR-27a-3p angomir group, and decreased in p-FANCD2 group and p-CD44 group, but no significant change in hsa-miR-27a-3p angomir+p-FANCD2 group and hsa-miR-27a-3p angomir+p-CD44 group (Fig. 8m). The concentration of iron and lipid ROS increased in hsa-miR-27a-3p angomir group and decreased in p-FANCD2 group and p-CD44 group, no significant change in hsa-miR-27a-3p angomir+p-FANCD2 group and hsa-miR-27a-3p angomir+p-CD44 group (Fig. 8n, o). These results indicated that hsa-miR-27a-3p inhibited the proliferation, migration and invasion of glioma cells and promoted apoptosis and ferroptosis by down-regulating the expression of FANCD2 and CD44.

### Silencing lncRNA TMEM161B-AS1 and/or overexpression of hsa-miR-27a-3p inhibited tumor growth in nude mice

A in vivo tumor xenograft assay was used to further determine the functions of lncRNA TMEM161B-AS1 and hsa-miR-27a-3p. First, qRT-PCR was used to detect the transfection efficiency of U87 cells (Fig. S2a) and U251 cells (Fig. S2b) after transfected with no template control (NC), si-TMEM161B-AS1, hsa-miR-27a-3p angomir, si-TMEM161B-AS1 + hsa-miR-27a-3p. The tumor volume of the si-TMEM161B-AS1 transfection group and hsa-miR-27a-3p angomir transfection group were significantly smaller than that of the NC group, and the si-TMEM161B-AS1 + hsa-miR-27a-3p angomir transfection group had the smallest tumor volume (Fig. S2c, d). The expression levels of FANCD2 and CD44 in the si-TMEM161B-AS1 transfection group and the hsa-miR-27a-3p angomir transfection group were significantly decreased, and the si-TMEM161B-AS1 + hsa-miR-27a-3p angomir transfection group had the lowest FANCD2 and CD44 expression levels (Fig. S2e, f). These results revealed that silencing lncRNA TMEM161B-AS1 and/or overexpression of hsa-miR-27a-3p inhibited tumor growth in nude mice.

## Discussion

In this study, we analyzed the differential expression genes and ncRNA in GBM cancer tissue and normal tissue from TCGA database and TMZ resistance-related genes and ncRNA in TMZ-sensitive and TMZ-insensitive U251 cells from GSE100736 dataset. In vitro studies have shown that lncRNA TMEM161B-AS1 regulated the expression of FANCD2 and CD44 by sponging hsa-miR-27a-3p, and changed the malignant biological behavior and TMZ resistance of U87 cells and U251 cells.

In recent years, the results of randomized clinical trials (RCT) and prospective studies have shown that postoperative adjuvant chemotherapy for glioma patients can prolong the overall survival time [35–38]. However, the emergence of drug resistance casts a shadow over the treatment of glioma. Therefore, further studies will be needed to better understand the pathogenesis and specific resistance mechanisms of glioma. It’s of great significance to find more effective therapeutic targets for glioma and to develop a more refined classification system.

Ferroptosis is induced by a variety of small molecular substances, leading to disorders of lipid oxide metabolism in cells, resulting in cell death caused by the production of excessive ROS [39]. Studies have shown that ferroptosis played an important role in the occurrence and development of tumors [40, 41]. After analyzing the data from CGGA, GSE16011 dataset and the Cancer Genome Atlas dataset, Liu *et al*. [21] found that several ferroptosis-related genes played an important role in glioma progression.

Long non-coding RNA (lncRNA) is a type of non-coding RNA with a length of more than 200 nucleotides [42]. Recent studies have shown that lncRNA not only plays an important role in many biological processes [43–45], but also acts as oncogene or tumor suppressor gene [46]. For example, lncRNA TP73-AS1 was related to the invasiveness of GBM and the sensitivity of GCS cells to TMZ [47]. Zhang *et al*. [48] found that lncRNA LINC01446 promoted GBM cell tumor progression through the miR-489-3p/TPT1 axis. *TMEM161B-AS1* gene is located on chromosome 5q14.3, one recent study found that lncRNA TMEM161B-AS1 was specifically associated with the light polysomal fraction [45]. However, the role of lncRNA TMEM161B-AS1 in GBM has not been reported until now.

FANCD2 played an important role in bone marrow stromal cells (BMSCs) in resisting ferroptosis-related damage [23]. Wu *et al*. [49] found that FANCD2 was associated with the risk of clear cell renal cell carcinoma (ccRCC), and high FANCD2 expression corresponded to a high risk of ccRCC. CD44 was closely related to the invasion and migration of glioma cells due to its key role in the adhesion between glioma cells. CD44 promoted the resistance of glioma cells to radiotherapy and chemotherapy, and promoted tumor cell formation and other biological functions [50–52]. In this study, we found that *FANCD2* gene and *CD44* genes act as oncogenes, and knockdown of *FANCD2* or *CD44* genes in U87 and U251 cells were related to ferroptosis. We found that the expression levels of *FANCD2* and *CD44* genes were related to the survival of Chinese glioma patients according to the data from the mRNAseq_325 dataset in the CGGA database. Interestingly, we found that knockdown of FANCD2 and CD44 were associated with TMZ sensitive in U87 cells and U251 cells. We suggest that FANCD2 and CD44 may be one of the potential molecular targets to solve the TMZ resistance of U87 cells and U251 cells.

Bioinformation predictions suggested that lncRNA TMEM161B-AS1, FANCD2 and CD44 are potential targets for hsa-miR-27a-3p, which we have also confirmed in the in vitro dual-luciferase reporter assay and cell transfection experiments. Based on the results, we speculate that there may be a lncRNA-miRNA-gene network among lncRNA TMEM161B-AS1, hsa-miR-27a-3p, FANCD2 or CD44, which involves in the occurrence of gliomas.

In order to verify our hypothesis, we found that knockdown of lncRNA TMEM161B-AS1 inhibited the proliferation, migration, invasion of glioma cells, and promoted apoptosis and ferroptosis by down-regulating the expression of FANCD2 and CD44 through sponging hsa-miR-27a-3p. Previous studies have shown that ferroptosis was caused by fatal lipid peroxidation [53]. Excessive consumption of ferritin or transporter caused the accumulation of iron in cells and subsequent peroxidation, leading to lipid peroxides and ferroptosis [54]. In this study, we found that when FANCD2 and CD44 were highly expressed in U87 and U251 cells, the concentration of iron and lipid ROS decreased significantly. When the expression of FANCD2 and CD44 was inhibited, the concentration of iron and lipid ROS were increased significantly, cell proliferation, migration and invasion were inhibited, and the apoptosis rate was significantly increased. Studies in nude mouse models found that knockdown of lncRNA TMEM161B-AS1 and/or overexpression of hsa-miR-27a-3p inhibited tumor growth. The above findings suggested that FANCD2 and CD44 may be potential target molecules of glioma.

However, there are some limiting factors that need to be further studied and resolved. First of all, the specific molecular mechanisms of the effects of FANCD2 and CD44 on the proliferation, migration and invasion of glioma cells need to be elucidated. Secondly, this study failed to detect cyclin and cell cycle. Third, the failure to study the survival period of nude mice is also one of the shortcomings that need to be remedied. Fourth, the signal pathways involved in the production of malignant biological behaviors of tumor cells are intricate and it is worth studying whether there are other signal molecules involved. Therefore, only studying the regulatory effects of lncRNA and/or miRNA may be due to their effects on different genes. Regulatory effects may adversely affect the regulation of these targets. Fifth, in order to study the role of the genes of study in the regulation of ferroptosis, analysis of the expression of genes related to this process in the different experimental conditions need to be performed.

## Conclusions

In summary, this study demonstrated that the lncRNA TMEM161B-AS1-hsa-miR-27a-3p-FANCD2/CD44 network regulated the malignant biological behavior of GBM cells and TMZ sensitivity. The results of this study not only contributed to the intensive study on the explicit mechanism of occurrence of glioma, but also provided promising therapeutic targets for the treatment of glioma.

## Materials and methods

### Dataset

All the datasets used in this study were obtained from public databases, including RNA-seq data from glioma patients in the TCGA dataset (https://portal.gdc.cancer.gov/), and the GSE100736 dataset, the Chinese Glioma Genome Atlas (CGGA) dataset (http://www.cgga.org.cn/).

### Cell culture

The U87 (RRID: CVCL_0022), U251 cells (RRID: CVCL_0021) and human embryonic kidney (HEK) 293 T cells were derived from American Type Culture Collection (ATCC, Manassas, VA). Cells were incubated at 37°C under 5% CO_2_ and cultivated in Dulbecco’s Modified Eagle Medium (DMEM, GIBCO, Grand Island, NY) containing 10% Fetal Bovine Serum (FBS) (Gibco, Carlsbad, CA, USA) and 1% penicillin-streptomycin (GIBCO, Grand Island, NY). All experiments were performed with mycoplasma-free cells.

### Real-time fluorescent quantitative PCR (qRT-PCR)

Total RNA was isolated from the cells with Trizol reagent (Life Technologies Corporation, Carlsbad, CA, USA). The concentration of the isolated RNA was measured by NanoDrop-2000 Spectrophotometer (Thermo Scientific, Wilmington, USA). According to the supplier’s instructions, the PrimeScript RT kit (Takara, Shiga, Japan) was used to reverse-transcribe total RNA into cDNA, and then the SYBR Premix Ex Taq kit (Takara, Shiga, Japan) was used for PCR analysis. The primer sequences used for qRT-PCR analysis in this study were listed in Supplemental Table S1. Glyceraldehyde-3-phosphate dehydrogenase (GAPDH) and U6 were used as endogenous controls, the expression level was normalized to the endogenous control, and the fold change was calculated using the 2^-ΔΔCt^ method.

### Cell transfection

Short hairpin RNA (shRNA) against *FANCD2* or *CD44* gene, as well as their non-targeting sequences were constructed in pGPU6/RFP/Neo vector (GenePharama, Shanghai, China). Hsa-miR-27a-3p agomir, hsa-miR-27a-3p antagomir, and their respective negative control were synthesized (GenePharama, Shanghai, China). U87 and U251 cells were seeded in 24-well plates (Corning, NY, USA), when cells reached 70%-80% confluence, the recombinant plasmid were transfected into U87 and U251 cell by Lipofectamine 3000 (Life Technologies, Carlsbad, CA). G418 (Sigma-Aldrich, St Louis, MO, USA) was used to screen stably transfected cells. The transfection efficacy was analyzed by qRT-PCR and/or western blot. To evaluate the transfection efficiency of FANCD2 and CD44 silencing vectors in U87 and U251 cells, cells were divided into Control group, sh-no temple control(sh-NC) group, sh-FANCD2-1 group and sh-FANCD2-2 group for *FANCD2* gene knockdown, and Control group, sh-NC group, sh-CD44-1 group, sh-CD44-2 group for *CD44* gene knockdown. To evaluate the transfection efficiency of TMEM161B-AS1 silencing vector in U87 cells and U251 cells, cells were divided into Control group, si-NC group and si-TMEM161B-AS1 group. To evaluate the role of hsa-miR-27a-3p in the malignant biological behavior of TMEM161B-AS1 silenced U87 cells and U251 cells, cells were divided into 4 groups, namely NC group, si-TMEM161B-AS1 + hsa-miR-27a-3p antagomir group, hsa-miR-27a-3p antagomir group and si-TMEM161B-AS1 group. To evaluate whether hsa-miR-27a-3p and *FANCD2* gene or hsa-miR-27a-3p and *CD44* gene interact at the predicted binding sites, we divided HET-293T cells into NC group and hsa-miR-27a-3p agomir group. To evaluate the effect of hsa-miR-27a-3p silencing on the malignant biological behavior and ferroptosis of U87 cells and U251 cells, cells were divided into NC group, hsa-miR-27a-3p agomir group, si-FANCD2 group, si-CD44 group, hsa-miR-27a-3p agomir+p-FANCD2 group, hsa-miR-27a-3p agomir+p-CD44 group. In order to evaluate the role of TMEM161B-AS1 and hsa-miR-27a-3p in the growth of gliomas, U87 cells and U251 cells were divided into NC group, si-TMEM161B-AS1 group, hsa-miR-27a-3p angomir group, si-TMEM161B-AS1 + hsa-miR-27a-3p angomir group.

### Dual luciferase reporter assay

lncRNA TMEM161B-AS1, *FANCD2* 3’UTR or *CD44* 3’UTR fragments containing the predicted wild-type (wt) or mutant (mut) hsa-miR-27a-3p binding sites were synthesized by RiboBio Co., Ltd.(Guangzhou, China) and cloned into the XbaI and SalI sites of the downstream of Renilla luciferase gene in the vector pmirGLO (Promega, Madison, WI, USA). Approximately 1×10^4^ HEK-293T cells were plated into 96-well plates and co-transfected with 50 nM TMEM161B-AS1-wt (or TMEM161B-AS1-mut) or FANCD2-wt (or FANCD2-mut) or CD44-wt(CD44-mut) and 50 nM hsa-miR-27a-3p agomir or Blank vector pmirGLO (NC, Promega, Madison, WI, USA) by Lipofectamine 3000 (Invitrogen, Life Technologies, Carlsbad, CA). 24 h after transfection and the luciferase activity was measured using the dual luciferase reporter assay system (Promega, Madison, WI, USA) according to the manufacturer’s instruction.

### Cell Counting Kit-8

2×10^3^ U87 and U251 cells were seeded in 96-well plates, after 48 h incubation, 20 μl of Cell Counting Kit-8 (Beyotime Institute of Biotechnology, Jiangsu, China) was added. Cells were incubated for 2 h at 37°C and the absorbance was detected at 450 nm (OD_450_).

### Transwell assay

Transfected U87 or U251 cells were harvested after 24 h transfection. The cell migration and invasion abilities were determined by Transwell assay. For migration ability detection, 2× 10^5^ U87 and U251 cells were plated in the upper chamber of Transwell assay inserts (Millipore, Billerica, MA, USA) containing 200 μl of serum-free DMEM with a membrane (8-mm pores). For the invasion assay, the transfected cells were plated in the top chamber with a Matrigel-coated membrane (BD Biosciences, Franklin Lakes, NJ, USA) in 500 μl serum-free DMEM with 750 μl 10% FBS-DMEM in the bottom chamber. Then, the inserts were placed into the wells of the bottom chamber of a 24-well plate filled with conditioned medium. The lower surface of the membrane was fixed with methanol and glacial acetic acid (v/v: 3:1) and stained with crystal violet after 24 h of incubation. Cell numbers were calculated in 5 random fields after photographed with a digital microscope (×200).

### Apoptosis detection assay

U87 cells and U251 cells apoptosis were evaluated by Annexin V-FITC/PI staining (BD Biosciences, San Jose, CA, USA) followed by flow cytometry (FACScan, BD Biosciences). After being washed three times with PBS, U87 cells and U251 cells were harvested in binding buffer at a concentration of 1×10^6^ cells/ml, and 5 μl of PI and 5 μl FITC were added to the cell suspension and incubated for 15 min at room temperature in the dark condition.

### Western blot

The collected cells were lysed with RIPA buffer (Beyotime Institute of Biotechnology) for 0.5 h on ice, and centrifuged at 15,000 g at 4°C for 45 min. BCA protein assay kit (Beyotime Institute of Biotechnology, Jiangsu, China) was used to detect the concentration of extracted protein. After denaturation, the protein samples were separated by 15% sodium dodecyl sulfate-polyacrylamide gel electrophoresis (SDS-PAGE) membrane and transferred to polyvinylidene fluoride (PVDF) membrane. Membranes were blocked by TBST buffer (0.05% Tween 20, 0.15 M NaCl, 50 mM Tris-HCI, pH 7.5) containing 5% non-fat milk for 2 h at room temperature and then incubated with primary antibodies as follows: FANCD2 (1:2000, Abcam, Cambridge, MA, USA), CD44 (1:1000, Abcam, Cambridge, MA, USA) and β-actin (1:2000, Abcam, Cambridge, MA, USA) overnight at 4°C. Membranes were then washed three times with TBST and incubated with horseradish peroxidase-labeled goat anti-rabbit IgG (1:2000, Abcam, Cambridge, MA, USA) for 2 h at room temperature. After adding developer for 1 min, the immunoblots were analyzed by the ImageJ2X software (National Institutes of Health, Bethesda, MD, USA), and the relative integrated density values (IDVs) were calculated using FluorChem FC2 system (Alpha Innotech, San Leandro, CA, USA) based on β-actin as an internal control.

### RNA immunoprecipitation

The Magna RIP RNA Binding Protein Immunoprecipitation Kit (Millipore, Billerica, MA, USA) was used to performed the RNA immunoprecipitation (RIP) assay according to the manufacturer’s protocol. Cells were lysed by RIP lysis buffer and 0.1 ml U87 cells or U251 cells extract were incubated with RIPA buffer containing magnetic beads conjugated with human anti-Argonaute2 (Ago2) antibody (Abcam, Cambridge, MA, USA) at 4°C for 8 h. Then washing buffer was used to wash the samples and isolated the RNA–protein complexes from beads by proteinase K for 0.5 h at 55°C. Then the RNA was extracted and analyzed by qRT-PCR.

### Caspase 3/7 activity measurements

U87 cells and U251 cells were collected and incubated for 1 h in the dark with 100 μL Caspase-Glo 3/7 reagent. The absorbance at 485/530 nm was detected by a microplate reader.

### Lipid ROS measurement

The details of the procedures had been previously described [39, 55, 56]. U87 cells and U251 cells were collected and resuspended in DMEM with 10% FBS, then 10 μM C11 BODIPY (Thermo Fisher, Bonn, Germany) was added and incubated for 0.5 h at 37°C, 5% CO_2_ in the dark. Then washed the cells with PBS for three times to remove the excess C11 BODIPY. The level of lipid ROS was proportional to the change in fluorescence emission peak from 590 nm to 510 nm caused by oxidation of the polyunsaturated butadienyl moiety of the dye. This analysis was performed by a flow cytometry.

### Iron measurement

The total iron level in U87 cells and U251 cells were detected by Iron Assay Kit (Sigma, Missouri, USA) according to the manufacturer’s protocol. The details of the procedures had been previously described [39, 55, 56].

### Tumor xenograft assay

In this study, 4-week-old specific pathogen free male nude mice were selected and assigned into 4 groups, each of which was respectively injected with U251 cells and U87 cells transfected with NC, hsa-miR-27a-3p angomir, si-TMEM161B-AS1, si-TMEM161B-AS1 + hsa-miR-27a-3p angomir. Each nude mouse was subcutaneously injected with 4×10^5^ cells in the right flank area for subcutaneous implantation. Tumors volume were measured 45days after injection, according to the formula: volume (mm^3^) = length × width^2^/2. Then the nude mice were sacrificed and tumors were isolated. All mice were randomly assigned to each experimental group.

### Statistical analysis

Data were presented as means ± standard deviation (mean ± SD). All statistical analyses were performed by the Prism 8.0 GraphPad software (GraphPad Software, La Jolla, CA, US). All experiments were repeated at least three times and the differences were analyzed by one-way ANOVA or the Student’s t-test (two tailed). Differences were considered as statically significant when *p* < 0.05.

## Supplementary information

Figure S1

Figure S2

Supplementary legends

Supplemental materials

Supplemental Table S1

All authors responded to the email confirming their agreement to these changes

## Data Availability

The datasets used and/or analyzed during the current study are available from the corresponding author on reasonable request.
