# Supplementary figures and images for: Over-expression of lncRNA TMEM161B-AS1 promotes the malignant biological behavior of glioma cells and the resistance to temozolomide via up-regulating the expression of multiple ferroptosis-related genes by sponging hsa-miR-27a-3p
Source: Cell Death Discov. 2021 Oct 23;7:311. doi: 10.1038/s41420-021-00709-4 (PMC8542043; doi:10.1038/s41420-021-00709-4)

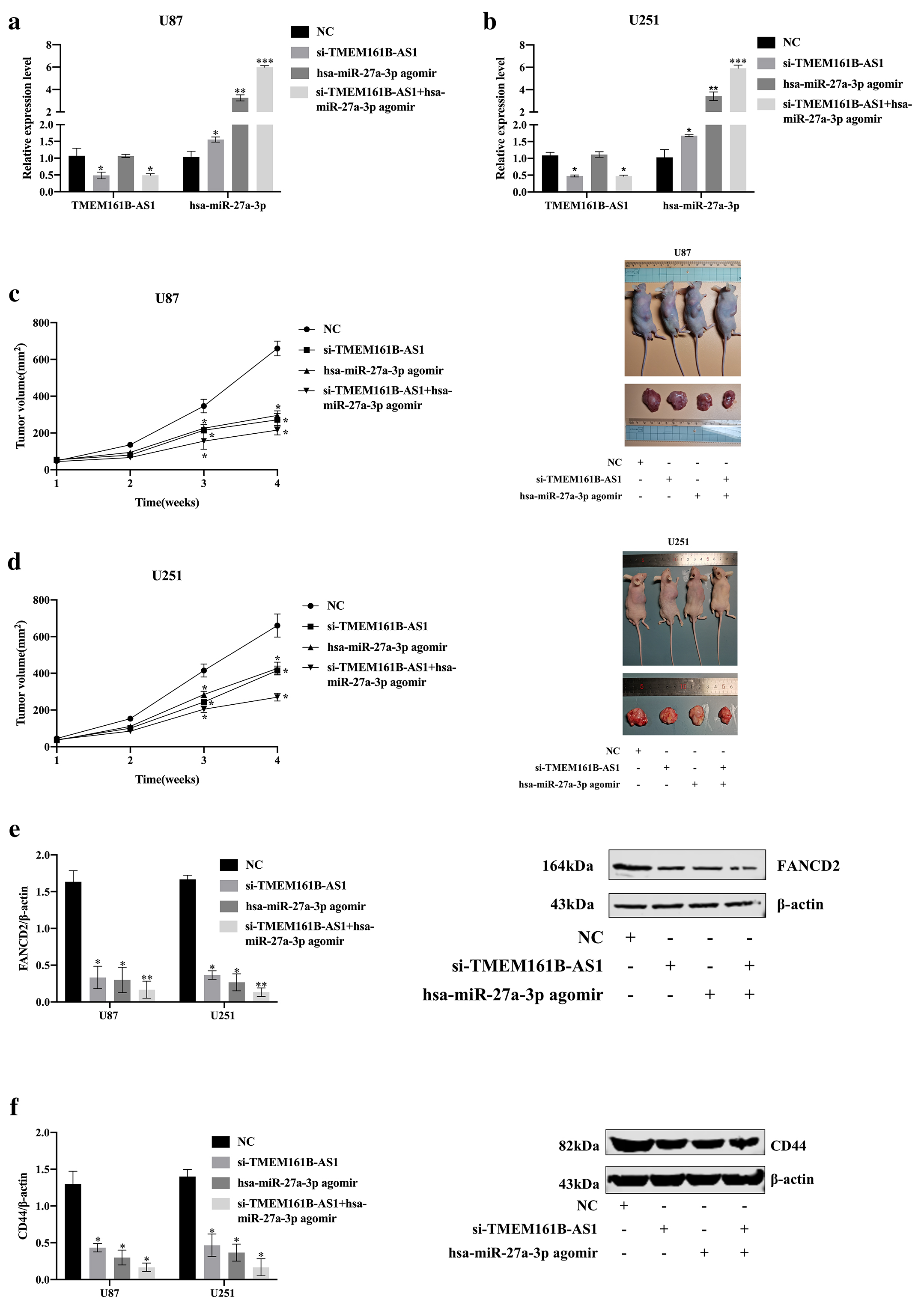

Supplement: Supplementary file 1 — Figure S1 [file 41420_2021_709_MOESM1_ESM.jpg]

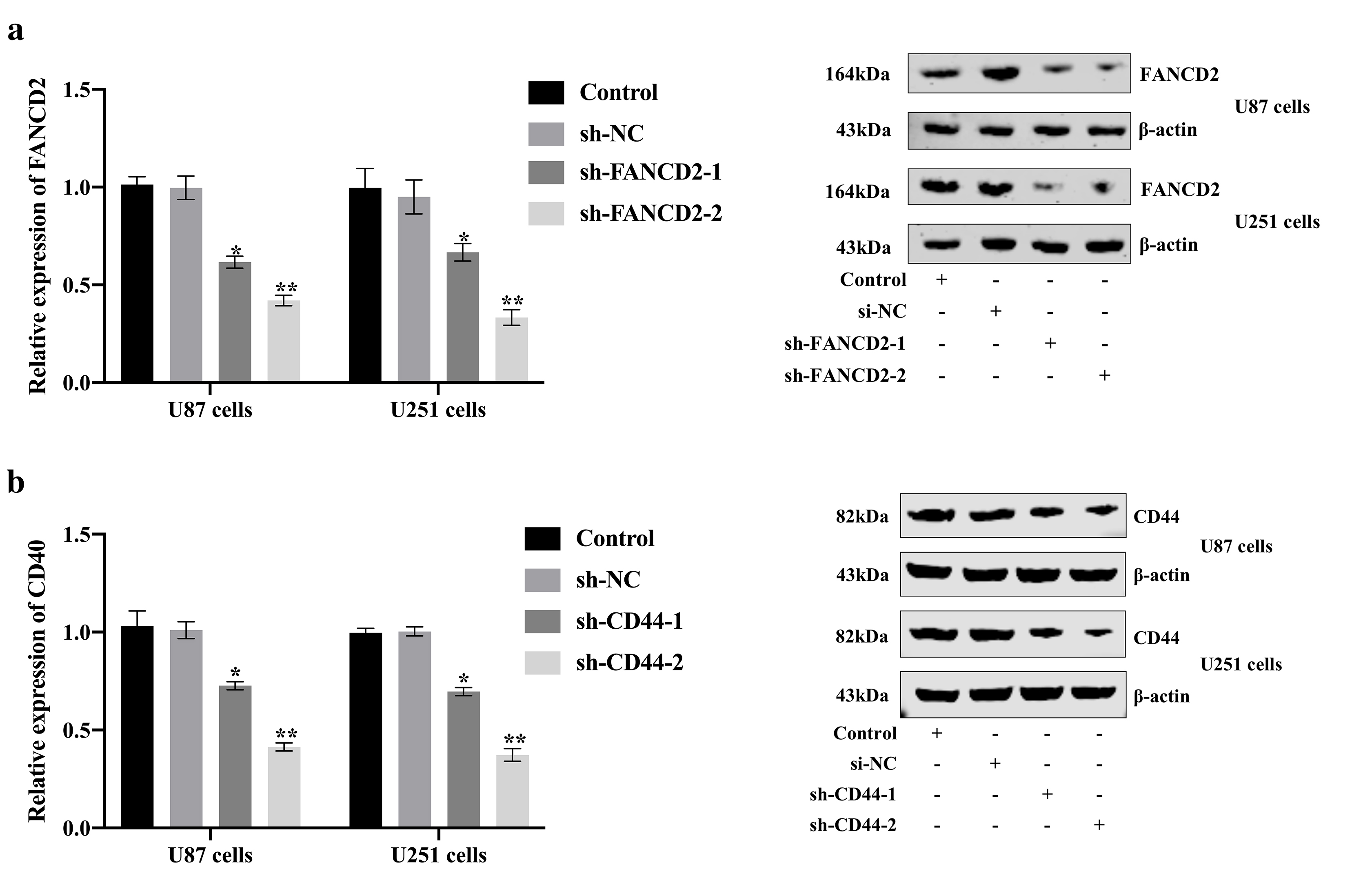

Supplement: Supplementary file 2 — Figure S2 [file 41420_2021_709_MOESM2_ESM.jpg]
