# Supplementary material for: Over-expression of lncRNA TMEM161B-AS1 promotes the malignant biological behavior of glioma cells and the resistance to temozolomide via up-regulating the expression of multiple ferroptosis-related genes by sponging hsa-miR-27a-3p
Source: Cell Death Discov. 2021 Oct 23;7:311. doi: 10.1038/s41420-021-00709-4 (PMC8542043; doi:10.1038/s41420-021-00709-4)
Supplement: Supplementary file 3 — Supplementary legends [file 41420_2021_709_MOESM3_ESM.doc]

**Figure S1** the expression of FANCD2, CD44 after cells transfection with two short-harpin plasmids of FANCD2 (sh-FANCD2-1, sh-FANCD2-2) (a), CD44 (sh-CD44-1, sh-CD44-2) (b). Data are presented as the mean±SD (n=3, each group). *p<0.05 vs. sh-NC group. **p<0.01 vs. sh-NC group.

**Figure S2** Silencing TMEM161B-AS1 and/or hsa-miR-27a-3p overexpression inhibited the growth of glioma in nude mice. **a**, **b**. The expression level of TMEM161B-AS1 and hsa-miR-27a-3p in U87 cells and U251 cells transfected with no template control (NC), si-TMEM161B-AS1, hsa-miR-27a-3p angomir, si-TMEM161B-AS1+hsa-miR-27a-3p angomir were detected by qRT-PCR. **c, d**. Comparison of tumor volume in nude mice subcutaneously implanted tumor models after transfection of U87 and U251 cells with NC, si-TMEM161B-AS1, hsa-miR-27a-3p angomir, and si-TMEM161B-AS1+ hsa-miR-27a-3p angomir. **e, f**. The FANCD2 protein and CD44 protein expression level in NC, si-TMEM161B-AS1, hsa-miR-27a-3p angomir, si-TMEM161B-AS1+ hsa-miR-27a-3p angomir transfected U87 and U251 cells. *p<0.05, compared with NC, **p<0.01, compared with NC, ***p<0.001, compared with NC. n=3 per group.
