# Supplementary material for: Over-expression of lncRNA TMEM161B-AS1 promotes the malignant biological behavior of glioma cells and the resistance to temozolomide via up-regulating the expression of multiple ferroptosis-related genes by sponging hsa-miR-27a-3p
Source: Cell Death Discov. 2021 Oct 23;7:311. doi: 10.1038/s41420-021-00709-4 (PMC8542043; doi:10.1038/s41420-021-00709-4)
Supplement: Supplementary file 4 — Supplemental materials [file 41420_2021_709_MOESM4_ESM.pdf]

gene_id	log2FoldChange	log2CPM	PValue	FDR	Up/down
HOXD10	11.47032961	2.902151542	1.34E-10	1.30E-09	Up
C6orf15	10.8945637	2.330157893	0.000217387	0.000750693	Up
PRAME	10.2291218	3.01370723	0.000158704	0.000564346	Up
HOXC11	10.19379273	1.637848591	9.16E-07	5.06E-06	Up
NKX2-5	9.938408756	2.800241387	1.43E-09	1.21E-08	Up
HOXD13	9.905019806	2.772247155	3.14E-07	1.88E-06	Up
LINC0273[illegible]	9.888775191	1.336507986	2.24E-12	2.72E-11	Up
HOXD9	9.746222687	2.560635553	3.17E-10	2.92E-09	Up
[illegible]OXD-AS[illegible]	9.624040606	1.075883471	1.31E-12	1.64E-11	Up
HOXA11	9.480364982	0.92936184	3.58E-07	2.12E-06	Up
HOXC10	9.4073351	3.481056805	2.82E-08	1.98E-07	Up
[illegible]DAMDEC[illegible]	9.396136717	2.245153348	8.31E-09	6.26E-08	Up
HOXA2	9.238762718	2.051519638	1.19E-07	7.60E-07	Up
HOXA3	9.172284108	2.692629505	2.26E-10	2.12E-09	Up
CLVS1	-2.000418277	2.052271763	0.000685487	0.002105043	Down
[illegible]LOX15P1	-2.000544546	-2.793947349	2.74E-05	0.000114792	Down
HOXA5	9.158794315	3.182592888	3.27E-12	3.92E-11	Up
ADGRG7	9.110216284	0.5760642	0.000723578	0.002210471	Up
HOTAIR	9.033771949	1.888796117	5.08E-06	2.44E-05	Up
HOXA10	9.016010911	3.018941975	8.49E-10	7.36E-09	Up
HP	9.00190778	4.268918375	3.18E-06	1.60E-05	Up
HOXD11	8.926098824	1.807886146	1.20E-08	8.86E-08	Up
[illegible]OXA-AS[illegible]	8.909233715	0.379713185	8.03E-10	6.99E-09	Up
GRK6P1	-2.000732284	-1.963212955	1.57E-13	2.17E-12	Down
HOXA7	8.895554815	1.767483185	5.96E-08	3.97E-07	Up
HOXC13	8.877547175	1.686291896	1.75E-05	7.62E-05	Up
HOXA6	8.828334884	0.302462517	4.80E-09	3.75E-08	Up
SAA1	8.58748597	5.350617805	8.92E-05	0.000336098	Up
SHOX2	8.579923454	4.012614832	5.51E-16	9.97E-15	Up
[illegible]OXA-AS[illegible]	8.565055693	2.060081709	1.67E-08	1.21E-07	Up
HOXA4	8.537565641	2.074341075	3.02E-07	1.81E-06	Up
[illegible]OXC-AS1	8.51013988	-0.003010188	1.71E-05	7.46E-05	Up
[illegible]OXC13-A[illegible]	8.487489447	-0.029263732	0.000590747	0.001844779	Up
HOXB4	8.478179155	2.468287925	3.54E-06	1.76E-05	Up
[illegible]OXC-AS[illegible]	8.423851123	-0.087583405	6.04E-07	3.44E-06	Up
EN1	8.388273561	3.068837427	9.21E-10	7.96E-09	Up
HOXD8	8.376281895	2.790673312	2.40E-08	1.70E-07	Up
LTF	8.369303908	9.266303175	7.54E-05	0.00028815	Up
HOXA9	8.327416927	1.231358221	2.38E-05	0.000100606	Up

GATA4	8.306209994	1.145409968	0.000102779	0.000381831	Up
HR	-2.003004984	4.481345304	5.58E-07	3.20E-06	Down
TRPM1	-2.003019637	-2.707627005	0.000458438	0.00146739	Down
TBX5	8.11789731	2.107418659	0.000396645	0.001287998	Up
PI3	8.087992831	4.50249741	0.000250311	0.000854228	Up
PITX1	7.976742213	2.87769974	1.32E-07	8.38E-07	Up
H3C8	7.861959763	-0.647088437	1.46E-06	7.79E-06	Up
HOXB7	7.836422859	2.720494483	3.71E-08	2.56E-07	Up
TFAP2B	7.705847264	0.588042481	0.000306554	0.00102329	Up
LSM11	-2.003315978	3.097950252	1.41E-30	1.03E-28	Down
HOXC9	7.700803939	1.264100221	7.88E-07	4.39E-06	Up
AC078991.[illegible]	7.671559846	-0.79251053	6.37E-15	1.02E-13	Up
MYBL2	7.633219716	5.541850543	5.03E-18	1.11E-16	Up
FOXD3-AS	7.594597434	2.4740994	1.59E-12	1.96E-11	Up
AL049830.1	7.445528434	-0.997743629	3.38E-15	5.54E-14	Up
FP671120.6	7.423039455	2.114987208	0.000997016	0.002946465	Up
AC093895.[illegible]	7.394987755	0.310631674	5.37E-06	2.58E-05	Up
HAND2	7.38997945	1.766277944	1.58E-05	6.91E-05	Up
H19	7.32265482	5.59997907	0.000207027	0.000718448	Up
UBE2C	7.300646051	5.227823196	1.60E-20	4.53E-19	Up
POSTN	7.279238063	7.843603168	7.01E-05	0.000269884	Up
CHCHD2P	7.274229415	-1.166681281	2.84E-08	1.99E-07	Up
MMP9	7.262700807	5.619372741	1.72E-07	1.07E-06	Up
HOXB3	7.245359326	4.125958407	1.45E-07	9.14E-07	Up
RRM2	7.243248768	5.806394188	1.01E-26	5.19E-25	Up
PBK	7.227123218	4.843976587	4.52E-21	1.36E-19	Up
ZFYVE28	-2.005050957	3.682543555	2.52E-19	6.35E-18	Down
TOP2A	7.221099036	6.866538641	9.10E-20	2.41E-18	Up
MNX1	7.19833811	0.113505734	0.000114675	0.000420924	Up
HOXC8	7.182523681	0.712783263	2.35E-06	1.21E-05	Up
HOXA11-A	7.166236544	0.067611178	1.32E-05	5.90E-05	Up
AL606970.[illegible]	7.149818544	-1.27652393	5.30E-05	0.000208933	Up
GSC	7.084849885	1.443568795	1.68E-11	1.84E-10	Up
DLGAP5	7.082030129	3.727500606	6.35E-21	1.87E-19	Up
SAA2	7.066782401	3.201816453	0.000788593	0.002388072	Up
OTP	7.042837467	2.44253739	4.13E-07	2.43E-06	Up
TLX1	7.022397134	0.621043615	0.000517645	0.001635463	Up
PLA2G2A	7.000967512	5.966069127	0.00056058	0.00175973	Up
AC098591.[illegible]	6.989234455	-1.406709344	4.60E-15	7.44E-14	Up
HOXC-AS[illegible]	6.941297286	-1.456604631	3.27E-05	0.000134486	Up

HOXA1	6.904297453	0.946806957	4.12E-11	4.32E-10	Up
AC034207.1	6.898468607	-1.478012607	1.96E-14	2.97E-13	Up
RPS11P7	6.895438067	-1.491930685	4.03E-13	5.36E-12	Up
MELK	6.825098177	4.119813859	1.45E-18	3.40E-17	Up
HOXC6	6.799347732	1.645271633	6.83E-08	4.51E-07	Up
RPS3AP7	6.796122722	-1.583228162	1.47E-11	1.63E-10	Up
AL590135.1	6.77937734	-1.597839011	1.70E-10	1.63E-09	Up
DPEP1	6.778001822	3.240873057	8.83E-09	6.63E-08	Up
TCEAL2	-2.005967719	5.287220174	9.63E-07	5.29E-06	Down
FP236383.3	6.769731958	-1.693477382	0.000801277	0.002423188	Up
LINC01198	6.758007176	1.362012172	7.86E-05	0.000299353	Up
SNRPD2P1	6.754278988	-1.610692808	8.20E-13	1.05E-11	Up
POTEF	6.726130303	-0.351278685	3.43E-26	1.68E-24	Up
IL21-AS1	6.721216847	-1.658797456	4.63E-05	0.00018448	Up
HOXB5	6.694546372	0.284871974	0.000829698	0.002502819	Up
HOXD4	6.674587573	-0.394999561	1.11E-06	6.05E-06	Up
NKX3-2	6.673573765	0.674250456	2.27E-07	1.39E-06	Up
HOXB9	6.658531658	0.248998777	0.000145728	0.000523162	Up
H2AZP2	6.638773396	-1.714664881	1.02E-13	1.44E-12	Up
KIF20A	6.626360776	4.214482625	8.88E-23	3.17E-21	Up
AURKB	6.590011383	3.953385291	1.78E-18	4.12E-17	Up
HOXB6	6.566080692	0.204708628	7.74E-05	0.000295209	Up
SCNN1B	6.544628924	2.588881394	5.04E-05	0.000199519	Up
E2F8	6.512964569	1.966554769	4.85E-19	1.19E-17	Up
FOXD3	6.494952199	1.16000903	2.91E-06	1.47E-05	Up
AL133163.1	6.492333063	-1.834457119	1.49E-12	1.84E-11	Up
HAND2-AS	6.421263011	-0.008171892	0.000606174	0.001886412	Up
SRIP3	6.418574532	-1.902914095	2.16E-11	2.34E-10	Up
HOXB2	6.40866128	3.424807438	4.32E-06	2.11E-05	Up
AC004057.1	6.38392486	0.425102165	8.78E-14	1.25E-12	Up
H3P16	6.369115284	0.763386636	6.68E-28	3.75E-26	Up
TROAP	6.355749154	4.02364648	4.32E-18	9.64E-17	Up
HOXA13	6.355636508	1.662397913	0.000110179	0.000406025	Up
LINC01956	6.348012159	-0.088685983	2.50E-06	1.28E-05	Up
AC074131.1	-2.005989741	-2.861580431	1.82E-06	9.53E-06	Down
CA3	6.275939956	5.727957897	0.000101233	0.000376574	Up
H2AC18	6.274497302	-2.039748624	1.03E-06	5.62E-06	Up
LINC01587	6.265455117	-0.815814409	1.13E-06	6.16E-06	Up
BIRC5	6.261159133	6.02366096	5.07E-16	9.24E-15	Up
LINC02783	6.256739984	-2.048547668	0.000335549	0.0011089	Up

FCAB6-AS	-2.007248106	-2.990146679	2.50E-05	0.000105276	Down
KRT17P7	6.233046909	-2.073045204	0.000947432	0.002815231	Up
TUBBP6	6.221833799	-0.133797362	0.000278038	0.00093876	Up
SNRPFP1	6.216583472	-2.076482188	2.33E-12	2.84E-11	Up
Z99714.1	6.209563162	-2.08104054	6.56E-09	5.03E-08	Up
IGF2BP3	6.203276379	3.693307172	1.72E-11	1.89E-10	Up
LINC0230	6.196208312	0.648461846	1.06E-08	7.86E-08	Up
SNRPGP1	6.18491904	2.615574475	1.76E-29	1.16E-27	Up
BTF3P4	6.184285586	-2.093588164	1.83E-11	2.00E-10	Up
LINC0138	6.170412324	-2.127672146	0.000341133	0.00112516	Up
RPL10P5	6.167045633	-2.107518548	2.08E-10	1.97E-09	Up
H2AC19	6.161481645	-2.13190163	3.95E-07	2.33E-06	Up
HK1	-2.008106235	7.027108417	1.20E-22	4.22E-21	Down
AC015911.	6.153958461	-2.128676038	2.98E-08	2.08E-07	Up
HOXC5	6.147437281	-2.141156522	5.86E-05	0.000228923	Up
IDO1	6.143964363	1.064287241	0.000185162	0.000649215	Up
AC079328.	6.133038593	-0.909638892	1.31E-19	3.41E-18	Up
BARHL1	6.132345457	-0.927653396	3.95E-06	1.95E-05	Up
AC020905.	6.122342061	-2.164768153	1.65E-05	7.22E-05	Up
LINC01571	6.111372121	-0.961399815	4.57E-06	2.22E-05	Up
AC011933.	6.108587276	-2.155313271	9.92E-11	9.83E-10	Up
AC012005.	6.099071627	-2.182790974	4.48E-07	2.61E-06	Up
AC010904.	6.092344533	-2.167767218	6.08E-11	6.21E-10	Up
CHI3L1	6.088420614	12.10281853	1.35E-05	6.00E-05	Up
EVX2	6.085612908	-0.984973024	6.15E-06	2.92E-05	Up
H2BC9	6.07024476	-0.333214269	7.53E-08	4.95E-07	Up
PIMREG	6.06339058	4.969654804	4.59E-14	6.72E-13	Up
MEOX2	6.057114978	5.172325568	8.87E-07	4.91E-06	Up
AC093155.	6.034632349	-2.208365653	1.66E-11	1.83E-10	Up
NDC80	6.029356706	3.924631938	8.05E-21	2.35E-19	Up
CMTM4	-2.008251621	5.668249558	5.32E-13	6.95E-12	Down
AC025181.	6.021352327	-2.224454388	7.67E-10	6.70E-09	Up
RPS2P2	6.017669456	-2.232215683	1.02E-10	1.01E-09	Up
RPL32P31	6.013759086	-2.235004642	3.36E-10	3.08E-09	Up
NT5DC1	-2.008694654	3.595271638	5.40E-11	5.56E-10	Down
RPLP0P6	6.00416299	5.355179615	1.15E-32	1.02E-30	Up
LINC0134	5.998330122	-2.263962521	0.000213066	0.000737364	Up
COL3A1	5.981885775	9.178228514	7.04E-05	0.000271081	Up
AC025211.	5.977885854	-2.27938636	1.09E-05	4.92E-05	Up
CENPA	5.974991218	2.921563285	7.37E-19	1.78E-17	Up

AC090692.1	5.967712869	2.378871927	3.58E-09	2.85E-08	Up
AL031229.1	5.956418963	-2.2770974	1.85E-09	1.54E-08	Up
RPL12P2	5.949691613	-2.281010397	1.71E-08	1.23E-07	Up
HOTAIRM	5.942549236	3.176282268	4.93E-09	3.84E-08	Up
RPL10P13	5.925095256	-2.307557285	7.67E-09	5.81E-08	Up
NCAPG	5.922352704	4.162818334	5.94E-20	1.60E-18	Up
HJURP	5.919595512	3.981470416	5.47E-19	1.34E-17	Up
RPL18P11	5.902432384	-2.317995802	1.39E-09	1.18E-08	Up
AL590640.2	5.900343472	-2.32488217	1.40E-09	1.18E-08	Up
AL118558.4	-2.008913885	-1.164786491	2.82E-10	2.62E-09	Down
AC007344.1	5.888528263	-0.469394293	3.72E-05	0.000151204	Up
FAM111B	5.867669536	3.327173484	4.89E-14	7.15E-13	Up
RPL5P22	5.866468489	-1.144724251	6.88E-13	8.87E-12	Up
EEF1A1P4	5.858818448	-2.359836899	2.38E-09	1.95E-08	Up
AC096915.1	5.858135878	-2.352722482	1.91E-09	1.58E-08	Up
AC005521.1	5.849602243	-2.365517608	2.10E-10	1.99E-09	Up
RPS3AP51	5.838201781	-2.379104573	1.43E-07	9.01E-07	Up
AKR1B1P7	5.833951842	-2.378813368	2.63E-10	2.46E-09	Up
PLEKHS1	5.829836331	0.941124931	1.19E-05	5.34E-05	Up
ASF1B	5.816370396	4.562067171	1.89E-18	4.35E-17	Up
IL2RA	5.811716466	2.737360567	0.000412881	0.001335513	Up
IGFBP2	5.805368002	9.062023435	3.60E-12	4.29E-11	Up
AC008073.3	-2.010980305	-2.930446664	3.31E-06	1.65E-05	Down
RPS15AP5	5.801619242	-2.402621394	9.49E-09	7.09E-08	Up
RPS18P12	5.795278183	2.70958892	1.61E-20	4.55E-19	Up
IBSP	5.792577626	4.185384649	3.59E-05	0.000146427	Up
MKI67	5.776136898	5.522696579	7.02E-13	9.03E-12	Up
HNRNPA1P	5.768457127	-2.438663357	5.00E-10	4.47E-09	Up
CFAP77	5.755734179	1.923888431	1.74E-06	9.20E-06	Up
BPIFB2	5.754404136	-0.150669884	0.000234706	0.000806153	Up
CBX3P1	5.714075724	-2.478871392	4.97E-09	3.88E-08	Up
RHOBTB2	-2.01128148	5.286288038	6.32E-21	1.87E-19	Down
RPSAP2	5.706072145	-2.474072576	2.86E-09	2.30E-08	Up
METTL7B	5.702609735	7.168588285	1.41E-07	8.92E-07	Up
LINC01111	5.693640499	-2.506161184	0.000912629	0.002723331	Up
AL713998.1	5.684759638	-2.509614975	4.91E-06	2.37E-05	Up
H4C15	5.68323183	-2.509937037	9.13E-07	5.04E-06	Up
LINC0046	5.676406824	-2.514126403	0.000243867	0.000835427	Up
AL132777.1	5.669662841	-2.516008879	6.01E-08	4.01E-07	Up
LDHAP1	5.664722737	-2.517982462	2.69E-08	1.89E-07	Up

RPL30P4	5.662333724	-0.679561626	7.84E-17	1.55E-15	Up
E2F2	5.660333468	3.201460776	8.17E-13	1.04E-11	Up
AC023232.1	5.65953476	-2.506662249	2.15E-09	1.77E-08	Up
AC007690.1	5.653029326	-2.516100011	1.57E-08	1.14E-07	Up
LINC0114[illegible]	5.643893384	-2.541372888	0.000145742	0.000523162	Up
CDC45	5.642358713	3.660111738	1.17E-15	2.04E-14	Up
LINC0258[illegible]	5.639075877	2.8183485	2.33E-05	9.89E-05	Up
LINC0205[illegible]	5.634759331	-2.543947392	0.000271163	0.000918131	Up
AL139095.[illegible]	5.630812541	1.946052417	7.03E-22	2.30E-20	Up
MSNP1	5.620858047	0.308536262	2.20E-16	4.17E-15	Up
AC026410.[illegible]	5.614570979	-2.549986919	1.91E-07	1.18E-06	Up
AL163973.1	5.6102014	-2.554599004	2.19E-07	1.34E-06	Up
GPR82	5.606423731	1.976369064	1.79E-11	1.96E-10	Up
AC010677.[illegible]	5.603992558	-2.555531499	3.84E-08	2.64E-07	Up
CTAGE3P	-2.011880082	-3.145777593	3.88E-07	2.29E-06	Down
PRR32	5.595243167	0.020784031	7.78E-06	3.62E-05	Up
AL035446.1	5.587268891	1.785941775	2.19E-05	9.32E-05	Up
CDCA2	5.570878122	2.767314669	9.92E-16	1.73E-14	Up
AC018553.[illegible]	5.570507395	-1.436910801	4.90E-06	2.36E-05	Up
AL357084.1	5.562971075	-2.588520968	1.13E-06	6.15E-06	Up
AL354919.[illegible]	5.560578169	1.81154275	2.01E-06	1.04E-05	Up
TMSB15A	5.559864814	3.652567268	0.00010592	0.000392286	Up
YBX1P6	5.556442518	-0.794605197	3.82E-19	9.48E-18	Up
AL445363.[illegible]	5.55221055	-2.595410394	3.06E-08	2.13E-07	Up
HMGN2P1[illegible]	5.546392818	-2.605824218	5.69E-08	3.81E-07	Up
PAX3	5.538324327	1.825701173	0.000372011	0.001216285	Up
HMGN2P2	5.535781898	-2.610705625	5.51E-08	3.69E-07	Up
AC098590.[illegible]	5.526096565	-1.448248826	7.25E-17	1.43E-15	Up
IRX5	5.51832879	1.537078763	2.20E-07	1.34E-06	Up
RASGRP1	-2.012743607	4.046203427	1.63E-07	1.02E-06	Down
CLIC1P1	5.515475187	-0.834422598	3.76E-14	5.56E-13	Up
HSBP1P2	5.497583374	-2.623388235	3.06E-08	2.13E-07	Up
AL353151.1	5.496399303	-1.486676818	9.42E-11	9.35E-10	Up
RPS23P2	5.496040928	-2.631780612	3.75E-08	2.58E-07	Up
KC877373.[illegible]	5.49282439	-2.635977621	2.17E-08	1.54E-07	Up
AL138878.[illegible]	5.491180881	-2.646161154	7.38E-08	4.86E-07	Up
BTF3P2	5.487002229	-2.651832917	8.52E-09	6.40E-08	Up
SEPTIN2P[illegible]	5.482369245	-1.492752686	2.60E-14	3.91E-13	Up
RPS29P11	5.467929559	-2.657734928	1.47E-07	9.24E-07	Up
AL133415.1	5.462538524	2.071719541	6.40E-21	1.88E-19	Up

RPL23P5	5.451738066	-2.660166641	2.74E-08	1.92E-07	Up
AL590004.2	5.449665682	-2.665669272	8.41E-08	5.48E-07	Up
POTEKP	5.441044344	-0.11198518	1.57E-21	4.96E-20	Up
WARS2-IT1	5.430791789	0.699322333	4.42E-13	5.83E-12	Up
RPL36AP13	5.421141909	-2.690873337	7.44E-07	4.18E-06	Up
LINC01349	5.41345278	-2.713253575	0.000145877	0.000523517	Up
AL669983.1	5.409312256	-0.492915261	9.10E-16	1.60E-14	Up
KIF14	5.408287973	2.982762574	4.13E-16	7.62E-15	Up
AC090587.2	-2.01278425	-1.723731424	1.89E-11	2.07E-10	Down
AC104212.2	5.402830698	-1.565750025	7.36E-11	7.43E-10	Up
PTPRN2	-2.013604289	6.435274405	3.93E-14	5.80E-13	Down
EGFR	5.399219582	10.70047636	5.76E-05	0.000225567	Up
RPS15AP24	5.394895538	0.101880714	1.01E-18	2.39E-17	Up
NNMT	5.388234715	6.858228858	7.44E-06	3.48E-05	Up
TMEM35A	-2.014027181	4.229891417	1.49E-07	9.35E-07	Down
H4C14	5.375429779	-1.596762794	1.40E-09	1.18E-08	Up
AC019129.1	5.371730727	-2.730431569	3.89E-07	2.29E-06	Up
KIFC1	5.368512032	4.725830015	5.53E-15	8.89E-14	Up
AC133134.1	5.366559333	-0.540119493	4.99E-17	1.01E-15	Up
MAGED4B	5.362467477	1.175078518	3.41E-21	1.03E-19	Up
AC092418.1	5.358900685	-2.737298704	1.14E-06	6.20E-06	Up
AL512785.1	5.358203616	0.299848018	1.35E-05	6.03E-05	Up
TMEM121B	-2.015810075	3.555931228	1.14E-11	1.28E-10	Down
IDI2-AS1	-2.01660991	-0.684125539	1.18E-05	5.31E-05	Down
SNRPEP4	5.357642337	1.283942681	1.49E-08	1.08E-07	Up
HOXB-AS1	5.357351424	0.91972493	1.73E-05	7.54E-05	Up
LHX9	5.352896224	2.493794876	2.11E-05	9.02E-05	Up
RPL7P58	5.347183528	-2.748588161	2.99E-07	1.79E-06	Up
HOXD12	5.333063168	-1.614896705	0.00019371	0.00067639	Up
NUSAP1	5.328682605	5.757863425	1.70E-18	3.95E-17	Up
RPL34P27	5.323079848	-0.568656986	4.69E-13	6.17E-12	Up
TREML3P	5.322523224	-0.57417267	2.00E-05	8.61E-05	Up
H3P24	5.322124165	-2.76565725	4.15E-08	2.83E-07	Up
MFFP2	5.316760234	-1.654108156	2.15E-10	2.03E-09	Up
AL451081.2	5.316023365	-0.581420195	3.29E-15	5.41E-14	Up
ARMCX7P	5.313093206	-1.638045856	1.87E-12	2.29E-11	Up
PCLAF	5.309304073	4.158834504	2.14E-15	3.59E-14	Up
MTHFD2P[illegible]	5.308991027	-2.783173946	3.53E-06	1.76E-05	Up
ARPC3P5	5.30836297	-1.641989276	5.06E-11	5.23E-10	Up
AC024451.1	5.30658628	-2.7781257	1.11E-07	7.11E-07	Up

AP001625.2	-2.016656997	-2.498197789	0.000329955	0.00109267	Down
AL136968.2	5.306136645	-1.635728745	3.19E-11	3.38E-10	Up
CEP55	5.3036916	3.173820886	2.06E-20	5.78E-19	Up
IL1R2	5.299137096	1.710387569	2.10E-05	8.97E-05	Up
RPS3AP29	5.2974797	-2.773806768	2.24E-07	1.37E-06	Up
FCF1P6	5.295130476	-2.800018189	0.000367671	0.001204145	Up
AC078850.1	5.294452651	-1.655956036	4.08E-05	0.000164426	Up
RPS26P35	5.293796889	-2.797670636	1.17E-05	5.28E-05	Up
RSL24D1P1	5.289303083	-2.790699768	1.19E-06	6.44E-06	Up
AL583856.1	5.288075407	-1.044916104	3.09E-13	4.15E-12	Up
LINC0199[illegible]	5.281705605	-0.295583017	1.67E-05	7.27E-05	Up
FABP5P10	5.280006325	-2.812974393	8.52E-05	0.000322092	Up
BUB1	5.279143999	4.499250646	2.16E-19	5.46E-18	Up
EEF1A1P1[illegible]	5.278346156	0.606269117	7.42E-19	1.79E-17	Up
SRPX2	5.275926337	5.197481196	2.06E-07	1.27E-06	Up
TAGLN2P1	5.274616138	1.088883502	4.30E-17	8.71E-16	Up
INPP5A	-2.017135464	4.270903024	2.26E-25	1.03E-23	Down
NINJ2	-2.017779766	2.710337098	2.79E-07	1.68E-06	Down
AC013356.1	5.271557626	-0.630895648	1.59E-13	2.21E-12	Up
CA9	5.266944757	4.274293489	4.07E-05	0.000164069	Up
RPS19P1	5.266443897	0.737932182	5.21E-19	1.28E-17	Up
AL122020.1	5.264681468	1.305124173	3.34E-17	6.82E-16	Up
AP000350.[illegible]	-2.017942187	-0.687338265	0.000263856	0.000896023	Down
BCAS1	-2.01932358	6.526093047	0.000862845	0.002588983	Down
POTEE	5.259466008	-0.035512181	2.74E-21	8.35E-20	Up
RPS27AP3	5.259226058	-2.799818975	4.20E-07	2.46E-06	Up
TGM5	5.258174876	1.441781673	4.85E-05	0.000192758	Up
ABCC3	5.252201856	6.247986608	1.16E-06	6.29E-06	Up
AC008696.1	5.238366359	-2.835867106	7.10E-07	4.00E-06	Up
IMMTP1	5.236451677	-2.826047427	2.08E-07	1.28E-06	Up
WDR38	5.234935997	1.932047269	0.000947012	0.002814566	Up
AC125238.2	5.234089489	-2.831301769	3.86E-08	2.64E-07	Up
GTSE1	5.229709653	3.965994544	1.59E-15	2.71E-14	Up
RPL12P1	5.227059789	-2.836163803	1.40E-07	8.84E-07	Up
RGS8	-2.020600614	2.272290209	6.33E-06	3.00E-05	Down
RPS4XP8	5.221691661	-1.723654153	1.35E-10	1.31E-09	Up
AC092324.2	5.221516444	-2.838654542	3.03E-06	1.53E-05	Up
SBF1	-2.020840407	6.93728199	2.67E-17	5.51E-16	Down
AL009178.2	5.219703408	0.785485537	0.000269038	0.000911577	Up
AC099335.1	5.21748538	-1.088191065	6.32E-16	1.14E-14	Up

NIPAL1	-2.02084446	-1.261328184	3.76E-09	2.99E-08	Down
AC124856.1	5.203800548	-2.849604386	2.66E-06	1.35E-05	Up
KNL1	5.196930625	3.565888264	9.82E-16	1.72E-14	Up
CALM2P1	5.185312363	-2.85765385	1.76E-06	9.28E-06	Up
RPS26P38	5.178694713	-2.880861398	3.64E-05	0.000148109	Up
HMGB1P4.	5.174870226	-2.883574074	0.000838278	0.00252579	Up
AL109809.1	-2.021450958	-0.970812897	4.44E-13	5.86E-12	Down
SRGAP3	-2.021717538	5.506459169	1.25E-15	2.16E-14	Down
SGO1	5.171964456	2.57700786	3.15E-14	4.70E-13	Up
MFAP2	5.171342007	3.754870184	0.000128913	0.000467702	Up
UBD	5.171070188	0.72815991	5.14E-05	0.000203377	Up
AC099560.2	5.168208524	1.80103192	6.04E-20	1.62E-18	Up
PHBP8	5.160415008	-2.883445865	3.97E-07	2.33E-06	Up
CENPU	5.160414316	4.505786246	4.14E-16	7.64E-15	Up
GALNT5	5.157353523	2.174355497	5.58E-05	0.000218996	Up
CCNB2	5.156678457	4.609343669	1.67E-15	2.85E-14	Up
AC104619.3	5.154614568	1.399855592	3.99E-25	1.78E-23	Up
AL355355.2	-2.022835238	-1.825546601	2.82E-09	2.27E-08	Down
GLDN	-2.023194885	5.043295754	9.08E-07	5.02E-06	Down
RPL3P3	5.150970805	-1.157596248	6.45E-17	1.28E-15	Up
RPSAP15	5.147317481	1.093500283	1.13E-19	2.96E-18	Up
APTM4BP	5.143453764	-2.903843984	2.27E-06	1.17E-05	Up
ELENOTP	5.142229275	0.093978069	5.13E-16	9.33E-15	Up
AL591846.1	5.141761841	3.295330001	3.79E-05	0.000153999	Up
ATP5MC2P	5.141091277	-0.739146361	2.52E-17	5.23E-16	Up
AC010422.3	5.1351566	1.944479878	1.76E-17	3.71E-16	Up
TPT1P9	5.1350717	2.383289661	1.56E-18	3.65E-17	Up
APOBEC3I	5.132586115	2.036738147	3.14E-11	3.33E-10	Up
TNNI3K	-2.0238155	-1.116068876	2.03E-09	1.68E-08	Down
INHCAFP	5.126363756	-2.917988236	1.87E-05	8.08E-05	Up
RPL5P4	5.125525326	1.27947125	4.16E-22	1.39E-20	Up
SKA1	5.124050658	2.97943486	1.65E-15	2.82E-14	Up
AC139085.1	5.121127694	-2.911762575	8.04E-06	3.74E-05	Up
AL714022.1	5.119800088	-2.912168425	9.25E-06	4.25E-05	Up
PKD1L2	-2.024403795	0.50070605	2.67E-05	0.00011216	Down
AL138899.2	5.119445169	-1.781149742	0.00046366	0.001482129	Up
SAP18P2	5.118800473	-2.903962312	2.58E-06	1.32E-05	Up
AC239600.2	5.114598305	-1.817025118	7.47E-11	7.53E-10	Up
HOXD3	5.111594271	-0.728919209	2.74E-06	1.39E-05	Up
TFAP2A-AS	5.104126156	0.978939194	8.39E-07	4.66E-06	Up

RPL34P26	5.101358811	-2.924807526	9.12E-06	4.19E-05	Up
AC010409.2	5.101008704	-1.82151588	7.03E-11	7.11E-10	Up
AC113382.1	-2.026291356	-3.087715914	3.46E-09	2.75E-08	Down
MYL6P5	5.089159037	-0.185698998	1.56E-15	2.68E-14	Up
AC026798.1	5.086920577	-2.93118721	7.06E-06	3.32E-05	Up
SPOCD1	5.079366396	6.60474589	4.00E-06	1.97E-05	Up
ESCO2	5.0746134	3.061556353	5.72E-14	8.28E-13	Up
RPS4XP3	5.073599059	-0.212492008	1.83E-16	3.49E-15	Up
TERT	5.073118113	0.336621418	0.0001418	0.000510219	Up
LINC0203:	-2.026392522	-2.606698818	7.13E-08	4.70E-07	Down
MYOCD	-2.027918123	-1.83993342	8.72E-05	0.000328853	Down
HLA-DQA1	5.071561266	3.776570189	8.66E-05	0.000327078	Up
RPS3AP5	5.067279766	2.882468666	4.07E-09	3.22E-08	Up
AC010524.1	5.05825469	-2.966393347	0.000151505	0.000541689	Up
RPS3AP25	5.056045356	0.00366284	2.06E-14	3.12E-13	Up
RPS20P10	5.053463566	-1.247315464	4.70E-11	4.89E-10	Up
ABCA13	5.051972218	1.824022272	0.000113693	0.000417604	Up
TTK	5.051420076	3.566130396	1.38E-13	1.91E-12	Up
AC008802.1	5.046595591	-1.880751742	1.37E-07	8.65E-07	Up
AL513175.2	5.035942552	-2.975151427	2.03E-06	1.05E-05	Up
OSR2	5.034232767	1.139764863	1.60E-06	8.49E-06	Up
AC008694.2	5.030292753	-2.974309394	2.67E-05	0.000111985	Up
HNRNPLP	5.022635825	-1.260118344	1.38E-17	2.94E-16	Up
LINC0047:	5.017743048	1.458766949	0.000179432	0.000631123	Up
MIR548AA:	5.017383431	-2.994737148	0.000300183	0.001004236	Up
AC135068.(	5.017223443	-2.971250921	8.08E-06	3.75E-05	Up
AC005019.1	5.01543649	-2.978044026	5.09E-06	2.45E-05	Up
MESTP4	5.015144916	-2.989767801	5.52E-05	0.00021691	Up
RPL24P8	5.01387067	1.991203685	6.56E-21	1.93E-19	Up
IGIP	-2.028340394	4.523190543	5.67E-22	1.87E-20	Down
RPL5P17	5.013767407	-0.539655105	3.78E-14	5.59E-13	Up
RPS3AP6	5.013452291	3.990124618	2.15E-17	4.48E-16	Up
AC020658.1	5.011813978	-2.983336701	1.97E-06	1.03E-05	Up
KPNA2P1	5.010279484	-2.982435691	3.85E-06	1.90E-05	Up
MANCR	5.003965529	-1.936314912	1.75E-06	9.20E-06	Up
HOXC4	4.999482863	2.819087698	4.74E-15	7.66E-14	Up
AC104619.1	4.99239859	-1.910312325	6.03E-11	6.17E-10	Up
RAB11FIP2	-2.028964802	4.005137754	4.30E-39	7.47E-37	Down
AC026403.1	4.992346325	2.773090283	1.43E-20	4.07E-19	Up
LINC0220:	4.988198544	-0.610501141	2.36E-05	9.99E-05	Up

AC006539.1	4.986191975	-2.997998883	4.67E-06	2.26E-05	Up
AP002982.1	4.985215443	-1.915977364	3.68E-12	4.38E-11	Up
AC069213.1	-2.029464003	-2.491770902	7.25E-09	5.51E-08	Down
RPS18P13	4.981243435	-1.935831754	2.32E-09	1.90E-08	Up
AL161781.1	4.979664623	-2.998376744	8.57E-06	3.96E-05	Up
GSX1	4.978772517	2.560484995	0.000147069	0.000527134	Up
AC093663.1	4.978123298	-3.006531426	1.92E-05	8.30E-05	Up
AL033519.3	4.975229494	0.697955471	5.03E-18	1.11E-16	Up
AL672212.1	4.974847577	-3.009176498	1.34E-05	5.98E-05	Up
RPL34P1	4.971559497	-3.009118758	1.74E-05	7.56E-05	Up
AL512310.7	4.969534245	-3.01257561	4.80E-06	2.32E-05	Up
AC093270.2	4.96489706	-3.006504013	2.67E-06	1.36E-05	Up
RPL7AP53	4.960534465	-1.322536932	6.48E-12	7.47E-11	Up
COL1A1	4.959636028	9.114049747	0.000435758	0.00140124	Up
H3P6	4.954299333	4.514007313	7.26E-32	6.03E-30	Up
RPS3AP26	4.950604243	3.613004617	6.00E-18	1.32E-16	Up
RPL7AP31	4.948416883	-0.93086161	8.34E-14	1.19E-12	Up
KLHL14	-2.029903831	-0.226859624	0.00051518	0.001628746	Down
LINC01206	4.947277588	1.416754698	0.000462808	0.001480061	Up
IFITM3P2	4.946645966	-0.08421591	1.24E-10	1.21E-09	Up
ZNF25	-2.03006102	4.48835212	1.81E-28	1.08E-26	Down
AC108114.1	4.946602954	-3.025738999	4.05E-06	1.99E-05	Up
AC104563.1	4.944954357	0.108421434	4.15E-20	1.13E-18	Up
CYP17A1	-2.030465497	-1.160842803	1.34E-08	9.84E-08	Down
SETP8	4.941440393	-1.972119019	1.39E-11	1.54E-10	Up
TPT1P6	4.94080116	0.062676666	1.29E-13	1.80E-12	Up
AC008147.3	4.939593542	-3.048551597	0.000607872	0.001891492	Up
AC016894.1	4.939469725	-3.019893355	8.02E-07	4.47E-06	Up
EEF1GP4	4.938346745	-3.027469942	5.84E-07	3.34E-06	Up
EEF1A1P2	4.930220294	-1.352732534	1.84E-12	2.26E-11	Up
CSTA	4.929850406	1.9685714	1.07E-08	7.95E-08	Up
PDE6H	-2.03150038	-3.227049832	5.69E-05	0.000222873	Down
SLC25A6P2	4.926713184	-1.968444155	1.92E-11	2.10E-10	Up
YBX1P1	4.924925091	2.890546121	7.33E-30	4.99E-28	Up
KIF4A	4.916636236	4.619612252	2.71E-17	5.60E-16	Up
RPL31P35	4.916425342	-3.045167495	3.52E-06	1.76E-05	Up
ANXA2P2	4.915994053	3.366160928	2.63E-12	3.18E-11	Up
BUB1B	4.912410963	3.904435584	2.33E-15	3.91E-14	Up
RAET1K	4.911142921	-1.98652989	6.62E-07	3.75E-06	Up
RPS27P10	4.903961858	-3.055967409	9.64E-06	4.40E-05	Up

RPS26P47	4.896553171	-0.632020908	2.32E-06	1.19E-05	Up
TMSB4XP2	4.894629198	1.145896599	6.14E-17	1.22E-15	Up
RPSAP17	4.88945147	0.016543703	1.08E-16	2.11E-15	Up
RPS6KA5	-2.032021678	3.129518411	2.56E-15	4.26E-14	Down
TGFBI	4.888648042	8.01529982	7.62E-08	5.00E-07	Up
SPINK8	4.877933469	2.03009908	2.02E-09	1.67E-08	Up
RPL5P34	4.877682172	2.600467459	1.19E-25	5.49E-24	Up
FAM131A	-2.032612899	5.78995792	6.07E-39	1.03E-36	Down
POTEI	4.877564073	-0.975420875	8.72E-19	2.09E-17	Up
AC015689.	4.872322945	-3.086958126	1.04E-05	4.73E-05	Up
DTL	4.870633913	4.147818927	7.34E-14	1.05E-12	Up
RPSAP6	4.869600493	-2.031226413	4.87E-09	3.80E-08	Up
CLIC4P3	4.868333709	-2.019617698	2.41E-08	1.71E-07	Up
H3P1	4.868315855	-2.007578443	5.06E-11	5.24E-10	Up
AC018523.	4.867989206	-2.016502981	8.24E-11	8.25E-10	Up
ACTBP2	4.858424257	2.570136633	1.46E-33	1.45E-31	Up
AL158163.	-2.034888188	-2.581148449	1.95E-11	2.12E-10	Down
MCM10	4.857071953	2.960770176	6.16E-12	7.13E-11	Up
AC025176.	4.853978555	-2.032098905	2.82E-06	1.43E-05	Up
RPL10AP3	4.851650782	-3.085783071	2.09E-05	8.95E-05	Up
FTLP3	4.849370975	4.897433258	9.62E-16	1.69E-14	Up
AC113398.	4.847662578	0.168975825	1.69E-18	3.94E-17	Up
BX640514.	4.840304166	-1.014459396	7.92E-05	0.000301244	Up
KIF2C	4.836434093	4.724379421	9.59E-16	1.68E-14	Up
CDK1	4.834973273	4.961209267	5.11E-16	9.30E-15	Up
ZDBF2	-2.03489266	3.714954516	1.39E-10	1.34E-09	Down
FOXM1	4.831533272	5.70078953	4.39E-15	7.13E-14	Up
SDHCP2	4.830386778	-3.103258559	6.27E-06	2.97E-05	Up
RPL34P18	4.819298856	0.77165178	1.94E-17	4.07E-16	Up
AC023906.	4.818155837	-1.469968296	1.93E-11	2.11E-10	Up
AL390755.1	4.818058545	5.585001757	3.39E-09	2.71E-08	Up
AC010343.	4.817156932	3.479377368	4.83E-21	1.44E-19	Up
RPS2P55	4.814387877	1.417137673	6.76E-26	3.21E-24	Up
CRNDE	4.813940166	3.47508076	3.53E-12	4.21E-11	Up
AL603882.1	4.811688874	-1.457434464	4.65E-13	6.12E-12	Up
SLAMF9	4.808980405	0.528413653	0.000147843	0.000529646	Up
FP236241.1	4.804220247	-3.112633881	3.87E-05	0.000156563	Up
HMGN2P	4.801712671	3.330713135	7.09E-27	3.68E-25	Up
LINC0157	4.795373544	4.295391192	4.72E-10	4.23E-09	Up
COL4A1	4.791458097	8.882192161	9.07E-12	1.03E-10	Up

\C097658.:	4.790833295	0.524649603	2.46E-17	5.11E-16	Up
ACTG1P21	4.786382949	-2.092838361	1.88E-11	2.05E-10	Up
\RHGAP2:	-2.035710283	6.303225072	1.67E-17	3.52E-16	Down
GPX8	4.780416999	4.349623949	5.32E-09	4.13E-08	Up
E2F7	4.780349746	3.192180913	6.50E-13	8.40E-12	Up
RPS4XP13	4.778551953	-0.486970343	1.54E-14	2.37E-13	Up
\L121871.1	4.777049973	-0.760140506	1.51E-13	2.09E-12	Up
RPL19P11	4.776433851	-3.141951617	2.70E-05	0.000113167	Up
CAPZA1P1	4.77544485	-3.140333759	2.34E-05	9.90E-05	Up
HMGN1P3	4.769422905	0.093012273	5.94E-28	3.37E-26	Up
RPL37AP1	4.767480046	1.183082096	2.60E-23	9.73E-22	Up
PRRG1	-2.036150994	4.015676694	7.01E-14	1.01E-12	Down
\C009362.:	4.766336433	-1.083167601	3.21E-12	3.85E-11	Up
\L121768.1	-2.036376275	-2.640179973	2.53E-09	2.06E-08	Down
GYG1P3	4.764498336	-3.151310594	1.47E-05	6.50E-05	Up
\C098935.:	4.763970801	-0.284904778	2.56E-15	4.26E-14	Up
RPS10P3	4.762063087	1.488404767	7.48E-19	1.80E-17	Up
CD200	-2.03673523	4.843507064	1.31E-14	2.03E-13	Down
\L021328.1	4.76112516	-1.0855962	0.000153102	0.000546382	Up
\L034405.1	-2.037030359	-2.116012319	0.000846404	0.002547805	Down
\L357054.2	-2.037053013	-3.134892527	4.20E-06	2.06E-05	Down
TPT1P4	4.758807209	1.617958446	1.27E-17	2.72E-16	Up
YBX3P1	4.758724614	-1.507741144	3.46E-10	3.17E-09	Up
\C021074.:	4.758557349	0.819136421	4.61E-20	1.25E-18	Up
CDC25C	4.756421512	2.574639954	3.21E-13	4.31E-12	Up
RPS2P48	4.750401292	-0.763273288	2.17E-17	4.53E-16	Up
\C007686.:	4.745982593	-2.129520955	1.03E-08	7.69E-08	Up
\C093142.:	4.740032649	-3.156430243	1.92E-05	8.28E-05	Up
RPS16P2	4.738095058	-3.157098945	2.68E-05	0.000112437	Up
F2R	4.737231525	6.684686898	1.04E-16	2.03E-15	Up
LINC0029	-2.037623372	3.991627333	1.42E-37	2.03E-35	Down
ASPM	4.734480652	3.915327644	9.07E-12	1.03E-10	Up
RPL23AP8:	4.73260348	-3.151481964	2.06E-05	8.84E-05	Up
\C093789.:	4.729719041	-1.531155059	1.42E-11	1.57E-10	Up
EIF4A1P2	4.725841545	0.580366993	1.31E-24	5.56E-23	Up
\C007683.:	4.724969161	0.580500752	2.15E-24	8.97E-23	Up
AACS	-2.03839186	4.722532475	1.86E-35	2.19E-33	Down
RPL8P2	4.723911371	-2.13746854	2.61E-09	2.12E-08	Up
SDCBPP3	4.719187855	-1.133084775	2.14E-15	3.59E-14	Up
RPL37P2	4.716361023	0.86064609	7.73E-21	2.26E-19	Up

LEFTY2	4.715718852	3.076011056	7.55E-06	3.52E-05	Up
AL583805.1	4.715544583	-0.799239332	4.30E-12	5.08E-11	Up
AC209007.[illegible]	4.713747276	-1.129184748	4.07E-13	5.40E-12	Up
[illegible]RTCAP2P	4.713557385	-1.119410727	1.88E-15	3.19E-14	Up
RPL39P15	4.711221126	-0.807285721	2.54E-11	2.73E-10	Up
RPL23AP7[illegible]	4.710433636	-2.14066294	8.37E-11	8.37E-10	Up
AC090142.[illegible]	4.710270846	-3.162612623	6.20E-06	2.94E-05	Up
SEC61G	4.708342642	9.222741093	0.000594147	0.001853991	Up
RPL38P3	4.707151277	-3.173876373	3.62E-05	0.000147562	Up
RPSAP45	4.706265221	-1.148608935	2.27E-12	2.77E-11	Up
RPS27P14	4.703182227	-3.192859194	0.000324071	0.001074791	Up
RPL5P9	4.702078797	-0.55206403	9.55E-13	1.21E-11	Up
RPS15AP3[illegible]	4.697784961	-0.141909087	2.03E-16	3.86E-15	Up
POTEJ	4.696732229	-0.817084917	1.31E-16	2.53E-15	Up
AP003097.1	4.696506709	-2.163797247	1.80E-09	1.49E-08	Up
RPL5P1	4.693897297	1.051871089	2.94E-18	6.66E-17	Up
EEF1A1P1[illegible]	4.691472616	1.265082469	3.38E-23	1.25E-21	Up
SEC11B	4.677805707	-2.172425759	2.08E-09	1.71E-08	Up
MAGED4	4.676758945	1.127373306	9.87E-16	1.73E-14	Up
RPS26P31	4.674944589	-1.17848734	1.11E-08	8.21E-08	Up
RPA2P2	4.669741611	-3.196804028	9.04E-06	4.15E-05	Up
RPL12P4	4.663617587	2.159529679	3.96E-25	1.77E-23	Up
CYP19A1	4.66235515	2.341318063	1.47E-06	7.87E-06	Up
CXCL10	4.659755917	4.514832746	5.35E-05	0.000210863	Up
CYRIA	-2.03936729	5.950579278	1.49E-25	6.88E-24	Down
TREM1	4.651320535	4.058616884	4.17E-05	0.00016753	Up
RPL10P8	4.650303217	-1.613540559	6.67E-11	6.79E-10	Up
INF2	-2.039678189	6.198043398	1.37E-20	3.91E-19	Down
AC008677.[illegible]	4.649189957	-1.610675614	1.29E-11	1.44E-10	Up
EEF1A1P3[illegible]	4.648239705	1.063106539	3.31E-19	8.26E-18	Up
RAB42	4.644525408	3.227004713	7.65E-16	1.36E-14	Up
RPS12P23	4.643275801	-1.610858223	1.70E-10	1.63E-09	Up
H2BS1	4.639441998	-3.234530138	0.000331129	0.001095713	Up
AC016571.[illegible]	-2.040287276	-1.961427342	2.73E-06	1.39E-05	Down
OPTN	-2.04075422	5.881409495	7.61E-23	2.73E-21	Down
RPS5P3	4.638843527	-2.214882706	2.53E-07	1.53E-06	Up
CHI3L2	4.635173785	8.419868234	0.000128665	0.000466945	Up
CDK4	4.630333949	8.999866803	0.000728764	0.002225137	Up
RPL7P36	4.625561397	-3.231692694	7.31E-05	0.000280141	Up
EML2	-2.041231859	4.960329529	3.63E-16	6.76E-15	Down

CENPK	4.624128825	3.44651471	9.26E-15	1.46E-13	Up
AC016735.1	-2.042182964	-2.879747074	1.11E-05	5.04E-05	Down
JNDC3A-A	-2.042873919	0.099045362	2.32E-09	1.90E-08	Down
EEF1A1P1	4.617090214	-0.213646951	1.38E-16	2.67E-15	Up
RPL29P2	4.615088495	-2.223721703	4.55E-09	3.57E-08	Up
FABP5P1	4.605681457	-0.898671252	4.09E-06	2.01E-05	Up
SRP72P1	4.604677	-3.251935577	0.000299042	0.001000766	Up
AC006386.2	4.60313619	-3.253511558	0.000131304	0.00047548	Up
AC016739.1	4.600979088	3.696870168	1.29E-25	5.94E-24	Up
LYPLA2P1	4.600465741	-1.652520638	1.80E-14	2.75E-13	Up
RPSAP12	4.600295376	1.502046971	6.03E-19	1.47E-17	Up
AC131235.1	4.598094291	2.40794128	2.35E-18	5.35E-17	Up
AC104212.3	4.597500294	-0.923264618	5.53E-13	7.19E-12	Up
RPS7P8	4.596040299	-2.233018552	3.76E-08	2.59E-07	Up
RPL12P13	4.595859705	-2.235144941	6.70E-09	5.12E-08	Up
EEF1A1P2	4.595321144	0.561452229	6.02E-19	1.47E-17	Up
RPS7P3	4.59434705	-0.930078267	9.56E-12	1.08E-10	Up
TDRD6	-2.043221256	0.434666457	4.21E-08	2.87E-07	Down
CCT6P2	4.586223802	-0.092583447	1.41E-06	7.57E-06	Up
OR2I1P	4.581049407	0.163964344	0.000250784	0.000855666	Up
FABP5P7	4.573854138	1.516674681	4.07E-06	2.00E-05	Up
AC018475.1	4.57373683	0.072359878	2.10E-18	4.80E-17	Up
TMSB4XP8	4.572539915	5.235703386	2.24E-18	5.11E-17	Up
RPS2P7	4.570978965	1.745666437	6.40E-23	2.30E-21	Up
AC104843.1	4.570503738	-2.264429689	3.30E-08	2.29E-07	Up
RPS3AP49	4.569505412	0.198865953	9.29E-13	1.18E-11	Up
AC106806.1	4.566121425	-2.261114163	1.49E-07	9.37E-07	Up
FOXJ1	4.562226893	5.487837669	3.43E-05	0.00014052	Up
GBX2	4.557599443	1.92920863	0.000296503	0.000993192	Up
AL513190.1	-2.044266069	-2.230369658	3.71E-08	2.55E-07	Down
PPIAP1	4.555261315	-3.277376009	7.99E-05	0.000303914	Up
AC097634.3	-2.044593972	-2.989415834	2.07E-05	8.87E-05	Down
AL365226.1	4.555221461	-3.268136302	0.000133356	0.000482001	Up
RPS23P8	4.554634607	2.851271234	1.67E-26	8.37E-25	Up
RPS19P7	4.552605556	-1.688525391	3.21E-10	2.96E-09	Up
CBX3P9	4.552002561	0.044494028	2.50E-18	5.69E-17	Up
RPL27P6	4.548901981	-1.697081163	2.95E-10	2.74E-09	Up
RPL5P26	4.545916377	-3.277235397	4.63E-05	0.00018446	Up
EEF1A1P1	4.541786992	-2.277405261	1.10E-08	8.19E-08	Up
KLHL11	-2.045751302	1.416509342	4.23E-13	5.61E-12	Down

RPL39P29	4.538477534	-3.284722563	0.000367944	0.001204901	Up
APOL4	4.536956548	5.652930544	1.34E-06	7.20E-06	Up
AC034102.1	4.530534145	1.08595072	8.28E-18	1.80E-16	Up
RPL9P18	4.526957181	-0.518596285	3.25E-11	3.44E-10	Up
RPS2P4	4.526165293	-0.121572643	2.04E-20	5.73E-19	Up
AL356653.1	4.525145402	-0.713306761	2.22E-16	4.20E-15	Up
SEPTIN7P	4.524950119	-2.300612826	3.86E-08	2.65E-07	Up
KIF18A	4.520455442	2.739848476	2.96E-15	4.87E-14	Up
AC104248.1	4.518953347	-2.325044439	0.00081106	0.002450445	Up
FAM135B	-2.04593882	3.455043954	3.69E-08	2.54E-07	Down
EZH2	4.516957579	5.236677552	1.88E-17	3.96E-16	Up
AL355032.1	4.515473086	-0.005655896	5.73E-16	1.03E-14	Up
GJA3	4.499813387	0.72520772	0.00014424	0.000518027	Up
NIPSNAP3	-2.046673814	1.075930982	8.12E-11	8.14E-10	Down
AC005476.1	4.49254474	-1.729315193	4.92E-12	5.77E-11	Up
OR2B6	4.481236188	-1.752791079	6.91E-05	0.000266635	Up
PFN1P1	4.478598006	1.111970423	1.61E-23	6.17E-22	Up
FTLP15	4.476148609	-2.348505282	3.69E-06	1.83E-05	Up
RPL27AP5	4.474772333	-1.336103889	2.27E-11	2.45E-10	Up
AC069236.1	4.46956175	-2.351007428	4.71E-09	3.69E-08	Up
AC010719.1	4.467866261	-1.355390457	4.14E-05	0.000166667	Up
RPL39P39	4.466251205	-3.33125638	0.000479864	0.001527318	Up
AC132219.2	-2.046938607	-0.998677962	1.63E-13	2.24E-12	Down
RPS2P28	4.462674824	-2.352945469	4.62E-09	3.62E-08	Up
AC012414.4	-2.047007438	-3.114201814	0.000191075	0.000668079	Down
EEF1A1P3	4.459993799	0.216650695	2.19E-17	4.56E-16	Up
AC090058.1	4.45897639	-2.347524804	3.03E-08	2.11E-07	Up
HNRNPH1P	4.448745128	-2.369740264	1.83E-09	1.52E-08	Up
CXCL9	4.447745214	2.539272027	9.52E-05	0.000356437	Up
ANXA3	-2.047376705	0.554924194	4.13E-06	2.03E-05	Down
FGFBP2	4.446684781	2.540021037	0.000399657	0.00129696	Up
MGAT4C	-2.047574422	2.0508382	0.000599637	0.001869503	Down
AC024082.2	4.446208451	-3.34758924	0.00059338	0.001851999	Up
NR5A2	4.445621779	1.445700254	5.05E-13	6.61E-12	Up
DMBX1	4.445250452	-0.184521545	0.000558963	0.001755036	Up
AKR1B1P2	4.444835044	-2.357169444	8.99E-09	6.74E-08	Up
COL8A1	4.444535354	5.296427698	0.000250232	0.000854084	Up
EEF1A1P4	4.442502909	2.056237616	1.02E-20	2.94E-19	Up
RPL23AP58	4.436737752	-2.351585561	1.22E-09	1.04E-08	Up
IRX3	4.430151063	2.054761382	3.63E-05	0.000148037	Up

ESPL1	4.429959876	3.707429807	1.28E-13	1.79E-12	Up
RPS3AP42	4.428110931	-3.350510024	0.000479371	0.001526085	Up
RPL17P34	4.42626207	-1.059964642	7.41E-12	8.47E-11	Up
ATP5MGP2	4.420282982	-1.804400628	6.27E-10	5.53E-09	Up
RPL7P27	4.419430302	-3.350943612	0.000196308	0.000684879	Up
PGK2	4.418337309	-3.362173703	0.000463283	0.001481278	Up
ATP1B3P1	4.414332548	-1.805807785	2.53E-12	3.06E-11	Up
IQCA1-AS1	4.410554158	-1.827310633	3.33E-05	0.000136546	Up
AC061975.1	-2.048159752	-3.224004533	2.96E-06	1.49E-05	Down
TIMP4	4.408814843	6.713422489	1.58E-07	9.90E-07	Up
PTGDR2	-2.048326015	0.709655737	8.68E-07	4.81E-06	Down
AC092112.1	4.408268445	-0.866476247	1.50E-05	6.61E-05	Up
RPL12P33	4.405771358	-1.81942161	6.41E-10	5.65E-09	Up
DTNB	-2.048373665	4.159813646	1.01E-33	1.03E-31	Down
DIRAS1	-2.048643345	6.032856209	4.04E-12	4.79E-11	Down
RPL22P2	4.405334792	0.024371778	4.28E-18	9.57E-17	Up
ATP5F1AP1	4.404507194	-1.815703346	3.29E-10	3.03E-09	Up
RPL5P5	4.404071132	-1.099129693	1.36E-10	1.32E-09	Up
CD163	4.403722941	7.623881582	2.46E-05	0.000103901	Up
MACIR	-2.049939524	4.23503529	3.82E-21	1.15E-19	Down
SLC25A5P2	4.39859136	-1.821071672	4.44E-11	4.64E-10	Up
RPL19P16	4.397619366	-0.827314485	1.27E-17	2.71E-16	Up
FMOD	4.394580485	6.965754376	0.000472569	0.001507095	Up
PPM1L	-2.050086273	4.865370842	1.96E-16	3.73E-15	Down
FTLP2	4.391302543	2.27311808	9.72E-13	1.23E-11	Up
FCGR2B	4.386237104	3.612800877	2.33E-05	9.87E-05	Up
AC112777.1	4.386035651	0.991344407	3.36E-08	2.33E-07	Up
RPL36AP21	4.385961415	-2.415174	7.89E-08	5.17E-07	Up
COL1A2	4.382543687	9.384052199	0.000682074	0.002096349	Up
HSPD1P6	4.382437303	-0.677104624	1.14E-09	9.78E-09	Up
RPL15P14	4.380011355	-1.10495139	1.22E-12	1.53E-11	Up
FAUP1	4.379490719	1.716306877	3.02E-22	1.03E-20	Up
RBBP4P4	4.378349485	-2.417058632	1.70E-08	1.22E-07	Up
AC090114.1	4.374840411	0.990934268	3.65E-15	5.98E-14	Up
H2AC13	4.374816649	-1.862938503	0.000760075	0.002309723	Up
AL583785.1	4.373937522	-0.857074915	0.000625133	0.001940175	Up
AC146944.3	4.373919101	-2.412984223	0.000456546	0.001462144	Up
RPS15AP11	4.370369642	-0.002209201	3.22E-15	5.29E-14	Up
CLIC4P1	4.369704193	-1.449199474	3.67E-09	2.92E-08	Up
AC135507.1	-2.050189017	-0.95323429	8.09E-18	1.77E-16	Down

NEIL3	4.368568	2.223568737	4.44E-12	5.24E-11	Up
RPSAP3	4.367099536	-0.460244858	3.92E-14	5.79E-13	Up
TPT1P7	4.366272571	-3.387987672	0.000593917	0.001853474	Up
AC079250.1	4.363958322	1.619553726	1.26E-15	2.19E-14	Up
HPD	4.363565682	1.951594896	2.65E-05	0.000111305	Up
AP001010.1	-2.051061258	-3.180069645	1.44E-05	6.39E-05	Down
EEF1A1P1	4.359047921	3.68907808	3.51E-19	8.73E-18	Up
CLEC12A	4.35892911	0.932139853	2.11E-05	9.02E-05	Up
MRPL3P1	4.358822526	-1.132362538	1.04E-15	1.81E-14	Up
RPS2P17	4.352474927	0.670100072	6.84E-21	2.00E-19	Up
AC073861.1	4.35186929	4.549562475	7.44E-16	1.33E-14	Up
Z97353.1	4.349758893	-0.02309933	2.41E-17	5.01E-16	Up
PDPN	4.345297382	8.213197502	6.69E-09	5.11E-08	Up
AL022718.1	4.336925196	-1.885882228	1.13E-07	7.23E-07	Up
ADGRE3	4.336515382	-1.142122099	0.000776661	0.00235491	Up
RPS7P10	4.336440832	1.690011899	1.40E-16	2.71E-15	Up
RPL10P1	4.327566237	-0.480218062	8.99E-15	1.42E-13	Up
AC125807.1	4.322568372	0.29565636	1.15E-12	1.44E-11	Up
IFITM3P4	4.319873721	-1.189048809	4.66E-09	3.65E-08	Up
CNIH3-AS1	4.319039511	-0.506038529	0.000806142	0.002436352	Up
LINC00570	-2.051863118	-3.316307399	0.000280042	0.000944754	Down
RPL9P32	4.316445323	-0.195285799	4.14E-12	4.90E-11	Up
RPS7P15	4.315204266	-2.474167555	4.69E-07	2.72E-06	Up
IGFBP3	4.312807527	8.446942431	1.81E-05	7.86E-05	Up
AC091042.1	4.304777157	-2.473445522	7.66E-07	4.29E-06	Up
AC025594.1	4.301942695	-2.472343871	0.000199492	0.00069456	Up
PTPN4	-2.05238532	4.231847474	7.17E-35	8.10E-33	Down
RPS27P21	4.301348894	-1.19593605	2.46E-11	2.65E-10	Up
AL353662.2	4.295404295	-0.927171503	1.20E-12	1.51E-11	Up
AC104849.1	4.291170757	-1.914627592	3.11E-10	2.87E-09	Up
NEK10	-2.053869517	0.759842154	1.17E-09	9.97E-09	Down
BTF3P10	4.290569945	-0.937136468	1.45E-17	3.09E-16	Up
AL162430.1	4.288438178	-0.361580432	2.11E-17	4.41E-16	Up
AL359233.1	-2.054227795	-2.545980737	0.000292481	0.000980861	Down
EEF1A1P24	4.288189853	1.053042061	1.00E-18	2.39E-17	Up
RPS11P5	4.287749275	2.469665258	1.17E-19	3.05E-18	Up
PARP1P1	4.287319055	-1.519063924	4.36E-12	5.15E-11	Up
HCRTR1	-2.054259748	-2.196816609	2.00E-05	8.61E-05	Down
CKS1BP3	4.28454139	-2.492155057	1.24E-06	6.69E-06	Up
RPL7P10	4.280663834	-2.487705229	5.03E-08	3.39E-07	Up

AC106800.2	4.280469749	-2.502668637	5.21E-07	3.00E-06	Up
PGDP1	4.280454333	-1.520958307	7.73E-14	1.11E-12	Up
AL355974.2	4.276531592	1.846496241	4.95E-07	2.87E-06	Up
LINC0226(	4.275270239	-1.245518511	1.54E-06	8.21E-06	Up
ATP6V1B1	4.274297538	1.82817966	1.66E-08	1.20E-07	Up
NOX4	4.273293406	3.003036377	1.67E-12	2.05E-11	Up
AC023512.1	4.272795459	-0.249018303	2.74E-15	4.54E-14	Up
RPSAP47	4.27203907	0.157942741	3.58E-15	5.86E-14	Up
AC125611.4	4.269413221	-0.557461057	2.35E-09	1.92E-08	Up
RPSAP21	4.268611103	-2.501650228	5.12E-07	2.95E-06	Up
C2CD4B	-2.054456603	-0.748311664	2.58E-06	1.32E-05	Down
GAPDHP6	4.268454297	-1.23223506	1.49E-10	1.44E-09	Up
AL136226.1	4.267313822	-2.499670484	1.50E-07	9.45E-07	Up
TMSB4XP4	4.263320006	3.304192446	1.06E-14	1.65E-13	Up
RPS26P15	4.262997562	-1.24383836	1.12E-08	8.32E-08	Up
RPL26P37	4.26071135	-2.515365246	2.17E-06	1.12E-05	Up
RBP7	-2.054641938	1.215417722	8.71E-07	4.82E-06	Down
ACTBP11	4.260169212	0.745920621	7.01E-25	3.07E-23	Up
AL109741.2	4.259689732	-1.23695862	1.60E-10	1.54E-09	Up
RPS29P3	4.259655628	-0.735552569	2.68E-11	2.87E-10	Up
RPS27AP5	4.258531484	2.139497245	9.22E-21	2.68E-19	Up
AP001086.1	4.252849938	-2.502689427	4.30E-07	2.51E-06	Up
AP000529.1	4.252422426	-2.508032344	3.35E-08	2.32E-07	Up
AL357055.1	4.252308337	-1.939162865	2.64E-09	2.14E-08	Up
RPS27P29	4.250891529	-1.544219923	3.24E-10	2.98E-09	Up
RPL7AP6	4.249650115	3.605575114	2.56E-21	7.85E-20	Up
RPS10P14	4.248398236	-2.511414918	1.44E-06	7.72E-06	Up
SIX1	4.248260931	3.199047099	1.32E-05	5.90E-05	Up
IQGAP2	4.247943036	4.777400831	7.92E-14	1.13E-12	Up
LINC00672	-2.054825042	3.050145663	5.99E-06	2.85E-05	Down
RPL35AP2	4.24355451	-2.514423373	5.80E-07	3.32E-06	Up
TOM1L2	-2.055252028	6.383133585	4.28E-25	1.90E-23	Down
ORC1	4.241232378	2.858884277	8.82E-14	1.26E-12	Up
EEF1A1P2(	4.239512277	-2.521657392	1.26E-06	6.80E-06	Up
PAICSP4	4.239330768	-0.588773565	5.18E-11	5.35E-10	Up
RPL35P5	4.239290841	1.743450863	1.76E-15	2.99E-14	Up
EEF1A1P2	4.236747285	1.227190879	8.08E-21	2.35E-19	Up
PPIAP13	4.234330304	-1.23609414	5.49E-11	5.65E-10	Up
AC091932.1	4.232225647	-0.727300524	0.000715929	0.002189891	Up
EIF4A1P1(	4.232143094	2.31091999	1.73E-28	1.03E-26	Up

C21orf62	4.231150555	5.425072395	3.67E-07	2.17E-06	Up
Z74021.1	4.226871662	1.570429028	7.63E-17	1.51E-15	Up
AL450996.1	4.224154517	-2.521340812	9.41E-08	6.09E-07	Up
AC073349.	-2.055567334	-2.283616424	8.35E-06	3.87E-05	Down
RPL3P12	4.223305369	-1.977304615	1.12E-09	9.63E-09	Up
NSA2P1	4.222668416	-2.553068273	0.00017631	0.000621355	Up
CDH15	4.219371491	1.084597787	0.000770899	0.002340887	Up
MS4A6A	4.218325666	6.577438767	6.54E-08	4.34E-07	Up
MT2P1	4.214314973	1.952211392	7.41E-08	4.88E-07	Up
LOX	4.214206699	5.763321358	1.38E-05	6.13E-05	Up
SDHCP4	4.213665842	-2.539916331	3.83E-07	2.26E-06	Up
AL049871.1	4.212906418	0.175561835	3.32E-05	0.000136165	Up
C9orf129	-2.055631134	-0.657008599	3.29E-07	1.96E-06	Down
AC083904.	4.212298943	-2.538931727	1.14E-08	8.46E-08	Up
RPL24P7	4.210466711	-1.575645395	3.92E-10	3.57E-09	Up
RPL13P2	4.210287219	-1.587308385	2.03E-11	2.20E-10	Up
RPL31P49	4.205974289	0.061600531	1.86E-14	2.83E-13	Up
EFHD1	-2.055647872	6.11279836	1.14E-07	7.27E-07	Down
LYZ	4.204415746	5.490730696	1.14E-05	5.16E-05	Up
PRDX2P4	4.201675132	-1.986878757	3.20E-10	2.95E-09	Up
NUF2	4.20135858	3.883317696	3.71E-12	4.41E-11	Up
RPL10AP2	4.200437518	0.289394176	2.91E-16	5.48E-15	Up
AIDAP1	4.19804574	-2.558906833	2.63E-08	1.85E-07	Up
TFPI	4.190792116	5.329147423	4.33E-09	3.41E-08	Up
FCGBP	4.190570093	8.899288931	9.05E-07	5.01E-06	Up
AC008753.	4.189684617	-1.007837482	2.06E-13	2.81E-12	Up
EIF4A1P6	4.185731044	-2.555789095	4.01E-08	2.74E-07	Up
MDFI	4.181566475	5.713105116	2.66E-07	1.61E-06	Up
AC055716.	4.177360894	-2.017936912	3.66E-10	3.34E-09	Up
FAM83F	-2.056441245	-2.202467544	9.43E-09	7.05E-08	Down
BX322784.	4.176159349	-2.026726367	2.32E-09	1.90E-08	Up
CTBP2P4	4.176027325	-2.585485471	9.63E-08	6.22E-07	Up
AC007688.	4.17484339	0.281589979	5.75E-14	8.33E-13	Up
AC090281.	4.17455834	-2.007200877	6.98E-11	7.08E-10	Up
EEF1A1P2	4.174272306	-0.318296969	4.67E-16	8.54E-15	Up
OR51E1	4.172913885	1.134544138	2.48E-05	0.000104714	Up
AC024940.	4.172669381	1.354980838	7.87E-18	1.72E-16	Up
ANP32BP1	4.16464316	-0.845087742	2.38E-16	4.50E-15	Up
UHRF1	4.163356662	5.3715509	2.67E-09	2.16E-08	Up
AC093422.	4.163048419	-2.570116315	1.25E-06	6.73E-06	Up

RPSAP54	4.161135636	1.152098372	3.47E-17	7.07E-16	Up
RPS3P6	4.158187469	-1.06287568	4.52E-12	5.32E-11	Up
RPS29P16	4.157765112	-0.34370728	3.37E-13	4.51E-12	Up
RPS29P5	4.157089269	0.129256607	3.13E-14	4.68E-13	Up
AC009806.	-2.056979354	-2.74100718	5.35E-09	4.15E-08	Down
PTX3	4.156515724	5.456684285	0.000177927	0.000626288	Up
RPS15AP1	4.155827019	-2.038072552	9.73E-06	4.44E-05	Up
EEF1A1P2	4.150438104	0.776717582	9.19E-19	2.20E-17	Up
AL138785.1	4.149727613	-0.339917255	4.64E-13	6.12E-12	Up
AL096829.1	4.149114473	-2.580538317	4.76E-07	2.76E-06	Up
AL731533.	-2.058558243	-0.908532635	8.14E-11	8.16E-10	Down
EIF4A1P9	4.148166189	-2.58979424	1.91E-08	1.37E-07	Up
AL049873.1	4.146904881	1.551673448	3.63E-16	6.76E-15	Up
AC069218.	4.144621481	0.108211772	1.81E-16	3.46E-15	Up
TUBBP2	4.143507039	-0.355468142	1.77E-16	3.38E-15	Up
CDCA7	4.141948974	4.606466133	7.56E-16	1.35E-14	Up
TUBA1C	4.139573548	6.877872131	4.37E-14	6.42E-13	Up
HTRA4	4.13551242	-1.094966435	0.000856167	0.002571357	Up
EEF1A1P7	4.133807361	1.220953488	1.09E-20	3.13E-19	Up
AC090602.	4.132877059	-1.649373419	3.02E-09	2.43E-08	Up
NPM1P48	4.131615977	-3.511030312	0.000971668	0.00288012	Up
HASPIN	4.130631435	1.411182843	3.93E-11	4.12E-10	Up
ACTG1P9	4.125841038	0.006228437	3.11E-18	7.04E-17	Up
LINC01451	4.12542746	-1.354830514	7.35E-07	4.13E-06	Up
CDCA8	4.124417427	4.289150804	7.57E-13	9.71E-12	Up
PHLPP2	-2.059433575	4.021277904	9.08E-24	3.59E-22	Down
KIF23	4.124246745	4.007363694	1.15E-13	1.62E-12	Up
CKAP2L	4.12229754	3.73937862	2.08E-13	2.84E-12	Up
HMMR	4.119438754	3.419764806	2.30E-11	2.48E-10	Up
RPSAP58	4.118799412	-0.87341231	2.57E-12	3.11E-11	Up
SKA3	4.116199048	3.433324991	1.87E-11	2.04E-10	Up
CPXM1	4.11079164	6.376781381	1.78E-08	1.28E-07	Up
AL358176.	4.106002965	-2.055595083	1.29E-08	9.44E-08	Up
AC090921.	-2.059741442	-2.998657238	3.94E-08	2.70E-07	Down
RPL18AP1	4.104305114	-1.364582224	1.60E-10	1.54E-09	Up
FRRS1	4.103436812	1.060174056	8.43E-10	7.31E-09	Up
RPL29P33	4.102097508	-1.362795008	1.05E-12	1.33E-11	Up
AL162151.	4.099294515	1.685631296	2.29E-20	6.38E-19	Up
SOX1-OT	-2.059982226	0.904827987	0.000501112	0.001589152	Down
AP000280.1	4.098613194	-1.647535621	0.000197469	0.00068818	Up

NCAPH	4.098569937	3.99047501	6.20E-12	7.16E-11	Up
SPC24	4.098330765	4.163474421	5.90E-11	6.04E-10	Up
AL157902.2	-2.06114474	-2.43362165	4.88E-10	4.37E-09	Down
TRMT112P	4.098149712	-0.247783322	1.58E-16	3.03E-15	Up
KEL	-2.061397	-0.316968894	1.09E-08	8.11E-08	Down
AL022322.1	-2.062687134	0.262034768	5.11E-07	2.95E-06	Down
MESTP3	4.097534357	-2.108583661	4.52E-05	0.000180454	Up
ANXA1	4.096633475	8.402577627	1.57E-08	1.14E-07	Up
EEF1A1P1	4.096279479	1.284870873	2.71E-19	6.79E-18	Up
AL049552.1	-2.063021136	-1.273183298	1.61E-07	1.01E-06	Down
AL356000.1	4.088393901	-1.6938522	5.69E-12	6.62E-11	Up
ATP6V0D2	4.087473657	-0.68926391	0.000307911	0.001027346	Up
RPL27AP	4.081793419	-1.69239096	1.08E-08	8.01E-08	Up
LHPP	-2.063340527	5.312997215	4.10E-10	3.72E-09	Down
TTBK1	-2.06348661	4.598468906	7.12E-13	9.16E-12	Down
RPSAP49	4.081745332	-1.137505511	8.71E-11	8.68E-10	Up
AC026700.1	4.081373869	-2.102893944	1.16E-09	9.91E-09	Up
RPL7P59	4.080803833	-2.642738776	3.65E-06	1.81E-05	Up
PPIAP48	4.079529215	-1.359055625	0.00042932	0.001381885	Up
WEE1	4.076014591	5.78544442	6.49E-20	1.74E-18	Up
ADAMTS1	4.074862994	2.888527186	3.31E-05	0.00013598	Up
RPL39P3	4.074689702	3.643774897	2.77E-14	4.14E-13	Up
CD207	4.074525973	-0.711853575	0.000347683	0.001144798	Up
MDK	4.072699174	7.405464494	3.88E-13	5.16E-12	Up
CENPM	4.071880394	3.352127571	5.22E-11	5.39E-10	Up
RAC1P4	4.070547025	-2.092797068	4.46E-10	4.01E-09	Up
HAS2	4.068411687	4.305965149	7.51E-10	6.57E-09	Up
NDUFB9P	4.068322352	-1.379439335	1.28E-11	1.43E-10	Up
RPS3AP21	4.064879247	1.340661649	1.21E-12	1.51E-11	Up
KIF18B	4.062573242	4.27700218	2.62E-09	2.13E-08	Up
SYNGAP1	-2.06374464	5.738362566	6.83E-21	2.00E-19	Down
RPL17P26	4.061006176	-2.097482117	1.50E-07	9.43E-07	Up
EEF1GP5	4.06058124	0.159998089	1.41E-19	3.66E-18	Up
HMGN2P4	4.058422491	0.059799708	6.28E-17	1.25E-15	Up
MS4A4E	4.058415897	-0.283121339	5.59E-06	2.67E-05	Up
AL158211.5	-2.066542188	-0.656723562	3.33E-10	3.06E-09	Down
SERBP1P6	4.05660729	-1.711716597	6.99E-11	7.08E-10	Up
GPR65	4.05511404	3.602222474	2.63E-09	2.14E-08	Up
GAPDHP6	4.053805377	1.424638808	3.00E-18	6.79E-17	Up
ARPC3P2	4.053738171	-2.656035539	6.44E-07	3.65E-06	Up

AC090578.1	-2.066741343	-1.242969353	1.65E-09	1.38E-08	Down
CASC8	4.052664623	-2.147067196	0.000389659	0.001267799	Up
GPR63	-2.06680628	1.160010073	4.75E-07	2.76E-06	Down
NDUFB4P1	4.051946133	-1.416261735	8.53E-12	9.68E-11	Up
RPSAP46	4.051855132	-0.91773191	9.17E-11	9.13E-10	Up
FTH1P10	4.0489149	2.049130258	4.44E-16	8.15E-15	Up
RPL18P13	4.048904555	-0.917808066	4.45E-12	5.24E-11	Up
AGTPBP1	-2.067436947	4.523674525	1.90E-22	6.61E-21	Down
HPRT1	-2.068169149	4.602909471	7.96E-16	1.41E-14	Down
AGAP1	-2.068922927	6.407142129	5.65E-17	1.13E-15	Down
AL603766.1	4.047222474	-1.729155294	5.93E-12	6.88E-11	Up
CLEC4E	4.046626152	1.82631584	2.85E-05	0.000118951	Up
TARS3	-2.069363035	3.984199239	5.40E-24	2.18E-22	Down
RHAG	4.043740356	-2.67154682	3.89E-05	0.00015751	Up
AC091429.1	4.040637023	-0.076586598	6.62E-15	1.06E-13	Up
RPL5P18	4.038807264	-1.180388244	6.27E-12	7.25E-11	Up
RPL5P32	4.038406967	-2.674733128	8.55E-06	3.95E-05	Up
AL354710.1	4.035751936	-1.727962142	1.65E-09	1.38E-08	Up
ATP5PBP6	4.035406274	-2.666721273	4.71E-06	2.28E-05	Up
PLP2	4.034265407	6.416620823	1.97E-09	1.63E-08	Up
ANXA2	4.034226353	8.800175849	6.45E-10	5.68E-09	Up
IFITM3P1	4.033711689	-1.428169837	3.60E-08	2.48E-07	Up
PLD1	-2.070140774	3.395119084	3.95E-10	3.59E-09	Down
TMSB4XP1	4.029843868	1.231748188	2.86E-14	4.28E-13	Up
HSPE1P4	4.027426688	-1.745122391	1.89E-10	1.80E-09	Up
FAM234B	-2.07069645	4.91052058	5.05E-22	1.67E-20	Down
RPS4XP11	4.024420455	0.130519447	1.37E-14	2.12E-13	Up
CACNA1G	-2.071276432	4.079180139	4.79E-06	2.31E-05	Down
RPL13AP5	4.022786832	5.452728401	1.22E-18	2.88E-17	Up
AL031727.1	4.02076719	1.555693898	9.86E-14	1.40E-12	Up
PHBP11	4.020486697	-2.133903781	6.93E-11	7.04E-10	Up
NHSL2	-2.071803838	0.11310493	2.32E-10	2.18E-09	Down
AC022149.1	4.018811829	0.537336013	1.26E-12	1.58E-11	Up
ILF2P1	4.01810356	-2.681110563	4.35E-07	2.54E-06	Up
AC004386.1	4.017842607	-1.767149445	6.63E-08	4.39E-07	Up
GDF15	4.015847396	4.023201661	2.58E-05	0.000108587	Up
BDH2P1	4.015370277	-0.775710413	5.66E-11	5.81E-10	Up
RPS13P2	4.013722656	1.786811336	1.57E-18	3.67E-17	Up
AL139421.1	4.01211602	-1.750245255	3.30E-10	3.03E-09	Up
ATP6V1C1	-2.071903892	6.230333414	3.68E-46	1.28E-43	Down

RELL2	-2.072118144	2.28001862	1.54E-15	2.64E-14	Down
AL122013.1	4.011970503	-2.696083432	1.00E-06	5.50E-06	Up
PKMP4	4.010486484	-1.204459925	3.12E-11	3.31E-10	Up
AL445433.1	4.010099949	-1.18943855	8.31E-10	7.21E-09	Up
LINC0258[illegible]	4.010027159	1.267269343	2.23E-05	9.49E-05	Up
AC010457.[illegible]	4.009899681	-1.765952526	9.59E-05	0.000358681	Up
AP000936.[illegible]	4.009168667	-0.482524587	1.02E-12	1.28E-11	Up
RPL15P18	4.005021663	-0.218180273	4.60E-16	8.43E-15	Up
C13orf42	-2.0722208	-3.155128749	0.000124849	0.000454276	Down
RPL23P8	4.004197837	0.261560929	9.12E-18	1.98E-16	Up
RPS4XP7	4.000136579	-1.005943203	8.80E-11	8.77E-10	Up
RIIAD1	-2.07269982	0.498386443	4.83E-07	2.80E-06	Down
LINC0204[illegible]	-2.072852326	-1.454907468	4.30E-06	2.10E-05	Down
CCNA1	-2.072895181	1.517174671	3.12E-05	0.00012897	Down
AL359092.[illegible]	3.996022145	-2.149860918	1.05E-08	7.82E-08	Up
OIP5	3.99588676	2.072331815	8.20E-12	9.33E-11	Up
IGF2BP2	3.995591733	4.243343526	8.37E-05	0.000317029	Up
CCNA2	3.993392499	4.508735131	2.37E-14	3.57E-13	Up
AC099789.[illegible]	3.99160576	-2.705060154	1.80E-07	1.11E-06	Up
[illegible]TP5MC2P	3.989382195	-2.161525898	2.05E-08	1.46E-07	Up
FTLP17	3.98893174	-1.471579322	3.21E-08	2.23E-07	Up
EEF1A1P1[illegible]	3.988759671	3.270665009	9.81E-20	2.59E-18	Up
B4GALNT[illegible]	-2.07344968	2.037523555	1.90E-06	9.92E-06	Down
RPSAP1	3.988031451	-2.718335156	7.28E-06	3.41E-05	Up
EEF1B2P1	3.9862742	-1.479634659	8.05E-10	7.00E-09	Up
AL645608.[illegible]	3.983288736	-2.19752032	7.96E-05	0.000302872	Up
RNVU1-1	-2.073740526	-2.813194976	8.20E-07	4.56E-06	Down
RPS2P35	3.980893116	-1.461820589	7.96E-12	9.06E-11	Up
EEF1GP2	3.976811996	-2.698930211	4.72E-07	2.74E-06	Up
AC108734.[illegible]	3.975936012	-2.710730244	1.29E-07	8.19E-07	Up
RPL5P6	3.975372601	-1.793555027	9.91E-09	7.39E-08	Up
CLSTN1	-2.074044026	8.750235757	2.84E-23	1.06E-21	Down
TEX29	-2.074372871	-0.606298685	3.10E-09	2.48E-08	Down
RPL29P19	3.974163808	-1.832334147	1.22E-05	5.49E-05	Up
SLC25A3P[illegible]	3.973840374	-2.161039619	2.62E-10	2.45E-09	Up
EF1AKM[illegible]	3.971079106	5.376775128	0.000568064	0.001781475	Up
SLC39A12	-2.075095185	3.207604144	2.51E-05	0.000105949	Down
AC107075.[illegible]	3.96720183	0.05367322	2.20E-14	3.33E-13	Up
RPL12P6	3.965427164	-1.476203663	4.43E-11	4.63E-10	Up
DMRTA2	3.963832949	4.041962259	0.000393429	0.001278911	Up

AL121723.1	3.963315034	-0.655360843	1.94E-17	4.08E-16	Up
DYNLL1P2	3.956705935	-2.19510613	4.08E-09	3.22E-08	Up
SERBP1P1	3.956636301	-0.058902575	7.81E-15	1.24E-13	Up
AL731892.1	3.950370188	-2.723973966	2.04E-06	1.06E-05	Up
PPIAP87	3.949592641	-2.188947462	6.37E-08	4.24E-07	Up
SCN1B	-2.075205315	4.360645761	1.67E-12	2.06E-11	Down
AC005587.1	3.949132005	-2.735748043	1.32E-05	5.89E-05	Up
PPIAP23	3.948460644	-2.730322528	2.10E-06	1.09E-05	Up
MAP2K4	-2.075457727	5.344314652	1.68E-43	4.33E-41	Down
AC004552.1	3.948140024	-0.82471201	7.37E-09	5.60E-08	Up
AL161742.1	3.942641928	-2.734148591	8.24E-07	4.58E-06	Up
AC005480.2	3.942233079	-1.035429163	9.96E-11	9.87E-10	Up
RPL34P31	3.941749976	-1.512130355	6.01E-08	4.01E-07	Up
RPS12P4	3.939144257	-1.512698775	7.93E-10	6.91E-09	Up
AC004453.1	3.936661556	3.241196896	3.66E-15	5.99E-14	Up
CNGA3	3.934465185	5.033566898	4.25E-05	0.000170539	Up
STEAP3	3.931717721	6.573182575	9.26E-09	6.93E-08	Up
SFRP4	3.928528052	5.471391258	1.00E-05	4.55E-05	Up
IGFL4	-2.07569905	0.852572654	7.71E-07	4.31E-06	Down
HGAP29-A	3.92650816	-2.786507561	0.000745064	0.002268414	Up
AC009245.1	3.925593645	0.676371851	7.62E-20	2.02E-18	Up
IK3CD-AS	3.921972388	0.9207415	3.52E-06	1.76E-05	Up
MS4A4A	3.920782812	5.055425366	4.35E-06	2.12E-05	Up
KIAA1109	-2.076650575	5.725090726	1.72E-23	6.53E-22	Down
AL080243.2	3.917943438	2.177264316	2.68E-18	6.09E-17	Up
RPS3AP16	3.908654795	-2.216756982	2.62E-06	1.33E-05	Up
SATB2-AS1	-2.07709474	0.884114849	2.99E-11	3.18E-10	Down
AC098859.1	3.907617591	-1.847704946	4.87E-12	5.72E-11	Up
CLEC5A	3.899995469	4.106750179	2.37E-06	1.22E-05	Up
PAQR6	-2.078120156	6.515168925	1.44E-09	1.22E-08	Down
TMEM14D	3.899664346	-0.894159163	4.35E-13	5.76E-12	Up
MEX3A	3.898303138	5.407831773	9.16E-08	5.94E-07	Up
RPS4XP19	3.894663513	-2.771829299	3.65E-06	1.81E-05	Up
EIF3LP1	3.893499845	-2.239917284	1.87E-08	1.34E-07	Up
LINC0142	3.893380526	1.419430331	1.43E-06	7.64E-06	Up
NME2P1	3.893337888	0.981986381	4.51E-18	1.01E-16	Up
RPSAP8	3.892999809	-0.605900457	1.72E-12	2.11E-11	Up
HSPE1P5	3.892384315	-2.76696863	2.23E-06	1.15E-05	Up
RPL12P19	3.892125408	-1.546583116	4.41E-11	4.61E-10	Up
AL162426.1	-2.07818027	-2.327084666	2.72E-06	1.38E-05	Down

TACC3	3.890463848	6.3026928	0.000143767	0.000516442	Up
'RKCQ-AS	-2.07880598	1.995355985	8.87E-10	7.67E-09	Down
ACTBP8	3.889655097	-0.304467796	1.36E-17	2.89E-16	Up
RPS3AP44	3.889261865	-0.491036086	1.07E-09	9.18E-09	Up
ιL034370.1	3.88849584	-0.730990333	7.53E-18	1.65E-16	Up
ιC008799.1	3.886063499	-1.54878738	2.91E-08	2.03E-07	Up
IMGN2P2	3.883804433	-2.799527292	1.84E-05	7.97E-05	Up
MXRA5	3.88159516	5.18521276	0.000163611	0.000580221	Up
NACA2	3.880655232	0.2356463	3.37E-19	8.38E-18	Up
NKRD18C	-2.079331895	-1.567795753	5.68E-06	2.71E-05	Down
HSPE1P6	3.877609775	-2.253220585	5.16E-08	3.47E-07	Up
ιC011625.1	3.876166121	-2.26856581	0.000860657	0.002583227	Up
FPR3	3.875692738	3.833979922	2.62E-06	1.33E-05	Up
LDH7A1P	3.875591852	0.395826173	1.51E-11	1.67E-10	Up
ιL035411.1	3.875224364	0.525568866	8.73E-16	1.54E-14	Up
ιF279873.1	3.875156654	-1.112648031	1.17E-12	1.47E-11	Up
RPSAP29	3.874018366	-0.931777773	2.06E-11	2.24E-10	Up
UBE2SP2	3.873213767	-0.367254964	6.83E-10	6.01E-09	Up
SIGLEC7	3.872978376	2.236761419	1.54E-07	9.65E-07	Up
RPL13P12	3.872821497	5.129542079	9.30E-12	1.05E-10	Up
F2RL2	3.871779581	2.248518915	0.000329243	0.001090563	Up
ZDHHC11I	-2.080462599	2.853058328	5.16E-06	2.48E-05	Down
ιC112656.1	3.870067318	-2.263927316	2.33E-07	1.42E-06	Up
RPS20P14	3.868758	1.234003027	1.25E-18	2.95E-17	Up
SF3A3P1	3.868422857	-2.270192245	1.80E-09	1.50E-08	Up
RPSAP63	3.867335485	-2.246989031	4.93E-08	3.33E-07	Up
ιL080243.1	3.866449557	-0.039443402	1.48E-21	4.70E-20	Up
LINC0063	-2.080624124	3.13194049	3.70E-09	2.94E-08	Down
RPS2P6	3.865760645	-2.269315152	9.14E-09	6.84E-08	Up
ιC112191.2	3.865197699	-1.33248747	2.60E-11	2.78E-10	Up
RPS3AP53	3.86452365	-2.798008427	3.18E-05	0.000131155	Up
MSR1	3.864371561	6.389642177	2.18E-08	1.55E-07	Up
ιL662795.2	-2.080984118	3.268575415	2.30E-17	4.79E-16	Down
RPSAP4	3.862183571	-0.045509819	4.65E-13	6.12E-12	Up
TAAR3P	3.861666299	-0.014512546	5.15E-06	2.47E-05	Up
ERCC6L	3.860989253	1.612496056	4.73E-11	4.92E-10	Up
CKS2	3.85965113	5.000844779	1.29E-11	1.44E-10	Up
ιL355309.1	3.858934611	-2.804720529	2.47E-05	0.000104292	Up
NRNPA1I	3.858864966	-0.373412615	5.77E-15	9.25E-14	Up
MMP2	3.858850565	7.306487774	5.89E-08	3.93E-07	Up

RPL10P16	3.857676696	3.381099149	2.27E-21	7.01E-20	Up
HMGN2P3	3.857618672	1.019085245	4.27E-19	1.06E-17	Up
DEPDC1	3.857528705	3.359194645	3.10E-10	2.87E-09	Up
TPTE2P1	-2.081529645	-0.194382194	1.91E-06	9.98E-06	Down
ACTG1P11	3.857294153	-2.797732742	1.33E-06	7.17E-06	Up
S100A4	3.851100897	5.157248745	1.82E-06	9.58E-06	Up
RPL7P13	3.850822344	-2.810514307	1.16E-05	5.24E-05	Up
AC011773.3	3.849211055	-2.821345854	1.53E-05	6.72E-05	Up
LOXL2	3.848867912	6.068256406	4.06E-06	1.99E-05	Up
VNN1	3.84767072	0.638811511	7.35E-07	4.13E-06	Up
HTR7	-2.081564172	0.10099455	2.21E-08	1.57E-07	Down
AC044787.1	3.846100924	1.256686209	5.26E-17	1.06E-15	Up
RPL18AP3	3.845925532	4.718836037	8.44E-14	1.21E-12	Up
RPL31P28	3.845495098	-2.811145774	1.30E-05	5.80E-05	Up
RPSAP76	3.842848085	-1.906041245	4.08E-09	3.23E-08	Up
EIF4A1P7	3.840632201	-1.121565839	3.24E-14	4.83E-13	Up
AL355994.2	-2.081578615	-1.544425812	0.000674274	0.002074864	Down
NTD3-TM	-2.081709347	-2.125319935	1.26E-10	1.23E-09	Down
AC060764.1	3.839899148	-2.819951127	0.000207472	0.000719734	Up
PP1R14BP	3.839102033	-1.603258904	2.65E-11	2.84E-10	Up
SOX11	3.838675725	6.077963576	3.17E-06	1.59E-05	Up
AMIGO1	-2.0821538	4.772417009	2.34E-19	5.90E-18	Down
POLD2P1	3.837566323	-2.298804927	1.61E-08	1.17E-07	Up
TPX2	3.836470964	5.926351154	1.05E-12	1.32E-11	Up
NRNPA1P	3.834088677	-1.590563429	3.67E-10	3.35E-09	Up
ARL4AP2	3.833421522	-2.2955385	9.48E-08	6.13E-07	Up
TNFRSF19	3.830603815	5.898582784	6.41E-12	7.40E-11	Up
HADHAP2	3.830201776	-0.949293726	5.34E-16	9.68E-15	Up
AC002056.1	3.829197405	-1.603207328	3.60E-13	4.80E-12	Up
AC091685.2	3.827966024	-1.359849538	5.67E-12	6.60E-11	Up
SPC25	3.827632154	3.379488084	1.30E-10	1.26E-09	Up
RAD51	3.824845068	2.956439965	1.36E-12	1.70E-11	Up
AC024293.1	3.823241833	4.637359431	1.03E-15	1.80E-14	Up
LEF1-AS1	3.822705554	0.567190883	1.31E-06	7.04E-06	Up
AC008677.1	3.819730411	-2.83468281	3.16E-07	1.89E-06	Up
TNC	3.818920349	9.333286894	4.53E-09	3.55E-08	Up
MMP14	3.817222134	7.97597792	1.18E-08	8.67E-08	Up
RPL5P3	3.816551041	-0.655150247	2.91E-12	3.51E-11	Up
AC036164.1	3.816489781	-2.82878566	3.73E-06	1.85E-05	Up
RPL29P12	3.816446898	-0.651502387	8.67E-14	1.24E-12	Up

RPS3AP4	3.815627746	-2.829161443	3.97E-05	0.000160144	Up
RPL10P14	3.813742449	-2.828887227	2.08E-05	8.92E-05	Up
RPSAP55	3.812454482	-1.930180574	3.39E-08	2.34E-07	Up
RPS15AP1	3.812151487	1.66579347	2.84E-15	4.69E-14	Up
TFAP2A	3.812042412	3.100152685	0.000484759	0.001541194	Up
RPL7AP30	3.810176329	1.357372332	3.92E-17	7.96E-16	Up
CTCFL	3.809372175	-2.314886794	9.93E-06	4.52E-05	Up
LINC01411	-2.08215928	1.079927043	0.000263728	0.000895693	Down
RPL13AP7	3.800518675	1.218778342	4.64E-16	8.49E-15	Up
NK4-TEX	-2.082733246	-2.199869771	5.57E-07	3.20E-06	Down
SINHCAF	3.799272164	4.751714159	5.64E-11	5.80E-10	Up
PNKDP1	3.798336458	-2.847518124	1.99E-06	1.04E-05	Up
GAPDHP6	3.797070222	-1.177531182	3.77E-11	3.97E-10	Up
RBBP4P5	3.794191421	-2.332704583	2.62E-08	1.84E-07	Up
GLRX3P2	3.794166838	-1.633606594	1.03E-09	8.89E-09	Up
RPL9P21	3.793171014	-1.172882093	2.69E-09	2.18E-08	Up
AC092720.	-2.08303865	-1.970155312	1.83E-09	1.52E-08	Down
PDCD1LG	3.792071542	2.814561871	1.27E-06	6.88E-06	Up
COL4A2	3.790766999	8.854767989	5.77E-09	4.45E-08	Up
EEF1A1P	3.788094252	6.7751557	6.28E-18	1.38E-16	Up
AL021392.1	3.787908077	-0.565674873	0.000302187	0.001010468	Up
RTRAFP1	3.780375121	-2.31229564	2.13E-08	1.52E-07	Up
LNCOG	3.779795323	-0.557328734	1.57E-05	6.88E-05	Up
RPS6P22	3.778046004	-2.879735211	0.00017663	0.000622407	Up
AC005083.	3.776163663	-0.98036631	9.38E-06	4.30E-05	Up
RPL23AP6	3.776071959	0.814632227	7.34E-20	1.95E-18	Up
AC092017.	3.77534374	-1.652820722	2.81E-09	2.27E-08	Up
SLC4A9	-2.084798684	-2.066954133	4.81E-08	3.25E-07	Down
RPS26P28	3.772855415	-1.951008383	2.14E-06	1.11E-05	Up
SD17B3-A	-2.085472325	-2.312193133	7.07E-07	3.99E-06	Down
DCTN1	-2.086161457	7.574489208	1.43E-35	1.69E-33	Down
MYH15	-2.086429392	0.951733886	9.84E-07	5.40E-06	Down
SERPINH1	3.772748652	7.531314947	5.85E-11	6.00E-10	Up
PLAU	3.763761234	5.567844158	1.96E-06	1.02E-05	Up
RPS10P5	3.763418896	-0.85421109	7.97E-12	9.08E-11	Up
NFIA-AS2	3.762473965	3.434858703	7.84E-07	4.37E-06	Up
RPS3AP15	3.762209521	-2.877638758	0.000117772	0.000431084	Up
BTF3P5	3.76124969	-1.659027744	5.92E-11	6.06E-10	Up
DBIP1	3.758808435	-1.704320639	3.03E-07	1.82E-06	Up
BTBD8	-2.087443529	-1.350146695	3.65E-16	6.79E-15	Down

AC087385.2	3.758751113	-0.836725186	2.87E-11	3.07E-10	Up
CA12	3.757242129	6.610154676	2.54E-05	0.000107134	Up
ANGPT2	3.753007475	5.641701822	6.97E-10	6.11E-09	Up
RPL9P3	3.749612079	-0.865367366	1.12E-09	9.59E-09	Up
AL392023.2	-2.088215161	-2.765899905	0.000128104	0.000465119	Down
GRID1-AS1	-2.089196033	-3.459755693	0.000132399	0.000478963	Down
RPL13AP2	3.748890995	-2.380992824	0.000353046	0.001160797	Up
HSPD1P1	3.748236187	0.70185639	2.38E-22	8.22E-21	Up
RNASE2	3.747980066	3.392294098	1.61E-05	7.06E-05	Up
FAM156B	3.747272779	-1.253707752	1.56E-12	1.92E-11	Up
RPL27P7	3.747205311	-2.88201842	3.30E-05	0.0001357	Up
SERPINE1	3.743242283	7.983866668	0.000341579	0.001126264	Up
GAPDHP3	3.741876679	0.49588991	8.92E-16	1.57E-14	Up
CTNNA1P	3.740627925	-1.219000605	2.14E-12	2.61E-11	Up
AC024451.3	3.738600985	-2.889407461	3.54E-06	1.76E-05	Up
AC011511.3	3.737973571	-2.910697958	2.48E-05	0.00010478	Up
AL390039.1	3.737578507	-1.046556844	2.30E-13	3.13E-12	Up
HOMER1	-2.09154484	4.162765908	3.05E-15	5.02E-14	Down
RPE65	3.735099187	3.840244017	1.66E-05	7.23E-05	Up
MYH14	-2.092049455	4.913911654	9.22E-07	5.09E-06	Down
FAM83A	3.733564774	-2.363442053	1.78E-05	7.72E-05	Up
LARP6	-2.092564375	5.340864152	4.95E-23	1.81E-21	Down
AC093866.1	-2.092789224	-3.251773949	8.39E-06	3.88E-05	Down
NCTAM34	3.731868244	0.277465756	1.36E-06	7.32E-06	Up
AC017007.3	3.731532342	-2.894966526	2.05E-05	8.78E-05	Up
AC010240.2	3.729526291	-1.668598562	1.26E-10	1.23E-09	Up
EIF5AL1	3.729508174	2.489240081	1.66E-18	3.87E-17	Up
AL022328.3	-2.093479055	-0.428875233	1.46E-12	1.81E-11	Down
AL445189.2	3.727894847	-0.465627072	2.43E-11	2.62E-10	Up
RPS2P46	3.720057584	3.755719573	9.17E-26	4.32E-24	Up
SUMO2P3	3.720010355	-2.003750997	5.24E-10	4.67E-09	Up
LINC0095	-2.094354851	2.704239631	2.69E-09	2.18E-08	Down
TEPP	-2.095649159	-2.78258795	2.16E-05	9.21E-05	Down
COPS8P2	3.717151961	-0.899072268	2.37E-14	3.57E-13	Up
ABR	-2.095743012	7.719444595	1.86E-23	7.02E-22	Down
H3P14	3.715530505	-0.478909732	2.16E-15	3.62E-14	Up
AC114964.1	3.71391219	-2.385013417	2.82E-07	1.70E-06	Up
SNRPGP2	3.713434377	2.344268086	1.96E-13	2.68E-12	Up
SSBP3	-2.096555144	6.11885683	1.88E-21	5.87E-20	Down
RBM7P1	3.713042309	-2.913380072	8.23E-06	3.82E-05	Up

HMGN2P2	3.710288933	-2.914056743	4.14E-06	2.03E-05	Up
PCOLCE	3.708702414	6.520680763	0.000671774	0.002068445	Up
AC106820.1	3.702989302	-0.476345106	4.39E-10	3.96E-09	Up
AC092115.1	3.700645674	1.178057296	3.32E-22	1.13E-20	Up
EXO1	3.699288586	3.064922406	5.73E-11	5.88E-10	Up
RPS26P6	3.698535688	-1.270093088	2.68E-07	1.62E-06	Up
AC105250.1	3.698512651	-0.379587866	1.00E-12	1.27E-11	Up
RPL41P2	3.698129176	1.853986449	5.28E-16	9.58E-15	Up
RPS26P20	3.698063544	-2.410049169	7.34E-06	3.43E-05	Up
CNNM2	-2.096682928	3.368102139	2.07E-27	1.13E-25	Down
RPL36AP26	3.689956266	-2.035993838	8.25E-08	5.39E-07	Up
RPL10P2	3.688806582	-2.401320743	9.60E-08	6.20E-07	Up
SPEAR-AS	-2.0968155	-2.4868301	0.000381454	0.001244335	Down
TNFSF9	-2.096925965	0.419188861	1.13E-06	6.15E-06	Down
AC092047.1	3.688682643	-2.909306111	5.30E-06	2.54E-05	Up
RPL17P25	3.687522019	-2.9138266	2.08E-05	8.90E-05	Up
RPL22P12	3.684327409	-2.935596209	1.26E-05	5.65E-05	Up
LINC01865	-2.097077173	-2.986820584	0.000131325	0.000475497	Down
CDK18	-2.097338492	6.017606133	7.92E-08	5.19E-07	Down
AC092597.1	3.684163855	0.26535337	1.89E-15	3.20E-14	Up
AC090686.1	3.681936219	-1.08882636	9.78E-09	7.29E-08	Up
ABCC8	-2.097826624	3.135752313	2.82E-05	0.000117841	Down
AP005431.1	3.681318679	-1.740356659	5.20E-09	4.05E-08	Up
RPSAP61	3.681167047	0.431908132	1.87E-13	2.56E-12	Up
EEF1A1P1	3.67963242	4.098767731	1.27E-20	3.64E-19	Up
RPS7P4	3.679363294	-0.437896162	3.46E-10	3.17E-09	Up
STIP1P3	3.679230165	-1.094579695	1.90E-13	2.60E-12	Up
RPS2P5	3.673899383	6.911180195	2.45E-12	2.97E-11	Up
LINC01197	-2.097854406	0.38032636	1.75E-08	1.26E-07	Down
MEST	3.668332533	7.909284935	3.02E-07	1.81E-06	Up
RPL7P37	3.668114313	-2.424795011	1.06E-06	5.80E-06	Up
KANTR	-2.098008332	-0.67761884	5.67E-12	6.60E-11	Down
TYRO3	-2.099260467	6.576559088	6.60E-18	1.45E-16	Down
GXYLT1P	-2.100343654	-3.408977501	0.000146052	0.000524078	Down
EEF1A1P6	3.66765306	4.742358015	1.26E-20	3.59E-19	Up
DLK2	-2.10122108	1.020118221	7.87E-09	5.95E-08	Down
HSPA5P1	3.66712733	0.243490527	8.38E-15	1.32E-13	Up
CALM1	-2.101651497	9.795314878	1.17E-23	4.57E-22	Down
AC007687.1	-2.102039966	-3.121289871	9.13E-06	4.19E-05	Down
AC114491.1	3.665950813	1.817863194	1.63E-12	2.01E-11	Up

XKR5	3.66374386	-0.416715166	8.00E-05	0.000304272	Up
AC018695.1	3.661737712	-2.928517914	5.89E-06	2.80E-05	Up
CRTAC1	-2.102672685	3.419979126	4.98E-05	0.000197539	Down
EIF1P3	3.660907848	-0.320517672	1.08E-15	1.89E-14	Up
ANO5	-2.103790462	2.988821086	1.54E-05	6.76E-05	Down
LSM3P3	3.660401933	-2.948643738	1.52E-05	6.68E-05	Up
BX679664.1	3.660152745	-1.489503458	4.99E-09	3.89E-08	Up
ETV4	3.658501784	4.538827649	2.00E-05	8.60E-05	Up
RPS3AP12	3.658357502	-1.755974107	2.67E-07	1.62E-06	Up
AC108058.1	-2.105045495	-2.754823904	4.20E-05	0.000168835	Down
ANLN	-2.107173134	5.812198076	1.23E-11	1.37E-10	Down
HNRNPCL	3.656417619	-1.740896705	1.14E-11	1.28E-10	Up
TMEM150I	3.65629286	0.202025282	2.72E-05	0.000113893	Up
PRRT3	-2.108844031	3.664902351	1.17E-20	3.36E-19	Down
AC010273.3	3.65393914	-0.823303841	7.09E-07	4.00E-06	Up
AC090337.1	3.652993615	-2.950215562	0.000644207	0.001990582	Up
GNB5	-2.109886137	4.957153697	1.84E-33	1.80E-31	Down
MYL12AP1	3.650154726	-2.440777766	3.67E-07	2.17E-06	Up
ACTBP7	3.649650883	0.797676989	6.39E-24	2.57E-22	Up
AC009078.1	3.649005056	-2.968664537	0.000774731	0.00235079	Up
PPIAP31	3.648345013	1.653863917	1.59E-16	3.04E-15	Up
Z96811.1	3.646220489	-2.071111683	2.60E-08	1.83E-07	Up
DGKI	-2.110042518	3.810913385	3.74E-09	2.97E-08	Down
RPL23AP63	3.644057032	-1.509557707	2.53E-11	2.71E-10	Up
AP001148.1	-2.110834254	0.797745266	3.53E-13	4.72E-12	Down
RPL29P30	3.643273929	-2.433172494	5.57E-07	3.20E-06	Up
SLC34A2	3.642925366	1.651502052	0.000487911	0.00155002	Up
TRAV36DV	3.63921957	-2.075162718	0.000665047	0.00204992	Up
EIF3IP1	3.638579804	-2.961741042	3.82E-05	0.000155123	Up
EEF1A1P9	3.637920864	3.386585436	1.26E-19	3.27E-18	Up
AC069271.1	3.636440795	-2.440748427	3.10E-06	1.56E-05	Up
SERINC2	3.636219915	4.079528825	1.56E-06	8.28E-06	Up
RPL10P4	3.635654759	-2.067870189	5.23E-08	3.51E-07	Up
ST14	3.635454103	4.27296614	2.44E-07	1.48E-06	Up
HNRNPA3I	3.635189396	-2.074882613	5.44E-09	4.22E-08	Up
EMP3	3.633993702	6.820944913	7.38E-09	5.60E-08	Up
LYPD6B	-2.111313246	-0.685764501	0.00063647	0.001969633	Down
LPL	3.633981212	7.349014071	2.37E-06	1.22E-05	Up
AL109618.1	3.633249123	-0.963519839	1.22E-11	1.36E-10	Up
AL161787.1	3.632810073	1.089357023	6.01E-13	7.78E-12	Up

RPL13AP6	3.631909091	0.222366558	9.01E-14	1.28E-12	Up
COX7A2P2	3.630373774	-2.074099872	2.28E-07	1.39E-06	Up
GSTT2	3.629145573	-0.284762357	0.000652672	0.002014578	Up
AL049597.1	3.629033582	-0.967123114	3.13E-10	2.89E-09	Up
CES5AP1	-2.111921993	-3.071904473	2.67E-05	0.000112234	Down
SLC47A2	3.628917419	4.078006057	0.000122177	0.000445452	Up
HSPE1P2	3.62661541	-0.358145627	1.17E-15	2.03E-14	Up
AC022968.	3.624048133	-0.090200154	2.56E-13	3.46E-12	Up
AC009474.	3.623154107	-2.974559177	0.000178561	0.00062829	Up
TMEM45A	3.621139974	4.751222834	1.80E-09	1.50E-08	Up
XIRP2	3.619112912	-1.787117417	0.000515841	0.001630659	Up
ADH5P4	3.617376184	-0.028776925	7.21E-17	1.43E-15	Up
CORO2A	-2.111992511	1.405177413	1.79E-08	1.29E-07	Down
GLRB	-2.11372913	4.54644532	1.06E-16	2.06E-15	Down
OR7E47P	-2.113850487	-2.943577577	1.12E-06	6.09E-06	Down
AL096711.2	-2.113945622	1.195797445	8.04E-12	9.15E-11	Down
CHCHD2P	3.615049087	2.347655382	5.63E-06	2.69E-05	Up
GAPDHP7	3.61346199	1.362849914	1.69E-16	3.25E-15	Up
ELAVL3	-2.114821602	5.874824239	1.28E-07	8.12E-07	Down
CD48	3.613173635	2.971265562	5.27E-06	2.53E-05	Up
AC020983.	3.613006195	-2.465203403	1.87E-06	9.78E-06	Up
MKRN9P	3.612896296	-2.133622145	0.000247416	0.000845978	Up
S100A11P1	3.610438951	-2.986214202	0.000277237	0.0009362	Up
AL592114.1	3.608250216	-0.865408217	7.94E-09	6.00E-08	Up
FRK	-2.11498788	0.302889014	1.18E-11	1.32E-10	Down
RPL10AP6	3.607429927	2.4716505	4.02E-17	8.16E-16	Up
PTTG1	3.60671816	5.20533373	6.82E-12	7.83E-11	Up
LINC02541	3.605755331	-2.092926821	2.43E-05	0.000102604	Up
AL356234.1	3.603006634	-2.998755795	3.50E-05	0.000143243	Up
AC092490.	-2.115250911	-1.905318032	1.08E-05	4.89E-05	Down
BMPR1AP2	3.602336121	-2.490078918	3.04E-07	1.82E-06	Up
TPI1P3	3.601855793	-1.557441085	1.52E-10	1.46E-09	Up
AC079140.2	3.601205967	-0.999598028	2.69E-10	2.51E-09	Up
RPS8P10	3.600996214	-2.963336113	1.64E-05	7.17E-05	Up
NID1	3.60097383	7.180274356	3.51E-07	2.09E-06	Up
SRPX	3.598828876	6.954356696	0.000134565	0.000486127	Up
GBP1P1	3.598494826	2.91699411	0.000213627	0.000739214	Up
EIF4EBP1	3.597122716	5.39530479	1.24E-17	2.66E-16	Up
SETP4	3.595879594	-2.979704812	9.40E-06	4.30E-05	Up
FBXO39	3.595414872	0.361267137	3.25E-05	0.000133616	Up

HMGN2P2	3.594304516	-2.979584081	6.56E-05	0.000254115	Up
CPNE5	-2.115665568	5.630181806	4.01E-07	2.36E-06	Down
CCT5P2	3.594257489	-2.483618416	2.66E-07	1.61E-06	Up
RPL19P5	3.59416171	-0.847905658	1.68E-13	2.32E-12	Up
RPS7P1	3.594091535	3.75827498	2.45E-14	3.69E-13	Up
AL109936.	3.592109796	-2.099766586	2.03E-08	1.45E-07	Up
NPM1P24	3.591428175	-0.997360839	3.67E-13	4.89E-12	Up
DDIAS	3.588932593	3.023784909	5.75E-13	7.46E-12	Up
NMB	3.586500689	6.513481884	7.70E-07	4.31E-06	Up
RPS10P16	3.585032884	-0.880808579	4.33E-08	2.95E-07	Up
VIM	3.583173884	11.50419871	1.90E-16	3.61E-15	Up
AL132640.	-2.115725317	-2.740609661	1.55E-09	1.30E-08	Down
EEF1A1P1	3.582786424	1.048338306	2.73E-15	4.52E-14	Up
CEACAM	3.58222285	-0.415098792	0.000281774	0.000949929	Up
HNRNPKP	3.581843589	0.71889435	1.21E-23	4.69E-22	Up
LINC0051	3.581761248	-1.192361899	5.93E-05	0.000231443	Up
WDR82P1	3.580887198	-2.50914897	3.34E-06	1.67E-05	Up
AC022018.	3.580474849	-0.736877227	4.65E-11	4.84E-10	Up
TUBB2A	-2.116906926	6.98374421	5.43E-21	1.61E-19	Down
RPS4XP14	3.580336948	-0.630514593	1.11E-10	1.09E-09	Up
AL445487.	3.579974717	-2.486611415	5.21E-07	3.00E-06	Up
PA2G4P4	3.578117286	-0.060620404	3.29E-18	7.41E-17	Up
AF279873.	3.5777966	-2.481815747	2.06E-07	1.26E-06	Up
RPL23AP4	3.577287946	-1.179376643	9.62E-13	1.22E-11	Up
ACTBP9	3.576876005	-0.131752673	3.51E-17	7.15E-16	Up
KLHDC8A	3.573939993	7.31317988	1.18E-06	6.42E-06	Up
CFL1P4	3.573634903	-1.031584241	8.29E-14	1.19E-12	Up
PABPC1P1	3.573539539	-2.11830078	7.87E-09	5.96E-08	Up
EEF1A1P1	3.570794939	3.173573921	1.38E-20	3.92E-19	Up
RPL29P25	3.570610371	-3.008187982	3.84E-05	0.000155605	Up
RPL7AP34	3.569719555	-1.822391605	4.23E-09	3.34E-08	Up
GNRHR	-2.117060489	-2.115673888	4.11E-12	4.87E-11	Down
ELOBP2	3.569697264	-1.583464886	2.82E-10	2.63E-09	Up
ENTR1P2	3.568451081	-3.014384697	1.47E-05	6.49E-05	Up
GBP1	3.567585488	6.598877786	1.90E-06	9.93E-06	Up
RPS3AP10	3.567489447	-2.121046984	1.16E-06	6.32E-06	Up
AP000781.	3.565487276	-2.520290914	4.43E-05	0.000176994	Up
TRIM58	-2.118816441	-1.037297497	9.16E-07	5.06E-06	Down
RNASE10	3.563201894	-2.507991425	4.51E-05	0.000180191	Up
TUFMP1	3.562313323	-1.588711231	1.95E-11	2.12E-10	Up

RPL12P17	3.562033781	-1.845089725	3.57E-09	2.84E-08	Up
EEF1B2P3	3.560735125	2.807394175	5.55E-15	8.92E-14	Up
AL031284.1	3.559603941	-1.385216383	2.17E-11	2.34E-10	Up
KIAA1217	-2.119235596	3.824756432	3.42E-10	3.13E-09	Down
U40455.1	3.559276072	-2.131850458	3.55E-08	2.45E-07	Up
VSIG4	3.558958547	7.570135192	2.00E-05	8.61E-05	Up
RPL23AP42	3.558530059	3.208739196	3.40E-20	9.34E-19	Up
RPL17P41	3.556857509	-3.010764474	8.43E-05	0.000318845	Up
GGNBP1	-2.119716414	-2.624128615	1.32E-09	1.12E-08	Down
MEF2D	-2.120177642	5.951144064	4.34E-30	3.02E-28	Down
AC106800.1	3.554899236	0.556660402	9.33E-14	1.33E-12	Up
RPS7P11	3.553728791	1.765029147	2.54E-13	3.44E-12	Up
GLB1L3	-2.12097677	1.594258659	1.60E-05	6.99E-05	Down
RPL15P2	3.551527197	-0.179637199	6.95E-14	1.00E-12	Up
EFNA3	-2.122590834	2.71898112	5.43E-13	7.09E-12	Down
CARD16	3.551445349	3.116449559	8.30E-07	4.62E-06	Up
GPRIN1	-2.123319924	4.774161079	1.56E-10	1.51E-09	Down
AC008026.1	3.55081707	0.835388608	2.61E-12	3.16E-11	Up
RPS29P17	3.550554454	-2.142837748	1.09E-07	6.99E-07	Up
NPM1P9	3.549599443	-1.856387757	1.51E-09	1.27E-08	Up
AL451074.5	3.549345847	-2.512943494	4.24E-06	2.08E-05	Up
SPNS2	-2.1239131	4.60374764	2.57E-13	3.47E-12	Down
APOC1	3.546607645	7.517302363	1.18E-06	6.40E-06	Up
MAPK10	-2.124367847	5.760756335	2.35E-19	5.93E-18	Down
RPL23AP37	3.543447336	-1.843174559	7.61E-11	7.66E-10	Up
MBNL2	-2.124576649	5.638565197	6.59E-29	4.12E-27	Down
AC092865.1	3.541834616	-1.592578144	3.34E-09	2.67E-08	Up
SRP14P1	3.54115682	-2.148085702	1.90E-08	1.37E-07	Up
PLLP	-2.124667301	5.930825796	6.79E-08	4.49E-07	Down
TUBAP2	3.541035463	3.621336311	2.02E-18	4.64E-17	Up
N4BP3	-2.125370585	1.830540652	6.59E-12	7.59E-11	Down
AC009090.3	-2.12537841	-1.304087539	2.69E-09	2.18E-08	Down
SOCAR	3.540833239	-2.537268074	0.000876546	0.002625992	Up
AC006504.3	-2.125713631	-2.483406035	8.24E-09	6.21E-08	Down
RPL7P33	3.539810151	-3.020824943	5.38E-05	0.000211813	Up
HNRNPA1P	3.538337445	-3.009593188	1.47E-05	6.50E-05	Up
TOX4P1	3.538171842	-2.151133244	1.47E-10	1.42E-09	Up
TCP1P1	3.538006654	-1.223153435	6.92E-12	7.93E-11	Up
GJB5	-2.126267807	-0.719737386	2.70E-07	1.63E-06	Down
HNRNPUP	3.537019008	-0.094836189	8.15E-17	1.60E-15	Up

'NFRSF12/	3.535953093	6.337322504	1.02E-07	6.55E-07	Up
AL158201.1	3.535568279	0.719811955	2.70E-11	2.89E-10	Up
AC009269.4	-2.126628319	-2.103981889	1.50E-08	1.09E-07	Down
C15orf48	3.534831856	1.550245752	0.000261207	0.000888283	Up
ACTG1P2	3.534205497	-3.036027696	6.45E-05	0.000250203	Up
RPL7AP66	3.534081127	1.024534333	1.58E-15	2.70E-14	Up
TBX15	3.533503648	3.513908339	1.28E-07	8.13E-07	Up
AC133435.1	3.532532068	-0.344958583	6.17E-21	1.82E-19	Up
WBP1P1	3.532175407	-1.427333897	1.66E-13	2.29E-12	Up
RPS17P5	3.531711461	-2.530938199	1.49E-05	6.59E-05	Up
ASIP	3.531499421	0.328162431	8.86E-08	5.75E-07	Up
CELSR1	3.528223796	3.893701534	9.50E-05	0.000355787	Up
GAS2L3	3.527797937	3.987045499	3.35E-11	3.54E-10	Up
AC097358.2	3.526531278	-1.847362758	1.77E-05	7.70E-05	Up
AL356535.1	3.525920338	-0.126827998	4.77E-09	3.73E-08	Up
ERPINH1I	3.52522654	-2.55259182	9.34E-06	4.28E-05	Up
LINC0210(	3.524135345	-2.154053706	3.54E-05	0.000144637	Up
AC025271.1	3.522450056	-3.053389222	0.000994057	0.00294013	Up
TMEM71	3.522407604	2.690311071	6.89E-07	3.89E-06	Up
AC233279.1	3.522248573	-2.540059322	2.56E-07	1.55E-06	Up
ACTN1-AS	3.521652951	-2.54166231	0.000298415	0.000998901	Up
HS3ST3B1	3.521631885	3.718935491	0.000201305	0.000700195	Up
ALDH8A1	-2.126835391	0.291910514	1.19E-08	8.77E-08	Down
AC092131.1	3.521508301	-0.172113269	0.000654678	0.002020553	Up
INRNPA3I	3.521137874	0.036696599	1.59E-15	2.71E-14	Up
H4C11	3.520826851	-1.634347113	7.31E-05	0.000280264	Up
IFITM9P	3.519254648	-1.614161631	4.71E-07	2.74E-06	Up
MRPS36P1	3.51899098	-2.162304398	7.04E-08	4.65E-07	Up
EEF1A1P3(	3.518177543	-0.655153474	1.08E-13	1.53E-12	Up
CYTL1	3.516947586	4.307340408	7.92E-06	3.68E-05	Up
ZNF215	-2.126873619	-0.878335991	1.55E-06	8.26E-06	Down
TGFB1I1	3.515965392	5.322558123	3.48E-15	5.70E-14	Up
AC099518.2	-2.12701738	-2.334842531	3.19E-09	2.55E-08	Down
GAPT	3.51510999	2.290784669	2.37E-07	1.44E-06	Up
RAMACL	3.514713717	-1.452836256	7.75E-09	5.87E-08	Up
NR4A2	-2.127463101	2.820930606	1.63E-08	1.18E-07	Down
PPP1R12B	-2.128196153	5.170333727	2.22E-22	7.68E-21	Down
SEC16B	-2.128513761	-1.473516167	1.60E-12	1.97E-11	Down
PRPH	3.514569656	2.803649427	2.55E-06	1.30E-05	Up
AC131212.1	-2.12928255	-2.998582944	2.80E-08	1.97E-07	Down

NBEAP1	-2.129751829	-2.511857002	5.05E-05	0.000199757	Down
C10orf55	3.511052862	-0.294179383	6.15E-06	2.92E-05	Up
NLRP2	-2.129979191	0.262054084	1.22E-05	5.47E-05	Down
AL512430.3	3.509785976	-3.033436284	0.000146341	0.000524853	Up
AL353678.1	3.509637854	-1.872446467	6.56E-08	4.34E-07	Up
AL596244.1	-2.130374196	3.008258953	4.13E-19	1.02E-17	Down
FOLR1	3.509369317	2.450197586	0.00041678	0.001346161	Up
SERBP1P5	3.509139023	0.274994946	3.86E-15	6.29E-14	Up
AC092675.1	3.506307548	0.749575772	3.28E-07	1.96E-06	Up
AL034379.1	3.504618318	-0.478209712	3.11E-12	3.74E-11	Up
CDK2	3.503703992	5.257348147	6.11E-23	2.20E-21	Up
NRNPA1P	3.503598305	-2.546285099	4.86E-06	2.34E-05	Up
NAH17-AS	-2.130886865	-0.3870785	0.000590913	0.001845098	Down
AC011506.1	3.502127704	-2.175111024	1.77E-07	1.10E-06	Up
LINC01235	3.502036572	2.525220648	2.67E-05	0.000112139	Up
AL606469.1	-2.131932541	-2.789572834	9.07E-07	5.02E-06	Down
MICAL3	-2.132152652	4.541430485	1.99E-13	2.72E-12	Down
LBX2	3.501814257	-0.312069956	9.11E-05	0.000342268	Up
LINC00404	-2.13241567	-0.178283697	0.000330157	0.001093087	Down
CDC20	3.501707475	5.120799451	3.37E-09	2.69E-08	Up
AC026367.2	-2.132851567	-1.431132826	1.14E-08	8.44E-08	Down
RPL17P33	3.500623181	-2.557628402	1.03E-05	4.67E-05	Up
AC008026.2	3.497486162	-1.890569207	4.53E-08	3.08E-07	Up
MTRNR2L1	3.496382244	-2.556530716	4.71E-05	0.000187431	Up
AC126773.6	-2.134851011	-2.081342354	2.16E-10	2.04E-09	Down
AL158050.1	3.496276933	-0.824680104	2.73E-09	2.21E-08	Up
DHDH	-2.135859803	-0.367224429	2.77E-10	2.58E-09	Down
F2RL3	3.494086359	0.602798499	1.52E-05	6.70E-05	Up
AC097358.1	3.492458309	-2.552765367	3.85E-06	1.90E-05	Up
RPS15AP40	3.4912589	-2.557731123	1.45E-05	6.41E-05	Up
ERP27	3.490330281	0.986811135	8.38E-05	0.000317385	Up
GAPDHP69	3.489565824	0.255679011	3.29E-10	3.03E-09	Up
PLK5	-2.13613395	-0.404899924	8.43E-08	5.49E-07	Down
AC106872.6	3.487502413	-2.561787907	7.80E-06	3.63E-05	Up
RPS13P3	3.487319728	-3.047279878	9.73E-05	0.000363271	Up
AC135178.7	3.486637071	-2.563770379	8.32E-06	3.85E-05	Up
AC112187.1	3.486033107	-2.187866101	1.83E-07	1.13E-06	Up
BTF3P12	3.481829173	-2.548329332	1.36E-06	7.29E-06	Up
AP004245.1	3.480976597	-1.667074692	3.34E-12	3.99E-11	Up
ANKRD65	-2.138201924	3.052439943	1.56E-09	1.31E-08	Down

AC005300.1	3.480563735	-3.085095438	5.87E-05	0.000229433	Up
TMTC1	-2.139841592	4.271866556	1.52E-10	1.46E-09	Down
NHP2P2	3.480213889	-3.053846795	7.34E-05	0.000281198	Up
AL365436.1	3.480189561	-1.452657151	5.19E-09	4.04E-08	Up
USP43	-2.140268764	1.917338009	8.39E-05	0.000317711	Down
AL161909.1	3.475696244	0.237599762	7.04E-11	7.13E-10	Up
AC087752.1	3.474182566	-3.053897781	2.31E-05	9.82E-05	Up
MGP	3.473457723	7.101904435	0.000185892	0.000651526	Up
MACC1	3.473244901	0.433445419	2.36E-07	1.44E-06	Up
LINC0184[illegible]	3.472708175	-0.439580474	0.000125175	0.000455345	Up
CYP2A6	-2.141542738	-2.320535521	4.25E-06	2.08E-05	Down
AC099548.[illegible]	3.472500699	0.608959991	5.20E-06	2.49E-05	Up
GAPDHP4[illegible]	3.471986002	1.380078293	1.29E-14	2.00E-13	Up
LINC0070[illegible]	-2.141798112	-1.91171185	6.35E-05	0.000246596	Down
PPP1R9A	-2.143200672	4.645483221	1.12E-09	9.58E-09	Down
RPL23AP3[illegible]	3.470087725	-3.053397298	2.71E-05	0.000113648	Up
AL161636.[illegible]	3.469676488	-2.572552863	6.73E-06	3.17E-05	Up
AC004832.[illegible]	3.4696432	-2.580811642	0.000420575	0.00135781	Up
LMNB1	3.466784659	5.783362704	5.87E-11	6.01E-10	Up
WDR47	-2.143718072	5.561385893	7.13E-36	8.93E-34	Down
AC002456.1	3.466593582	1.432558406	4.04E-08	2.76E-07	Up
RPL26P19	3.466371483	2.319390301	5.02E-14	7.32E-13	Up
MPP2	-2.144140037	5.234770509	5.64E-17	1.13E-15	Down
MICOS10P[illegible]	3.466250883	-1.917095376	2.42E-08	1.71E-07	Up
AC004801.1	3.46561685	-1.918969607	2.35E-08	1.66E-07	Up
AC024619.[illegible]	3.465334084	-0.999678513	5.34E-11	5.50E-10	Up
RPL9P7	3.46412638	1.334740959	2.97E-11	3.16E-10	Up
RPL3P10	3.46216896	-3.073213233	6.57E-05	0.000254373	Up
RPS3AP37	3.46216609	-2.583490695	1.53E-05	6.73E-05	Up
RPL12P35	3.461114761	-1.673375268	1.17E-11	1.31E-10	Up
AL365258.[illegible]	3.460952717	-1.455258356	3.09E-09	2.48E-08	Up
NHP2P1	3.456627123	-0.262199245	2.06E-15	3.47E-14	Up
OMP	3.455431983	-2.610984751	0.00016167	0.000574045	Up
AL359091.1	-2.1450821	1.955742871	0.000803287	0.002428233	Down
DAAM2-AS	-2.145410728	1.956662206	8.84E-06	4.07E-05	Down
CXCR2	3.45241698	1.142919884	5.85E-06	2.79E-05	Up
AC108734.1	3.452313956	-2.573444798	1.58E-06	8.41E-06	Up
PA2G4P2	3.451103103	-1.68580521	1.02E-11	1.14E-10	Up
AL356056.[illegible]	-2.145444491	-1.308902035	6.67E-09	5.10E-08	Down
AC064799.1	3.450217721	0.925801892	1.66E-12	2.04E-11	Up

RPS20P12	3.447056275	-2.594044094	3.90E-06	1.93E-05	Up
RANP1	3.446209591	1.25900768	3.44E-18	7.72E-17	Up
AL590867.2	3.444941885	4.013071499	5.94E-15	9.52E-14	Up
TPI1P4	3.44451133	-3.084122338	0.000414687	0.001340754	Up
RPS3AP47	3.443865526	1.281435645	4.28E-10	3.87E-09	Up
AC103591.1	3.441141781	-2.582613418	2.16E-06	1.12E-05	Up
TMSB10P1	3.441107409	-0.05527268	1.93E-10	1.84E-09	Up
ENPEP	3.440798399	4.024452114	2.99E-10	2.77E-09	Up
HSP90B3P	3.440509118	-0.751228084	1.06E-10	1.04E-09	Up
XRCC6P2	3.440494797	0.965326693	9.11E-20	2.41E-18	Up
GPR141	3.440302104	-0.377189673	0.000113625	0.000417442	Up
NAP1L4P3	3.439991442	-1.145519296	4.69E-11	4.88E-10	Up
RPL12P32	3.439851789	-2.601244904	1.49E-06	7.93E-06	Up
AC011492.1	3.437446723	-3.095681942	0.000104075	0.000386098	Up
AC026462.1	3.436875486	-0.769330047	4.46E-12	5.26E-11	Up
AL590682.1	3.436214614	-1.483605764	1.43E-10	1.38E-09	Up
RTN2	-2.146268577	3.915048945	1.77E-15	3.00E-14	Down
LRRN4CL	3.435863767	3.662462332	1.10E-05	4.99E-05	Up
GAPDHP3.	-2.146739193	-3.229580497	2.01E-07	1.23E-06	Down
RPS26P2	3.433174185	-2.254291822	4.25E-05	0.000170564	Up
H3P36	3.43261211	-0.639907389	1.42E-15	2.45E-14	Up
MAGOH3I	3.43223902	-3.104594383	0.000167515	0.000592316	Up
AC021231.2	3.432036965	-1.701479246	5.79E-12	6.73E-11	Up
FN1	3.432030536	10.49492013	1.16E-07	7.42E-07	Up
PKMP1	3.431745342	0.847862269	7.14E-15	1.14E-13	Up
AC087650.1	3.429619834	-1.936383748	7.59E-09	5.75E-08	Up
AL109615.1	3.428693121	-2.235564216	7.22E-08	4.76E-07	Up
EIF2S3B	3.428210404	0.588983528	4.91E-12	5.76E-11	Up
RPS28P5	3.425419974	-1.328458969	1.28E-11	1.42E-10	Up
LLPHP3	3.424965154	-2.260302692	5.50E-08	3.68E-07	Up
AL358472.1	3.42445452	-1.326384157	1.52E-08	1.10E-07	Up
BPIFB9P	-2.147499353	-2.428971267	6.28E-09	4.82E-08	Down
RPL3P9	3.421778287	-1.018447427	2.11E-10	2.00E-09	Up
AL135903.1	3.42064749	-2.624839061	2.44E-06	1.25E-05	Up
UBTFL6	3.418797657	-1.321245407	5.12E-06	2.46E-05	Up
AC002075.2	3.417835795	-0.894904948	5.34E-11	5.50E-10	Up
AC096533.1	3.417534606	-0.88899666	3.66E-14	5.43E-13	Up
AL135818.1	-2.15064115	-1.988808551	4.51E-08	3.06E-07	Down
EEF1A1P8	3.416182879	1.410910961	2.81E-14	4.21E-13	Up
PPIAP50	3.415556305	-2.23390508	1.52E-07	9.55E-07	Up

IGSF8	-2.151813002	6.424327384	4.10E-26	2.00E-24	Down
AL135978.1	3.414431194	-2.617453438	7.87E-07	4.39E-06	Up
DAB1	-2.15340133	2.840114693	7.58E-06	3.53E-05	Down
PELATON	3.41368562	1.585419308	0.000535447	0.001686162	Up
RPL23AP74	3.413389558	-0.460844588	2.02E-13	2.75E-12	Up
SYNGR4	-2.153537322	-1.597561422	6.31E-07	3.59E-06	Down
PLAC8	3.412244535	2.113934739	0.000317636	0.001055449	Up
NES	3.411976412	9.582176271	1.56E-09	1.31E-08	Up
RPL10AP1	3.411911417	-2.2508074	2.20E-07	1.35E-06	Up
GAL3ST1	-2.153929511	2.972981402	2.78E-05	0.000116072	Down
CHIC1	-2.15405105	3.694942832	6.28E-38	9.52E-36	Down
RPL30P14	3.411150256	-2.249467771	2.54E-07	1.54E-06	Up
AC087286.2	-2.154995697	-3.210825944	6.26E-06	2.96E-05	Down
RPS3P7	3.410630292	-3.11055699	0.00057642	0.001804732	Up
LINC02743	3.409820446	-0.302172046	0.000132696	0.000479978	Up
PPIAL4G	3.408532795	-2.61936984	5.91E-07	3.38E-06	Up
AC079061.1	-2.155120941	-1.793677508	2.29E-06	1.18E-05	Down
KRT86	-2.155363669	-0.926091834	7.56E-05	0.000288922	Down
PRELID1P4	3.408148612	0.490469409	1.86E-18	4.31E-17	Up
HAUS1P1	3.407418401	-3.125702235	0.000200233	0.000696884	Up
RPL4P4	3.405762225	3.643625496	1.11E-20	3.19E-19	Up
EI24P2	3.403976093	-1.733757274	2.07E-08	1.48E-07	Up
MTCO2P2	3.403581829	1.24286205	1.29E-10	1.25E-09	Up
GAPDHP6	3.402747172	0.105359924	3.16E-13	4.24E-12	Up
NPM1P12	3.402699285	-2.618834875	9.29E-07	5.12E-06	Up
AMZ1	-2.156274445	2.124058434	2.81E-08	1.97E-07	Down
CCDC80	3.401978439	7.484017542	8.63E-12	9.78E-11	Up
APOBEC3C	3.401547983	5.382344421	2.89E-09	2.32E-08	Up
RPS2P1	3.399861337	-2.271626886	1.86E-07	1.15E-06	Up
CRISPLD1	3.399515829	7.035349001	5.52E-10	4.91E-09	Up
SFTPD	-2.157394897	-0.448343123	3.02E-06	1.52E-05	Down
GP6	-2.157463166	-1.94806079	3.13E-09	2.51E-08	Down
S100A10	3.399127854	7.75195485	3.00E-06	1.52E-05	Up
CD248	3.397284419	5.422784466	2.85E-07	1.71E-06	Up
NCOA4P3	3.394188409	-2.63157402	1.14E-06	6.20E-06	Up
AL390119.1	3.394001599	-3.110615488	0.000260934	0.00088746	Up
NAT8L	-2.157647012	6.755307971	4.44E-12	5.24E-11	Down
XPNPEP2	3.39333287	0.971676238	0.00089996	0.002688591	Up
AKR1B1P3	3.391287624	-2.633032142	1.17E-05	5.27E-05	Up
RPL13AP3	3.389244973	-0.696140524	3.93E-10	3.57E-09	Up

AL138693.1	3.389074838	-1.222838938	3.95E-09	3.13E-08	Up
AC114284.1	-2.158036126	-2.197729252	7.98E-09	6.03E-08	Down
AL035398.1	3.38879123	-1.547455668	6.04E-09	4.64E-08	Up
SERPINA5	3.387607391	3.355367162	0.000109127	0.000402508	Up
AC025458.1	3.386413276	-0.691256018	1.73E-11	1.90E-10	Up
FLT3	-2.160573974	-1.053429501	3.21E-06	1.61E-05	Down
PDCD5P1	3.384961955	-1.981406839	8.24E-09	6.21E-08	Up
PPIAP74	3.384956539	-2.26846915	1.60E-07	1.00E-06	Up
AC016687.1	3.384866713	-2.272222233	7.45E-06	3.48E-05	Up
CGREF1	-2.161501473	4.048853091	1.61E-10	1.55E-09	Down
AGMO	3.384554721	2.904813737	9.03E-05	0.000339733	Up
RPS26P13	3.383490237	-2.265351399	1.95E-05	8.39E-05	Up
ELAPOR2	-2.161831676	3.972253354	1.06E-09	9.07E-09	Down
HNRNPRP	3.382450842	-1.993170314	2.17E-08	1.55E-07	Up
CHN2	-2.163704803	3.960703285	1.91E-15	3.22E-14	Down
POTEG	3.381930916	-1.978892383	2.91E-09	2.34E-08	Up
ADIG	3.381630495	-1.525534499	0.000612029	0.001902169	Up
AC079944.1	3.380775894	-0.69852865	5.29E-10	4.70E-09	Up
TIAF1	-2.163720597	2.11322067	4.70E-13	6.18E-12	Down
FOXD2-AS	3.380253222	1.591122323	1.66E-05	7.23E-05	Up
RPL7P50	3.37951256	-2.633487792	2.25E-06	1.16E-05	Up
FGFR2	-2.167261039	5.574114865	1.01E-11	1.14E-10	Down
HSPA9P1	3.378695427	-0.089776387	5.69E-14	8.25E-13	Up
MARVELD	3.378509479	2.003508719	0.000986298	0.00291928	Up
GAPDHP6	3.378308683	1.749857609	7.33E-16	1.31E-14	Up
RPL9P28	3.378293478	-1.20603796	8.21E-08	5.36E-07	Up
SRP9P1	3.37700355	0.957261287	7.60E-15	1.20E-13	Up
AL590617.1	3.37566627	-2.645762447	3.36E-06	1.68E-05	Up
RPS3AP36	3.373745265	-0.910744005	4.69E-08	3.18E-07	Up
AC074085.2	3.373226344	-2.280366312	4.45E-07	2.60E-06	Up
GAPDHP2	3.372119894	-0.73844329	1.35E-10	1.32E-09	Up
CADM3-AS	-2.168736973	-0.118866964	0.000128672	0.000466945	Down
BX679664.3	3.372040629	1.735830637	5.74E-13	7.46E-12	Up
AL589987.1	-2.169853223	-0.001327229	1.64E-05	7.16E-05	Down
MYL6P3	3.370893202	-1.983181308	1.67E-07	1.04E-06	Up
AC069120.1	-2.170537927	-2.913755341	7.79E-05	0.000296895	Down
RPL4P2	3.370885654	-1.06267715	4.99E-10	4.46E-09	Up
AC023644.1	3.370759936	-1.22672219	1.56E-09	1.31E-08	Up
AC092754.1	3.370447349	-2.661071894	2.62E-05	0.000109974	Up
MIR924HG	3.370118697	1.585529248	0.000197512	0.000688245	Up

FTLP5	3.369544854	-1.56281393	3.77E-06	1.87E-05	Up
CCNQP3	3.369138187	-3.15148685	0.00037558	0.00122712	Up
PIGUP1	3.367608982	-3.137458292	0.000158143	0.000562627	Up
RPL12P12	3.365988604	-0.255422362	1.23E-10	1.21E-09	Up
EIF4A1P4	3.363836338	-0.106939286	3.84E-16	7.12E-15	Up
SCML2P2	-2.171873843	-2.654296389	5.77E-08	3.85E-07	Down
CENPF	3.362636683	5.676554001	3.01E-07	1.81E-06	Up
ELMOD1	-2.171903054	4.874698479	1.47E-07	9.24E-07	Down
RPS10P27	3.36176249	-2.660489245	2.79E-05	0.000116767	Up
PSMD8P1	3.361599781	-1.993941058	3.00E-09	2.41E-08	Up
FOXD1	3.361082202	3.235158916	3.55E-08	2.45E-07	Up
RPS26P39	3.3610333	-1.996666032	5.87E-06	2.79E-05	Up
SUN2	-2.17251043	7.066407251	1.18E-19	3.07E-18	Down
DEPDC1B	3.358406436	3.057788253	1.33E-08	9.72E-08	Up
SOCS1	3.358026351	2.275791744	4.61E-06	2.23E-05	Up
AP001636.3	3.355521292	-1.252324179	3.96E-05	0.000159904	Up
OSTCP4	3.35537473	-3.136589313	0.000191923	0.0006708	Up
SEM1P1	3.355249596	-2.021380517	7.21E-07	4.06E-06	Up
PPIAP35	3.353650065	-2.019558312	8.71E-09	6.54E-08	Up
PLEKHA4	3.350016018	6.372877189	7.38E-08	4.86E-07	Up
RBM8B	3.348747618	0.244182711	1.19E-16	2.32E-15	Up
RPL21P1	3.34870629	-2.293245029	9.07E-07	5.02E-06	Up
MTFR2	3.345305788	1.353137975	6.68E-13	8.62E-12	Up
NPM1P27	3.344573334	2.991089763	6.81E-21	2.00E-19	Up
AC010683.1	3.344540233	-3.14782337	0.000172788	0.000609913	Up
ID3	3.343957029	8.247396746	7.01E-11	7.10E-10	Up
H3P47	3.343869593	-0.507954783	3.20E-13	4.29E-12	Up
AL355802.1	3.34188328	-0.111019656	1.89E-16	3.59E-15	Up
TPM3P8	3.340332266	-1.386530351	1.49E-10	1.43E-09	Up
H3P23	3.340183313	-2.674996562	1.35E-06	7.27E-06	Up
LINC0254[illegible]	3.340085359	-1.090577753	2.16E-06	1.12E-05	Up
FBXO43	3.339219827	0.451150247	6.40E-07	3.63E-06	Up
RPS4XP20	3.339114187	-2.667537831	7.09E-06	3.33E-05	Up
GPX1P2	3.337381862	-0.846102879	4.11E-08	2.81E-07	Up
KIF15	3.336325447	4.068778563	1.16E-09	9.92E-09	Up
SUMO2P1	3.33523431	0.609625635	1.71E-23	6.52E-22	Up
RPL35P4	3.335229454	-3.151365029	0.000135121	0.000487954	Up
PDE1C	-2.172846897	4.725766924	1.52E-09	1.28E-08	Down
RAD51AP1	3.333649959	3.435605966	4.82E-13	6.32E-12	Up
AL008718.[illegible]	-2.17404834	-2.179320118	2.78E-08	1.95E-07	Down

AL731532.2	-2.174133074	-3.031995494	9.24E-07	5.10E-06	Down
LINC02586	-2.174246715	-2.300007319	3.44E-08	2.38E-07	Down
AC067904.2	3.332959674	-2.019173249	4.41E-09	3.47E-08	Up
DNAJC19P	3.332673107	-0.854905199	1.42E-10	1.37E-09	Up
UBA52P5	3.332509938	-0.628061965	3.88E-14	5.72E-13	Up
EFEMP1	3.332249129	8.511379275	3.10E-05	0.000128245	Up
AC097638.1	3.331510883	-2.013315799	2.99E-07	1.79E-06	Up
EEF1B2P2	3.330177182	-1.108844022	9.61E-09	7.17E-08	Up
LINC0155	-2.174534493	-0.166851512	2.80E-12	3.38E-11	Down
DBF4P1	3.328041684	-1.793483214	8.29E-08	5.41E-07	Up
AC020899.1	3.327376023	-0.536821938	4.80E-11	4.98E-10	Up
BEX2	-2.175153237	5.212863371	2.05E-09	1.70E-08	Down
HMGN2P7	3.327356574	-2.029836055	5.18E-08	3.48E-07	Up
AC061992.2	3.326909812	0.442576137	9.29E-05	0.000348428	Up
AC027514.2	-2.17541689	-3.156567591	4.59E-06	2.23E-05	Down
BX248409.1	3.32619453	-2.016181239	7.47E-08	4.91E-07	Up
AL139246.5	-2.175542575	-0.715965969	1.20E-08	8.83E-08	Down
GMPSP1	3.32546612	-1.581928713	1.03E-11	1.15E-10	Up
RPS20P24	3.32465821	-2.686397636	1.13E-05	5.09E-05	Up
RPL13AP2	3.323042043	-1.248711013	7.39E-10	6.47E-09	Up
KIFC2	-2.177791788	4.853221689	5.13E-14	7.47E-13	Down
AC010729.2	3.321415766	-0.421015232	1.21E-06	6.53E-06	Up
TCEAL5	-2.178820973	4.091078697	4.52E-11	4.71E-10	Down
THRAP3P1	3.320419305	-2.682233101	7.46E-06	3.48E-05	Up
AL133260.1	3.319642857	1.175671634	2.04E-09	1.68E-08	Up
PPP1R13B	-2.17897114	4.071455265	1.07E-22	3.80E-21	Down
AC040169.2	-2.180310783	-3.142039594	1.08E-09	9.27E-09	Down
AL024509.2	3.319104268	-3.164389108	0.000344853	0.00113587	Up
S100A9	3.31790366	5.640480442	0.000786499	0.002382484	Up
AC139143.1	3.317080119	-2.044494638	2.55E-08	1.80E-07	Up
ADAMTS19	-2.180783359	-0.504072907	0.000390813	0.001271124	Down
LINC02418	-2.181160795	-2.579991534	3.03E-06	1.53E-05	Down
GUCA1A	-2.181344571	-0.321372701	0.000948387	0.002817488	Down
AJ011932.1	3.316907739	-0.874106932	3.62E-05	0.000147634	Up
PKP1	-2.181353259	-0.015831346	0.0001639	0.000581173	Down
MT1P1	3.316348709	-2.692748699	0.000289265	0.000971431	Up
MT1XP1	3.315027123	0.571002823	6.82E-07	3.86E-06	Up
MTND2P28	-2.181452982	6.733321585	5.42E-09	4.20E-08	Down
SCAI	-2.181828154	3.73905774	3.80E-33	3.62E-31	Down
POM121L9P	3.312388939	2.170573367	0.000280591	0.000946382	Up

AC091435.2	3.31147883	-0.98201658	0.000279169	0.00094225	Up
AC005262.1	3.311121984	-2.034811452	1.45E-06	7.74E-06	Up
LINC02320	-2.182698262	-3.06572588	0.000305218	0.001019421	Down
SYNE1	-2.183195828	6.100516719	9.90E-17	1.94E-15	Down
RPL9P14	3.310915011	-2.338107366	2.72E-06	1.38E-05	Up
SYNDIG1L	-2.183345302	-0.132741332	3.05E-05	0.000126402	Down
AP000811.1	-2.183825222	-3.16962424	7.08E-08	4.68E-07	Down
PLEKHD1	-2.184243166	-0.682837065	2.74E-13	3.70E-12	Down
ATP5MFP5	3.310766431	-1.81872014	5.34E-07	3.07E-06	Up
MAPT	-2.185314327	7.720687466	6.78E-12	7.79E-11	Down
PLK1	3.310153326	4.913980813	1.62E-09	1.36E-08	Up
AC073072.1	3.309528329	-1.580995715	2.35E-05	9.95E-05	Up
SLAMF8	3.309244296	3.502738448	2.86E-05	0.000119343	Up
VNN2	3.308476679	2.020412281	2.16E-05	9.21E-05	Up
PLIN1	-2.18610762	0.80361509	1.42E-07	8.93E-07	Down
TIMP1	3.30553857	8.986618509	9.67E-05	0.000361456	Up
CFL1P3	3.305436999	-2.328590798	5.83E-08	3.89E-07	Up
ATP6V1A	-2.186178199	6.703819557	2.16E-49	1.02E-46	Down
HNRNPCP	3.304145145	-1.275708447	2.37E-11	2.56E-10	Up
TUB	-2.186558299	5.512327235	1.48E-15	2.55E-14	Down
PCBP3-AS1	3.302533604	-0.242276719	0.000650764	0.002009335	Up
RAB1C	3.301288807	-0.40732469	7.15E-17	1.42E-15	Up
BZW1P1	3.301055	-2.058629175	2.28E-08	1.62E-07	Up
LINC02084	3.299588901	-1.650244557	0.000170174	0.000601202	Up
HNRNPCP	3.299085328	-2.040390722	8.39E-09	6.32E-08	Up
CFI	3.296863056	5.878014551	1.17E-05	5.28E-05	Up
RPS26P11	3.294921488	-1.808879488	1.26E-05	5.66E-05	Up
CLEC18C	3.293349249	-0.521434558	3.24E-05	0.000133399	Up
HNF4G	3.293211964	2.46123402	1.91E-08	1.37E-07	Up
CPVL	3.292820816	6.28428635	5.92E-07	3.38E-06	Up
TTBK2	-2.187255772	4.12451818	3.34E-40	6.51E-38	Down
AC090772.3	-2.187425775	-1.22715155	4.70E-16	8.59E-15	Down
EFR3B	-2.1884345	5.669763114	9.20E-24	3.63E-22	Down
UGT8	-2.18864732	5.6177844	0.000124314	0.000452498	Down
RPS15AP6	3.292354765	-2.732724281	0.000306651	0.001023496	Up
UBA52P6	3.290331593	-1.145497604	3.23E-10	2.97E-09	Up
AC104339.1	3.290132605	-2.08147169	1.13E-05	5.09E-05	Up
Z99129.4	-2.189159506	3.323764575	8.13E-18	1.77E-16	Down
AC103724.4	3.28882471	-2.726346487	0.000126501	0.00045988	Up
LGI1	-2.189296585	3.963085381	1.59E-07	9.97E-07	Down

RPS4XP1	3.288751889	-0.463531665	6.58E-12	7.57E-11	Up
LINC0150(	3.28729191	-1.620922181	0.000258402	0.000879774	Up
AC069213.:	3.285920539	-1.644818469	3.95E-06	1.95E-05	Up
HSPB1P1	3.28571936	0.817182094	4.51E-09	3.54E-08	Up
H3-5	3.284290239	0.922735211	2.58E-21	7.92E-20	Up
NRNPA1P	3.284191125	-2.061818908	6.54E-08	4.34E-07	Up
DDX12P	3.284132502	1.621408586	4.28E-06	2.09E-05	Up
NODAL	3.283590326	1.66433898	0.000201289	0.000700195	Up
DPYS	-2.190616165	-1.359924614	4.66E-07	2.71E-06	Down
RPS3AP48	3.283376446	-2.726584169	0.000119845	0.000437891	Up
CLIC1	3.282802715	7.957330573	1.45E-11	1.60E-10	Up
MYO15A	-2.190695897	0.586928221	1.43E-08	1.05E-07	Down
COX20P2	3.280597495	-2.356984313	1.27E-05	5.68E-05	Up
AC011495.:	3.280333485	1.378120088	6.95E-14	1.00E-12	Up
CERNA1	-2.192500983	-0.296447895	6.17E-13	7.98E-12	Down
BRIP1	3.279868429	2.763906599	7.98E-09	6.03E-08	Up
FBLIM1	3.279723994	5.074143105	2.68E-05	0.000112334	Up
PNMA8C	-2.19321845	2.102758732	1.01E-14	1.58E-13	Down
AC105021.:	3.279570602	-2.361983434	4.95E-08	3.34E-07	Up
S100A3	3.276138669	2.289839585	1.16E-05	5.24E-05	Up
KCNIP3	-2.193355213	4.126682429	8.19E-09	6.18E-08	Down
AL049836.1	-2.193739932	-2.078620618	2.04E-05	8.74E-05	Down
NDUFB1P:	3.27558522	-1.446224415	2.89E-08	2.02E-07	Up
FTH1P4	3.272957205	0.834095948	1.00E-12	1.27E-11	Up
PSMD12P1	3.268902938	-3.195273707	0.000312976	0.001042192	Up
HSPA8P5	3.26854469	0.658339644	7.40E-16	1.32E-14	Up
HP3-ACAI	-2.197354669	-2.945571305	1.28E-09	1.09E-08	Down
ANK2	-2.197762072	7.670960112	1.18E-17	2.54E-16	Down
BTF3P13	3.268531171	-2.716991463	3.87E-06	1.91E-05	Up
AC006122.:	3.265509733	-1.1547061	2.18E-10	2.05E-09	Up
HSPD1P5	3.265460007	-1.646518785	7.01E-11	7.11E-10	Up
COL6A6	-2.198198019	-2.025136033	2.07E-07	1.27E-06	Down
AC025871.:	-2.198247537	-3.021251561	3.55E-07	2.11E-06	Down
GPX1P1	3.264715497	3.653771529	6.67E-05	0.00025775	Up
RPL35P1	3.264685119	1.064177159	8.86E-10	7.67E-09	Up
HSPA6	3.264252274	4.185324914	3.08E-05	0.000127396	Up
DRP2	-2.19865216	3.644338213	2.19E-10	2.07E-09	Down
GBP5	3.263960605	2.694101544	0.000142653	0.00051284	Up
PCNAP3	3.263705459	-3.206976796	0.000792272	0.002397953	Up
AC124016.:	-2.19925424	0.740154946	2.50E-10	2.34E-09	Down

HMGN1P3	3.262293449	-0.791415194	3.82E-14	5.64E-13	Up
POLQ	3.262117266	2.089360241	8.07E-10	7.02E-09	Up
LINC0122	3.261675038	1.520010305	0.000771832	0.002342733	Up
PRKN	-2.199411335	2.851206638	7.33E-28	4.09E-26	Down
RPL3P2	3.260575437	1.412617574	1.84E-16	3.50E-15	Up
LAMB4	3.260456339	-0.354000987	1.15E-07	7.36E-07	Up
RPS3AP40	3.257516486	-3.213499686	0.000873658	0.002618111	Up
C4orf45	-2.201241463	-3.187416531	6.75E-08	4.47E-07	Down
RPL23AP1	3.256425545	-0.906236975	3.34E-13	4.48E-12	Up
VEZF1P1	3.255689377	-2.731862918	1.42E-05	6.32E-05	Up
HNRNPKP	3.255373013	1.946582286	7.58E-25	3.28E-23	Up
AC005099.	3.253684475	-0.931821962	3.30E-09	2.64E-08	Up
AC022483.	3.25061949	-3.210020158	0.000185629	0.000650695	Up
TEAD2	3.246235173	5.029653571	5.26E-07	3.03E-06	Up
EIF5AP2	3.244362437	-1.662214284	1.50E-09	1.26E-08	Up
PPM1AP1	-2.201864535	-3.361701144	2.33E-05	9.88E-05	Down
AL592295.1	3.244160529	-0.823394521	4.00E-15	6.52E-14	Up
CIT	-2.202089047	5.506094352	2.23E-14	3.37E-13	Down
EEF1A1P2	3.243729342	-3.206796397	0.000151648	0.000542064	Up
AC087442.	-2.203560284	-2.773007095	7.74E-06	3.61E-05	Down
LINC02521	3.243068836	-2.760097667	0.000230121	0.000791064	Up
GAPDHP6.	3.23998953	3.00322102	7.26E-15	1.15E-13	Up
AC015688.	3.239100059	-3.229500205	0.000936778	0.002787598	Up
CDKN2C	3.238589783	6.638922692	1.13E-08	8.33E-08	Up
RPL37P23	3.238478092	-0.305616904	1.47E-12	1.82E-11	Up
MTCYBP1	-2.203735333	0.655245005	2.69E-09	2.18E-08	Down
TOB2P1	3.237990506	-0.214744998	1.70E-06	9.00E-06	Up
AC005330.	-2.20546268	1.387688625	2.15E-11	2.33E-10	Down
ACTBP1	3.237916414	-0.620587106	7.39E-13	9.49E-12	Up
NAMPTP1	3.237572746	3.268778994	6.39E-05	0.000247836	Up
H3C10	3.236439348	0.887234079	2.23E-05	9.50E-05	Up
RPL13AP1	3.23595952	-3.211527845	0.000327346	0.001084652	Up
PPP3R1	-2.205694898	6.153681489	6.45E-55	6.00E-52	Down
CEP126	-2.20580062	2.947092623	1.59E-06	8.46E-06	Down
RPS15AP1	3.235364183	-0.539708919	1.95E-10	1.85E-09	Up
AC104667.	-2.206272656	-2.196974412	1.91E-08	1.37E-07	Down
GABRB1	-2.207053565	1.407446427	8.22E-06	3.81E-05	Down
NEK2	3.23422972	3.48924393	3.61E-08	2.49E-07	Up
LINC0201	3.234106151	-2.776755492	0.00047386	0.00151071	Up
ITPRIPL1	3.234000835	3.585410308	1.67E-13	2.31E-12	Up

PPIAP44	3.232739277	-3.215057209	0.000467943	0.001494328	Up
DYSF	-2.207565175	4.463850259	2.31E-11	2.49E-10	Down
IL12A-AS1	-2.207600138	-1.695200577	3.72E-10	3.39E-09	Down
BANF1P3	3.231285849	0.229976544	1.42E-15	2.44E-14	Up
GLRA4	-2.208209954	-2.713806168	1.84E-06	9.63E-06	Down
AC107954.1	3.231152314	-0.954411816	5.48E-09	4.25E-08	Up
HCG11	-2.208639483	1.384505532	7.57E-09	5.73E-08	Down
LDHAL6A	-2.209611432	-2.22525398	1.06E-08	7.89E-08	Down
KMT5AP2	3.22941523	-1.907835671	1.85E-05	7.99E-05	Up
HMGB3P6	3.229042683	-2.757581611	2.22E-05	9.47E-05	Up
NRM	3.227283914	4.700146052	6.08E-17	1.21E-15	Up
AC044810.1	3.227215996	-3.227650546	0.000376837	0.001230529	Up
TCF19	3.227202648	4.849883224	5.79E-11	5.94E-10	Up
EPOP	-2.210081484	3.072377375	1.03E-12	1.30E-11	Down
RPL15P3	3.225499201	3.546109961	5.26E-17	1.06E-15	Up
CYP2T1P	-2.210650684	-0.325641011	2.72E-21	8.29E-20	Down
AP000942.1	3.225294552	-2.396725847	3.48E-06	1.73E-05	Up
DEGS2	-2.211802319	0.766089852	4.02E-10	3.65E-09	Down
FSCN1P1	3.223461706	-0.072613722	2.66E-10	2.49E-09	Up
AC092447.2	3.223230729	-0.838193755	1.14E-08	8.43E-08	Up
EIF5P1	3.223011389	-2.409478757	1.07E-06	5.82E-06	Up
ELOVL4	-2.213489645	3.355578497	5.10E-16	9.28E-15	Down
AL391416.1	3.219812772	0.269771253	3.76E-12	4.46E-11	Up
SLC16A4	3.218715612	4.91190955	1.98E-08	1.42E-07	Up
LINC01995	3.2174057	-2.14686808	0.000377524	0.001232352	Up
CBX2	3.215763567	3.886511733	1.76E-06	9.27E-06	Up
AL109766.1	3.215585762	-0.95873381	5.64E-13	7.34E-12	Up
MORF4	3.215341297	-1.076767168	8.33E-11	8.33E-10	Up
STON1	3.214584894	4.400631198	4.20E-08	2.86E-07	Up
RPL7P1	3.212438495	2.43112076	2.52E-15	4.19E-14	Up
RPS4XP21	3.207138593	-3.239782996	0.000267301	0.000906332	Up
AL645608.8	-2.213512628	-2.842174367	1.12E-06	6.09E-06	Down
RNF165	-2.215671491	3.972694015	2.67E-06	1.36E-05	Down
SYT14	-2.216489775	2.146204919	9.41E-08	6.09E-07	Down
AK4P3	3.206682345	-2.782106947	3.10E-05	0.000128152	Up
FTH1P11	3.205883976	2.352316754	7.69E-13	9.83E-12	Up
S100A11	3.204162126	7.290171947	5.54E-08	3.71E-07	Up
ATP2B1	-2.21728273	6.45754675	6.17E-23	2.22E-21	Down
RPL17P22	3.203460592	-2.415501094	4.39E-06	2.14E-05	Up
FAM107B	-2.217379734	5.598737751	1.20E-15	2.09E-14	Down

CCL28	-2.218365425	-0.391914599	1.55E-05	6.83E-05	Down
RANP6	3.202893326	-1.690297786	2.13E-09	1.75E-08	Up
FAM133DI	3.202483013	-1.713647908	1.53E-07	9.62E-07	Up
HLA-DQA	3.201109393	6.312378894	2.29E-05	9.72E-05	Up
GSN-AS1	-2.218545692	-0.705294834	4.75E-12	5.58E-11	Down
FOLH1	-2.219037461	3.27206744	8.74E-08	5.68E-07	Down
HMGB1P1	3.198908317	-3.227758542	0.000307239	0.001025288	Up
NPM1P35	3.19884541	-2.774599046	3.37E-06	1.69E-05	Up
ST13P3	3.197834458	-0.969422761	9.48E-12	1.07E-10	Up
IAN2A1-D	-2.219123655	-1.338852744	8.51E-12	9.66E-11	Down
TULP4	-2.219287756	4.857162952	6.24E-25	2.74E-23	Down
SPATA12	3.197516056	-1.236456119	7.98E-06	3.71E-05	Up
RPL31P11	3.196215057	-2.147364966	3.93E-07	2.31E-06	Up
PLEK2	3.196119655	1.7671488	5.77E-08	3.85E-07	Up
PCBP3	-2.221009399	2.290636031	2.64E-09	2.14E-08	Down
RPS10P28	3.195802758	-1.688527141	2.25E-07	1.37E-06	Up
MKRN2OS	-2.221887767	-0.94758898	3.19E-12	3.82E-11	Down
CNOT7P1	3.195676951	-2.781029864	3.08E-06	1.55E-05	Up
KIAA0040	3.19557422	5.711338378	1.09E-07	6.99E-07	Up
SLAMF7	3.195312322	0.739036035	9.72E-05	0.00036295	Up
AC093677.	3.195292623	-2.771559238	1.63E-05	7.15E-05	Up
HLA-DRA	3.193466139	9.815366899	2.58E-06	1.32E-05	Up
AURKA	3.192457265	3.989902056	5.70E-12	6.63E-11	Up
RIMKLA	-2.222150826	3.69966752	1.96E-13	2.68E-12	Down
AC117382.	3.192095235	-0.867751618	1.81E-14	2.76E-13	Up
FABP5P11	3.190964661	-2.150415929	0.00067655	0.002080818	Up
MAP1LC3C	3.190337943	1.703276682	0.000223926	0.000771284	Up
LBX2-AS1	3.186891192	1.665582569	6.40E-08	4.25E-07	Up
GLULP3	3.18646672	-1.388302772	4.61E-06	2.24E-05	Up
MYRF-AS1	-2.222628211	-3.103793777	4.07E-06	2.00E-05	Down
AC090543.	3.185883104	-0.988916099	1.81E-06	9.52E-06	Up
AC027309.	3.184261348	1.765476077	2.83E-16	5.33E-15	Up
PPIAP66	3.182948506	-1.723487305	1.19E-08	8.77E-08	Up
LINC0063	-2.223100955	0.888868095	0.000103602	0.000384591	Down
CDC42P6	3.182843996	1.455372168	2.05E-15	3.45E-14	Up
SEPTIN7P	3.181742772	-0.08833528	1.16E-10	1.14E-09	Up
LINC0147	-2.224142771	-3.404328123	0.000292443	0.000980845	Down
EPHA7	-2.224287267	2.251856349	0.000123506	0.000449844	Down
FO393411.	3.181145678	1.06364157	2.09E-15	3.51E-14	Up
AC026367.	-2.225479158	-1.351025373	6.14E-10	5.43E-09	Down

TPRN2-AS	3.177942757	1.644980978	1.26E-06	6.80E-06	Up
RPL6P30	3.177664375	-2.789532824	1.98E-05	8.53E-05	Up
RPL14P1	3.177205933	3.411739003	2.55E-20	7.07E-19	Up
CSRP2	3.176578403	7.071606922	5.19E-08	3.49E-07	Up
INRNPDP	3.17599561	-2.158890487	3.22E-08	2.23E-07	Up
SMIM3	3.174659914	6.376149971	1.25E-06	6.76E-06	Up
AL365366.1	3.172558196	-1.723821899	1.56E-09	1.31E-08	Up
RPL7P52	3.171960274	-2.787537289	2.74E-05	0.000114835	Up
TDGP1	3.171266081	-2.790796324	1.90E-05	8.21E-05	Up
TNFRSF9	3.171191563	-0.454483181	0.000738643	0.00225148	Up
AC005531.1	3.170224215	-2.807458662	0.000877245	0.002627814	Up
PPIAP58	3.168337379	-2.45197588	1.82E-06	9.57E-06	Up
AC104390.1	3.167276768	-2.164836784	6.44E-08	4.28E-07	Up
AC004692.1	3.166683396	-3.256537312	0.000698947	0.002141813	Up
SLC9A6	-2.226414289	5.635957137	1.85E-36	2.41E-34	Down
CMIP	-2.226613436	6.110867291	5.16E-28	2.93E-26	Down
HENMT1	-2.226634238	2.109772647	4.44E-10	3.99E-09	Down
PLK2	-2.227406393	5.010012856	2.17E-11	2.35E-10	Down
PSME2P2	3.16533249	0.78986749	1.17E-12	1.47E-11	Up
DELEC1	3.165043909	-0.46692272	1.21E-05	5.43E-05	Up
ARF4P1	3.164777249	-1.56209879	6.04E-09	4.64E-08	Up
RPL22P16	3.161542467	-2.44348786	2.19E-07	1.34E-06	Up
AC145098.2	3.160165481	-0.148304946	9.97E-05	0.000371421	Up
LINC01978	-2.227605063	-1.527258544	1.54E-07	9.67E-07	Down
AC011416.2	3.15901974	-2.430313556	1.61E-07	1.00E-06	Up
SEPHS1P6	-2.227953584	-0.952403379	2.83E-16	5.33E-15	Down
ASAP1-IT2	3.158899671	-1.131049837	5.30E-05	0.000208933	Up
PKMP5	3.158726029	-1.244865347	3.19E-09	2.55E-08	Up
CLMAT3	3.158219052	-1.95719544	1.52E-07	9.52E-07	Up
EPHX4	-2.228553459	1.78718457	1.20E-13	1.68E-12	Down
RPL26P30	3.157704757	-0.545670823	8.20E-14	1.17E-12	Up
IQGAP3	3.157551528	4.225610179	1.63E-08	1.18E-07	Up
DNAJB7	-2.229704092	-2.666473318	5.20E-09	4.05E-08	Down
UNC5B-AS	-2.230230777	-2.098634194	3.14E-07	1.88E-06	Down
RPL5P24	3.153420892	-1.250288097	6.04E-08	4.03E-07	Up
AC005840.1	3.153094318	-0.409697795	6.84E-09	5.22E-08	Up
CMTM3	3.151169218	6.425075615	2.64E-24	1.09E-22	Up
SLC46A2	-2.230248482	-2.125578881	6.32E-06	2.99E-05	Down
AC120036.4	-2.231864742	1.676932109	8.66E-14	1.24E-12	Down
AC012558.1	3.15075893	-1.312663078	0.000252917	0.000862433	Up

ACTN4P1	3.150749179	-1.016271741	5.20E-10	4.64E-09	Up
RPL7AP60	3.150107828	-1.157089391	1.52E-10	1.47E-09	Up
RPL19P21	3.148743633	-0.624468669	3.86E-13	5.14E-12	Up
CD99	3.14764522	9.311584879	5.47E-11	5.64E-10	Up
RPS15P5	3.147450961	-0.912333667	1.45E-11	1.60E-10	Up
IFITM4P	3.147213474	-2.192308562	0.000353203	0.001161116	Up
LINC0194	3.144343655	-0.928477004	4.29E-05	0.000171945	Up
RPSAP5	3.143725137	-1.267406131	4.30E-08	2.93E-07	Up
TBX2-AS1	3.140934323	1.894501379	1.88E-08	1.35E-07	Up
ATDN1P	3.14024965	-1.950186545	2.65E-08	1.87E-07	Up
AC080038.	3.13896231	1.41910332	2.46E-07	1.49E-06	Up
AC121493.	-2.232288536	-0.864418639	3.05E-08	2.13E-07	Down
APOC1P1	3.138784732	-0.211430024	2.00E-05	8.59E-05	Up
SOCS2	3.138666353	6.006073912	0.000430744	0.001386134	Up
Z95327.2	3.13855121	-2.462225592	8.33E-07	4.63E-06	Up
AC007066.	3.138472134	-1.751060781	1.90E-07	1.17E-06	Up
AL589674.1	3.13791005	-1.946499665	1.78E-08	1.28E-07	Up
TAGLN2	3.136709721	8.913512686	1.03E-09	8.89E-09	Up
RND1	-2.232632174	3.840439457	2.14E-13	2.92E-12	Down
AC004884.	3.133676793	-2.465412636	1.04E-05	4.74E-05	Up
NF114-AS	3.133629935	-2.209153669	0.000456833	0.00146274	Up
MTHFD2	3.133294709	5.97386659	2.84E-09	2.29E-08	Up
AP001469.1	-2.233460816	-2.665570799	5.70E-11	5.85E-10	Down
TUBB3P1	3.133280501	-2.832529606	4.92E-05	0.000195142	Up
ANP32C	3.132836579	-1.748725178	6.68E-09	5.11E-08	Up
LINC0177	3.132156213	1.627185995	6.82E-06	3.21E-05	Up
PAH	-2.233649303	0.102311225	4.38E-06	2.14E-05	Down
RPL23AP9	3.130147859	-2.831164995	7.84E-05	0.00029865	Up
LINC0044	-2.233832664	-2.834307595	0.000104132	0.000386258	Down
ATP5F1CP	3.129653603	-1.416773739	3.34E-10	3.07E-09	Up
AKAP11	-2.233908103	6.08129882	2.57E-40	5.07E-38	Down
FLRT1	-2.23439463	3.063123309	3.05E-07	1.83E-06	Down
CD44	3.129032787	9.273605961	3.17E-07	1.89E-06	Up
PPIAP46	3.128986072	-1.318085877	2.79E-05	0.00011669	Up
RAPGEF2	-2.234681276	5.649850748	1.56E-34	1.73E-32	Down
HMGN2P3	3.128192547	-2.496524519	5.45E-06	2.61E-05	Up
EEF1A1P3	3.127976956	-0.835411044	1.08E-10	1.07E-09	Up
C1QTNF1	3.126351673	6.437299818	0.000151683	0.000542121	Up
DRG1P1	3.125284967	-1.765887832	2.44E-09	1.99E-08	Up
SPARC	3.12473337	11.85565263	1.28E-14	1.99E-13	Up

GINS2	3.122670422	4.246874235	7.19E-10	6.30E-09	Up
POPDC3	-2.235292124	1.538223439	2.37E-05	0.000100414	Down
SLC25A5P7	3.121704745	-2.821200602	3.28E-05	0.000134914	Up
PSAT1P3	3.120367055	-1.432558779	2.68E-07	1.62E-06	Up
SDHDP2	3.118569686	-2.837713507	2.91E-06	1.47E-05	Up
AC074276.1	3.117248189	-2.829894637	1.69E-05	7.36E-05	Up
PA2G4P1	3.116913973	-1.776566549	4.43E-09	3.48E-08	Up
EIF5AP4	3.116462194	-0.283189755	3.30E-17	6.76E-16	Up
LDHBP2	3.116365461	0.064746264	2.89E-14	4.32E-13	Up
LRRTM4	-2.235523	2.768867089	1.31E-05	5.85E-05	Down
RPL23AP61	3.115658404	-2.831727047	1.54E-05	6.77E-05	Up
FOXD4L6	-2.235595359	-3.382799721	6.09E-05	0.000237109	Down
AL391219.1	3.115333621	-2.828159876	3.94E-05	0.000159333	Up
FDPSP1	3.113459447	-2.211134221	1.71E-07	1.06E-06	Up
AC104985.1	-2.235765951	-1.784105127	4.67E-09	3.66E-08	Down
AC078817.1	3.110560717	0.074961927	2.29E-10	2.15E-09	Up
AC137932.2	-2.236140549	-1.603300676	4.16E-12	4.92E-11	Down
AC113367.1	3.109914176	-1.591306458	1.00E-07	6.45E-07	Up
RPL7P47	3.109619553	-0.943165546	1.30E-10	1.26E-09	Up
AC138466.1	-2.236329045	-3.462218428	5.62E-05	0.000220214	Down
SIT1	3.109487549	-0.627066375	0.000194323	0.000678366	Up
AIPL1	-2.236791475	-3.184153217	0.000152649	0.000545141	Down
DNAJC12	-2.237107967	2.953576023	4.15E-16	7.65E-15	Down
RPL4P5	3.107208893	1.612882882	4.05E-16	7.47E-15	Up
GOT2P2	-2.237988484	-2.837165746	4.65E-11	4.84E-10	Down
AP000943.2	-2.238408141	-3.164929186	1.78E-06	9.38E-06	Down
IGFBP7	3.105212892	9.650253328	4.97E-08	3.35E-07	Up
H2AC11	3.105045301	1.139996879	4.13E-06	2.03E-05	Up
PIF1	3.104252778	2.698273437	1.62E-07	1.01E-06	Up
ATP5F1EP	3.103077856	0.949245424	7.65E-14	1.10E-12	Up
PYGL	3.101751137	6.299587077	5.14E-10	4.59E-09	Up
BAIAP3	-2.238899268	5.901924417	2.61E-07	1.58E-06	Down
EIF1P7	3.100187141	-1.287660857	5.71E-11	5.87E-10	Up
GBP2	3.099141215	6.127155287	6.53E-07	3.70E-06	Up
ATP11A-AS	-2.239468658	-3.536271444	9.30E-06	4.26E-05	Down
RPL7AP11	3.098754401	0.341858763	1.99E-11	2.16E-10	Up
AL132640.1	-2.239602196	-2.625408754	6.59E-10	5.80E-09	Down
GPX7	3.098113135	4.787166109	1.30E-12	1.62E-11	Up
OPRL1	-2.241521428	3.409982574	1.66E-23	6.34E-22	Down
MAP3K7CL	3.095747938	4.126824209	1.48E-05	6.54E-05	Up

RPL15P5	3.095384966	-0.9532523	1.54E-11	1.69E-10	Up
EGFLAM	3.094616739	3.145527792	7.21E-05	0.000276693	Up
AL132656.4	-2.241809011	-0.775451601	5.00E-18	1.11E-16	Down
BEST3	3.092619516	4.219398474	0.000203106	0.000705862	Up
AL121956.4	-2.242010996	-2.734746889	1.75E-06	9.21E-06	Down
BFSP1	-2.24309797	0.460488219	8.84E-09	6.64E-08	Down
AL139100.1	3.092592113	0.983656098	1.40E-10	1.36E-09	Up
PAQR9-AS	-2.244840919	-1.052817432	1.47E-06	7.87E-06	Down
GBP3	3.087661423	5.33528646	0.000310609	0.001035027	Up
HSPA8P16	3.08616503	-2.232946722	1.21E-06	6.57E-06	Up
SCD	-2.244968582	8.726888213	1.78E-15	3.02E-14	Down
AC108134.2	3.085999805	-1.338846428	0.000153876	0.00054887	Up
RPL23AP59	3.083858127	-3.299662999	0.000649561	0.002005834	Up
KRT8P13	-2.245004409	-3.55460351	1.74E-05	7.58E-05	Down
PTMAP2	3.08329724	2.023972928	1.08E-17	2.33E-16	Up
WNT5A-AS	3.081675116	-0.903384159	2.38E-06	1.22E-05	Up
SOX4	3.081114142	7.324528084	1.80E-05	7.80E-05	Up
TXNP1	3.073745274	-3.308189607	0.000942583	0.002803136	Up
GREM1	-2.245353513	2.253616391	0.00011043	0.000406745	Down
TENT5A	3.072885278	5.010359414	7.25E-10	6.35E-09	Up
AC064875.1	3.06951928	3.097571323	0.000202657	0.000704639	Up
AC025062.1	3.06874223	-1.798697817	7.75E-08	5.08E-07	Up
AL137025.1	-2.246347977	-0.736849837	7.94E-09	6.00E-08	Down
HSPB1P2	3.068071102	-0.193765829	2.58E-09	2.10E-08	Up
AL356019.2	-2.247216436	0.04120172	2.89E-19	7.25E-18	Down
FCGR2A	3.067030977	6.254415283	1.61E-06	8.53E-06	Up
OR1H1P	-2.247989641	-3.142556405	0.000327163	0.001084171	Down
GPR27	-2.248196734	2.80084425	8.51E-06	3.93E-05	Down
DIXDC1	-2.248563412	4.995545114	1.78E-33	1.75E-31	Down
IGFL2-AS1	-2.250319573	-3.407823523	0.000314309	0.001046028	Down
UBE2NP1	3.065074602	-2.878288949	6.26E-05	0.00024305	Up
RPL7AP50	3.06502235	1.38599219	7.79E-13	9.96E-12	Up
FKBP9P1	3.064777414	2.360859631	0.000125489	0.000456372	Up
ANG	3.064287459	2.31111839	1.30E-08	9.54E-08	Up
MIR4482	-2.251687967	-2.823817964	1.68E-06	8.88E-06	Down
LRRC17	3.062949077	4.705848547	0.000142582	0.000512664	Up
PPIAP26	3.062544759	-2.871471762	4.23E-05	0.00016983	Up
SEMA3B	-2.252541379	5.780830661	5.21E-12	6.10E-11	Down
RDH10	3.061804708	6.250166635	2.10E-05	8.96E-05	Up
AC112491.1	3.060115817	-0.038001497	7.53E-11	7.59E-10	Up

SMO	3.059796506	6.215686619	3.84E-08	2.64E-07	Up
GAPDHP7	3.057476936	-0.330037725	1.38E-10	1.34E-09	Up
RPSAP39	3.05685073	-1.829522839	2.82E-07	1.70E-06	Up
ATP6V0A1	-2.25331439	7.149991045	1.14E-40	2.32E-38	Down
SETP14	3.056283653	1.09165341	1.10E-15	1.91E-14	Up
MFAP4	3.056274904	5.966570329	0.000479965	0.001527469	Up
AC005696.4	-2.253320702	2.292966791	3.88E-07	2.29E-06	Down
LGI4	-2.254837191	4.473599518	3.13E-07	1.87E-06	Down
AC243960.2	3.055985198	-0.27014451	5.14E-12	6.02E-11	Up
MCOLN2	3.054955881	0.500876239	0.00011986	0.000437891	Up
RPL4P3	3.053089943	-0.71363453	5.71E-12	6.63E-11	Up
LINC01127	3.050313074	-0.024967705	6.22E-05	0.000241857	Up
GOLGA6L	-2.255175032	-2.768507583	4.14E-07	2.43E-06	Down
PACRG-AS	-2.255235926	-1.371482438	6.37E-06	3.01E-05	Down
RPL3P7	3.049915886	1.255979539	4.37E-13	5.77E-12	Up
PPIAP51	3.048127044	-2.041015747	1.76E-07	1.09E-06	Up
ACOT7	-2.255559227	5.730886098	1.44E-19	3.70E-18	Down
RPL7P20	3.046575882	-2.888795421	0.000316297	0.001051742	Up
SH3PXD2A	-2.255645503	5.194446351	1.97E-12	2.41E-11	Down
ST13P19	3.045890488	-0.91077366	6.89E-12	7.90E-11	Up
PROS1	3.044006903	6.098405943	4.21E-07	2.47E-06	Up
HSPA8P9	3.042443942	0.334159535	1.80E-14	2.74E-13	Up
RHGAP11	3.036785947	4.181807774	4.98E-11	5.16E-10	Up
AC073335.1	3.036634891	-1.832211359	2.61E-08	1.84E-07	Up
LCN8	-2.256765616	-3.048487745	4.07E-05	0.000164069	Down
MAPK9	-2.257384504	5.724685663	3.20E-71	4.61E-67	Down
MYL8P	3.036450111	-2.538846079	6.03E-06	2.86E-05	Up
AL034417.1	3.034303364	-2.265376195	9.34E-07	5.15E-06	Up
FOXP4-AS	3.033907791	-1.494646624	4.21E-06	2.06E-05	Up
AC022182.3	3.031911503	-1.133465655	5.20E-10	4.64E-09	Up
ENO2	-2.257518202	8.075887139	8.69E-16	1.54E-14	Down
RPS27AP10	3.031698312	3.636741969	9.72E-15	1.53E-13	Up
FBLL1	-2.257807434	2.946453811	3.08E-06	1.55E-05	Down
LINC01841	3.028918832	-1.857827166	5.88E-06	2.80E-05	Up
RPL22P1	3.027226319	1.790209488	2.76E-14	4.14E-13	Up
AC046195.1	-2.25783366	-3.206776343	3.16E-06	1.59E-05	Down
EEF1DP5	3.026722687	-2.893873073	0.000580046	0.001814705	Up
AC095032.1	-2.258027002	-3.003339147	7.70E-07	4.31E-06	Down
HHLA2	-2.259140494	-2.965626219	5.10E-07	2.95E-06	Down
ANK1	-2.259674126	2.629173532	1.55E-08	1.12E-07	Down

NAIPP1	3.023986881	-1.379234485	0.000795859	0.002407547	Up
AL009174.1	3.021571191	1.336834691	2.41E-10	2.26E-09	Up
PHBP21	3.021168028	-2.041034035	4.93E-08	3.33E-07	Up
KHSRPP1	3.020944501	-1.152766447	7.40E-05	0.000283607	Up
RPL18AP15	3.020642888	-2.891435347	0.000253992	0.000865689	Up
AJUBA	3.020023335	3.540630845	7.78E-10	6.79E-09	Up
AL355472.1	3.018557395	1.458454991	1.57E-14	2.41E-13	Up
CDHR1	-2.26003691	3.50525696	1.79E-07	1.11E-06	Down
OMG	-2.260872584	5.230195466	2.27E-09	1.86E-08	Down
LPO	-2.263095873	-1.57832785	1.39E-09	1.17E-08	Down
MYL12BP2	3.01787423	-2.278156131	1.83E-06	9.61E-06	Up
EME1	3.016455237	2.736365585	1.82E-09	1.51E-08	Up
GNRHR2P	3.012744425	-2.32172302	6.04E-07	3.44E-06	Up
RPS3AP22	3.012426049	-2.559103061	5.71E-05	0.000223736	Up
UBE2SP1	3.012194559	0.942090134	1.58E-08	1.14E-07	Up
BTF3P9	3.011104311	-2.273947153	7.93E-07	4.42E-06	Up
LSMEM2	-2.263541441	-0.832948591	2.17E-10	2.04E-09	Down
AC234778.1	3.010740235	-2.906325044	0.00014429	0.000518143	Up
ATF4P3	3.010707346	0.027566184	2.69E-14	4.03E-13	Up
RPLP1P5	3.009809499	-2.904126805	0.000110431	0.000406745	Up
ISL2	3.009788315	0.772107838	0.000103063	0.000382737	Up
BTN2A3P	3.008957544	1.674806939	1.35E-13	1.88E-12	Up
RPL23AP96	3.008169082	-2.89924238	2.83E-05	0.000118269	Up
AC005822.1	3.007564039	-0.680025303	7.51E-16	1.34E-14	Up
NIBAN1	3.006346003	5.632364547	4.63E-06	2.24E-05	Up
AC007560.1	3.004074395	-1.274705597	5.53E-13	7.19E-12	Up
ZDHHC8P	-2.263952645	2.339567612	6.09E-07	3.47E-06	Down
C1orf226	3.003916324	5.052109074	3.07E-11	3.26E-10	Up
FMN1	-2.263961795	2.220279477	6.15E-11	6.27E-10	Down
AC093503.2	-2.264064919	-0.644351432	6.10E-06	2.90E-05	Down
TTC7B	-2.264352	5.105680615	4.04E-36	5.15E-34	Down
RPS27AP12	3.003504102	-1.51737173	3.75E-08	2.58E-07	Up
PALM	-2.266749465	6.388307462	2.08E-13	2.84E-12	Down
RPS27AP1	3.002444723	-1.391719369	1.07E-07	6.85E-07	Up
GNAZ	-2.267016678	5.094406306	5.11E-21	1.53E-19	Down
CAMTA2	-2.268754014	5.741537167	1.09E-43	2.85E-41	Down
CDCA7L	3.00240222	5.290826618	1.27E-07	8.09E-07	Up
CALM2P3	3.001523455	-0.219982721	4.77E-11	4.95E-10	Up
AC005746.2	3.000205663	-2.069725739	0.000819588	0.002474446	Up
RPL23AP12	2.999798674	-2.554098802	4.39E-06	2.14E-05	Up

S100A2	2.999595966	2.026718888	7.39E-07	4.15E-06	Up
TMSB10P2	2.998866929	-2.566383072	3.17E-05	0.000130659	Up
C1R	2.99842614	8.213145586	2.16E-05	9.19E-05	Up
AC026111.1	2.998266117	-2.293877796	9.29E-07	5.12E-06	Up
AFAP1L1	2.997633087	4.784865718	4.50E-07	2.63E-06	Up
PATE2	-2.269749018	-3.289577992	0.000398368	0.00129297	Down
RPL31P7	2.997197238	-2.907680891	9.14E-05	0.000342959	Up
SPCS2P1	2.996633597	-2.911275146	3.00E-05	0.000124626	Up
EDARADD	2.993928658	0.393220312	0.000147381	0.000528124	Up
PDE4DIPP	-2.270487434	-2.566759778	1.56E-05	6.86E-05	Down
AC093591.1	2.99386554	-1.561094587	6.31E-07	3.59E-06	Up
TSPAN6	2.993330778	6.555300793	1.81E-13	2.49E-12	Up
STC2	2.99166641	4.390069457	0.000283614	0.000955347	Up
LINC01303	2.990985199	-1.319271221	0.000812905	0.00245576	Up
MISP	-2.270765433	-2.403859723	4.74E-07	2.75E-06	Down
CASP4	2.989195352	4.835846847	9.59E-09	7.16E-08	Up
LPCAT4	-2.271231169	4.529689114	7.49E-35	8.43E-33	Down
PPP1R14B	2.988807073	6.415012769	3.98E-16	7.35E-15	Up
SPSB4	2.988164256	4.036609026	2.15E-06	1.11E-05	Up
KBTBD11	-2.272288906	5.691374959	1.03E-14	1.62E-13	Down
HSPD1P7	2.987919912	-2.918832732	3.15E-05	0.000130133	Up
IPO7P2	2.986547502	-1.424694972	6.24E-09	4.80E-08	Up
RPS24P17	2.985491038	-2.292875697	5.24E-06	2.52E-05	Up
AL031658.1	-2.272680721	-2.247356556	1.16E-09	9.92E-09	Down
VEGFA	2.984611554	8.818778604	0.000223263	0.000769456	Up
GAPDHP2	2.984161666	-0.52528955	1.33E-11	1.48E-10	Up
AC026410.1	2.983200251	-0.77114978	8.07E-13	1.03E-11	Up
AL391056.1	-2.273009659	-2.344865751	8.55E-06	3.95E-05	Down
AC092798.1	2.983165603	-1.721971875	6.17E-07	3.51E-06	Up
AL117339.3	-2.273333617	-0.253239827	4.19E-13	5.56E-12	Down
NDUFA5P1	2.981633358	-1.199638628	2.93E-10	2.72E-09	Up
ADGRB3	-2.275048345	4.72843514	2.12E-15	3.57E-14	Down
F8A3	2.980927338	-1.729844786	3.71E-07	2.20E-06	Up
AC026469.1	-2.276999111	-2.682694801	2.30E-05	9.77E-05	Down
LINC00643	-2.278917867	2.50986022	0.000186146	0.000652269	Down
PRDX2P1	2.980432815	-1.893380144	6.45E-08	4.28E-07	Up
RPLP0	2.978418975	10.61130453	1.62E-13	2.24E-12	Up
PPP1R1A	-2.279884961	3.267278334	0.000175093	0.000617218	Down
RGS14	-2.280912816	3.203769589	6.75E-14	9.73E-13	Down
RPL7P26	2.977883081	-1.581644893	1.47E-07	9.23E-07	Up

LYPLA2P2	2.977072608	-2.594620655	7.26E-06	3.40E-05	Up
CRY2	-2.283876522	5.263312569	2.45E-26	1.21E-24	Down
PPIAP43	2.976539224	-1.288887341	5.67E-10	5.04E-09	Up
PTTG3P	2.976053278	-2.584591676	6.55E-05	0.000253475	Up
AL034397.1	2.975313215	-1.564554288	3.97E-09	3.14E-08	Up
TSPAN12	2.974893973	5.437390521	2.63E-06	1.34E-05	Up
GAPDHP5	2.974744686	-2.316457289	1.63E-06	8.61E-06	Up
MEM72-AS	-2.284097645	-1.077594928	1.73E-14	2.64E-13	Down
AC131009.2	-2.284441568	-2.444913005	1.94E-05	8.37E-05	Down
CALM2P4	2.974348641	-2.078238612	1.63E-06	8.65E-06	Up
AL359918.2	2.974008401	0.421604695	1.50E-16	2.89E-15	Up
TOLLIP	-2.284522749	6.73377194	2.08E-41	4.51E-39	Down
RPL7P48	2.970466776	-2.08856995	1.34E-06	7.23E-06	Up
AC133528.1	-2.285711573	-3.126549407	1.38E-08	1.01E-07	Down
LRRC74A	2.968824208	-2.117188958	0.000349332	0.001149571	Up
RUNX1T1	-2.287352625	2.680041308	1.37E-09	1.16E-08	Down
HECTD4	-2.287826327	5.91395546	1.89E-27	1.04E-25	Down
KRT19	-2.288236641	-1.206699791	3.58E-05	0.000146066	Down
AC020893.2	2.967100153	-2.327633671	4.66E-08	3.16E-07	Up
CYP51A1P	2.966880251	-1.737621166	2.28E-06	1.17E-05	Up
CENPI	2.966268614	2.250565769	5.00E-10	4.47E-09	Up
AL137782.1	-2.289450689	-0.977550479	4.18E-22	1.39E-20	Down
PPIAP6	2.965561063	-0.48159336	1.60E-10	1.54E-09	Up
AC233964.1	2.965268491	-1.074004045	2.96E-08	2.07E-07	Up
RPL15P20	2.963619282	-1.732551439	4.58E-07	2.67E-06	Up
CXCR4	2.962163695	6.205771515	2.67E-08	1.88E-07	Up
SRP14P2	2.962097417	-0.092714673	6.29E-12	7.27E-11	Up
AC098847.1	2.961321877	-1.894034377	1.70E-07	1.06E-06	Up
CLIC5	-2.289607379	0.922238166	1.43E-08	1.04E-07	Down
AFAP1-AS1	-2.290370428	-1.371308813	9.60E-16	1.68E-14	Down
CACNA1D	-2.290446223	2.681814842	9.69E-11	9.62E-10	Down
TERF1P4	2.960410129	-2.616808734	3.18E-06	1.59E-05	Up
CASP1	2.959582593	4.6911747	2.28E-07	1.39E-06	Up
RPS6KA6	-2.291155122	1.570566671	4.68E-05	0.000186324	Down
XRCC6P1	2.959374912	-1.581036719	8.10E-09	6.12E-08	Up
PPP1R1B	-2.293498425	5.648121402	2.79E-05	0.000116693	Down
H2AZP3	2.959086584	-0.493609625	1.79E-10	1.71E-09	Up
VWC2L	-2.293510942	0.917937512	1.06E-05	4.79E-05	Down
ADGRE1	2.95836412	1.967663383	0.000600959	0.00187261	Up
SUMO2P6	2.956918068	-2.088179373	3.17E-08	2.21E-07	Up

SHC3	-2.294186429	5.788701372	1.13E-12	1.42E-11	Down
LINC0163(	-2.294425226	0.255083528	8.84E-05	0.000333137	Down
AC010978.:	2.956780889	-2.956976344	8.80E-05	0.000332039	Up
LGALS3	2.956437591	7.850683883	0.000387659	0.001262577	Up
AC010469.:	2.955850155	-1.610943104	9.14E-07	5.05E-06	Up
AL662791.1	2.955835492	-1.450355571	2.42E-06	1.24E-05	Up
SEZ6L2	-2.295349665	6.383780819	4.20E-09	3.32E-08	Down
KRT81	-2.295649147	-2.17489608	5.98E-05	0.000233084	Down
CCNB1	2.954622324	5.694209901	5.36E-09	4.15E-08	Up
FCER1G	2.953370606	6.323529144	7.61E-07	4.26E-06	Up
EIF2S2P4	2.952058566	0.592725996	4.42E-17	8.93E-16	Up
RPS10P2	2.951554915	-0.583550595	7.22E-09	5.49E-08	Up
YWHAG	-2.296540137	8.506645109	8.59E-46	2.81E-43	Down
C2CD2L	-2.299415038	4.565484449	2.12E-51	1.13E-48	Down
BTRC	-2.300075491	4.947979992	1.18E-46	4.24E-44	Down
RPL29P11	2.949693079	0.599700659	4.45E-13	5.86E-12	Up
RPL17P36	2.949557385	-0.031653322	1.17E-09	9.95E-09	Up
ALDOAP1	2.949114407	-1.58261749	5.17E-09	4.02E-08	Up
EIF4A1P5	2.94868848	-2.112738942	1.98E-07	1.22E-06	Up
CCT5P1	2.948177695	-2.094976196	1.15E-07	7.35E-07	Up
CTIF	-2.301128177	5.677675926	2.62E-27	1.42E-25	Down
NRNPA1P	2.946767987	-1.100633564	3.13E-12	3.76E-11	Up
PDE11A	-2.301139926	0.057586873	3.30E-06	1.65E-05	Down
ARHGEF2:	-2.302180521	2.538444413	3.06E-13	4.11E-12	Down
AC134043.:	2.945274182	-0.077323555	4.40E-08	3.00E-07	Up
WASF4P	2.94518647	-1.319185584	1.47E-07	9.25E-07	Up
ACTR3P2	2.945025418	-2.946830545	0.000192191	0.000671653	Up
RPL21P17	2.9443149	-2.34597385	1.37E-05	6.09E-05	Up
HCN2	-2.302239519	5.043352931	1.28E-08	9.36E-08	Down
LC25A38P	2.943916279	-2.62236084	2.32E-06	1.19E-05	Up
ITGB3	2.943323685	1.923185225	3.34E-05	0.000137077	Up
GAPDHP7(	2.942637303	-0.085997954	1.16E-09	9.91E-09	Up
HIVEP2	-2.302563354	5.065462012	1.74E-23	6.62E-22	Down
AC099522.:	2.942623327	-2.960060395	9.46E-05	0.000354231	Up
SH3BP5	-2.303851461	2.846583144	1.48E-14	2.28E-13	Down
H3P44	2.942142452	-2.10538996	1.81E-08	1.30E-07	Up
RPL7P23	2.942133959	0.542268776	7.08E-12	8.11E-11	Up
AC007685.:	2.937504369	-2.332456288	7.20E-06	3.37E-05	Up
IKBIP	2.937401408	4.577710553	9.33E-15	1.47E-13	Up
ARHGEF3:	-2.305057084	-0.11914419	1.55E-23	5.97E-22	Down

LINC0112[illegible]	-2.305501134	0.116216384	6.01E-08	4.01E-07	Down
LDB2	-2.305716847	4.277678354	1.33E-12	1.65E-11	Down
CD1D	2.936032201	1.43381451	6.51E-05	0.000252226	Up
AC008443.[illegible]	-2.306034412	-3.288955085	1.91E-07	1.18E-06	Down
ARHGEF[illegible]	-2.306287546	6.034149416	1.53E-30	1.12E-28	Down
NAMPT	2.935905216	8.830124125	0.000265215	0.000899896	Up
AC116347.[illegible]	2.935440488	0.064229279	1.83E-10	1.74E-09	Up
CADPS	-2.306482422	5.54729555	4.21E-08	2.88E-07	Down
CT75	-2.307413108	0.377020557	6.32E-07	3.59E-06	Down
TLR8	2.934980375	2.116124184	0.000512626	0.001622275	Up
RAC1P5	2.933812931	-2.343247955	4.40E-07	2.57E-06	Up
RPL10P12	2.931132536	-1.756346151	1.40E-06	7.51E-06	Up
LAMP3	2.930545409	0.814312006	1.24E-06	6.68E-06	Up
AL008726.1	2.929523662	-1.004997621	5.77E-05	0.000225733	Up
Z97200.1	2.928372011	0.546350344	0.000365635	0.001198159	Up
HLA-DMB	2.928102837	6.385053572	2.32E-06	1.19E-05	Up
BZW1P2	2.927242005	1.366518863	8.10E-16	1.43E-14	Up
RPS3AP11	2.925823014	-2.965507884	0.000245439	0.000840014	Up
ARHGAP3[illegible]	-2.308092042	5.531297423	1.14E-24	4.87E-23	Down
AC005070.[illegible]	-2.308126322	0.503000658	1.45E-20	4.13E-19	Down
ROGDI	-2.308388449	5.319638405	1.00E-27	5.53E-26	Down
AC006970.[illegible]	-2.309559784	-2.878248663	9.37E-10	8.10E-09	Down
AEBP1	2.925190151	8.932543088	2.85E-05	0.000118804	Up
PPL	-2.309888946	3.62911908	3.57E-06	1.77E-05	Down
RPS29P8	2.923916089	-2.948651953	0.000301348	0.001007898	Up
PPIAP42	2.923560945	-1.940283085	8.02E-08	5.25E-07	Up
RPS19P3	2.922504975	0.332473663	6.13E-12	7.09E-11	Up
WDR7	-2.310433534	5.068498741	1.34E-54	1.07E-51	Down
ABCC6P2	2.921535808	-0.848362787	5.23E-05	0.000206382	Up
SNX5P1	2.921270867	-1.598866672	2.23E-09	1.83E-08	Up
HAS2-AS1	2.919245329	1.618979172	6.09E-10	5.38E-09	Up
TUBB6	2.919233007	6.211851134	2.89E-06	1.46E-05	Up
RPL15P13	2.917613302	-2.634873847	1.62E-05	7.11E-05	Up
FKBP10	2.917288209	7.753451961	2.14E-12	2.61E-11	Up
CHST9	2.917176557	4.389300317	0.0009698	0.00287547	Up
ITGA4	2.916799907	3.512967977	1.87E-05	8.08E-05	Up
TNFSF13B	2.91539782	4.406113595	3.26E-06	1.63E-05	Up
ARL6IP1P[illegible]	2.914929641	-1.62294912	5.48E-09	4.24E-08	Up
NPM1P46	2.914780946	-1.765225366	1.97E-08	1.41E-07	Up
AL450405.1	2.914683486	3.983124555	9.47E-11	9.40E-10	Up

AL845552.2	-2.31118994	-3.106971267	1.96E-07	1.21E-06	Down
RPL7P32	2.914681536	-0.409591683	2.91E-10	2.70E-09	Up
RPL23AP32	2.912613231	-1.774759187	6.21E-05	0.00024147	Up
VAMP1	-2.311551782	3.987386283	5.01E-17	1.01E-15	Down
AC018371.2	2.912595857	-2.62958148	8.47E-06	3.92E-05	Up
RPL31P63	2.910000034	-0.410307756	2.12E-08	1.51E-07	Up
H3C9P	2.907765425	-1.229733554	1.66E-11	1.83E-10	Up
GJA1P1	2.907009249	-0.567567112	1.41E-05	6.25E-05	Up
VCAM1	2.905753006	6.073308793	6.27E-05	0.000243756	Up
AL359091.2	-2.312245108	-0.703215761	8.01E-08	5.24E-07	Down
HMGN1P4	2.90536603	-2.655859234	1.47E-05	6.50E-05	Up
RHEBP1	2.904724447	-1.773389033	1.73E-07	1.07E-06	Up
SEMA3D	-2.312248148	1.909034376	0.000396639	0.001287998	Down
S1PR3	2.903755063	6.401386992	2.15E-08	1.53E-07	Up
WDR76	2.901921351	4.191991156	4.60E-11	4.79E-10	Up
LINC02198	2.900810784	-1.181401166	3.81E-05	0.000154797	Up
ITGA5	2.900244542	6.458831914	3.85E-07	2.27E-06	Up
APBB1	-2.312315955	7.045695323	2.54E-34	2.74E-32	Down
GPR158	-2.313644867	4.382836991	1.12E-11	1.26E-10	Down
SLC25A5P8	2.899433908	-2.981638887	0.000160909	0.000571483	Up
FTH1P2	2.898818615	2.531132577	1.64E-11	1.81E-10	Up
C4orf47	2.898326009	2.258149288	1.61E-06	8.53E-06	Up
RPL21P39	2.898087672	-1.64194425	1.64E-06	8.68E-06	Up
SLC11A1	2.89793692	6.056793368	2.04E-05	8.76E-05	Up
C6orf118	2.8978814	1.446054817	0.000165194	0.00058504	Up
TPO	-2.31380569	-2.856224975	8.45E-05	0.000319799	Down
AF201337.1	2.896760812	0.281914647	1.09E-08	8.11E-08	Up
CENPH	2.89673033	3.870419374	6.55E-10	5.77E-09	Up
YBX1P10	2.895930042	2.805983083	1.02E-14	1.59E-13	Up
SUB1P3	2.89584523	-1.143294413	4.58E-08	3.11E-07	Up
RPS8P4	2.895581028	-2.16255646	3.70E-05	0.000150502	Up
RPL6P25	2.895470626	-2.992393542	0.000129943	0.000471142	Up
RPL35P2	2.894649503	0.620113398	5.21E-10	4.64E-09	Up
NPIPB10P	2.894146416	-2.673072078	0.000127815	0.000464187	Up
AP002784.2	2.892973046	0.946220312	1.49E-12	1.85E-11	Up
AC011477.5	-2.314008766	-3.293331269	1.06E-05	4.82E-05	Down
AL031985.2	2.892885693	-1.635944378	7.30E-07	4.10E-06	Up
KCNH7	-2.314908847	-0.106715345	1.13E-05	5.09E-05	Down
ADAM12	2.892520743	5.174102371	7.26E-05	0.000278329	Up
TCHH	-2.314938804	-1.280340958	1.07E-07	6.88E-07	Down

KCNQ1DN	-2.315760532	-1.652813707	2.51E-05	0.000105609	Down
EIF1P5	2.891566769	-1.479860359	4.28E-09	3.37E-08	Up
AL603825.1	2.891554379	-0.943225233	5.84E-10	5.18E-09	Up
NRNPA1P	2.88822479	0.599970569	1.05E-14	1.64E-13	Up
PSAT1P4	2.887957084	-2.662573626	0.00011128	0.00040961	Up
ANXA2R	2.887771673	2.742508727	2.23E-06	1.15E-05	Up
AL139156.2	2.887744528	-1.369112708	1.11E-08	8.21E-08	Up
SNX32	-2.315980581	2.682349084	5.91E-17	1.18E-15	Down
ELN	2.887568445	7.592865418	2.40E-06	1.23E-05	Up
AC091563.1	-2.317196896	0.274948135	3.71E-12	4.41E-11	Down
RPL5P12	2.886033476	-1.128316908	2.83E-08	1.98E-07	Up
HMGB1P4	2.885855342	-2.372421892	2.65E-06	1.35E-05	Up
TMEM59L	-2.318464709	6.998648203	1.19E-19	3.11E-18	Down
PNOC	-2.318567888	1.827161305	0.000309915	0.001033194	Down
RPL7AP7	2.884802536	-3.004150515	0.000402137	0.001303982	Up
RPL7P6	2.884715039	-0.623949514	9.55E-10	8.25E-09	Up
SORCS3	-2.318874374	3.524579855	1.96E-07	1.21E-06	Down
AC011005.4	2.884002868	-1.026126437	0.000411476	0.001331567	Up
RUNDC3B	-2.318909531	2.643958654	1.76E-15	2.99E-14	Down
CACNB3	-2.319427176	4.659587749	5.32E-23	1.93E-21	Down
AC113404.3	2.88343523	0.592888679	3.30E-10	3.03E-09	Up
FANCD2	2.883209714	4.211812583	1.72E-10	1.65E-09	Up
ERBB4	-2.320370167	4.067150267	1.44E-08	1.05E-07	Down
LRIT2	-2.320633459	-2.051602665	8.62E-06	3.98E-05	Down
HMGN2P6	2.882931647	-1.970174284	1.11E-07	7.11E-07	Up
ATP5POP1	2.88290265	-2.985828467	9.04E-05	0.000339947	Up
SV2A	-2.321029709	7.202815031	1.56E-29	1.04E-27	Down
AC092692.1	-2.322695964	-2.249537135	1.25E-11	1.39E-10	Down
AC009019.1	-2.323481491	-2.178977851	1.34E-16	2.58E-15	Down
MYO1D	-2.323897101	3.924217962	8.59E-12	9.75E-11	Down
MTCO1P53	-2.323922394	1.546392758	2.69E-11	2.88E-10	Down
CAMK2G	-2.325029646	6.195191575	3.30E-20	9.07E-19	Down
CD8B2	-2.326155679	-2.667291453	0.000110296	0.000406351	Down
TNFRSF10	2.881942641	1.521739032	3.93E-06	1.94E-05	Up
CTNNA3	-2.327229023	2.645016803	2.49E-06	1.27E-05	Down
FAM241B	-2.327820997	3.140268279	1.51E-12	1.87E-11	Down
PPIC	2.880483202	4.406259393	4.35E-10	3.93E-09	Up
AC116158.1	-2.328106486	-2.301863804	8.06E-14	1.15E-12	Down
RPS3AP13	2.879652535	-1.813959007	8.17E-08	5.34E-07	Up
GPR37	-2.329184763	6.007958283	1.48E-09	1.24E-08	Down

NT5C1A	-2.329869404	-1.164767709	9.59E-05	0.000358681	Down
AC025459.1	2.878329322	-2.66034108	0.00033986	0.001121732	Up
FLRT2	-2.331177958	4.016661719	1.20E-07	7.66E-07	Down
NRNPABI	2.877621221	-2.674464147	2.92E-05	0.000121547	Up
DGKZ	-2.333071629	6.691954522	2.11E-29	1.38E-27	Down
NRNPA1P	2.876173905	-1.964776881	1.52E-08	1.11E-07	Up
CNTNAP1	-2.333517504	6.193248458	5.30E-21	1.58E-19	Down
MIGA1	-2.334307652	4.429174588	3.28E-57	3.50E-54	Down
MEPE	-2.335503348	0.275721757	0.000236107	0.000810577	Down
PPIAL4C	2.876162871	-2.995576225	0.000137432	0.000495739	Up
TCP1P3	2.876160602	-1.500362456	4.71E-10	4.23E-09	Up
KCNH4	-2.335783528	0.286010155	6.40E-08	4.25E-07	Down
BTN3A2	2.875950623	6.051696815	4.05E-09	3.20E-08	Up
AC025171.5	2.875759367	0.263581214	0.000535216	0.001685617	Up
MRTFB	-2.337636835	6.011575688	7.00E-29	4.35E-27	Down
PTK2B	-2.338894255	6.035040185	8.36E-17	1.64E-15	Down
HMGN2P2	2.875331325	-3.010536783	8.04E-05	0.000305582	Up
YPEL2	-2.339633498	4.226644575	1.46E-36	1.92E-34	Down
SLAMF6	2.875225646	0.208354886	0.000369552	0.001209651	Up
MTRNR2L	2.87308054	-1.394729187	6.88E-06	3.24E-05	Up
SERPINA1	2.872619206	6.459789713	0.000115432	0.000423486	Up
NPM1P39	2.872053098	-0.03877973	4.02E-12	4.77E-11	Up
PSMB8	2.871885997	6.523969533	5.94E-09	4.57E-08	Up
RPL24P4	2.871108588	3.141925425	7.10E-11	7.18E-10	Up
AC095041.1	2.870928065	-2.995292209	0.000121731	0.00044405	Up
VAX2	2.869777541	2.456967994	1.61E-05	7.03E-05	Up
AP001065.3	-2.340075775	-3.347744677	0.000147194	0.000527517	Down
WAKMAR	2.869256515	0.414809675	6.57E-07	3.72E-06	Up
SMPD3	-2.341356214	3.144799858	5.43E-08	3.64E-07	Down
GPRASP2	-2.341859575	4.793258474	1.40E-49	6.85E-47	Down
RPS26P8	2.867097813	-1.287889292	5.90E-06	2.81E-05	Up
PACRG-AS	-2.342252232	-0.319597796	0.000108346	0.000400239	Down
NCAM2	-2.342914417	5.650556751	3.38E-12	4.04E-11	Down
TUBBP1	2.866740706	2.150183043	7.93E-16	1.40E-14	Up
RKAR1B-A	2.865761507	1.287856992	1.15E-05	5.18E-05	Up
LAMC1	2.865541958	7.47283259	1.18E-07	7.52E-07	Up
PSMA2P3	2.863342529	-2.408646285	5.10E-06	2.45E-05	Up
METTL21E	-2.343534312	-2.295662244	1.39E-11	1.54E-10	Down
CLEC18B	2.863313466	3.03128544	1.83E-05	7.93E-05	Up
GAPDHP2	2.861622256	-2.412088252	1.67E-05	7.29E-05	Up

AR	2.861023277	4.574635321	2.76E-06	1.40E-05	Up
EN2	2.859490787	2.471499186	0.000861058	0.00258416	Up
LY96	2.857916752	3.66579087	0.00010173	0.000378226	Up
PIEZO2	-2.343842918	2.914484468	1.49E-07	9.38E-07	Down
GPX3	2.857771885	7.375157836	0.000981671	0.002906779	Up
CD58	2.855526766	4.26991114	1.27E-08	9.35E-08	Up
NPTX2	-2.344990203	6.313070799	0.000353065	0.001160797	Down
AC012645.3	-2.346304134	-1.29743774	4.89E-16	8.92E-15	Down
AL356215.1	2.853819891	-0.329766899	0.00041448	0.001340534	Up
ANXA8L1	-2.346664285	-2.243646903	0.000229957	0.000790595	Down
LDHAP5	2.853455029	-0.84307384	7.46E-08	4.91E-07	Up
AC091799.1	2.85019285	-3.006424127	0.000212884	0.00073682	Up
PLEKHG5	-2.34686891	4.066182313	1.38E-18	3.24E-17	Down
RPS24P6	2.850050626	-3.024296329	0.000343218	0.001131261	Up
FAM177B	2.849818261	1.165656513	0.000108714	0.00040125	Up
AC022034.2	2.849666298	-2.6925689	0.000104409	0.000387237	Up
LINC00463	-2.346879547	-0.990027979	8.48E-06	3.92E-05	Down
PIGCP1	-2.346887808	1.303515069	6.17E-27	3.23E-25	Down
CAV1	2.84883401	6.801037475	0.000262945	0.000893666	Up
SNHG25	2.847615586	0.210975946	6.08E-05	0.000236878	Up
SILC1	-2.347608711	3.912413584	6.00E-07	3.42E-06	Down
RAB6C	-2.347935548	-0.944621793	9.04E-17	1.78E-15	Down
LINC02371	-2.352227284	-2.640684998	2.30E-11	2.48E-10	Down
GIF2-RAB5	2.847227824	-2.422369729	0.00035233	0.001158741	Up
LINC02397	2.845231505	-0.654522992	1.66E-05	7.23E-05	Up
CYGB	-2.352402676	2.988004215	9.30E-06	4.26E-05	Down
ALOX5AP	2.844368325	6.373824823	2.11E-05	9.02E-05	Up
SIDT1	-2.353213232	3.004182672	1.06E-08	7.91E-08	Down
AL357992.1	2.84431361	-1.840114941	0.000371497	0.00121488	Up
FZD7	2.843449345	5.699880863	6.70E-06	3.16E-05	Up
AC099654.2	2.842670388	-3.031839342	0.000108427	0.000400488	Up
ANKRD19P	-2.354101683	0.755128601	1.87E-28	1.11E-26	Down
PMEL	2.841842411	0.398535562	0.000113706	0.000417604	Up
DMBT1	-2.356552306	-1.094578307	1.32E-08	9.70E-08	Down
AC068522.1	2.839983577	-1.527683537	1.23E-05	5.53E-05	Up
EML5	-2.356658591	-0.491606548	2.38E-09	1.95E-08	Down
AL390728.1	2.839328314	-0.830685119	2.52E-10	2.36E-09	Up
HERC1	-2.357382462	5.593487907	8.53E-44	2.26E-41	Down
SNX6P1	2.839190324	-3.02390315	0.000182387	0.000640108	Up
H3-3A	2.838510733	6.02217202	1.75E-21	5.48E-20	Up

RORB-AS1	-2.359221953	-1.165425533	7.24E-07	4.08E-06	Down
AP001024.1	2.838447222	-0.131170503	1.08E-12	1.36E-11	Up
AC080023.2	2.838211474	-0.911269061	5.02E-09	3.91E-08	Up
IGSF10	-2.360050002	0.252112305	1.52E-10	1.47E-09	Down
AL358334.3	2.836220838	-2.446459872	9.11E-05	0.000342268	Up
TOMM20P4	2.833933658	-1.110820345	1.87E-08	1.35E-07	Up
KLHL2	-2.360184772	4.977727558	1.53E-30	1.12E-28	Down
RPS20P2	2.832489869	-3.023000228	0.0003818	0.001245323	Up
HIGD1AP1	2.830221225	-1.013964812	3.29E-08	2.28E-07	Up
AC005324.4	-2.360814976	-3.13668952	6.46E-07	3.66E-06	Down
SORCS2	-2.362578714	4.14813848	1.15E-11	1.29E-10	Down
LINC00951	-2.363161612	-1.806685059	8.57E-09	6.44E-08	Down
FANCI	2.829462354	5.195107691	2.55E-09	2.07E-08	Up
ZIC1	2.828470158	5.91768464	6.11E-07	3.48E-06	Up
LINC00642	-2.363957848	-2.562546804	4.74E-08	3.21E-07	Down
AC018647.1	-2.364777418	2.66423549	3.91E-11	4.11E-10	Down
GLULP4	2.828265362	-0.300255802	1.13E-07	7.21E-07	Up
HIGD2AP2	2.826117112	-3.038615903	0.000428709	0.001380203	Up
ZNF812P	2.82406335	0.919510072	0.000531168	0.001675247	Up
IL32	2.823182775	4.36135502	2.85E-05	0.000118912	Up
RNU6-529P	-2.3657195	-0.697413066	1.14E-10	1.12E-09	Down
AL596275.1	2.823029628	-1.201272352	3.71E-11	3.91E-10	Up
LGALS9B	2.822184584	-2.44548533	0.000150533	0.000538477	Up
GAPDHP4	2.821706411	-0.330367971	3.92E-10	3.56E-09	Up
RPL7P44	2.821210229	-2.005714392	7.78E-07	4.34E-06	Up
YBX1P2	2.820906192	1.480994414	1.09E-15	1.90E-14	Up
TMEM196	-2.366115441	1.594913073	0.000212851	0.000736797	Down
AC006927.1	2.820675864	-3.031849188	9.13E-05	0.000342953	Up
AC092667.1	-2.36675258	-1.528466942	3.71E-08	2.55E-07	Down
AC000067.1	2.820082861	-2.447612933	0.000978117	0.00289715	Up
SOSTDC1	-2.366932065	0.85676034	0.000325943	0.001080253	Down
FTH1P12	2.819674577	1.060839405	1.01E-10	1.00E-09	Up
ZNF804B	-2.367424524	-3.046678846	0.000248707	0.000849988	Down
TPRG1L	-2.368232883	5.685189803	1.47E-31	1.20E-29	Down
CCDC68	-2.36934958	0.075624772	4.11E-10	3.72E-09	Down
CACNA2D	-2.369781385	2.489312786	2.59E-07	1.57E-06	Down
AC107890.1	2.819068312	-3.037697982	0.000423179	0.001364703	Up
AL390783.1	-2.370179631	-3.075211263	3.03E-06	1.53E-05	Down
AC005884.2	2.818602725	-3.044752502	0.000631935	0.001957912	Up
AL512633.1	2.816538117	-1.432291539	2.68E-08	1.89E-07	Up

TRIM5	2.815060207	4.543047944	7.94E-12	9.05E-11	Up
ATCAY	-2.370697774	6.481650349	3.63E-05	0.000148005	Down
EIF3LP2	2.812282321	-1.329003114	2.15E-10	2.03E-09	Up
NRNPA1P	2.812170355	-2.707914888	1.36E-05	6.05E-05	Up
GDAP1	-2.371085665	5.016935489	5.88E-32	4.93E-30	Down
TSPYL4	-2.371298681	6.395039446	4.52E-32	3.84E-30	Down
TMEM63A	-2.372347023	5.286964849	1.33E-16	2.58E-15	Down
SLC24A4	-2.372463115	3.175084028	3.18E-06	1.60E-05	Down
AC106795.2	-2.373158504	-0.887507402	9.57E-10	8.26E-09	Down
WDR17	-2.374240797	3.520642474	5.74E-23	2.08E-21	Down
LINC0062	-2.375142259	-0.762292426	2.41E-10	2.26E-09	Down
LINC02391	2.811700554	-0.627780673	3.61E-06	1.79E-05	Up
NRNPA1P	2.811067932	-1.118278839	9.65E-10	8.33E-09	Up
SNRPA1P1	2.810804759	-1.437808751	2.39E-09	1.96E-08	Up
FAXC	-2.375221847	3.853162049	1.53E-19	3.94E-18	Down
AC093700.1	2.806869993	-2.029911979	3.95E-05	0.000159566	Up
BTBD9	-2.375456443	4.660061745	9.40E-36	1.15E-33	Down
PREPL	-2.376338312	6.767771849	2.13E-59	2.79E-56	Down
RPS3A	2.80662221	9.578662537	6.56E-08	4.34E-07	Up
HLA-DPA1	2.806312224	8.381183759	4.88E-06	2.36E-05	Up
PPIAP60	2.805572773	-2.234516664	2.39E-06	1.23E-05	Up
PRDX4	2.805482853	7.029148881	1.36E-09	1.15E-08	Up
TMEFF2	-2.376505085	4.307825842	3.52E-05	0.000143756	Down
PRCD	-2.376846262	1.170406446	1.57E-08	1.14E-07	Down
TBC1D9	-2.377962028	4.633852846	5.20E-23	1.89E-21	Down
NRG4	-2.379095781	-0.942256409	7.68E-11	7.73E-10	Down
LFNG	2.804520576	6.954311905	7.53E-06	3.51E-05	Up
NPM1P33	2.804494216	-2.447335042	6.65E-06	3.14E-05	Up
FTH1P1	2.804361901	-1.222617516	4.26E-08	2.91E-07	Up
TRPV4	2.801700289	1.007883802	1.38E-05	6.16E-05	Up
TGIF1	2.801487223	5.620421337	1.75E-18	4.05E-17	Up
UNC79	-2.379356204	3.424384139	1.26E-10	1.23E-09	Down
RPS15P4	2.801036957	3.617722641	4.86E-17	9.81E-16	Up
HNRNPLP	2.800616701	1.692321128	2.29E-22	7.90E-21	Up
FAM171A1	-2.379746506	5.639899652	2.22E-22	7.68E-21	Down
CDCP1	2.800444988	3.65049207	0.000190616	0.000666554	Up
SLIT2	-2.379824278	3.109094523	2.57E-06	1.31E-05	Down
AL031729.1	2.800416961	0.128656672	1.11E-10	1.09E-09	Up
ZNF540	-2.379897603	2.122694812	3.17E-29	2.03E-27	Down
CDK7P1	2.79980329	-3.058418095	0.000223938	0.000771284	Up

ACTBP14	2.798951011	-2.722108589	1.82E-05	7.90E-05	Up
VAMP8	2.798443913	5.448530137	1.66E-05	7.25E-05	Up
AC104297.1	2.798024209	-2.238477094	1.28E-06	6.89E-06	Up
CA4	-2.383047036	2.49012028	7.65E-08	5.02E-07	Down
ATP2C2	-2.384351167	0.421473948	2.53E-09	2.06E-08	Down
POLE2	2.796603377	2.45735421	2.77E-09	2.24E-08	Up
ACTG1P19	2.795993609	-1.722534439	3.18E-09	2.54E-08	Up
NDRG4	-2.3852868	7.955701009	6.32E-16	1.14E-14	Down
RFX5-AS1	2.795615146	2.110655368	2.77E-11	2.96E-10	Up
AL357055.2	2.795466424	-2.226264018	4.80E-07	2.78E-06	Up
ADAMTS1.	2.795136501	3.889423412	0.000377168	0.001231471	Up
HK2P1	2.794489366	-2.053947231	0.000273295	0.000924807	Up
NACA4P	2.794114616	-0.279603535	2.42E-12	2.94E-11	Up
FDPSP5	2.791851322	-2.726953402	9.02E-06	4.15E-05	Up
MAB21L3	-2.388215752	-3.513449005	0.000100213	0.000373018	Down
CD93	2.788940533	6.222383857	5.94E-07	3.39E-06	Up
LINC02381	2.788677606	3.963448419	2.59E-07	1.57E-06	Up
ANTXRL	-2.390201617	-3.255638369	9.00E-05	0.000338583	Down
FAM83D	2.78841453	3.438046724	7.51E-06	3.50E-05	Up
BIN1	-2.390300439	7.065722791	2.69E-22	9.24E-21	Down
KLC2	-2.390306603	5.518355579	1.07E-39	2.01E-37	Down
KIF11	2.787650453	4.268716131	2.03E-08	1.45E-07	Up
PRELID1P	2.787545277	-0.781363218	5.64E-12	6.58E-11	Up
ALG1L15P	2.78721681	-2.253773711	5.43E-07	3.12E-06	Up
AC079331.2	2.787074593	-1.477811815	6.13E-05	0.000238643	Up
AC080013.1	-2.390762657	0.146702672	1.29E-13	1.80E-12	Down
UBBP1	2.786574202	0.909733432	1.85E-13	2.54E-12	Up
NRNPA1P	2.786396091	2.981515602	2.20E-21	6.83E-20	Up
FDPSP4	-2.392405106	-3.199782004	3.99E-10	3.62E-09	Down
SMU1P1	2.785289943	-1.142979624	1.80E-10	1.72E-09	Up
MIR155HG	2.784787149	1.231698563	4.99E-05	0.000197588	Up
MMP25	2.783977703	2.620797606	4.31E-06	2.11E-05	Up
MADD	-2.392526803	6.114016287	3.91E-42	9.02E-40	Down
MMP17	-2.393113873	3.664969651	1.16E-07	7.41E-07	Down
MAP6D1	-2.394052316	4.69223728	8.76E-14	1.25E-12	Down
AC011912.1	-2.395250406	-1.13549061	2.23E-15	3.74E-14	Down
DGCR5	-2.395252706	3.537347769	3.25E-13	4.37E-12	Down
AL033523.1	-2.399728944	-0.533389225	7.62E-07	4.27E-06	Down
MORF4L1P	2.783157945	-2.045593636	4.66E-07	2.71E-06	Up
AC012447.1	2.783051698	-0.523461679	2.08E-07	1.28E-06	Up

AC110749.1	2.781416239	-2.229924731	1.95E-06	1.02E-05	Up
AC005920.4	-2.401360451	-3.153536732	9.89E-08	6.38E-07	Down
STRC	-2.401890196	-2.219412059	1.28E-08	9.42E-08	Down
AC006026.1	2.780922184	-2.746506138	5.58E-05	0.000218865	Up
LGALS9C	2.780374205	-1.887562701	0.00060378	0.001880385	Up
NT5DC2	2.778672305	6.835534593	6.19E-11	6.32E-10	Up
RPL21P131	2.778217522	-2.234662553	3.03E-06	1.53E-05	Up
AL158212.3	-2.402199703	2.757503317	2.73E-29	1.76E-27	Down
PPIAP33	2.777189946	-2.734533726	4.45E-05	0.000177869	Up
SLC2A4	-2.402225437	0.43681053	1.09E-13	1.53E-12	Down
PLEKHG2	2.777014621	5.869324218	2.06E-11	2.23E-10	Up
AL008627.1	2.776988642	-2.744325964	6.23E-05	0.000242186	Up
FTH1P8	2.776007005	3.074075654	2.66E-11	2.85E-10	Up
NPM1P18	2.774426212	-2.744394399	5.31E-05	0.000209177	Up
CDHR2	-2.402531017	0.836254103	8.49E-11	8.47E-10	Down
AL158211.1	-2.403678922	-2.041399417	2.12E-12	2.59E-11	Down
HNRNPA1P	2.773666302	-2.752017439	1.76E-05	7.63E-05	Up
CTNNBL1P	2.773497079	-2.257918463	3.11E-06	1.56E-05	Up
AC007160.1	2.772665686	-2.063024978	1.72E-06	9.05E-06	Up
TMEM37	2.770854006	3.157947771	5.81E-06	2.77E-05	Up
RBM47	2.770632395	3.959330072	1.60E-06	8.47E-06	Up
MYOM2	-2.403780567	3.65921681	4.48E-23	1.64E-21	Down
WDR45P1	2.770134284	-0.080236943	2.08E-06	1.08E-05	Up
MRC2	2.770092419	7.638486237	2.11E-06	1.09E-05	Up
SHCBP1	2.767043321	3.729154241	6.84E-11	6.95E-10	Up
LINC0128[illegible]	-2.404792226	-3.352749699	8.55E-07	4.74E-06	Down
LINC0287[illegible]	2.766411587	-0.378877435	2.69E-05	0.000112959	Up
UBBP2	2.765096718	-1.471600926	7.55E-09	5.72E-08	Up
AC021683.[illegible]	-2.405093251	-1.422377321	1.69E-05	7.36E-05	Down
AC007182.[illegible]	2.763833292	-0.751150278	9.15E-08	5.93E-07	Up
AC092809.[illegible]	2.763251678	-0.621531935	5.62E-14	8.15E-13	Up
HLA-DRB[illegible]	2.761185605	4.167318223	0.000967262	0.002869126	Up
STEAP2	-2.405478214	3.358476662	3.82E-08	2.62E-07	Down
AC023794.[illegible]	2.760252958	-1.41162839	0.000122562	0.000446744	Up
LY6E-DT	-2.406386066	0.443613862	4.37E-10	3.94E-09	Down
CELF6	-2.406417426	-2.855411757	1.57E-07	9.85E-07	Down
NPAS1	-2.406669251	1.914346648	1.66E-11	1.83E-10	Down
LAMA5-AS	2.758986017	-0.038602702	0.0001525	0.000544705	Up
SLC40A1	2.757482657	6.423986919	2.75E-06	1.40E-05	Up
SLC43A3	2.756655704	5.111838026	8.76E-13	1.12E-11	Up

AC004584.:	-2.407106612	-2.853592305	1.42E-07	8.96E-07	Down
AL109810.1	2.755957971	-3.069596685	0.000405645	0.001314173	Up
AC246787.:	2.754809789	1.833009977	2.41E-09	1.97E-08	Up
FSTL1	2.754078397	8.107263089	9.81E-11	9.73E-10	Up
VAX1	-2.407308843	0.054783645	0.000739545	0.002253754	Down
NRNPA1P	2.752639073	-3.070056869	0.000149336	0.000534729	Up
USP30-AS1	2.752395402	-0.899751365	0.000815391	0.002462495	Up
MAPRE1P:	2.751437824	-2.269518091	2.58E-07	1.56E-06	Up
PSMB9	2.75033662	5.698680565	1.49E-06	7.95E-06	Up
AC092919.:	2.748256162	-1.818128729	0.000358184	0.001176285	Up
PPP3CA	-2.409412328	6.332465769	7.27E-55	6.35E-52	Down
FGF12	-2.410033313	4.789220982	8.57E-08	5.58E-07	Down
GRK3	-2.410286375	4.214653431	5.01E-29	3.15E-27	Down
AC007785.:	-2.413373879	-2.426590605	2.61E-09	2.12E-08	Down
HLA-DQB:	2.746023714	6.55524679	0.000254555	0.000867402	Up
QDPR	-2.413863891	6.889062704	9.92E-21	2.87E-19	Down
GAPDHP2.	2.744608813	-2.272622615	3.67E-05	0.000149383	Up
RPL15P21	2.744364337	-3.09992532	0.000879976	0.002635445	Up
TDRD9	-2.415305015	0.659571292	8.42E-09	6.34E-08	Down
DENND2I	2.743695229	3.030834589	1.55E-06	8.26E-06	Up
PHBP3	2.743108138	-2.267881639	3.94E-07	2.32E-06	Up
TAC1	-2.419850982	2.642751299	0.000556798	0.001749192	Down
AC005790.:	2.742980872	-1.761789392	0.000284883	0.000959177	Up
RPL5P29	2.742390658	-1.502331253	6.56E-07	3.72E-06	Up
PGF	2.742385877	4.830377239	5.50E-05	0.000216206	Up
AC104763.:	2.74105772	-2.769118727	5.73E-05	0.000224522	Up
FAM126B	-2.420373525	3.923879512	7.10E-70	5.11E-66	Down
PRR11	2.740641494	4.184992219	2.16E-14	3.27E-13	Up
AC010336.:	-2.421125896	-2.69081729	9.27E-14	1.32E-12	Down
PLVAP	2.739166499	5.53162136	1.57E-05	6.88E-05	Up
WASF1	-2.422009623	5.608053806	9.80E-14	1.39E-12	Down
NHLRC1	-2.422661386	0.33903432	1.78E-11	1.95E-10	Down
S100A6	2.737014064	9.079986568	3.78E-06	1.87E-05	Up
ERICH5	-2.423249763	-2.441372812	4.92E-07	2.85E-06	Down
IL24	2.734761987	-0.357588379	0.000310512	0.001034946	Up
CENPE	2.733966978	3.374496571	2.19E-07	1.34E-06	Up
FBXO27	-2.423557027	2.601360695	1.21E-12	1.52E-11	Down
NAT1	2.731442667	1.655464905	1.16E-07	7.42E-07	Up
ZC3H11B	2.731037266	-1.300358339	2.79E-05	0.000116723	Up
NRNPA1P	2.73041456	-2.505890651	9.26E-06	4.25E-05	Up

LRRC8B	-2.424238757	5.035693507	1.32E-26	6.70E-25	Down
KIF17	-2.424795187	2.544779592	1.94E-17	4.08E-16	Down
AP001889.1	2.730358498	-3.095221021	0.000132837	0.000480367	Up
PTPN22	2.729749436	1.121702698	1.51E-05	6.67E-05	Up
LRP11	-2.42511988	4.485886055	7.28E-39	1.22E-36	Down
PPP1R3F	-2.427380487	4.142912048	4.91E-31	3.79E-29	Down
DUX4L27	-2.429450951	-3.069417623	9.83E-05	0.000366598	Down
RPL13AP20	2.728725846	1.200390721	5.34E-12	6.24E-11	Up
FAM174B	-2.431308964	2.673350473	1.38E-28	8.30E-27	Down
ANXA5	2.726847036	9.402152278	2.01E-10	1.91E-09	Up
SLC13A4	-2.432241821	1.543575534	1.23E-11	1.38E-10	Down
ATP2B1-AS	-2.433402677	2.157851962	1.03E-21	3.30E-20	Down
CDH13	-2.433413826	4.963783814	1.31E-07	8.29E-07	Down
BTF3P7	2.725982297	-2.073719739	1.41E-07	8.89E-07	Up
KRTAP5-7	-2.437375104	-3.037614106	4.98E-09	3.88E-08	Down
BCL2L12	2.725523183	3.309550962	1.64E-09	1.37E-08	Up
LINC0136(	2.723062268	-2.128970932	0.000292933	0.000982148	Up
AC107983.1	2.721203735	0.148362606	1.40E-10	1.36E-09	Up
RPL13AP25	2.71925293	3.111060003	3.14E-10	2.89E-09	Up
AC006483.1	2.717199005	-2.290413926	0.000169975	0.000600645	Up
PCDH20	-2.438276053	-2.259658381	2.94E-05	0.000122102	Down
AC110619.1	-2.438557372	-0.24571129	3.46E-10	3.17E-09	Down
CERKL	2.717142795	3.296358375	3.57E-06	1.77E-05	Up
FABP3	-2.439406762	4.238739023	7.36E-15	1.17E-13	Down
ADAMTS6	2.717100298	2.745838724	3.59E-07	2.13E-06	Up
TOMM20P	2.716962736	-3.098876218	0.000705821	0.002161269	Up
HNRNPA1P	2.715201511	-0.635541491	2.22E-10	2.09E-09	Up
AC002091.1	2.715133109	-1.243311123	0.000121421	0.000443145	Up
TPI1P1	2.715036176	3.349152805	5.25E-11	5.42E-10	Up
AL031283.1	-2.439496403	-1.411372787	4.27E-09	3.36E-08	Down
LRRC38	-2.441246821	-1.773282061	2.55E-05	0.000107479	Down
HCN4	-2.441389599	0.833055498	1.00E-06	5.49E-06	Down
AC007849.2	2.714787848	-2.772579939	3.04E-05	0.000126171	Up
RPSAP11	2.714543811	-1.035045142	4.86E-08	3.28E-07	Up
ARMC10P1	2.714464392	-1.215534874	8.14E-09	6.14E-08	Up
TAMALIN	-2.441959915	3.743608198	1.06E-20	3.05E-19	Down
PKLR	-2.442197843	-2.883010245	4.92E-09	3.84E-08	Down
MROCKI	2.711956185	3.268779579	0.000611124	0.001899903	Up
SEPTIN3	-2.443045258	7.184709522	1.50E-12	1.85E-11	Down
LIMA1	2.710876323	7.226789611	1.57E-09	1.31E-08	Up

EEF1DP1	2.710128902	0.88417362	9.63E-15	1.51E-13	Up
3X005019.1	-2.444115414	-3.303531783	0.000156361	0.000556976	Down
MTATP8P1	2.707551057	1.272991391	4.85E-05	0.000192547	Up
AGBL2	2.706613316	1.111510291	9.56E-05	0.000357474	Up
F2RL1	2.705807412	3.352336284	0.000831794	0.002507828	Up
SPIN2A	-2.445646581	-3.014807988	1.08E-08	8.01E-08	Down
H2AC20	2.705656609	-0.099071107	0.000730087	0.002228938	Up
AP000721.2	-2.445649032	-2.232985301	3.16E-08	2.20E-07	Down
AC110285.1	-2.448135552	1.141259028	4.17E-06	2.04E-05	Down
AC022001.3	-2.448732409	-3.402124762	6.97E-08	4.61E-07	Down
ECSCR	2.704804436	2.191308022	5.39E-07	3.10E-06	Up
AC005000.1	2.704616994	0.042365839	1.51E-15	2.59E-14	Up
PIP5K1B	-2.448902515	2.40760035	3.84E-12	4.55E-11	Down
PNMA8A	-2.449167636	6.292628048	4.37E-39	7.55E-37	Down
F7	-2.449268358	-1.333028362	5.37E-05	0.000211615	Down
WNT7B	-2.449373896	2.787029587	1.42E-05	6.31E-05	Down
BE2E1-AS	2.703515255	-0.125775497	2.02E-07	1.24E-06	Up
SF3A3P2	2.702829486	-1.945936301	9.13E-08	5.92E-07	Up
RNPS1P1	2.700976529	0.714386414	9.52E-17	1.86E-15	Up
ELAVL4	-2.450848914	4.294186246	1.40E-06	7.49E-06	Down
AC007016.1	2.700505989	-1.967921318	8.10E-06	3.76E-05	Up
UTS2R	-2.450866928	-2.567424895	0.000423715	0.001366109	Down
FBXL2	-2.451030943	3.454514581	7.08E-11	7.16E-10	Down
ARL5C	-2.451071982	-3.332356753	4.02E-08	2.75E-07	Down
AKAP6	-2.452881151	5.657956661	1.05E-20	3.03E-19	Down
ADAMTS7	2.699930196	3.436909528	0.000306245	0.001022497	Up
PELI3	-2.454090665	3.712650414	1.13E-29	7.66E-28	Down
AC073367.1	-2.456209605	-2.730307277	6.08E-11	6.21E-10	Down
EIF4EP2	2.699715816	0.387947848	6.58E-13	8.50E-12	Up
MAPRE3	-2.456355006	5.98091124	8.39E-36	1.03E-33	Down
BAAT	-2.458968558	-1.448082153	1.44E-12	1.79E-11	Down
FCGR2C	2.699178178	2.029839637	0.000402346	0.001304513	Up
ARNILA	-2.459051541	-1.249104867	3.05E-17	6.26E-16	Down
NSA2P3	2.698039752	-2.804079348	0.000115619	0.000424045	Up
GTF2IP7	-2.460780826	-3.173264528	2.51E-07	1.52E-06	Down
AC026992.2	-2.463282597	-3.315568695	3.84E-08	2.64E-07	Down
TIMM8BP2	2.697501599	-1.306994764	1.78E-06	9.38E-06	Up
NID2	2.693603202	4.168076201	4.17E-05	0.000167686	Up
CHTF8P1	2.691373055	-3.119560369	0.00044688	0.001433739	Up
DYNLT1	2.691271605	6.778173126	9.05E-13	1.15E-11	Up

ATP9A	-2.466294748	7.2839646	3.10E-28	1.80E-26	Down
USP8P1	2.688907571	-2.819838864	0.000230674	0.000792776	Up
PP1R26-AS	-2.467660983	-0.273557083	7.92E-19	1.90E-17	Down
AC116533.1	2.688454641	3.543833319	4.83E-11	5.01E-10	Up
CABYR	-2.469028959	1.361025286	8.82E-13	1.12E-11	Down
RPL10P3	2.688215504	-0.699367313	1.88E-08	1.35E-07	Up
AL135999.2	-2.469908023	-2.315014621	6.66E-09	5.10E-08	Down
IQSEC1	-2.47148546	6.31445596	9.76E-35	1.09E-32	Down
RPL3P1	2.686711018	-1.533568022	3.29E-08	2.28E-07	Up
AC021127.1	-2.471562935	-3.206274811	1.96E-05	8.43E-05	Down
KCNIP4	-2.471759269	3.920102203	3.41E-08	2.36E-07	Down
DNAH11	2.686578372	1.816703746	0.00080492	0.002432912	Up
FUNDC2P2	2.684689133	-2.347308574	9.80E-06	4.47E-05	Up
PTGES3P2	-2.47396167	-1.209279897	1.15E-08	8.50E-08	Down
PLA2G4D	-2.47409449	-3.005025601	5.32E-07	3.06E-06	Down
FAM13C	-2.474492829	3.460917366	5.45E-24	2.20E-22	Down
ECM2	2.682619096	5.006680839	8.39E-06	3.88E-05	Up
BCRP2	-2.474803532	-2.944231161	2.96E-12	3.56E-11	Down
SEPHS1P4	2.681320595	-2.136621624	1.35E-06	7.28E-06	Up
MOAP1	-2.474872821	6.021723278	4.29E-40	8.31E-38	Down
HRK	-2.476901901	2.762416673	2.30E-10	2.16E-09	Down
LINC02285	2.679351043	-0.408002335	0.000287019	0.000965126	Up
COX7CP1	2.678927678	-0.202664242	8.77E-11	8.75E-10	Up
PLPP2	-2.478758573	3.110959142	1.18E-08	8.67E-08	Down
AC015849.0	2.678647404	-2.804965985	0.000191604	0.000669767	Up
BEST2	2.678011883	-2.549369006	0.000497435	0.001577664	Up
ETF1P2	2.677252249	-2.547805165	2.72E-06	1.38E-05	Up
AC159540.2	2.676303843	-2.830098644	0.000383304	0.001249381	Up
HSPA8P7	2.674450223	-1.143137011	2.37E-09	1.94E-08	Up
AC122718.1	2.674227593	-1.837560139	2.04E-05	8.74E-05	Up
PTPRD	-2.481120176	5.69811829	6.08E-16	1.09E-14	Down
AC126615.1	2.673770662	-1.528385464	3.51E-07	2.08E-06	Up
LINGO1	-2.48246039	6.382844029	1.81E-10	1.73E-09	Down
TK1	2.673596692	4.451685784	3.34E-08	2.31E-07	Up
FTH1P5	2.672915362	0.93863004	3.24E-09	2.59E-08	Up
RPL5P23	2.670829556	-0.154509522	2.40E-09	1.96E-08	Up
AC026316.1	2.670134824	-2.120463315	5.58E-06	2.67E-05	Up
ACSL6	-2.482611333	5.000018092	2.14E-12	2.61E-11	Down
NRNPA1P	2.669679869	-2.338965232	1.00E-06	5.50E-06	Up
AC233280.1	2.668017243	-0.542199085	0.000102964	0.000382467	Up

PRRX1	2.667503904	6.692396903	7.12E-10	6.25E-09	Up
FUT1	-2.482783884	0.444581217	1.77E-23	6.69E-22	Down
ZNF90	2.666604431	1.956975456	2.54E-08	1.80E-07	Up
GALNT14	-2.484681909	1.791795205	2.70E-09	2.18E-08	Down
AC000089.1	2.665843256	0.810931771	1.37E-11	1.51E-10	Up
CTSC	2.664929424	6.619270087	1.83E-05	7.92E-05	Up
RPS3AP38	-2.48699387	-2.822029841	8.37E-12	9.52E-11	Down
NRNPA1P	2.663837291	-2.830123243	9.12E-05	0.000342509	Up
AC011979.1	2.662541365	-0.816632207	4.25E-07	2.49E-06	Up
GDI2P2	2.659535105	-1.436077019	8.24E-08	5.38E-07	Up
RIPOR2	-2.488489419	3.852714906	1.87E-13	2.57E-12	Down
GNG5	2.658889046	6.666067497	1.90E-14	2.89E-13	Up
LINC0268	-2.489578529	-1.989691258	1.41E-07	8.92E-07	Down
ALDOAP2	2.658882819	-0.520372792	4.01E-10	3.64E-09	Up
CALM3	-2.489845061	8.930683428	2.98E-48	1.23E-45	Down
PDE4C	-2.492757626	1.595208725	8.51E-08	5.55E-07	Down
LZTS3	-2.492985394	5.883168786	5.82E-24	2.35E-22	Down
AC015912.	2.658235321	-0.992295489	4.90E-05	0.000194472	Up
ARL6IP1P	2.657144367	-2.343345844	1.71E-06	9.00E-06	Up
CDCA4	2.657048685	3.825575278	2.67E-09	2.16E-08	Up
CACNA1A	-2.493013746	4.340162593	3.90E-10	3.55E-09	Down
TUBAP	2.656762085	-2.818760566	0.000249407	0.00085216	Up
AC016596.	2.656652041	0.599121144	1.96E-09	1.62E-08	Up
AC103770.	-2.496212121	-2.288474138	2.98E-05	0.00012389	Down
GNAS-AS1	2.655410628	-0.228042954	4.66E-07	2.71E-06	Up
LINC0201	-2.502911961	-1.129960392	8.11E-13	1.04E-11	Down
NRIP2	-2.503371951	2.154532124	1.04E-25	4.87E-24	Down
FCMR	2.654971879	1.704568545	1.20E-06	6.49E-06	Up
PCAT1	2.654189795	-2.576441116	0.000762794	0.002317254	Up
RASL10A	-2.504221683	2.682705632	3.98E-11	4.18E-10	Down
BARX2	-2.505919882	-1.364384993	6.81E-07	3.85E-06	Down
PARPBP	2.652150055	2.768843502	4.52E-10	4.07E-09	Up
AL121769.1	2.652048026	-2.574953949	6.10E-05	0.000237463	Up
POU3F1	-2.506711923	1.209119584	1.51E-07	9.51E-07	Down
WBP1P2	2.65154202	-1.167138834	8.45E-10	7.33E-09	Up
BRCA2	2.648041049	2.516389481	1.61E-08	1.17E-07	Up
RAB13	2.647068909	6.797511936	1.03E-12	1.30E-11	Up
H3P43	2.646903772	-2.340850075	2.17E-06	1.12E-05	Up
PPIAP30	2.646409768	-2.827473231	0.000124672	0.000453686	Up
IFI30	2.645331181	0.823197679	1.96E-05	8.43E-05	Up

HGAP31-/	2.644458516	-0.662008033	1.31E-07	8.33E-07	Up
BEND3P3	-2.50705658	-0.53818588	9.10E-16	1.60E-14	Down
GNG5P2	2.644268952	-1.180278744	6.66E-09	5.10E-08	Up
CAP2	-2.510087299	5.495397303	1.64E-28	9.79E-27	Down
LILRB2	2.644204097	2.952047941	0.000432626	0.00139157	Up
WWTR1	2.644001542	7.342167428	2.43E-06	1.24E-05	Up
\C093117.:	2.643537761	-0.701891792	0.000262132	0.000891114	Up
LINC0247:	-2.510412267	-1.443273471	5.67E-09	4.38E-08	Down
OR13J1	-2.510784518	-2.802548511	6.51E-09	4.99E-08	Down
RPSAP74	2.642419408	-3.155682783	0.000834012	0.002513727	Up
\C091607.:	2.642399421	-3.141445001	0.000711914	0.002178765	Up
LRR1	2.641315245	3.33553327	1.59E-12	1.96E-11	Up
SETP20	2.640237859	-2.01413729	5.85E-06	2.79E-05	Up
PLCB1	-2.511816959	4.860804267	1.73E-14	2.65E-13	Down
SLC6A1	-2.511927907	6.476856278	1.13E-13	1.60E-12	Down
RPL23AP5'	2.639100458	-1.370324437	2.18E-08	1.55E-07	Up
TLL2	-2.512116899	1.002101042	5.52E-09	4.27E-08	Down
\C013470.:	2.637139889	-2.364544546	3.05E-05	0.000126402	Up
\RHGEF3'	2.636799958	2.07558864	2.35E-07	1.43E-06	Up
\P001267.2	-2.513703959	-1.905594119	7.26E-15	1.15E-13	Down
CADM3	-2.514954023	6.871297522	4.19E-07	2.46E-06	Down
PXDN	2.636636489	6.547190384	0.000138189	0.00049797	Up
C10orf90	-2.517506976	2.397734427	9.23E-12	1.04E-10	Down
\C010533.:	2.636491029	-2.201980699	0.000215784	0.000745695	Up
CDC25A	2.63601475	3.468476584	1.68E-06	8.87E-06	Up
\P005264.:	-2.518123092	-3.564896687	5.17E-07	2.98E-06	Down
\P002381.1	2.633397984	-1.863355952	1.54E-06	8.20E-06	Up
KLHL38	-2.51890766	-2.156113979	6.71E-11	6.82E-10	Down
ASB2	-2.519225743	0.440952655	1.28E-10	1.25E-09	Down
GARNL3	-2.519447463	3.49019347	1.87E-33	1.83E-31	Down
PTPN7	2.632864505	2.826405836	4.03E-07	2.37E-06	Up
CLDN11	-2.520375832	6.310868316	6.85E-07	3.87E-06	Down
\C092834.:	-2.520668073	-3.706657183	2.97E-05	0.000123436	Down
NIPA1	-2.521998346	5.096927803	2.61E-35	3.02E-33	Down
CD276	2.63115029	7.119647725	1.84E-12	2.25E-11	Up
PGK1P2	2.630674945	0.379617381	2.22E-09	1.83E-08	Up
NSUN7	2.630557205	2.204975081	4.86E-05	0.000193003	Up
\C087257.:	-2.522509943	-3.526233856	6.48E-05	0.000251377	Down
LMF1-AS1	-2.524561476	-1.776287791	9.15E-09	6.85E-08	Down
GJB1	-2.526518409	4.062524753	1.29E-05	5.76E-05	Down

AL683842.1	-2.526943445	-3.511844681	1.60E-06	8.49E-06	Down
JPH1	-2.52904488	2.260201387	1.02E-07	6.54E-07	Down
ZBTB42	2.63004375	3.400615252	7.29E-07	4.10E-06	Up
NTN1	2.629360701	6.670134693	3.69E-07	2.19E-06	Up
HLA-DMA	2.628978449	6.424989263	1.01E-06	5.54E-06	Up
C22orf24	-2.529297951	-1.717480745	4.41E-19	1.09E-17	Down
NPM1P40	2.62828414	-3.155162929	0.000899804	0.002688403	Up
SYDE1	2.628223328	5.460540675	7.29E-16	1.30E-14	Up
B3GALT2	-2.530534852	3.143647248	1.00E-09	8.65E-09	Down
SLC16A8	-2.531208245	0.077737639	2.76E-13	3.72E-12	Down
PGR	-2.531303985	-1.324586278	3.03E-08	2.12E-07	Down
MIR600HG	-2.5323333	3.011629804	9.76E-15	1.53E-13	Down
RCC1	2.628159125	5.114685885	9.31E-16	1.63E-14	Up
HMOX1	2.6279596	7.460929629	0.000196353	0.000684952	Up
LINC0192[illegible]	-2.532586484	-3.329942338	5.18E-08	3.48E-07	Down
TAC3	-2.535179366	1.876463491	3.68E-05	0.0001497	Down
HSPE1P7	2.627843186	-2.580625578	3.66E-05	0.000148841	Up
TMC5	-2.537077878	-1.463056873	2.93E-10	2.72E-09	Down
LRRC25	2.627511596	3.776114458	9.87E-06	4.50E-05	Up
AC005912.[illegible]	2.627030625	3.259202696	6.25E-11	6.37E-10	Up
AL391422.[illegible]	2.62619199	3.452331615	4.30E-09	3.39E-08	Up
TNNT1	-2.537344919	1.290083984	3.41E-06	1.70E-05	Down
HINT1P1	2.626164938	-1.850006003	8.68E-07	4.81E-06	Up
P2RY8	2.62578361	1.551265852	0.000164846	0.000583953	Up
MEM92-A[illegible]	2.625151696	-1.718676619	0.000615124	0.001910962	Up
SPRY1	2.623787261	5.645688635	1.08E-05	4.89E-05	Up
AC244034.[illegible]	2.621524174	-1.186210941	7.70E-08	5.05E-07	Up
RPS4XP2	2.62120898	-0.22385452	3.88E-09	3.07E-08	Up
AL132655.[illegible]	2.6198138	-2.006020614	0.000968911	0.002873131	Up
COX5AP2	2.618853586	-3.161068009	0.000611168	0.001899903	Up
RPL23AP3	2.618419958	-2.044470863	4.32E-06	2.11E-05	Up
RDH5	2.617439379	1.979951619	1.32E-05	5.89E-05	Up
AP001486.[illegible]	-2.539821382	2.874472216	6.23E-26	2.98E-24	Down
PIFO	2.616921097	5.080319803	0.000748707	0.002278782	Up
AC099340.[illegible]	2.616514148	-2.18081146	8.25E-06	3.83E-05	Up
CLCF1	2.616218508	3.28544941	0.000578228	0.001809805	Up
RNF43	-2.542269465	0.320117952	8.28E-07	4.61E-06	Down
RPL39	2.615977311	7.042623165	1.14E-07	7.27E-07	Up
GPR12	-2.542805092	2.312724186	1.76E-06	9.25E-06	Down
LYPLA1P[illegible]	2.615412805	-1.209197536	9.59E-07	5.27E-06	Up

AJM1	-2.545574157	3.568059303	2.69E-25	1.22E-23	Down
KCNQ2	-2.545762133	6.817821357	3.12E-08	2.17E-07	Down
ACBD7	-2.546045472	2.333490947	5.95E-10	5.27E-09	Down
AC104083.1	-2.547779266	2.559601055	1.44E-14	2.23E-13	Down
YWHAZP5	-2.548113548	-1.206063469	2.93E-41	6.25E-39	Down
AC026471.0	2.613487106	0.384049585	4.26E-08	2.91E-07	Up
RPL10P7	2.612503428	-2.596886091	8.39E-05	0.000317711	Up
CLMN	-2.548282431	4.014439833	1.39E-23	5.38E-22	Down
SNRPCP2	2.612030552	-2.383071986	5.52E-06	2.64E-05	Up
AL358334.2	2.611740972	0.991969623	2.84E-05	0.000118397	Up
RAB32	2.611612764	4.854561646	2.14E-06	1.11E-05	Up
SYT2	-2.54957203	0.458568982	5.61E-09	4.33E-08	Down
AC004129.1	2.610839218	-2.851324001	0.000177546	0.000625175	Up
GNAI1	-2.549860194	5.007583929	1.32E-13	1.84E-12	Down
MYO1G	2.610206113	3.150537529	0.000108949	0.000401954	Up
GDF10	-2.550670426	0.612056115	3.60E-05	0.000146706	Down
RBBP4P1	2.609986473	0.497598179	5.36E-13	7.00E-12	Up
PPFIA2-AS	-2.5509941	-2.063636521	1.65E-10	1.58E-09	Down
SPDEF	-2.552699168	-1.592825373	5.96E-10	5.28E-09	Down
TACR2	-2.552774356	-0.2262708	3.50E-22	1.19E-20	Down
CPEB1	-2.55359387	3.269696569	5.02E-23	1.83E-21	Down
AC087072.1	2.609445558	-2.842199139	7.72E-05	0.000294411	Up
AL353752.1	2.609439147	-2.865367458	0.000808539	0.00244334	Up
ETNPPL	-2.557927457	4.695530893	1.18E-05	5.31E-05	Down
FAM124A	-2.558275172	3.585301629	1.21E-17	2.61E-16	Down
CHEK2	2.608404262	3.130328957	3.35E-11	3.54E-10	Up
AJ009632.2	-2.558651856	-3.506329587	5.22E-08	3.51E-07	Down
CFC1	-2.558704736	-3.280271194	6.80E-05	0.000262581	Down
PPIAP55	2.608136348	-1.616013723	7.66E-07	4.29E-06	Up
TLCD4	-2.561026607	3.270509943	1.89E-13	2.59E-12	Down
AC111182.1	2.607793417	-1.519012194	0.000315035	0.001047839	Up
ADARB1	-2.561955553	4.026457384	8.00E-29	4.94E-27	Down
RPL21P120	2.606884894	-1.409588046	3.12E-06	1.57E-05	Up
GAS1	2.606408701	5.488485584	0.000514614	0.001627671	Up
RPS27AP11	2.605618574	0.053416284	5.31E-09	4.12E-08	Up
RGS1	2.605117037	6.975315012	0.000515147	0.001628746	Up
C1QB	2.604469972	8.912893693	3.98E-05	0.000160619	Up
NEXMIF	-2.563465467	2.957860342	3.16E-07	1.89E-06	Down
POU6F2	-2.563691129	0.127502356	1.12E-05	5.06E-05	Down
YWHAEP5	2.603305075	0.760673463	6.96E-13	8.97E-12	Up

RBP1	2.602086269	7.041498334	6.00E-05	0.000234129	Up
ATP5PDP2	2.601499618	-2.199567559	3.07E-06	1.55E-05	Up
KMT5AP1	2.600118487	-1.891821881	4.38E-08	2.98E-07	Up
XRCC6P4	2.598044944	-2.381267649	1.30E-06	6.99E-06	Up
GAS7	-2.564208909	7.511339852	8.76E-16	1.54E-14	Down
AF064860.2	2.596603774	-0.898985221	0.000383184	0.001249271	Up
AL135818.2	-2.564915643	-3.239609439	1.82E-06	9.54E-06	Down
PLIN4	-2.56511699	1.318343053	2.32E-09	1.90E-08	Down
ANTXR2	2.594595171	4.818259317	6.06E-08	4.03E-07	Up
MUC1	2.59450623	3.820505895	6.41E-06	3.03E-05	Up
LMTK2	-2.565246995	5.078703926	1.08E-37	1.57E-35	Down
AC079905.1	2.594063178	-2.594294879	0.000962402	0.002855296	Up
MTNR1A	-2.56694701	-3.775691096	6.60E-05	0.000255337	Down
LINC01574	-2.567804103	-2.387595923	1.13E-06	6.14E-06	Down
PLEKHA1	-2.567857345	4.510352883	1.11E-53	7.45E-51	Down
AC091057.1	2.593670849	2.376305193	2.92E-07	1.75E-06	Up
TREM2	2.593665023	6.61800636	1.55E-05	6.82E-05	Up
RPEL1	2.593663637	-2.206687189	4.40E-07	2.57E-06	Up
TH	-2.56895737	-1.820653218	3.37E-05	0.000138296	Down
SHISA9	-2.569713848	3.837810982	3.32E-08	2.30E-07	Down
ADRB1	-2.570551863	1.886794205	1.81E-13	2.49E-12	Down
AC023024.2	-2.570588142	-0.369974922	3.71E-09	2.95E-08	Down
COL5A2	2.593396914	7.069959108	0.00025159	0.00085811	Up
RPS8P3	2.59187604	-2.869293808	0.000431565	0.001388312	Up
BRSK1	-2.570625413	5.747377719	4.43E-26	2.14E-24	Down
PPIAP16	2.590549602	-2.031392894	4.58E-06	2.22E-05	Up
PABPC4L	2.590419869	0.743946433	6.11E-05	0.000237912	Up
SCIMP	2.586540138	2.955326375	4.62E-06	2.24E-05	Up
LMTK3	-2.571680245	4.905364511	8.62E-23	3.08E-21	Down
AL162391.1	-2.571785417	-2.864108552	8.55E-09	6.43E-08	Down
GINS1	2.586357563	4.707694704	6.57E-09	5.04E-08	Up
AP001542.1	-2.572427832	-3.415793209	0.000275192	0.000930678	Down
ACTG1P23	2.584952168	-0.701510361	3.71E-12	4.41E-11	Up
CERCAM	-2.572992954	6.404294217	1.61E-21	5.09E-20	Down
EDA2R	2.584825251	3.399877477	0.000484525	0.001540791	Up
PPIAP2	2.584715668	-1.757087551	1.02E-06	5.58E-06	Up
AC011825.1	2.584111528	-2.621023975	5.77E-05	0.000225641	Up
STOX2	-2.573150177	4.386808569	4.20E-22	1.40E-20	Down
CTSS	2.583132893	6.508235589	4.66E-05	0.000185382	Up
SGCD	-2.57479678	2.655754221	1.87E-06	9.79E-06	Down

TBX2	2.582620488	5.030787149	1.25E-06	6.74E-06	Up
KCNJ13	-2.576162961	-2.793228786	1.87E-05	8.08E-05	Down
AL050331.1	2.582554307	-0.970768068	1.18E-08	8.73E-08	Up
IL7	2.580448151	1.023496685	2.31E-05	9.83E-05	Up
SYT15	-2.57673735	-0.280059821	9.38E-13	1.19E-11	Down
C1QA	2.579142466	8.189634143	7.58E-05	0.000289769	Up
PPIAP40	2.578952568	-1.524589743	2.26E-07	1.38E-06	Up
RPS4XP6	2.578848726	0.066926258	3.05E-09	2.45E-08	Up
PPIAP19	2.576071528	-1.311213535	1.98E-08	1.42E-07	Up
RPL12P37	2.575213117	-2.869343262	0.000298316	0.000998686	Up
RAB38	2.57420156	0.52803999	0.000173937	0.000613591	Up
AC016546.1	2.574036962	-2.610644377	1.51E-05	6.68E-05	Up
EMILIN2	2.573661991	4.724775756	3.24E-05	0.000133336	Up
MS4A7	2.572604383	5.559309357	5.40E-05	0.000212478	Up
AL512844.1	2.572014488	-1.096758053	1.69E-08	1.22E-07	Up
RPL39P38	2.57135973	-1.540146849	7.03E-07	3.97E-06	Up
SLC13A5	-2.577954484	1.859706439	2.33E-05	9.88E-05	Down
TYROBP	2.570006361	6.873468203	1.59E-05	6.96E-05	Up
LINC01537	-2.578377566	-1.968063247	2.94E-12	3.54E-11	Down
SPRYD3	-2.578609314	6.34019861	9.21E-38	1.36E-35	Down
LINC00511	2.569219013	6.041628836	1.30E-07	8.26E-07	Up
HAS1	-2.580232763	-0.082441651	2.96E-05	0.000123146	Down
CSTF3-DT	2.567706826	-1.248857762	1.08E-05	4.89E-05	Up
MFSD1P1	-2.580257375	-2.869114601	1.37E-08	1.00E-07	Down
AP003721.1	-2.58087241	-1.276860853	2.46E-11	2.65E-10	Down
HECW2	-2.580905867	3.586366288	9.30E-22	3.01E-20	Down
AL365217.1	2.566716238	-2.223946937	0.000330853	0.001095014	Up
BTF3P8	2.565141421	-2.872898817	0.000107416	0.000397266	Up
AC138894.1	2.563186673	-2.660274731	0.000189676	0.000663588	Up
B2M	2.562651305	11.29459475	4.23E-08	2.88E-07	Up
AC022400.1	-2.581053619	-1.08007191	8.34E-19	2.00E-17	Down
CLDN7	2.56215745	0.767544486	2.74E-06	1.39E-05	Up
SPINT2	-2.581662233	4.365475775	1.58E-18	3.70E-17	Down
MTCO2P22	2.56159184	-0.274545246	5.52E-06	2.64E-05	Up
UPP2	-2.582049774	-0.186395468	1.59E-11	1.75E-10	Down
AC107032.1	2.560972496	-1.258479911	1.41E-07	8.89E-07	Up
HLA-A	2.560666998	10.15721063	5.28E-07	3.04E-06	Up
LYRM4-AS	2.559987854	0.604857044	6.01E-12	6.96E-11	Up
PHBP1	2.558436093	-3.197714335	0.000871325	0.002611982	Up
MIR7158	-2.583777344	-3.490014424	8.70E-05	0.000328496	Down

FO681492.1	-2.58407232	0.135346706	6.96E-10	6.11E-09	Down
MCM2	2.558397623	6.332399222	3.20E-08	2.22E-07	Up
RPL7P19	2.557885248	-1.651771112	1.35E-07	8.53E-07	Up
CYP2E1	-2.584547017	0.718266364	7.39E-12	8.45E-11	Down
ARL11	2.557444238	2.127870758	2.41E-05	0.000101909	Up
NPM1P19	2.557341727	-2.644929491	1.51E-05	6.64E-05	Up
HCG15	2.557049665	-0.149118414	9.50E-07	5.23E-06	Up
LMO7	-2.584638327	3.788360963	6.97E-29	4.34E-27	Down
MYPN	-2.584854809	-2.716565952	1.38E-09	1.17E-08	Down
AC018868.1	2.556437375	-1.024750723	3.65E-08	2.51E-07	Up
FCGR3A	2.556400578	7.646959701	5.03E-05	0.000198893	Up
TPRG1	2.555730876	0.773468678	1.62E-06	8.59E-06	Up
NMI	2.555610388	4.236940256	7.84E-09	5.94E-08	Up
FGF9	-2.586176664	1.095886284	1.33E-06	7.17E-06	Down
AC104958.2	2.554969586	-0.365731898	2.14E-06	1.11E-05	Up
AP000281.1	2.554863644	-2.644859074	0.000146459	0.000525209	Up
SMIM11A	2.554285677	-2.424970875	6.00E-06	2.85E-05	Up
TLL1	-2.587436477	-0.194394408	2.11E-06	1.09E-05	Down
AMER2	-2.587778445	5.833816629	3.95E-12	4.69E-11	Down
AP003393.1	-2.588169845	-1.045162265	7.43E-11	7.49E-10	Down
PPIAP39	2.553813008	-1.349967523	0.000467746	0.001493864	Up
IGFBP1	2.553766143	1.327345897	0.000634587	0.001965284	Up
PRKCQ	-2.588443205	1.897861692	2.92E-16	5.48E-15	Down
DHX9P1	2.546589555	-1.272281481	7.09E-09	5.40E-08	Up
LARGE1	-2.589857026	5.070685975	2.69E-27	1.45E-25	Down
NKRD18D	-2.590163161	-1.792790306	1.36E-07	8.58E-07	Down
CNP	-2.590364441	8.825371927	2.44E-22	8.39E-21	Down
SND1-IT1	-2.591191616	-2.900320916	6.89E-12	7.90E-11	Down
PKD2L1	-2.591290557	0.265582769	1.02E-07	6.54E-07	Down
GALNT6	-2.593609121	2.794204089	1.85E-14	2.81E-13	Down
VTI1BP1	2.546080984	-2.43509925	4.50E-06	2.19E-05	Up
ELOBP1	2.545937189	-2.221129071	3.01E-05	0.000124854	Up
GLDCP1	2.544876689	-0.303682357	1.56E-05	6.87E-05	Up
TRPV5	-2.59371056	-2.920439787	6.97E-10	6.11E-09	Down
PLBD1	2.544692353	3.599527281	4.57E-05	0.000182068	Up
B4GALT6	-2.593727109	3.353377828	7.59E-13	9.73E-12	Down
RPL34P33	2.543692972	-1.785112594	2.99E-06	1.51E-05	Up
C8orf88	2.543503598	1.775850182	3.46E-07	2.05E-06	Up
AC009005.1	2.543349327	0.86953229	7.52E-06	3.51E-05	Up
TRIM54	-2.594062406	-0.531736746	1.49E-08	1.09E-07	Down

FAM72D	2.543207004	-0.544163698	1.01E-07	6.51E-07	Up
SNAI3-AS1	-2.594600279	1.991792714	2.95E-44	8.32E-42	Down
AL358154.1	-2.595205529	-3.229405316	9.44E-07	5.20E-06	Down
ELENOWI	2.540454479	-2.644745466	0.000215699	0.000745581	Up
NPM1P5	2.540003842	-2.439268827	1.93E-05	8.34E-05	Up
MTCO1P12	-2.595341076	6.763152055	1.56E-09	1.31E-08	Down
AC079780.1	2.539672833	-1.95875059	1.25E-05	5.61E-05	Up
RPL12P8	2.538571923	-1.465055876	6.24E-07	3.55E-06	Up
PRDX1P1	2.536466572	-0.480567859	7.53E-11	7.59E-10	Up
CLEC2B	2.535360236	3.430430963	0.000842219	0.00253634	Up
SLC7A7	2.533627804	5.056182165	6.39E-07	3.63E-06	Up
AC025518.1	2.53327889	-2.458647629	0.00047767	0.001521511	Up
NCR3LG1	-2.596820382	1.222309985	4.04E-15	6.57E-14	Down
NAP1L5	-2.599155107	4.970636597	1.96E-43	5.00E-41	Down
ADCYAP1	-2.600739557	1.544077234	3.77E-07	2.23E-06	Down
LINC02828	2.532694039	-0.664659876	0.000166385	0.000588825	Up
PSMA6P1	2.532126034	0.275995746	5.33E-09	4.14E-08	Up
AC063944.1	-2.601480676	-1.882587355	1.43E-19	3.69E-18	Down
AC020663.2	-2.601981612	-2.795501087	6.76E-09	5.16E-08	Down
LAG3	2.531285502	0.928119505	8.64E-05	0.000326281	Up
SYT9	-2.602031637	1.965305652	1.58E-07	9.88E-07	Down
C5AR1	2.530965421	5.103503713	0.000264536	0.000897803	Up
TMEM191A	-2.602581418	-0.621140614	5.99E-17	1.20E-15	Down
RPH3A	-2.603004515	5.792529126	8.65E-06	3.99E-05	Down
AC108161.1	2.53080624	-1.808426844	1.61E-06	8.53E-06	Up
YWHAH	-2.606075267	7.970460917	2.64E-42	6.14E-40	Down
RPS4XP17	2.530581861	-1.092134974	4.43E-08	3.01E-07	Up
FHP1	2.530183919	-2.258587818	4.52E-07	2.63E-06	Up
RPL21P93	2.528665555	-1.016989008	7.87E-07	4.39E-06	Up
KL	-2.606550492	0.194214074	2.32E-15	3.89E-14	Down
AC004224.1	2.527157384	-2.461340707	0.000121525	0.000443469	Up
HLA-B	2.526352394	10.24071923	6.07E-06	2.88E-05	Up
NRXN1	-2.606802152	5.912135001	1.61E-11	1.77E-10	Down
CD180	2.525965111	3.029711549	2.56E-05	0.000107839	Up
HSPD1P12	2.525378371	-2.918619489	0.00016765	0.000592718	Up
C2CD4D	-2.608508185	-1.242187541	5.62E-11	5.78E-10	Down
OSMR	2.523221049	6.290563028	6.78E-05	0.000261525	Up
EVC2	2.523038833	2.376417832	0.000113421	0.000416797	Up
AC092384.1	-2.609029946	-2.772216242	2.20E-13	2.99E-12	Down
ZNF204P	-2.609038865	2.909306851	1.33E-17	2.85E-16	Down

KCNMB1	2.521815881	3.579664546	1.50E-05	6.61E-05	Up
HSPE1P8	2.521615632	-2.918373832	0.000398382	0.00129297	Up
KCNH6	-2.612607326	-1.812765945	9.32E-13	1.18E-11	Down
HLA-DPB1	2.521599215	7.814036955	1.16E-05	5.25E-05	Up
AC068535.1	2.521206666	1.275371189	0.000703204	0.002153711	Up
ATP5MDP	2.521007725	-1.486759741	9.04E-06	4.15E-05	Up
TP53	2.520686833	6.432091371	9.35E-11	9.30E-10	Up
KRT17P1	-2.615918365	-1.808490228	1.52E-06	8.08E-06	Down
LINC0168	2.518887245	-1.12452781	0.000461012	0.001474807	Up
PCDH11X	-2.616068427	1.533741166	0.000152223	0.000543784	Down
DNAH17	-2.61740309	2.725562378	1.21E-16	2.34E-15	Down
G3BP1P1	2.518568074	-2.260033251	1.45E-05	6.41E-05	Up
DBF4	2.515748311	4.353529343	1.31E-10	1.27E-09	Up
PKMYT1	2.514956433	3.818379408	3.61E-06	1.79E-05	Up
TICRR	2.513928709	2.774046278	3.12E-06	1.57E-05	Up
CFL1P2	2.513400894	-0.605804542	2.23E-12	2.72E-11	Up
LINC0276	2.51103439	-1.499806657	2.27E-05	9.66E-05	Up
SIGLEC9	2.51011621	3.437032229	1.52E-05	6.69E-05	Up
NOC2LP2	2.509411521	-1.383866244	9.42E-08	6.09E-07	Up
CDYL2	-2.617592047	3.298787407	1.48E-23	5.72E-22	Down
CHAD	-2.619010288	0.128117299	5.30E-07	3.05E-06	Down
HSPG2	2.507520786	6.473705813	7.47E-05	0.000285832	Up
BSPRY	-2.620123275	-0.390255415	3.44E-10	3.16E-09	Down
RPL7P15	2.507496584	-2.104267452	9.54E-07	5.25E-06	Up
AC010878.1	2.507182381	-2.105202348	1.21E-05	5.45E-05	Up
PABPC1L	2.505689755	4.910130568	5.84E-05	0.000228421	Up
MRAP2	-2.620342293	2.788963094	8.39E-09	6.32E-08	Down
TP53I3	2.505494016	4.45739565	4.67E-11	4.86E-10	Up
DLG3	-2.620983956	4.606013654	6.87E-38	1.04E-35	Down
HLA-DRB1	2.50407516	8.438067712	5.79E-05	0.000226298	Up
WBP1LP2	2.503512784	-0.263554177	9.74E-07	5.35E-06	Up
JAG1	2.502902185	6.937920071	2.51E-06	1.28E-05	Up
TMEM63C	-2.62159334	3.426859576	3.64E-14	5.41E-13	Down
MCRIP2P1	2.502819044	-1.701518953	7.87E-05	0.000299638	Up
MAP3K10	-2.622356603	4.923132486	3.51E-34	3.71E-32	Down
PTMAP4	2.50231608	0.60648523	1.75E-09	1.46E-08	Up
AC027130.1	-2.622509191	-1.122901658	1.39E-08	1.01E-07	Down
LINC0134	-2.622519975	-2.550540082	0.00026839	0.000909597	Down
RBBP8	2.50228154	5.303897603	1.49E-14	2.29E-13	Up
ZNF483	-2.624405416	1.152324124	2.44E-23	9.15E-22	Down

LINC0229¿	-2.626571271	-1.665494538	6.97E-09	5.31E-08	Down
AC109466.1	-2.626939466	-3.131966875	0.000151769	0.000542363	Down
KCTD8	-2.627386814	1.606919781	1.84E-09	1.52E-08	Down
PSMA6P2	2.502137484	-2.256488904	8.37E-05	0.000316931	Up
AC093816.1	2.49968658	-2.688217109	0.000146101	0.000524187	Up
AC104260.1	-2.627480656	-3.628975459	1.13E-05	5.10E-05	Down
AC093155.1	2.499034312	-2.685254494	0.000117333	0.000429641	Up
GIMAP2	2.498771135	3.890041291	2.61E-07	1.58E-06	Up
HDAC11	-2.628805068	5.058976634	7.56E-34	7.81E-32	Down
EPHB4	2.497389274	5.390582874	9.72E-06	4.44E-05	Up
AC023906.4	-2.629437973	-3.164599998	1.82E-09	1.52E-08	Down
PDIA4	2.49651296	7.93719759	1.19E-10	1.17E-09	Up
SH3BGRL2	-2.629645581	4.248946766	5.04E-25	2.24E-23	Down
RPL7AP8	2.496209976	-1.832387591	1.20E-06	6.49E-06	Up
CHRM5	-2.630137036	-0.8622266	2.24E-09	1.84E-08	Down
MASP1	2.495586492	6.392010391	0.000251536	0.00085811	Up
CDKN2D	-2.630817333	3.937485494	3.34E-32	2.89E-30	Down
PDIA5	2.494295394	3.880897172	1.64E-07	1.02E-06	Up
PDP1	-2.630968096	4.934542409	3.60E-33	3.43E-31	Down
AC012085.1	2.4941122	0.80769747	1.43E-11	1.58E-10	Up
DBH	-2.631042979	-0.473210572	1.76E-12	2.16E-11	Down
GFRA2	-2.632816645	2.937134957	5.69E-09	4.39E-08	Down
GUCY1B1	-2.632966786	4.909619651	1.55E-21	4.91E-20	Down
IDH1	2.493582662	7.157856196	4.90E-12	5.75E-11	Up
AP002847.1	-2.633413761	-0.643280819	7.24E-20	1.93E-18	Down
GRIA2	-2.633537753	6.106800317	2.72E-11	2.91E-10	Down
NUFIP1P1	2.491642373	-2.95285485	0.000503949	0.001596567	Up
AC100793.3	-2.63477374	-1.877955865	4.59E-14	6.73E-13	Down
AL096701.1	2.490755768	-1.952984642	2.95E-05	0.000122655	Up
AL445433.2	-2.635567943	-2.971398866	1.56E-08	1.13E-07	Down
CFAP74	-2.636967336	0.463188819	4.60E-09	3.61E-08	Down
VAMP5	2.490457488	6.166355467	2.41E-05	0.000101993	Up
AC068896.1	-2.637118488	-1.989920234	4.36E-16	8.01E-15	Down
AL122018.1	2.487532441	-1.171359464	4.08E-07	2.40E-06	Up
AL390755.2	2.486937137	0.206670223	0.000118417	0.000433271	Up
NRNPA3P	2.486158257	-1.011365746	8.11E-10	7.06E-09	Up
Z99716.1	-2.637970434	-3.236721986	7.67E-10	6.69E-09	Down
FLNA	2.485787552	9.627330636	1.81E-07	1.12E-06	Up
NIPAL3	-2.638840814	5.690221867	8.93E-22	2.89E-20	Down
PCNPP5	2.485763543	-1.33459048	2.42E-09	1.97E-08	Up

AC099850.3	2.484130879	1.038127497	7.30E-11	7.38E-10	Up
CBX6	-2.638841649	6.468893671	2.69E-34	2.89E-32	Down
TNRC18P3	2.482644038	-2.159150213	3.47E-05	0.000142151	Up
NRNPA1P	2.482388371	-2.939056933	0.000347853	0.001145227	Up
CELF2-AS1	-2.639353578	-1.964820961	1.15E-09	9.80E-09	Down
EIF3CL	2.482018007	1.331949444	4.16E-11	4.35E-10	Up
TMOD2	-2.639693696	6.690921963	1.07E-30	7.95E-29	Down
TMEM176I	2.480765973	6.991205016	0.000314509	0.001046571	Up
SMCR5	-2.641082777	-2.827123198	6.14E-10	5.43E-09	Down
DLEU1	2.480203811	3.01327188	3.63E-10	3.32E-09	Up
MAP3K21	-2.64211135	0.32132756	4.46E-15	7.23E-14	Down
C4orf54	-2.642447088	-2.44005244	2.21E-07	1.35E-06	Down
PGM2L1	-2.643455798	5.84679599	2.03E-21	6.34E-20	Down
NYAP1	-2.644419299	4.19034796	1.18E-20	3.39E-19	Down
BCL2L2	-2.645137132	5.416776568	9.77E-36	1.18E-33	Down
FAM72C	2.480158956	-1.039701135	8.01E-05	0.000304576	Up
RPL7P24	2.480087729	-2.686994687	4.80E-05	0.000190829	Up
DGKB	-2.646505288	4.198358927	2.64E-11	2.83E-10	Down
SLC7A10	-2.647169912	1.581035861	3.58E-05	0.000146001	Down
EEF1GP8	2.479289022	-2.946053315	0.000199328	0.000694071	Up
PTMAP8	2.479057768	-1.420422394	8.54E-09	6.42E-08	Up
PPP1R3B	2.478896889	4.631859144	8.20E-07	4.56E-06	Up
FKBP7	2.477194365	3.527943158	4.20E-07	2.46E-06	Up
KCNK3	-2.647251488	2.595843845	3.96E-10	3.59E-09	Down
SHISA7	-2.648152227	3.937923922	1.41E-06	7.56E-06	Down
TNFAIP6	2.476586006	4.121435472	0.000818815	0.002472578	Up
RUNX3	2.475664126	2.756197978	2.87E-07	1.72E-06	Up
HMGB1P4	2.47525871	-2.708195066	0.000240241	0.000823889	Up
IL1RAP	2.475203835	6.385329835	0.00039582	0.001285811	Up
IACTR3-A	-2.649116149	-1.443190654	1.29E-05	5.76E-05	Down
AC025161.1	2.473187833	-2.699547089	4.90E-05	0.000194322	Up
BRSK2	-2.649794064	4.816475426	6.34E-12	7.32E-11	Down
AC110602.1	2.47307777	-2.933255403	0.000316318	0.001051742	Up
LRRC36	2.471572807	1.460514203	0.000122762	0.00044736	Up
ZNNT1	-2.649936526	2.367496053	1.49E-24	6.32E-23	Down
AAK1	-2.650832749	5.462150232	1.82E-49	8.75E-47	Down
AC006210.2	2.471502997	-1.98865412	9.52E-06	4.35E-05	Up
AL359636.2	-2.651316521	-3.059792088	2.52E-06	1.29E-05	Down
PLPPR4	-2.651359479	5.495267267	3.65E-16	6.79E-15	Down
H3TC2-D	-2.651718097	-2.428501139	1.78E-09	1.49E-08	Down

PDXP	-2.651870768	1.670859913	2.01E-19	5.10E-18	Down
AC027801.3	2.469898931	-2.962507078	0.000472355	0.001506652	Up
FAM192BP	2.469339107	-0.756950862	1.92E-09	1.59E-08	Up
AL391422.1	2.469045321	-0.846069655	0.000174948	0.000616856	Up
SCAMP5	-2.653018085	6.924938448	1.10E-33	1.11E-31	Down
AC097533.1	2.468261485	-2.979823933	0.000416243	0.001344877	Up
AL031133.1	2.467698745	-2.965763895	0.000461801	0.001477004	Up
DUSP26	-2.656517462	3.961204092	1.43E-10	1.38E-09	Down
AL391261.3	2.463532847	-2.311907537	4.69E-05	0.000186549	Up
C3orf49	-2.656816947	-1.569749517	3.08E-28	1.79E-26	Down
DOCK9	-2.657535307	5.120995417	1.04E-26	5.34E-25	Down
ST13P6	2.463141489	0.370772437	8.25E-10	7.16E-09	Up
GIPR	-2.658083956	0.215695605	1.06E-09	9.09E-09	Down
AKNAD1	2.462512634	1.536951447	0.00022536	0.000775811	Up
TSPYL5	-2.658874949	3.642965906	4.69E-11	4.88E-10	Down
CAMKK2	-2.659146367	5.785712323	2.26E-69	1.11E-65	Down
THAP12P1	2.461613308	-2.975122697	0.000515061	0.001628729	Up
SKP1P1	2.461169103	1.611564059	7.16E-13	9.21E-12	Up
SAPCD1	2.461078316	0.361469025	3.16E-05	0.00013056	Up
PRR29-AS1	-2.659216872	-2.869362227	1.70E-07	1.06E-06	Down
C1QC	2.458658701	8.629408386	3.76E-05	0.000152942	Up
SAMD9	2.458089822	4.8090198	8.23E-05	0.000312158	Up
RAPGEF3	-2.659250693	4.066011628	3.26E-28	1.89E-26	Down
NAV2-AS2	-2.660665947	-3.292296169	4.70E-10	4.22E-09	Down
RPS17P2	2.457682951	-1.729246288	2.29E-06	1.18E-05	Up
FAM111A	2.457582156	5.354461876	1.17E-11	1.31E-10	Up
SLC45A3	-2.661567576	2.890006524	2.94E-11	3.13E-10	Down
BMF	2.455877111	3.820453307	1.69E-06	8.92E-06	Up
AC021188.1	2.455007503	0.53971252	4.91E-06	2.37E-05	Up
CD300LF	2.454487887	2.181949663	9.45E-05	0.000354004	Up
CD74	2.452731695	11.03054841	1.42E-05	6.31E-05	Up
ACTL6A	2.452187292	5.743576667	2.71E-14	4.06E-13	Up
CETP	2.451596977	1.727093901	4.26E-06	2.08E-05	Up
DGRA1-AS	-2.661882752	0.645471134	3.53E-08	2.44E-07	Down
KRT1	-2.663470279	-3.315199874	4.42E-06	2.15E-05	Down
VAMP2	-2.663858926	7.513119731	1.03E-38	1.69E-36	Down
ADTRP	-2.664238172	-0.641927688	1.58E-08	1.14E-07	Down
NIPAL4	-2.664696365	1.866973029	5.07E-11	5.24E-10	Down
AL441963.1	2.449707686	-1.884341341	5.46E-06	2.61E-05	Up
GLRA2	-2.664943546	1.698483422	1.37E-05	6.09E-05	Down

SLC25A22	-2.66498091	5.590481402	2.71E-38	4.30E-36	Down
TMEM154	2.449110236	2.972795254	8.29E-06	3.84E-05	Up
PTMAP10	2.448737891	-2.157502019	1.42E-05	6.31E-05	Up
EVPL	-2.667253827	-2.069890214	3.94E-08	2.70E-07	Down
ANKRD22	2.447633744	2.583607935	0.000900927	0.00269092	Up
AL354798.1	-2.667301299	-1.959634062	1.46E-17	3.10E-16	Down
TLN2	-2.668699194	5.891266044	1.16E-35	1.39E-33	Down
PLEKHM3	-2.669621139	1.23092337	4.30E-26	2.08E-24	Down
CHST1	-2.670310702	4.89557567	3.58E-16	6.69E-15	Down
AC012213.4	-2.673393182	-2.711143063	6.53E-05	0.000253035	Down
CTSK	2.447272617	5.005811268	0.000956971	0.002840647	Up
CSRNP3	-2.674287188	4.242708665	1.70E-18	3.95E-17	Down
FBXO5	2.446512738	4.081938011	1.94E-10	1.85E-09	Up
TMEM266	-2.674438985	0.827613169	4.58E-26	2.21E-24	Down
KCNC4	-2.676172636	3.40904302	5.83E-25	2.57E-23	Down
NTNG2	-2.677206743	3.852382474	5.35E-15	8.62E-14	Down
APCD1-AS	-2.678814599	-3.229613112	3.99E-09	3.16E-08	Down
RPS2	2.445083577	9.689414488	2.39E-15	3.99E-14	Up
RPL6P27	2.444285592	3.445404557	6.16E-15	9.85E-14	Up
PTK7	2.440497174	5.944098601	4.16E-10	3.76E-09	Up
AC090877.2	-2.678891685	-3.488428838	3.23E-05	0.000133008	Down
TSPYL1	-2.679257908	6.512350079	1.85E-61	2.81E-58	Down
CAPG	2.440455938	7.092738181	3.82E-07	2.26E-06	Up
RPL31P17	2.440101974	-0.789526325	9.96E-07	5.46E-06	Up
CSMD1	-2.679964517	3.457013353	3.44E-13	4.61E-12	Down
CAMK2N1	-2.682671116	7.412495247	3.98E-22	1.33E-20	Down
EVA1B	2.439601019	3.876342038	8.19E-07	4.56E-06	Up
RAB26	-2.683152278	2.756552819	4.84E-17	9.77E-16	Down
CRIP1	2.439372716	0.024593538	4.08E-05	0.00016447	Up
RAB6B	-2.684446477	7.008478173	4.97E-26	2.39E-24	Down
AC097468.2	-2.686029606	-3.197124041	2.40E-09	1.96E-08	Down
EDIL3-DT	-2.687154171	-1.633865121	4.21E-08	2.87E-07	Down
AC004921.1	2.439189801	-1.269440999	0.000709092	0.002170823	Up
OR1F1	-2.687227819	-2.983593917	2.10E-06	1.09E-05	Down
GAPDHP6	2.43816491	-2.727582181	0.000628363	0.001948521	Up
AP000553.2	2.436627876	-1.372620258	0.000568952	0.001783679	Up
ATF4P4	2.435881551	-0.15357155	3.32E-12	3.97E-11	Up
PSME2P6	2.435619542	-2.965189088	0.000547086	0.001719993	Up
SLC22A15	-2.687800666	2.673845524	1.00E-16	1.96E-15	Down
C1orf94	2.435200437	2.15100615	0.000180869	0.000635401	Up

SLITRK5	-2.689253941	3.416160115	2.84E-11	3.03E-10	Down
LCN10	-2.690746286	-3.32398453	7.94E-06	3.69E-05	Down
NXT1	2.43369378	4.876300153	3.07E-10	2.83E-09	Up
WBP11P1	2.433487286	-2.512004221	3.89E-06	1.92E-05	Up
AP001972.1	-2.692730978	-2.98414317	1.10E-11	1.24E-10	Down
AL355864.1	2.432160473	-2.743309258	0.000281523	0.000949192	Up
IGFBP4	2.430889896	7.078465881	8.47E-06	3.92E-05	Up
KIF3A	-2.697153905	5.266560726	3.79E-58	4.29E-55	Down
PDZD4	-2.697971433	6.617706546	3.86E-23	1.42E-21	Down
CYP26C1	-2.698841132	-2.080443311	1.31E-13	1.82E-12	Down
APOBEC3I	2.430444693	1.068466462	1.84E-05	7.95E-05	Up
MFSD6	-2.698991906	5.219921752	2.64E-33	2.55E-31	Down
PTPN20	-2.701564548	-0.138133829	3.24E-06	1.63E-05	Down
LGALS1	2.427592444	8.804106628	1.17E-05	5.26E-05	Up
PHACTR3	-2.701582139	4.67822691	3.29E-09	2.63E-08	Down
HID1	-2.702226687	4.610783219	6.99E-27	3.63E-25	Down
S100A16	2.427061615	7.979532778	6.25E-06	2.96E-05	Up
AC120036.3	-2.702349972	-1.166759321	1.40E-18	3.30E-17	Down
LILRA6	2.426642756	0.838012253	0.000736931	0.002246983	Up
RPL12P10	2.426627561	-2.338529688	2.47E-05	0.000104248	Up
NRNPA1P	2.426246867	-2.984584081	0.000333921	0.00110428	Up
AC005077.4	-2.703164921	-1.564993113	2.62E-06	1.33E-05	Down
UBA52P3	2.426077139	-1.885747225	7.51E-07	4.21E-06	Up
AC090409.1	2.425883439	-0.502163703	4.88E-05	0.00019364	Up
AC114811.2	-2.703493049	0.811691172	3.93E-22	1.32E-20	Down
PPIAP9	2.424810355	-2.033258234	1.50E-05	6.61E-05	Up
LINC0150[illegible]	2.424673146	-1.562010029	0.000150155	0.000537328	Up
PGPEP1L	-2.704653412	-3.350056529	8.53E-07	4.74E-06	Down
RPS3AP18	2.424594529	-1.566252627	1.63E-06	8.62E-06	Up
PROCR	2.424186132	3.709080596	7.77E-07	4.34E-06	Up
SLC1A5	2.423535292	4.971820171	2.06E-06	1.07E-05	Up
SLC37A2	2.423279362	4.181054314	1.18E-05	5.31E-05	Up
PLAC9P1	-2.705848892	-1.868525006	1.27E-06	6.84E-06	Down
APOC2	2.422753005	1.857318291	0.00016294	0.000578125	Up
NECTIN1	-2.706889117	5.528896769	5.71E-19	1.39E-17	Down
GABPAP	2.422346959	-2.549474543	0.000246319	0.000842625	Up
CYFIP2	-2.710371574	6.732935758	4.98E-30	3.46E-28	Down
SP7	-2.711150119	-2.049821503	4.56E-07	2.66E-06	Down
SLC22A25	-2.711278413	-3.451108301	2.93E-06	1.48E-05	Down
C1S	2.421580788	7.776941256	0.000694246	0.002129897	Up

\L031587.4	-2.711513863	-3.295095672	3.37E-05	0.000138338	Down
XRCC2	2.420253557	2.719958405	2.24E-06	1.16E-05	Up
CDT1	2.419325243	4.196582986	2.53E-06	1.29E-05	Up
NUPR2	-2.71203861	-1.986695456	4.35E-06	2.12E-05	Down
\C109454.1	2.417740529	-1.782437956	8.40E-07	4.67E-06	Up
ZWINT	2.417203561	4.388464596	7.70E-08	5.05E-07	Up
RPL23AP48	2.415441387	-2.971780619	0.000329074	0.001090127	Up
TENM3	-2.7138718	4.141995517	4.28E-06	2.09E-05	Down
:YP2U1-AS	-2.714424564	-0.64096777	1.76E-08	1.27E-07	Down
UBE2NL	2.415293414	-2.363388319	2.43E-05	0.000102895	Up
CDKN3	2.41520233	3.610589785	1.94E-06	1.01E-05	Up
\C010327.4	-2.714632071	-2.781577603	1.23E-08	9.08E-08	Down
RPL39P18	2.414541493	-3.001437622	0.000412748	0.001335232	Up
EPB41L1	-2.715537141	6.606804612	3.57E-44	9.97E-42	Down
\L132800.1	2.414372134	-1.495372573	5.27E-05	0.000208044	Up
\C005381.1	-2.717022428	-3.387055109	6.82E-06	3.21E-05	Down
SYTL4	2.41425803	5.389329335	1.93E-06	1.01E-05	Up
UNC5C	-2.718214838	3.438109436	1.39E-09	1.18E-08	Down
RPS6P25	2.412824194	0.872993065	1.15E-09	9.81E-09	Up
TMEM144	-2.720985117	5.675770308	1.22E-10	1.19E-09	Down
\C107956.1	2.411414654	1.895051635	7.73E-08	5.07E-07	Up
PGAM1P4	-2.721179617	-2.031371832	1.44E-11	1.59E-10	Down
\C092384.3	-2.721531108	-3.105814852	9.74E-10	8.40E-09	Down
NPY6R	-2.721782965	-3.115627056	4.16E-07	2.44E-06	Down
\C002094.5	2.411339096	-1.302928169	0.000696134	0.002135009	Up
\C006450.3	-2.721832489	-0.707118213	1.31E-08	9.57E-08	Down
\L158847.1	-2.724513743	-2.59040131	8.27E-12	9.41E-11	Down
SH2D1B	-2.725037224	-1.879198537	4.05E-11	4.25E-10	Down
HELLS	2.410322438	3.361339058	1.41E-06	7.57E-06	Up
IDS	-2.725216728	8.322084505	4.97E-28	2.84E-26	Down
NPM1P50	2.409991097	-2.995079589	0.000631475	0.001956908	Up
PPIAP11	2.409910715	0.650111322	3.01E-09	2.42E-08	Up
RUSC2	-2.725359285	5.665680717	4.03E-38	6.21E-36	Down
RPL39P5	2.409580174	-2.039792862	3.94E-05	0.000159116	Up
TBC1D26	-2.726676536	-2.975912812	7.44E-09	5.64E-08	Down
PCNA	2.408058886	7.086675451	2.08E-12	2.54E-11	Up
HSPB3	-2.729430932	-0.214968439	1.81E-05	7.85E-05	Down
SORBS2	-2.729879391	4.498395558	4.54E-19	1.12E-17	Down
PSMB8-AS1	2.40698918	3.588535459	2.14E-07	1.31E-06	Up
\P001360.1	-2.730588366	-3.17378543	2.12E-05	9.05E-05	Down

PHEX	2.405840526	3.103101075	2.06E-07	1.27E-06	Up
TMEM179	-2.731101941	3.900959449	6.39E-09	4.90E-08	Down
ATP4A	-2.731368394	-2.817281942	3.25E-10	3.00E-09	Down
:FZP434H	-2.732104837	-2.979490551	9.45E-10	8.16E-09	Down
[TND4LP3	2.404700552	-0.006215549	4.63E-06	2.24E-05	Up
RHOH	2.404333519	1.707845854	7.46E-05	0.000285458	Up
GAL3ST4	2.403123315	6.041085515	4.90E-11	5.09E-10	Up
NSA2P7	2.402900586	-1.66517057	2.54E-06	1.30E-05	Up
FABP6	-2.732809167	-0.230063538	4.62E-08	3.14E-07	Down
DEFB131E	-2.733006921	-2.482820174	9.84E-14	1.40E-12	Down
KCNE3	2.402421001	3.56611894	1.18E-06	6.41E-06	Up
LILRB3	2.401351947	1.356860737	0.000258766	0.000880709	Up
CALHM6	2.399984844	2.949120576	0.000697117	0.002137341	Up
CAPN3	-2.733763855	2.358685331	1.41E-10	1.36E-09	Down
AC092384.2	-2.735240484	-2.919852569	2.92E-09	2.35E-08	Down
SEPTIN12	-2.736274044	-2.921129067	2.11E-08	1.51E-07	Down
AC093809.1	2.399959271	0.101966863	9.23E-05	0.000346297	Up
LINC0160	-2.736318839	0.266441337	8.05E-07	4.48E-06	Down
ZNF890P	-2.736548864	-2.060248427	3.34E-10	3.07E-09	Down
RNASEH2A	2.399368934	5.466347794	4.79E-09	3.74E-08	Up
AC015712.0	-2.736649693	-1.028286858	2.71E-10	2.53E-09	Down
LINC0114	-2.737341766	-0.385697866	6.48E-23	2.33E-21	Down
ARPC3P1	2.397651838	-0.777649987	1.25E-08	9.19E-08	Up
NACAP2	2.397565682	-3.003474015	0.000844873	0.002543534	Up
PLCH1	-2.737875665	2.628387784	3.73E-14	5.52E-13	Down
AC079907.1	2.396761686	-2.550788627	7.77E-05	0.000296159	Up
AL078604.1	2.396180873	-1.786950946	7.76E-06	3.61E-05	Up
AC068418.2	-2.738626198	-3.572304158	9.45E-06	4.32E-05	Down
FUNDC2P	2.394661468	0.165936564	1.34E-07	8.50E-07	Up
AC004816.1	-2.739534328	-0.272598138	5.65E-10	5.02E-09	Down
EGR3	-2.740083609	4.561058829	2.19E-11	2.37E-10	Down
YBX1	2.393826146	9.223371888	7.75E-16	1.37E-14	Up
HAUS6P1	2.39378309	-2.557485872	0.00050749	0.001607256	Up
HCP5	2.391567013	4.445337507	0.000672699	0.002070627	Up
AKR1B1P1	2.391460306	-2.55072245	2.00E-05	8.61E-05	Up
AC104046.1	2.389882681	-2.055035939	0.000324185	0.001075045	Up
FLVCR2	2.38959646	3.597893105	1.97E-08	1.41E-07	Up
ZNF385B	-2.740165954	1.755036984	3.07E-06	1.54E-05	Down
SYT3	-2.740555216	2.81454842	4.84E-15	7.82E-14	Down
RPS3AP43	2.38853697	-1.923693521	5.66E-05	0.000221943	Up

RAB15	-2.741419689	5.020760836	1.61E-28	9.63E-27	Down
BCL11B	-2.741854176	1.534778932	7.02E-11	7.11E-10	Down
DISP2	-2.746084142	3.966247241	9.92E-16	1.73E-14	Down
PINLYP	2.385737051	3.815138288	3.65E-06	1.81E-05	Up
AC004690.2	-2.747542593	-1.886073386	4.00E-06	1.97E-05	Down
CD80	2.38532668	-1.032702454	0.000646415	0.001997192	Up
SMC4	2.383744011	5.072337264	8.17E-09	6.16E-08	Up
PCSK6	-2.748042383	3.590896525	4.46E-08	3.03E-07	Down
AL732414.1	2.381480816	-2.388684207	1.11E-05	5.02E-05	Up
PWAR5	-2.749273864	-0.000256482	3.43E-12	4.10E-11	Down
CCL5	2.381217126	2.848517228	0.000954511	0.002834222	Up
DPYD	2.380558892	5.744738648	0.000181625	0.000637668	Up
PLIN2	2.379739257	6.258411827	0.00025843	0.000879774	Up
AL589765.7	-2.750294442	-2.781037282	9.50E-09	7.10E-08	Down
RPL3P6	2.379185457	-1.231542692	7.52E-08	4.95E-07	Up
NRNPA1P	2.376483223	-0.406644424	1.60E-09	1.34E-08	Up
AL663058.1	2.373863293	-2.772279648	0.000192528	0.000672587	Up
CEND1	-2.75125525	5.570643053	4.10E-19	1.02E-17	Down
MAST1	-2.751808964	4.680632025	1.41E-13	1.96E-12	Down
ADAMTS1(	-2.753027087	0.382181576	1.31E-09	1.11E-08	Down
RN7SL138I	2.372315763	-0.287225666	3.37E-05	0.0001382	Up
XRCC6P5	2.372092805	-2.769888886	0.000276949	0.000935523	Up
SH2D4A	2.371573852	3.01083905	1.14E-05	5.13E-05	Up
RASD2	-2.753635445	3.422219465	1.22E-17	2.62E-16	Down
SMPD4P1	-2.753914648	-3.023085656	4.76E-07	2.76E-06	Down
SLC2A10	2.370887177	5.069441441	5.34E-07	3.07E-06	Up
LY6D	-2.756209602	-3.798786266	0.000379517	0.001238297	Down
LAMA4	2.369403873	6.490630236	8.92E-06	4.11E-05	Up
LAMP5	-2.756260587	4.5719629	3.73E-12	4.43E-11	Down
SHTN1	-2.756725453	5.610000312	9.99E-19	2.38E-17	Down
AC008897.3	-2.757957141	-2.042565275	6.80E-20	1.82E-18	Down
HMGB1P1	2.369368588	0.257551951	5.46E-07	3.13E-06	Up
YWHABP2	-2.758738385	-2.209494783	8.26E-26	3.90E-24	Down
DNALI1	2.36846441	6.06889717	1.74E-06	9.20E-06	Up
FTH1P21	2.36542237	-2.569112582	0.000112218	0.000412694	Up
GCNT4	-2.75954731	0.666889829	1.66E-17	3.51E-16	Down
RP2	2.365386661	4.973103255	5.75E-16	1.04E-14	Up
ASPG	-2.759624393	-2.745012833	5.19E-07	2.99E-06	Down
STK32C	-2.759651051	4.38499375	7.36E-42	1.67E-39	Down
RPL37P6	2.365171524	-0.97213644	7.79E-08	5.10E-07	Up

TAP1	2.365141614	7.040897795	5.47E-05	0.000214992	Up
SYNJ1	-2.761519592	5.239245987	4.57E-62	8.23E-59	Down
CHST14	2.363684049	5.293487876	5.53E-13	7.19E-12	Up
RNASE6	2.36204481	4.60019698	3.31E-05	0.000136006	Up
CASP5	2.360178672	-0.660443453	0.000173784	0.000613126	Up
TXBP5-AS	-2.761983773	0.374745901	2.09E-19	5.29E-18	Down
CD68	2.359439982	1.52805887	3.72E-05	0.000151172	Up
KLC3	-2.76245351	-2.816646928	3.76E-15	6.14E-14	Down
KLHL3	-2.7633336	2.791233155	1.14E-24	4.89E-23	Down
SRGN	2.359120107	7.373032477	0.000420741	0.001358192	Up
AC022306.1	2.357404076	-2.24702033	9.66E-06	4.41E-05	Up
ZMAT4	-2.764332134	1.505992879	2.05E-07	1.26E-06	Down
PEBP1P2	2.357293796	0.264173835	1.80E-10	1.72E-09	Up
MYL6P2	2.356946754	-2.789416852	0.000378838	0.001236345	Up
WEE2-AS1	2.356540276	2.638522761	5.37E-08	3.60E-07	Up
ST13P15	2.355485399	-0.635782094	4.44E-08	3.02E-07	Up
SEMA6B	-2.764491545	5.23391043	3.91E-16	7.25E-15	Down
TF	-2.764806809	7.547853725	8.44E-09	6.35E-08	Down
SEPTIN7P	2.35455627	-0.763819541	6.20E-07	3.53E-06	Up
MXD3	2.354468067	4.009960228	4.12E-06	2.02E-05	Up
DCLK3	-2.76533287	0.241829437	1.48E-09	1.25E-08	Down
SNCAIP	2.354392177	4.837454427	1.49E-05	6.60E-05	Up
PTGES3P1	2.354096549	2.981704824	2.27E-14	3.43E-13	Up
TMEM171	-2.766096252	-1.026333395	5.24E-09	4.07E-08	Down
SORCS1	-2.766465868	3.137312805	2.01E-08	1.44E-07	Down
XKR4	-2.769604899	3.022085265	2.53E-09	2.06E-08	Down
RPL36AP1	2.353097335	-2.776618716	0.00013741	0.000495722	Up
CD300A	2.352512778	4.817219458	1.50E-05	6.63E-05	Up
AC011374.	-2.770660858	-2.199012997	4.70E-11	4.89E-10	Down
MYCBP2	-2.771966567	5.615876573	4.27E-56	4.11E-53	Down
RPL12P42	2.352412741	-1.751120213	1.53E-05	6.72E-05	Up
CACNA2D	-2.772293022	4.521580832	1.74E-11	1.91E-10	Down
AC087482.	-2.773019466	-2.257798529	7.17E-07	4.04E-06	Down
FNTAP2	2.352318757	-3.022854862	0.000340825	0.001124273	Up
SNX20	2.352145258	2.64779907	0.000152824	0.000545525	Up
TMEM244	-2.773823578	-3.400569756	0.000310581	0.001035027	Down
GNG12	2.35144753	7.163155839	3.40E-12	4.06E-11	Up
CISH	2.35026732	3.045809662	0.000426686	0.001374305	Up
AC087854.	2.34998465	-1.966553351	4.75E-06	2.30E-05	Up
CACNB2	-2.775563416	3.283447574	7.25E-17	1.43E-15	Down

MAPK8IP3	-2.775736345	6.834534655	1.65E-29	1.09E-27	Down
NTM-AS1	-2.778004223	-0.228622985	5.09E-08	3.43E-07	Down
CDS1	-2.77969546	2.952002438	1.39E-12	1.73E-11	Down
BCAS2P2	2.349852471	-2.579802473	0.000188796	0.000660833	Up
AL049796.1	-2.780356285	0.182320769	1.88E-45	6.04E-43	Down
SGK2	-2.782157636	1.708246091	2.39E-15	3.99E-14	Down
SLFN12	2.348964354	2.020851872	9.09E-06	4.18E-05	Up
AP000679.1	-2.783331641	-3.483572925	6.09E-08	4.06E-07	Down
MEF2C	-2.785622452	6.136528564	5.94E-51	3.11E-48	Down
HSFY3P	-2.78771576	-2.953410393	1.02E-06	5.59E-06	Down
PVALB	-2.789043257	-0.022744621	9.41E-06	4.31E-05	Down
APOBEC3C	2.34700219	3.923446946	2.15E-05	9.19E-05	Up
LINC00499	-2.792438379	-1.586147535	0.000197827	0.000689262	Down
RPL18A	2.346095029	8.743245125	6.82E-07	3.86E-06	Up
CYS1	-2.792689775	1.017382731	4.35E-13	5.76E-12	Down
NPC2	2.344327712	7.804455112	2.87E-06	1.45E-05	Up
TAS1R1	2.343898475	0.366838289	4.60E-05	0.000183519	Up
AL358942.1	2.343750533	-1.342452807	6.29E-08	4.19E-07	Up
CRACDL	-2.796064373	4.326630897	5.92E-18	1.31E-16	Down
AC110056.1	2.343688973	-2.801304791	0.000727956	0.002223141	Up
AL450998.1	2.343311709	1.279697067	3.81E-06	1.89E-05	Up
BEGAIN	-2.797460199	3.350940167	4.46E-15	7.23E-14	Down
LRRC53	-2.79855844	-2.005898074	3.31E-06	1.66E-05	Down
PPFIA4	-2.799179383	4.582442639	1.66E-17	3.50E-16	Down
SLC2A13	-2.800243083	4.519707368	2.31E-23	8.69E-22	Down
TGIF2	2.341394243	4.571658449	4.90E-08	3.31E-07	Up
BCL2L10	-2.800371905	-2.5080764	1.15E-13	1.62E-12	Down
AC127496.5	-2.801150517	-1.110550115	3.28E-20	9.02E-19	Down
AL132780.2	2.341285883	-0.568486526	1.89E-05	8.16E-05	Up
AL138955.1	-2.801407163	-1.744059132	2.04E-20	5.73E-19	Down
PDE6G	2.341195478	-0.094215533	5.80E-06	2.77E-05	Up
NCF4	2.340986113	3.764553892	2.00E-05	8.60E-05	Up
CKLF	2.339461081	3.483995525	1.30E-10	1.26E-09	Up
WWP1P1	2.338271238	-2.800799058	0.00086802	0.002603428	Up
CLK2P1	2.337540701	-2.104388273	1.60E-06	8.48E-06	Up
AL590556.1	2.336505611	-2.094184871	6.32E-05	0.000245222	Up
POLR2F	-2.80433518	1.596914307	3.18E-11	3.37E-10	Down
RPS3AP20	2.335059213	1.640388618	8.29E-07	4.61E-06	Up
MYO5A	-2.804384135	6.268299313	4.22E-36	5.36E-34	Down
EMX2OS	-2.804674861	3.582934337	1.06E-11	1.19E-10	Down

AC015712.4	-2.80564268	-2.693204019	5.67E-09	4.38E-08	Down
FBXW7	-2.805800956	4.97067641	1.66E-47	6.38E-45	Down
TTC9B	-2.809824158	3.552899669	1.26E-08	9.28E-08	Down
TRIB3	2.334364619	4.838988241	0.000108105	0.000399455	Up
CD151	2.33402235	7.945424908	1.95E-07	1.20E-06	Up
MOG	-2.810267628	5.852808065	2.81E-06	1.42E-05	Down
ATP5MGP1	2.333367102	-2.796011664	0.000165922	0.000587333	Up
NAP1L3	-2.810554661	5.282472567	8.18E-33	7.44E-31	Down
CST7	2.331925973	0.77462881	0.000267801	0.000907814	Up
LINC02188	-2.811350521	-3.15020002	2.23E-10	2.10E-09	Down
PDLIM3	2.331450021	6.208088997	1.42E-05	6.31E-05	Up
HAUS1	2.330350585	4.783349666	2.26E-09	1.86E-08	Up
NT5C3AP1	2.329140624	-1.216765425	2.84E-07	1.71E-06	Up
CARTPT	-2.815619541	-0.14068622	0.000916283	0.002732254	Down
NCOA7	-2.816773269	4.721735077	3.77E-46	1.29E-43	Down
PPP3CB	-2.81726151	5.697184298	8.51E-60	1.17E-56	Down
RPL7P11	2.328989561	-2.791452651	0.000856863	0.002573163	Up
PPIAP25	2.327244724	-3.035886842	0.000784476	0.002376854	Up
AL132838.1	2.326858916	-1.861657007	1.13E-06	6.14E-06	Up
LRRTM1	-2.817570698	2.763664782	6.04E-11	6.17E-10	Down
EFNA4	2.326608889	2.20585691	7.74E-07	4.33E-06	Up
SHANK3	-2.817866689	5.110204033	3.45E-28	1.99E-26	Down
OCA2	-2.818873344	-0.267904711	0.000302904	0.001012748	Down
HAR1B	-2.820768163	-2.201595853	1.37E-06	7.37E-06	Down
C11orf42	-2.821333104	-2.511928818	6.09E-17	1.22E-15	Down
STMN4	-2.823834673	5.786494622	1.51E-11	1.67E-10	Down
MIR137HG	-2.823883863	-0.029396123	5.91E-08	3.94E-07	Down
NEXN	2.325050518	2.740421278	1.36E-05	6.05E-05	Up
PLAAT4	2.323923841	6.145534811	0.000698227	0.002139834	Up
AL135999.3	-2.824566309	-3.104351115	3.34E-12	4.00E-11	Down
RUNX1	2.323455853	5.430660472	0.000108471	0.000400599	Up
MIR25	2.322897667	-0.741324645	2.89E-05	0.000120466	Up
GLS	-2.825151835	5.834441739	5.65E-59	7.08E-56	Down
SCRT1	-2.825296034	2.727281309	5.85E-07	3.34E-06	Down
ETV1	2.320194894	7.665748169	1.61E-06	8.52E-06	Up
AL020997.1	2.31950598	-2.82299859	0.000994988	0.00294228	Up
TNK2-AS1	-2.825562458	-1.754629157	3.67E-12	4.37E-11	Down
AP3B2	-2.827771019	4.983698599	3.06E-24	1.26E-22	Down
GNAO1	-2.827894499	7.074667581	4.79E-22	1.59E-20	Down
SLC26A4	-2.82853104	0.991622165	5.97E-16	1.08E-14	Down

UQCRHL	2.319028915	3.482045342	1.46E-14	2.25E-13	Up
PTPRZ1	2.318957258	10.67878036	9.52E-06	4.35E-05	Up
KCNIP2	-2.828748275	3.630549099	7.21E-12	8.25E-11	Down
FCGRT	2.316902338	7.575226232	4.83E-10	4.33E-09	Up
DTYMK	2.316779085	5.776542058	1.13E-10	1.11E-09	Up
CFL1P5	2.316776493	-1.390597894	1.88E-09	1.56E-08	Up
RPL7P9	2.314592435	3.153342686	1.90E-11	2.07E-10	Up
LINC0101[illegible]	-2.829041868	0.314046423	8.00E-09	6.04E-08	Down
BASP1	-2.829106182	6.320499604	2.61E-14	3.92E-13	Down
AC113615.[illegible]	-2.83093331	-3.576349281	8.23E-06	3.82E-05	Down
CABLES1	-2.831575915	3.990552684	1.98E-23	7.46E-22	Down
GABBR1	-2.832041819	7.177023442	3.51E-21	1.06E-19	Down
TSPYL2	-2.83225527	5.893089189	3.21E-32	2.79E-30	Down
BTBD16	-2.835013706	-0.819591339	2.96E-16	5.56E-15	Down
AC008105.[illegible]	-2.836634686	-0.233215503	2.18E-17	4.54E-16	Down
AL354809.1	-2.836823787	-2.062463729	7.04E-17	1.40E-15	Down
PVT1	2.314103313	2.891694395	6.93E-09	5.28E-08	Up
GRAMD1[illegible]	-2.837142522	4.77082155	8.62E-27	4.42E-25	Down
RPL24P2	2.312414993	1.020397055	3.08E-07	1.84E-06	Up
SFTPC	-2.837734154	0.112485659	2.04E-10	1.94E-09	Down
PP1R14B-A	2.311909714	0.614424599	0.000441821	0.001418771	Up
EPHA5	-2.838843564	3.204149114	8.74E-13	1.11E-11	Down
MIR1249	-2.842109636	-2.711551103	1.65E-11	1.81E-10	Down
PLTP	2.31179266	8.845296332	6.68E-05	0.000257976	Up
CTXN3	-2.842718898	-1.410969083	0.000204009	0.000708913	Down
2CD4D-A[illegible]	-2.842842181	-0.098655482	5.51E-11	5.68E-10	Down
SYT12	-2.843651982	3.761123863	3.39E-16	6.34E-15	Down
AC004023.[illegible]	-2.845777516	-2.972247275	1.64E-06	8.66E-06	Down
MCTP1	-2.846134217	3.126659614	7.45E-20	1.98E-18	Down
KCNA2	-2.847523832	2.556525632	1.75E-20	4.94E-19	Down
AQP3	-2.85038648	1.537313122	6.13E-15	9.80E-14	Down
CNN3	2.311750433	9.743410514	4.57E-10	4.10E-09	Up
ZNF503-AS	2.311631897	-1.253474693	6.55E-06	3.09E-05	Up
MAGEA1[illegible]	2.311102243	-0.341664787	7.42E-05	0.000284232	Up
PAIP2B	-2.852254472	3.671695082	1.44E-13	2.00E-12	Down
RNF122	2.310727556	4.242866206	1.99E-06	1.03E-05	Up
FUT9	-2.852599921	4.285946857	2.29E-10	2.15E-09	Down
PEAK3	2.308726773	1.23063987	1.10E-05	4.99E-05	Up
AP001178.[illegible]	-2.852982187	-1.362933876	2.23E-15	3.74E-14	Down
MAD2L1	2.308574192	5.041484846	1.67E-06	8.83E-06	Up

AC011474.2	-2.853943407	-2.728195325	1.30E-05	5.79E-05	Down
ABARAPI	-2.854076979	6.47512187	6.85E-43	1.69E-40	Down
LRRC46	2.308437514	1.708212141	0.000273963	0.000926958	Up
STK17A	2.30837175	6.465014054	1.43E-06	7.64E-06	Up
KYNU	2.307568736	2.371521623	7.63E-05	0.000291446	Up
SCN5A	-2.855327585	0.259279788	1.78E-09	1.48E-08	Down
HMGB2	2.307495811	6.861750649	1.64E-10	1.57E-09	Up
CASP6	2.306874124	3.629892385	1.24E-13	1.73E-12	Up
UBE2V1P2	2.30661455	-1.165730968	8.65E-09	6.49E-08	Up
LINC0187	-2.855718815	-1.988674237	4.66E-09	3.65E-08	Down
IL2RG	2.306296567	2.32356277	0.000102521	0.00038092	Up
PANX2	-2.856175395	2.584541232	1.71E-22	5.97E-21	Down
PANTR1	2.304367745	5.488953094	3.31E-05	0.00013596	Up
IQSEC2	-2.859138392	4.920141613	5.36E-37	7.32E-35	Down
KIF3C	-2.861520529	6.468676656	3.22E-39	5.69E-37	Down
BST1	2.302478585	2.50424367	6.28E-06	2.97E-05	Up
NPFFR2	-2.86209682	-3.397796644	0.000116953	0.00042841	Down
MIAT	-2.866128624	5.17512996	2.74E-10	2.55E-09	Down
FAM155A	-2.866495335	3.283541749	7.66E-11	7.70E-10	Down
AC000403.1	-2.866606661	-0.05472004	1.31E-21	4.19E-20	Down
FABP5P3	2.30220403	-1.754339192	5.28E-05	0.000208221	Up
ZNF300	2.301914824	4.398424142	3.15E-06	1.58E-05	Up
DOCK5	-2.868492395	3.818686396	1.33E-14	2.06E-13	Down
ANCL1-AS	-2.869559861	-1.129770634	1.50E-26	7.61E-25	Down
BATF	2.301673655	0.765065353	0.000722283	0.002208155	Up
TTLL7	-2.872186629	5.197825485	9.62E-36	1.17E-33	Down
TK32A-AS	2.301455869	-0.302975857	0.000142953	0.000513788	Up
NETO1	-2.873505609	2.958018026	9.42E-07	5.19E-06	Down
AC093012.1	-2.875583835	-3.169734576	1.45E-14	2.24E-13	Down
AL133338.2	2.300723581	-2.289436801	5.65E-07	3.24E-06	Up
KIF19	-2.878201513	1.834723762	4.85E-10	4.35E-09	Down
ADAMTS8	-2.880596586	2.494843263	9.38E-14	1.33E-12	Down
LINC0164	-2.880756234	-2.624664901	6.64E-07	3.76E-06	Down
AL049749.1	-2.882766477	-1.995016256	3.05E-05	0.000126411	Down
PPIAP7	2.300504601	-2.823130015	0.000269873	0.000914191	Up
AMTS19-A	-2.883183175	-3.310264815	1.07E-07	6.90E-07	Down
LINC0158	-2.883213219	-2.245244733	4.55E-10	4.09E-09	Down
INPP5J	-2.885359496	2.091047289	7.68E-25	3.32E-23	Down
NSA2P2	2.298895834	-2.615565699	0.000196998	0.000686621	Up
RPL21P11	2.297802237	-0.918585883	4.11E-07	2.42E-06	Up

IL34	-2.886926835	2.82358739	4.34E-19	1.07E-17	Down
SPOCK1	-2.888007667	6.431706051	4.40E-12	5.19E-11	Down
API5P1	2.297732337	-1.502045969	1.25E-07	7.99E-07	Up
PHACTR1	-2.888010705	4.870245495	1.33E-23	5.16E-22	Down
POGLUT3	2.297089882	5.50864353	2.60E-09	2.11E-08	Up
AC026748.1	-2.888520648	-2.885960704	4.57E-17	9.24E-16	Down
HS3ST5	-2.889043051	0.798768684	1.12E-10	1.10E-09	Down
CAMSAP3	-2.889296352	2.809705728	1.30E-08	9.53E-08	Down
AC106872.2	2.296175491	-2.007859265	6.64E-05	0.000256885	Up
HSPA2	-2.889628679	6.206146111	5.11E-17	1.03E-15	Down
TNNI2	2.294980712	-0.277596332	6.28E-05	0.000243815	Up
AC091153.1	2.293927235	0.068098341	1.05E-08	7.79E-08	Up
AC020917.1	2.293765679	-1.8869417	3.52E-07	2.09E-06	Up
AC005540.1	-2.890733027	-3.161844494	5.63E-21	1.67E-19	Down
SGO2	2.293754867	3.695924033	2.57E-09	2.09E-08	Up
P2RX5	-2.891032157	0.101956103	1.90E-14	2.89E-13	Down
HSPA8P14	2.29363563	-3.078549278	0.000949755	0.002821261	Up
PI4KA	-2.89172934	6.773062954	1.84E-48	7.80E-46	Down
CARD8-AS	2.293453028	2.374482726	3.91E-07	2.31E-06	Up
MT2A	2.290927365	9.332204949	0.000723235	0.002209894	Up
GJC1	2.290701754	4.91373606	7.54E-12	8.61E-11	Up
LIPE	-2.893064093	3.744376106	1.71E-21	5.36E-20	Down
SPRY4	2.290666926	6.655980284	0.000676587	0.002080818	Up
LAIR1	2.290647923	5.692482691	8.17E-05	0.000309983	Up
LINC00641	-2.893175919	4.675834779	3.10E-27	1.66E-25	Down
AC022795.1	2.290171152	-2.625615447	0.000775922	0.002353413	Up
PLEKHG3	-2.893720564	4.710564787	1.36E-22	4.76E-21	Down
FBP1	2.289689937	3.407541603	0.000139492	0.000502414	Up
ARRB1	-2.893888904	4.243667361	3.64E-32	3.13E-30	Down
PNMA6A	-2.893968713	2.226983329	1.17E-15	2.04E-14	Down
LRRC73	-2.896810363	1.885389184	1.71E-21	5.36E-20	Down
BMP1	2.288758919	5.892672496	3.24E-08	2.25E-07	Up
AC100861.1	2.287607705	-1.247586289	0.000488268	0.001550982	Up
TRIM6	2.286364768	1.786374194	0.000107181	0.000396547	Up
EPB41L3	-2.897385454	5.677418683	7.25E-19	1.75E-17	Down
NSF	-2.899592418	6.351863558	6.06E-69	2.50E-65	Down
FAM237A	-2.899649636	-2.79617431	1.97E-12	2.41E-11	Down
HMGB1P37	2.286326808	-1.895406502	1.19E-06	6.44E-06	Up
ARL4AP5	2.285906394	-2.635661502	0.00032523	0.001078137	Up
POGLUT2	2.284820118	3.336290024	6.32E-09	4.85E-08	Up

CPEB1-AS1	-2.900825296	-1.677301556	1.19E-17	2.56E-16	Down
RAB20	2.284535765	4.415411351	7.82E-07	4.37E-06	Up
SNRPG	2.284382382	5.974018799	9.47E-09	7.08E-08	Up
SNPH	-2.900916454	5.018204851	9.78E-32	7.98E-30	Down
AL512622.1	-2.902277953	-2.798105827	1.21E-11	1.35E-10	Down
AC026369.1	-2.903262167	-2.610648962	2.43E-09	1.98E-08	Down
THEG	-2.906861534	-3.677141602	1.65E-09	1.38E-08	Down
CHEK1	2.284251967	4.31387079	1.17E-10	1.15E-09	Up
NDUFA4L2	2.282842961	6.134003482	0.0003116	0.001038091	Up
PCDH7	-2.907260736	4.417981558	3.12E-11	3.31E-10	Down
AC091182.2	-2.909295295	-1.977303855	2.55E-07	1.54E-06	Down
TMCC2	-2.910323559	4.546332017	6.44E-22	2.11E-20	Down
CCDC144B	-2.911160655	-0.53105974	6.58E-10	5.79E-09	Down
PCDHB9	2.282759838	3.826822041	3.91E-06	1.93E-05	Up
CD164L2	-2.913582095	-3.229918876	8.61E-06	3.98E-05	Down
KCNMA1	-2.913665308	5.300016682	1.86E-37	2.65E-35	Down
AL138690.1	-2.91383736	-3.354585626	4.40E-08	2.99E-07	Down
ITGA1	2.282679297	5.385158792	7.33E-07	4.12E-06	Up
AARD	-2.915263233	-1.911133758	1.00E-06	5.50E-06	Down
GOLGA7B	-2.916041789	4.069714357	1.35E-12	1.68E-11	Down
CACNB4	-2.91968375	3.939612699	3.84E-35	4.40E-33	Down
BOK	-2.919743634	4.174878872	6.54E-15	1.04E-13	Down
RPL19P4	2.282645828	-2.620794428	0.000104053	0.000386067	Up
GLI2	2.282420086	3.801989776	0.000633956	0.00196375	Up
GPIHBP1	-2.919852301	2.568442762	1.04E-13	1.47E-12	Down
PARP9	2.282359293	6.37065967	6.40E-08	4.25E-07	Up
UQCRFS1P	2.2819597	-0.081551104	8.99E-08	5.83E-07	Up
NOD2	2.281510594	1.938835693	0.000181569	0.00063755	Up
AL162171.3	-2.920115373	-0.473902023	1.16E-23	4.53E-22	Down
KLHL32	-2.920170358	3.330152656	4.32E-13	5.72E-12	Down
LYPLA1	2.280918266	6.298005114	2.37E-09	1.94E-08	Up
PLCXD2	-2.920661943	-1.025460293	1.77E-09	1.47E-08	Down
RPLP0P2	-2.920893678	1.251611093	7.23E-25	3.14E-23	Down
FAM189A1	-2.921718327	3.10939256	3.17E-17	6.50E-16	Down
GSTO2	-2.922487959	1.414900133	6.85E-20	1.83E-18	Down
SHISA8	-2.922574161	-1.206668733	7.56E-16	1.35E-14	Down
IBA57-DT	-2.924130187	-1.097034594	5.69E-19	1.39E-17	Down
MSANTD1	-2.92528161	-0.093699049	2.02E-29	1.32E-27	Down
TCIM	2.280620745	5.581047386	0.000679896	0.002090548	Up
CKS1B	2.279575033	4.46010434	2.99E-07	1.79E-06	Up

TXNP6	2.27852773	-1.337007215	1.20E-06	6.50E-06	Up
AL671277.1	2.278513216	3.370996037	0.000372361	0.001217155	Up
C1orf162	2.278142687	4.598140726	8.83E-06	4.07E-05	Up
CED1B-AS	2.27743368	3.805610985	0.000137062	0.000494527	Up
DIAPH3	2.2767403	2.762206398	5.31E-07	3.05E-06	Up
RPL3P4	2.275286069	4.134224708	1.28E-11	1.42E-10	Up
AC093827.1	2.273740771	-2.835484725	0.000134852	0.000487104	Up
PLCL1	-2.925284291	3.48980707	1.70E-19	4.37E-18	Down
PLSCR1	2.273479781	6.008263018	2.35E-06	1.21E-05	Up
GABRA3	-2.925557119	3.50402017	1.00E-07	6.47E-07	Down
RPL37AP8	2.272251507	-1.131948865	5.03E-07	2.91E-06	Up
AC093802.1	-2.925697872	-3.269758007	3.08E-08	2.15E-07	Down
ABCF2P1	2.272127573	-2.156644462	2.06E-06	1.07E-05	Up
AC010655.2	2.271909364	-0.218645902	0.000887189	0.00265484	Up
AC093908.1	2.271072134	0.857959971	0.000132248	0.000478529	Up
SP140L	2.269756596	3.504098429	5.49E-06	2.63E-05	Up
SLC9A7P1	-2.927325715	0.021122334	6.84E-17	1.36E-15	Down
APLP1	-2.927380632	8.095146621	6.68E-23	2.40E-21	Down
UNC5D	-2.929598637	2.838969357	5.10E-08	3.43E-07	Down
ST13P18	2.267891304	-1.267728527	6.82E-09	5.21E-08	Up
PRSS23	2.267457269	6.019976654	5.77E-05	0.000225879	Up
GRIP2	-2.929809291	1.727946454	3.70E-09	2.94E-08	Down
DYNC1I2P	2.267268196	1.440366241	2.48E-13	3.36E-12	Up
HLA-DOA	2.266510411	5.448872418	0.000137958	0.0004972	Up
AL355355.1	-2.93083164	-2.959161991	1.27E-13	1.77E-12	Down
CABP4	2.264939747	1.602183214	9.96E-06	4.54E-05	Up
HYAL2	2.26442041	6.226176816	6.76E-14	9.75E-13	Up
PLP1	-2.932102356	10.18593982	1.25E-09	1.06E-08	Down
EPN3	-2.936572039	-0.779039201	7.63E-13	9.76E-12	Down
SETP21	2.264358308	-2.836860138	0.000277247	0.0009362	Up
RPL34P34	2.26426108	-1.139663869	1.54E-05	6.79E-05	Up
MYD88	2.263360721	5.667229008	1.05E-13	1.49E-12	Up
HAPLN2	-2.93848886	4.375585927	1.07E-08	7.95E-08	Down
DLG4	-2.939470016	6.494917249	1.05E-52	6.58E-50	Down
SMIM17	-2.94136656	0.295590973	2.47E-24	1.02E-22	Down
KLK6	-2.942138515	4.417621056	4.47E-07	2.61E-06	Down
GRIN2C	-2.942795964	3.183094529	1.02E-11	1.15E-10	Down
MYRIP	-2.942953043	3.718652131	6.55E-17	1.30E-15	Down
AC104836.1	-2.944889157	0.024517872	8.96E-20	2.37E-18	Down
GPBAR1	2.263156779	-0.423190291	6.23E-06	2.95E-05	Up

NXPH2	-2.945684767	-0.616565381	1.09E-07	7.02E-07	Down
RTP4	2.261030261	2.778333136	0.000226286	0.000778811	Up
RSPO2	-2.945948573	2.359847789	1.43E-07	9.01E-07	Down
IR181A2H	2.26044359	0.069747296	0.00071841	0.002197155	Up
ZC3H12B	-2.946783527	1.759894816	2.84E-09	2.29E-08	Down
FCGR1A	2.260153575	4.900440166	0.000164463	0.000582812	Up
DPP10	-2.947319345	3.565922125	2.69E-08	1.89E-07	Down
ASPHD1	-2.947359403	4.228588116	3.98E-20	1.09E-18	Down
FAAH	-2.948632817	3.508876892	7.73E-22	2.52E-20	Down
ANKRD24	-2.948704699	2.879360573	2.23E-18	5.10E-17	Down
CSMD3	-2.94978742	1.981809106	7.08E-08	4.68E-07	Down
DHRSX	2.25998752	5.164123519	5.94E-13	7.71E-12	Up
FAM87A	-2.950902496	-1.594690809	4.81E-14	7.04E-13	Down
LCNL1	-2.954999148	2.208479205	7.25E-09	5.51E-08	Down
AC021739.2	2.259861351	1.854420319	5.43E-05	0.000213601	Up
ENSAP2	2.259381184	-0.440544862	6.74E-09	5.15E-08	Up
AL121929.2	-2.955266705	-0.166314757	1.92E-12	2.35E-11	Down
MGAT3	-2.956501207	5.056096863	2.79E-22	9.57E-21	Down
ELAPOR1	-2.95936094	2.946024841	2.03E-19	5.15E-18	Down
KNCN	-2.96236297	-1.014160807	9.55E-05	0.000357466	Down
TIMELESS	2.257938274	5.448712317	1.92E-08	1.37E-07	Up
LYPD1	2.257743479	5.966381147	8.42E-05	0.000318565	Up
RAP1GAP	-2.963477107	5.868428484	2.42E-21	7.47E-20	Down
PLPPR3	-2.963496742	2.37226581	1.61E-06	8.55E-06	Down
AC023302.1	2.257634517	-2.058059979	0.000946976	0.002814566	Up
AL358216.1	-2.965334132	0.828641661	1.66E-07	1.03E-06	Down
SLC1A2	-2.965514822	8.739948516	6.86E-16	1.23E-14	Down
PCDHGC5	-2.9683431	4.191126456	5.58E-17	1.12E-15	Down
SH3TC2	-2.969076426	2.060054221	2.08E-18	4.78E-17	Down
PRIM2	2.25688405	3.656003317	2.52E-15	4.19E-14	Up
RHOC	2.255742804	8.324794199	6.48E-10	5.71E-09	Up
NDUFA9P1	2.253980813	-2.300627325	7.92E-05	0.000301244	Up
DBI	2.253947098	9.195670363	1.90E-06	9.92E-06	Up
LILRA2	2.253445868	3.433014629	6.02E-05	0.000234722	Up
PNMA6B	-2.970290709	-1.574541087	8.40E-07	4.67E-06	Down
DRAM1	2.251865134	4.473944488	1.65E-07	1.03E-06	Up
INSM2	-2.970752904	-0.792578067	2.38E-07	1.45E-06	Down
AC012618.1	2.251491311	-0.875616502	1.04E-07	6.67E-07	Up
LPA	-2.971485061	-2.511116246	4.12E-08	2.82E-07	Down
DNAJB1P1	2.250187571	-2.317438936	7.00E-05	0.000269528	Up

AL031710.1	-2.972316954	-2.604758289	1.31E-06	7.07E-06	Down
SLC26A9	-2.972345313	-0.316668492	6.69E-12	7.69E-11	Down
DUSP8	-2.972840624	4.430950615	1.96E-22	6.80E-21	Down
CACNA1S	-2.976766672	-1.28255387	7.33E-09	5.56E-08	Down
VSTM5	-2.977431924	0.238983809	1.61E-13	2.22E-12	Down
PDLIM1	2.250041517	5.096501442	3.11E-05	0.000128609	Up
AC005821.1	2.249874189	1.30062473	0.000740294	0.002255559	Up
ESYT3	-2.981060452	0.542764531	1.60E-19	4.10E-18	Down
CEBPD	2.249521622	6.403144436	2.56E-05	0.000107727	Up
AC138028.2	-2.981928464	-0.71190988	2.99E-25	1.35E-23	Down
ATP5MGL	2.249384745	-2.040304043	2.75E-05	0.000115066	Up
AC079944.2	2.249076279	0.152349331	8.28E-18	1.80E-16	Up
RDM16-D	-2.982984379	2.272296872	2.21E-15	3.70E-14	Down
UBE2D3P1	2.248665504	0.263092692	1.41E-12	1.75E-11	Up
ATL1	-2.983450729	4.511989627	1.16E-48	5.06E-46	Down
NBEA	-2.987258979	4.50401636	2.95E-30	2.07E-28	Down
PPIAP49	2.248301196	-2.662696279	0.000182949	0.000641926	Up
GOT1	-2.988814798	5.49222466	1.08E-53	7.41E-51	Down
YPEL4	-2.989107755	2.863284277	3.05E-25	1.37E-23	Down
ACTL6B	-2.989151404	3.449705803	1.26E-07	7.99E-07	Down
SDHDP6	2.24816465	-1.423566056	2.54E-05	0.000106994	Up
COX6A1P2	2.247893136	2.757682669	4.06E-09	3.21E-08	Up
CACNA1F	-2.991709499	-0.81594933	2.16E-24	8.98E-23	Down
NAV3	-2.992356836	3.825962818	8.27E-18	1.80E-16	Down
OXGR1	-2.993468108	-2.172595473	3.22E-11	3.41E-10	Down
LYNX1	-2.994639901	5.860049461	9.60E-28	5.32E-26	Down
SERPING1	2.247363248	7.878517121	0.000240681	0.0008252	Up
CD99P1	2.247297254	2.02484546	9.56E-09	7.14E-08	Up
AC105046.1	-2.994983822	-1.982146645	3.41E-10	3.13E-09	Down
AGBL4	-2.996120407	1.296129976	1.32E-15	2.28E-14	Down
RTP5	-2.996352652	2.344047785	7.36E-08	4.85E-07	Down
LINC0193(	2.246929681	-1.190003416	0.000916493	0.002732598	Up
AL365205.4	-2.997308587	-3.370218765	1.06E-12	1.34E-11	Down
SMIM22	-2.997797464	-2.260252683	6.57E-12	7.56E-11	Down
TP53TG5	-2.998609937	0.587161328	1.26E-38	2.06E-36	Down
MSN	2.245828019	9.106040297	9.03E-10	7.81E-09	Up
HRH2	-2.998612532	0.820075736	1.49E-13	2.06E-12	Down
AA1671-A	-2.998664171	-1.896736483	5.62E-20	1.52E-18	Down
EEF1A1	2.245445958	12.51826518	1.62E-10	1.56E-09	Up
AC017076.1	-3.006706325	-2.611368981	4.15E-06	2.03E-05	Down

AP000755.2	-3.008203062	-3.521113303	2.04E-05	8.77E-05	Down
ASB9	2.243932682	0.73398097	0.000249094	0.000851208	Up
SAMD9L	2.243128488	5.518006124	0.000164422	0.000582789	Up
IGSF5	2.241814141	1.358005123	8.84E-05	0.000333137	Up
RPSAP9	2.240453065	1.018260073	2.68E-10	2.50E-09	Up
CHIC2	2.238986183	4.648011425	0.00076269	0.00231718	Up
NPIPB13	2.238777995	0.214821329	0.000108716	0.00040125	Up
PPIAP8	2.238347472	-2.49377699	6.49E-05	0.000251381	Up
TRABD2A	-3.008342191	0.748442775	2.95E-16	5.53E-15	Down
CNTN5	-3.008420293	1.456669624	1.23E-10	1.20E-09	Down
CAP1P2	2.237736456	-0.315836186	9.77E-13	1.24E-11	Up
AC012213.1	-3.009221193	-2.127320161	1.47E-06	7.84E-06	Down
DSG3	-3.009277163	-3.698774715	0.000179976	0.000632629	Down
ATP1B1	-3.012231298	7.239955318	4.63E-26	2.23E-24	Down
LDHAP4	2.236282626	2.549530473	1.41E-06	7.57E-06	Up
LINC02338	-3.01372388	-3.336065011	1.97E-09	1.63E-08	Down
LINC02881	-3.014656961	-2.94235763	1.77E-10	1.69E-09	Down
C17orf102	-3.017545391	-0.634332736	1.48E-11	1.63E-10	Down
AC010328.2	2.234840668	-1.733138161	3.45E-07	2.05E-06	Up
TEF	-3.018648462	4.953847308	1.94E-34	2.11E-32	Down
AC241584.1	2.234717901	-2.489427295	0.000136159	0.000491395	Up
CNDP1	-3.019296483	5.263372862	1.41E-07	8.89E-07	Down
FRMPD2	-3.022522702	-0.574097964	5.26E-13	6.87E-12	Down
AC097263.1	2.234605678	0.040349494	2.13E-09	1.75E-08	Up
MTURN	-3.025246154	7.835840628	1.31E-21	4.18E-20	Down
CDC6	2.234086454	3.881730361	1.37E-06	7.34E-06	Up
HLA-C	2.233510408	9.862160107	4.48E-05	0.000179053	Up
INPP5F	-3.026039286	5.403928608	1.39E-30	1.02E-28	Down
AC087273.2	2.232853982	-0.230926251	0.000790551	0.002393499	Up
HPN	-3.028567211	0.717277835	4.09E-15	6.66E-14	Down
STAB1	2.232485219	7.286150569	0.000235526	0.000808778	Up
AL355472.3	-3.03090544	-2.323217812	1.22E-07	7.79E-07	Down
IL1RAPL1	-3.03498807	-0.176935909	5.57E-12	6.50E-11	Down
PRAP1	-3.03708745	-2.659561254	5.92E-19	1.44E-17	Down
PCDHA5	-3.037760325	-0.896484664	3.89E-10	3.55E-09	Down
LOXL3	2.231629989	5.128087334	1.94E-13	2.66E-12	Up
MYL12A	2.231601915	7.095667077	8.80E-08	5.72E-07	Up
BARD1	2.230692343	4.336872315	5.01E-13	6.56E-12	Up
AFF2	-3.037804224	1.653431057	4.08E-07	2.40E-06	Down
IL15	2.230092793	1.281009964	0.000603948	0.001880705	Up

LAPTM5	2.229546827	8.867375896	4.02E-05	0.000162117	Up
TRIM21	2.229418898	4.423548148	5.32E-09	4.13E-08	Up
RAD51AP2	-3.03836845	-2.591619676	1.95E-06	1.02E-05	Down
EEF1A2	-3.038512902	7.201166718	2.74E-15	4.53E-14	Down
PTGDS	-3.040476712	7.98800076	1.22E-12	1.53E-11	Down
KCNA3	-3.041119238	-0.02667766	8.73E-13	1.11E-11	Down
LINC0032(	-3.041322289	2.408634426	1.76E-10	1.69E-09	Down
PPP1R2B	2.229353995	-2.20572399	3.95E-05	0.000159441	Up
TM6SF2	2.228827427	0.914704125	6.30E-05	0.000244742	Up
AC073578.2	-3.041455847	-3.40220853	2.23E-11	2.41E-10	Down
KRT3	-3.041834637	-3.56783642	0.000867091	0.002600913	Down
RBBP4P2	2.228770046	-0.581320266	1.17E-10	1.15E-09	Up
AC148477.3	-3.042814669	-2.795266464	7.18E-06	3.37E-05	Down
SUSD2	2.22843612	3.578602287	0.000159381	0.000566612	Up
AL512844.2	2.226491684	-2.867235499	0.000513263	0.001624112	Up
RPRML	-3.04358065	0.296592709	5.86E-11	6.00E-10	Down
NRAP	-3.044417587	-0.477351476	2.17E-13	2.95E-12	Down
HSD3BP5	-3.045252258	-2.27691538	7.35E-13	9.44E-12	Down
AL157395.1	2.225595094	-1.817518259	0.000697971	0.002139505	Up
IRS3P	2.22484074	-0.655928731	0.000698065	0.002139566	Up
TDRD5	-3.045786038	-1.676745815	1.97E-09	1.63E-08	Down
STK17B	2.222042832	5.039500049	7.51E-09	5.70E-08	Up
AL592148.1	2.220871293	-2.325141976	0.000137873	0.000496957	Up
UNC80	-3.04588922	4.324873896	2.01E-26	1.00E-24	Down
EMP1	2.220715356	8.297032338	0.000272668	0.000922902	Up
BRD9P2	-3.046353214	-3.158978074	3.70E-10	3.38E-09	Down
EHD3	-3.047194777	5.280978697	4.56E-33	4.26E-31	Down
MAGEC3	-3.048413493	-2.139706463	9.96E-10	8.58E-09	Down
CALU	2.220558978	8.272206506	1.40E-06	7.48E-06	Up
HMGB1P24	2.218508146	-2.489865817	5.95E-05	0.000232179	Up
AL121895.1	-3.049149012	-0.671292926	7.48E-12	8.55E-11	Down
LINC0245(	-3.049816146	-1.184972014	1.31E-19	3.41E-18	Down
NALCN	-3.050152379	4.055826624	2.22E-21	6.88E-20	Down
DNAJA4	-3.050760802	4.668586082	3.13E-34	3.34E-32	Down
KCNC3	-3.051185307	3.607260753	1.09E-25	5.07E-24	Down
RPS5	2.21808579	9.468174802	7.92E-06	3.68E-05	Up
UPK1B	-3.051198711	-3.548075759	4.06E-09	3.21E-08	Down
ANKRD18I	-3.055359029	-2.10539862	7.52E-07	4.22E-06	Down
PIP4K2A	-3.056546841	6.060791761	2.73E-30	1.93E-28	Down
ANKRD53	2.218067619	1.704748405	0.000388632	0.00126503	Up

AC097381.:	-3.057298154	-2.836169584	1.63E-08	1.18E-07	Down
IFI16	2.217463405	6.851542102	4.49E-06	2.19E-05	Up
ADRA2C	-3.06108075	0.22149183	1.96E-11	2.13E-10	Down
AL132822.1	-3.062001263	-3.353876516	9.00E-08	5.84E-07	Down
MMRN1	2.216509832	2.930199033	5.62E-05	0.000220214	Up
SPCS2P4	2.215649314	1.829465742	2.71E-17	5.60E-16	Up
NCF1	2.215503951	2.483670049	3.33E-05	0.000136861	Up
PNLDC1	-3.062225224	-1.468712264	1.02E-14	1.60E-13	Down
RPS7P14	2.21548239	-1.114099123	5.06E-07	2.92E-06	Up
LINC0220:	2.215273938	-0.521765665	0.000106225	0.000393313	Up
SP100	2.215115675	5.373119681	4.31E-06	2.11E-05	Up
NORD113-	-3.063195898	-2.836103433	1.81E-10	1.73E-09	Down
LCTL	2.21432406	2.361700907	0.000207169	0.000718853	Up
FTH1P16	2.212330874	1.962211574	1.73E-08	1.24E-07	Up
FGF22	-3.063646795	-1.448016178	4.63E-18	1.03E-16	Down
UCP2	2.212186818	6.155577167	4.17E-06	2.05E-05	Up
DBNDD2	-3.063723351	3.846175151	1.14E-17	2.46E-16	Down
APOBEC3I	2.211280758	2.218810653	1.07E-05	4.87E-05	Up
LDLRAD2	2.210659642	0.501615771	0.000464129	0.001483298	Up
RAPGEFL:	-3.064775061	4.125688289	4.34E-40	8.34E-38	Down
PTH2R	-3.065413572	-0.29436914	2.23E-10	2.10E-09	Down
AP001324.1	2.209029743	3.148484079	2.26E-07	1.38E-06	Up
ANKRD34/	-3.065438724	2.652709942	7.36E-22	2.40E-20	Down
CALD1	2.207659321	8.069059119	1.29E-09	1.10E-08	Up
AOX3P	-3.069773761	-3.363527293	4.32E-09	3.40E-08	Down
ACTA2	2.206359136	6.33796419	0.000495003	0.001570125	Up
APBA1	-3.070753341	4.842566659	5.86E-32	4.92E-30	Down
NCF2	2.205701452	4.295292494	1.46E-05	6.46E-05	Up
MTND4P1:	-3.071700674	4.678207262	6.94E-30	4.76E-28	Down
RPL5P11	2.204621926	-1.751697135	2.17E-06	1.12E-05	Up
AC104306.:	2.203960301	-2.667930494	0.000517455	0.001635042	Up
ALOX12B	-3.072215295	-2.011747424	4.96E-13	6.49E-12	Down
BCHE	2.201664573	6.114939925	4.05E-05	0.000163271	Up
PARM1	-3.074030556	4.343403632	3.35E-14	4.99E-13	Down
PEG3	-3.07446364	5.981051859	1.00E-19	2.64E-18	Down
FLJ42969	-3.074652726	-2.987211342	3.05E-13	4.11E-12	Down
RCAN2	-3.079920599	5.347975757	4.92E-12	5.77E-11	Down
NPM1P6	2.201032813	-0.418679946	4.03E-09	3.19E-08	Up
SQOR	2.200020765	4.124699552	4.76E-06	2.30E-05	Up
STK33	2.19993588	4.179987643	1.92E-06	1.00E-05	Up

FAM72B	2.198627986	0.521783937	5.43E-08	3.64E-07	Up
RPSA	2.198453049	9.782750529	7.56E-08	4.96E-07	Up
RAD54L	2.195348318	2.879785021	5.75E-06	2.75E-05	Up
ZFR2	-3.081552271	1.799635337	7.68E-09	5.82E-08	Down
CBX7	-3.083149683	4.836899662	2.92E-36	3.76E-34	Down
PRKAR1B	-3.084003278	6.754033056	4.04E-42	9.24E-40	Down
CES5A	-3.084122072	-2.474495687	4.30E-13	5.70E-12	Down
AL354863.1	-3.084943035	-2.276179917	4.34E-06	2.12E-05	Down
ITPR1	-3.085644001	4.869810258	1.51E-39	2.76E-37	Down
MTND6P4	2.194954445	1.645318046	2.77E-06	1.40E-05	Up
AP000941.1	-3.086848023	-0.959974888	6.93E-18	1.52E-16	Down
HPN-AS1	-3.088712176	-0.306690016	1.24E-07	7.92E-07	Down
SYNJ2	-3.093103443	4.885662288	3.89E-22	1.31E-20	Down
BTN2A2	2.193803206	4.205316734	1.30E-16	2.52E-15	Up
NCF1C	2.193643017	1.590383352	2.07E-05	8.86E-05	Up
LINC0199[illegible]	-3.094513883	-3.364982052	2.33E-06	1.20E-05	Down
LINC0055[illegible]	-3.094964302	-3.182478812	5.16E-10	4.60E-09	Down
ARHGDIB	2.193196466	7.202503496	2.33E-07	1.42E-06	Up
AC108022.[illegible]	2.192514366	-2.20607675	0.000112725	0.000414399	Up
ADAMTS9	2.192215069	5.461385421	0.000382496	0.001247171	Up
CLCN4	-3.096499362	5.109275188	8.79E-48	3.42E-45	Down
EIF4HP1	2.191571037	1.39529155	2.28E-08	1.62E-07	Up
AC010186.[illegible]	2.188298855	1.065629545	5.98E-10	5.29E-09	Up
CLSTN3	-3.097115922	6.283888988	2.05E-52	1.26E-49	Down
HAPLN3	2.187261504	3.144534289	5.62E-06	2.69E-05	Up
NELL2	-3.09753326	6.098784309	3.39E-18	7.63E-17	Down
AL157396.1	-3.09955779	-2.118448455	2.19E-10	2.06E-09	Down
SLC6A13	-3.099562693	0.745092013	1.91E-15	3.22E-14	Down
HHATL	-3.099643987	3.924138342	2.75E-11	2.94E-10	Down
PGBD5	-3.099977374	4.94078457	6.04E-32	5.05E-30	Down
PPIAP59	2.186649018	-2.377615038	0.000897822	0.002683315	Up
RBMXP2	2.184878972	-0.603399627	4.72E-09	3.70E-08	Up
ABCB4	2.183582355	2.148622668	5.81E-07	3.32E-06	Up
RAB40B	-3.100564446	4.578781841	3.72E-37	5.11E-35	Down
AL513325.1	2.182910558	-2.372251748	6.82E-06	3.21E-05	Up
GPR179	-3.10095683	0.576906442	3.55E-13	4.75E-12	Down
DPP10-AS1	-3.10172391	-0.235687659	3.93E-07	2.32E-06	Down
KIAA0895LI	-3.101765203	-3.123704513	8.92E-19	2.13E-17	Down
CACNA1C	-3.105282186	3.880176401	1.34E-19	3.48E-18	Down
GPR150	-3.105433398	-0.812844794	2.62E-15	4.35E-14	Down

TTC22	-3.107530107	-2.460612885	1.80E-14	2.74E-13	Down
LINC0155(	2.182811702	1.672063426	3.02E-06	1.52E-05	Up
RPSAP18	2.181776978	-0.110833661	1.67E-09	1.40E-08	Up
OSTC	2.179359532	6.132973755	2.48E-08	1.75E-07	Up
IFI35	2.179084095	5.495939954	4.95E-05	0.000196152	Up
TMEM125	-3.108746416	2.727119794	1.55E-07	9.73E-07	Down
MIR4500H(	-3.110140697	-1.082853998	3.30E-11	3.49E-10	Down
CHRM4	-3.114027588	0.465339117	1.76E-17	3.71E-16	Down
D21S2088E	2.179061309	0.064589937	0.000743653	0.002264836	Up
MICU3	-3.115806199	3.183900537	1.60E-54	1.21E-51	Down
FAM81A	-3.115892615	3.107318051	2.57E-24	1.06E-22	Down
SSBP3P2	-3.11610904	-3.533851507	7.30E-13	9.39E-12	Down
AL133163.3	-3.119415889	-2.954694609	9.58E-07	5.27E-06	Down
EPCAM	-3.122850776	-0.179090998	7.55E-15	1.20E-13	Down
CIR1P2	-3.124922396	-3.123924021	4.65E-12	5.47E-11	Down
RPS15A	2.178651007	8.502626213	9.08E-08	5.89E-07	Up
MS4A8	-3.125635947	-2.638012741	4.63E-13	6.10E-12	Down
AL117329.1	-3.127920644	-3.330194831	0.000948159	0.002817101	Down
BDNF	-3.127976767	2.071405885	7.23E-10	6.34E-09	Down
TPBGL	-3.129595361	0.535361828	4.58E-19	1.13E-17	Down
ABLIM2	-3.129871024	3.514653774	7.16E-33	6.61E-31	Down
OPCML	-3.129890376	5.057873834	7.23E-11	7.30E-10	Down
MGAT5B	-3.131472061	4.410593569	2.36E-20	6.57E-19	Down
ATP6V1G2	-3.131554117	5.367001637	1.11E-22	3.94E-21	Down
AC130456.1	-3.133297029	-3.063420409	2.54E-12	3.08E-11	Down
AC107398.3	-3.135233068	3.896124108	5.54E-12	6.46E-11	Down
COL26A1	-3.136570302	2.477585453	2.78E-07	1.68E-06	Down
CYTIP	2.177468462	2.660804297	0.000157417	0.000560601	Up
RAB27B	-3.137471296	3.309272183	1.45E-14	2.24E-13	Down
EFEMP2	2.177018623	6.85456383	2.56E-06	1.31E-05	Up
PRLHR	-3.138095647	-1.087426379	2.75E-07	1.66E-06	Down
CYP4F35P	-3.138496	-2.445110271	8.82E-07	4.89E-06	Down
AC007424.1	-3.138637793	-2.774631969	2.18E-13	2.96E-12	Down
ZFP57	-3.142733082	-0.252670634	2.82E-08	1.98E-07	Down
SEPTIN5	-3.143357196	6.553405014	4.02E-41	8.51E-39	Down
CYBA	2.176058262	6.448653041	1.66E-05	7.24E-05	Up
SLC22A2	-3.143437271	-2.681190093	4.43E-06	2.16E-05	Down
AC093014.1	2.172161943	-2.112199955	2.74E-05	0.000114835	Up
AL157777.1	2.172119586	-2.720441656	0.000420912	0.001358422	Up
SMIM6	-3.143720552	-1.158779419	8.24E-11	8.25E-10	Down

MNS1	2.171455197	2.998601259	2.11E-05	9.02E-05	Up
PCDHAC2	-3.1445279	1.636246599	1.35E-30	9.98E-29	Down
XK	-3.145793976	1.717448914	2.70E-14	4.05E-13	Down
FNDC3B	2.171301328	5.592582747	6.31E-08	4.20E-07	Up
RPS3AP34	2.169634082	-1.882150603	2.93E-05	0.000121795	Up
CD14	2.16938096	7.585682331	0.000916818	0.002733285	Up
AL356585.1	2.169325157	-2.920201939	0.000295129	0.000988703	Up
PPIAP72	2.169203614	-2.550018664	9.23E-05	0.000346378	Up
CALCRL	2.169099066	5.810511873	0.000720207	0.002202042	Up
T6GALNAC	-3.147435326	2.588527472	6.42E-10	5.66E-09	Down
AC066612.1	-3.150952092	-3.508402076	7.68E-16	1.36E-14	Down
NUAK1	-3.151046582	4.916092135	2.56E-49	1.19E-46	Down
PTMAP9	2.168258593	0.407457392	1.13E-10	1.11E-09	Up
CASZ1	2.167028482	1.869526056	0.000143173	0.000514452	Up
CDCA3	2.165160723	3.756159001	3.96E-08	2.71E-07	Up
HOXD1	-3.151301612	-1.378821601	6.56E-09	5.03E-08	Down
AL136126.1	2.164567154	-0.444649755	1.84E-06	9.63E-06	Up
PPIAP64	2.164240617	-2.39186566	8.58E-05	0.000323965	Up
SRRM4	-3.151719557	3.740816386	5.45E-06	2.61E-05	Down
C1QTNF6	2.163142038	4.642612569	0.000121327	0.000442859	Up
RASGEF1A	-3.151732248	3.071093634	8.85E-18	1.92E-16	Down
TNIP3	-3.151929623	-2.456252301	2.01E-09	1.66E-08	Down
SEMA4D	-3.152702824	5.549034372	4.97E-34	5.23E-32	Down
RPS27AP19	2.162678347	-2.7041779	0.000914806	0.002728698	Up
FZD2	2.161346836	3.169409084	1.91E-05	8.25E-05	Up
OVOL2	-3.15464176	-3.105245633	6.45E-07	3.66E-06	Down
SPRN	-3.155565048	2.775208702	2.72E-30	1.93E-28	Down
VDAC1P1	2.160525113	0.165277092	6.18E-10	5.46E-09	Up
SH3RF2	-3.155706894	-0.734688794	3.01E-11	3.20E-10	Down
PAK5	-3.155877644	2.23745468	2.39E-07	1.46E-06	Down
ZIC4	2.158260481	2.777387759	0.000177851	0.000626095	Up
AC007375.3	-3.157662603	-1.455123642	2.55E-13	3.45E-12	Down
CENPW	2.156999493	2.402715654	1.82E-05	7.87E-05	Up
MBP	-3.157846591	10.11808558	2.30E-08	1.63E-07	Down
ANKRD18A	-3.159419164	-1.899919019	4.05E-08	2.77E-07	Down
ABCA2	-3.160015648	7.717387496	1.76E-31	1.42E-29	Down
THRB	-3.160365105	4.207302501	1.04E-33	1.05E-31	Down
CNTNAP5	-3.160977229	0.607160949	2.33E-09	1.91E-08	Down
EDNRA	2.156374057	4.839031182	3.27E-05	0.000134503	Up
PSD3	-3.161314808	6.205582559	4.29E-52	2.38E-49	Down

CBLN1	-3.161548805	0.724741421	3.94E-07	2.32E-06	Down
SPTBN4	-3.162788385	4.483058651	2.16E-33	2.10E-31	Down
FBXL21P	-3.163546752	-2.611171927	8.04E-06	3.74E-05	Down
XKR7	-3.163578769	1.326698878	4.03E-07	2.37E-06	Down
BCLAF1P2	2.155660906	-1.122367407	1.90E-06	9.93E-06	Up
AC103563.7	-3.165689999	-2.236795765	1.59E-14	2.44E-13	Down
AC008481.1	2.155638149	-1.789780055	5.53E-06	2.64E-05	Up
MFNG	2.155201147	4.641651122	8.67E-08	5.64E-07	Up
AL365361.1	-3.167507574	1.576209995	2.59E-14	3.90E-13	Down
RRM2P3	2.154387862	-1.094112891	0.000847425	0.002548882	Up
MAP7	-3.168123973	4.454031196	1.94E-27	1.06E-25	Down
UBL5P2	2.151722619	-0.238232715	3.24E-07	1.93E-06	Up
AC008750.3	2.149324707	-2.956057736	0.000916015	0.002731738	Up
LINC01099	-3.169818361	-3.309474489	9.87E-12	1.11E-10	Down
AL591222.1	-3.171379223	-2.449385743	5.29E-19	1.30E-17	Down
TRIM17	-3.171855586	0.502483703	1.11E-20	3.19E-19	Down
AC091729.1	-3.171883173	-3.168324056	1.29E-17	2.76E-16	Down
GMNN	2.149224129	4.939437239	3.49E-08	2.41E-07	Up
KALRN	-3.17296595	5.168671279	2.09E-38	3.36E-36	Down
RPS24P8	2.148422	-0.049867272	1.48E-06	7.90E-06	Up
PTGS1	2.147681682	5.673921909	0.000568041	0.001781475	Up
AC020928.2	-3.17334138	-2.226761293	2.49E-13	3.37E-12	Down
PRPF38AP1	2.146422878	-2.928789146	0.000671659	0.002068311	Up
B3GNT4	-3.173390553	0.500819854	7.68E-33	7.07E-31	Down
OLFM1	-3.175649401	7.185121774	8.23E-17	1.62E-15	Down
FCHO1	-3.176040347	3.332181092	8.09E-32	6.63E-30	Down
DCAF12L2	-3.181417335	-1.075644094	0.000207334	0.000719341	Down
SLC25A27	-3.182571713	3.301287462	2.41E-20	6.71E-19	Down
KIAA1143P	2.140252906	-2.750522222	0.000274973	0.000930048	Up
EIF4BP3	2.139587944	1.191088007	1.38E-10	1.34E-09	Up
RHOV	-3.183154951	-1.061291468	4.86E-18	1.08E-16	Down
CDK5R1	-3.184155495	5.558396954	1.63E-21	5.14E-20	Down
CD72	2.139323835	2.133657172	0.000510357	0.001615626	Up
NLRP3P1	-3.185548363	-3.754604903	7.73E-07	4.32E-06	Down
CD63	2.138761559	10.02359853	9.78E-07	5.37E-06	Up
GRM5-AS1	-3.185674655	-3.196023897	2.65E-08	1.87E-07	Down
NPY5R	-3.186222555	-0.797271325	7.71E-07	4.31E-06	Down
HAUS8	2.138433027	3.2603722	5.28E-10	4.70E-09	Up
SFTPD-AS1	-3.187396664	-3.553305479	3.34E-11	3.53E-10	Down
KCNJ1	-3.188409202	-1.871605557	2.43E-20	6.76E-19	Down

GSDMD	2.138285048	5.338927371	3.96E-05	0.000159789	Up
AL138688.2	-3.188621925	-2.819978731	3.18E-16	5.96E-15	Down
NCF1B	2.138113791	0.909164488	0.000132262	0.000478529	Up
AC010186.2	2.137454996	2.38357054	1.82E-09	1.51E-08	Up
DIRAS2	-3.192101741	4.950601212	5.16E-16	9.37E-15	Down
IGBP1P1	2.136843521	-2.261879032	1.67E-06	8.82E-06	Up
BCL11A	-3.193515797	3.00462399	2.46E-16	4.64E-15	Down
AC025048.(	2.13684209	-0.564279205	6.08E-05	0.000236682	Up
AP003108.1	-3.19456725	-2.666847572	2.15E-11	2.33E-10	Down
NKX6-2	-3.195728207	3.538330685	1.78E-08	1.28E-07	Down
NBPF8	2.136020357	3.071158784	1.54E-07	9.67E-07	Up
PRDX3P2	2.135417886	-2.746533474	0.00045014	0.001443553	Up
AC079336.5	-3.19883767	-1.795165164	1.75E-21	5.49E-20	Down
LY6H	-3.200823749	4.453219571	1.79E-10	1.71E-09	Down
AC003986.1	-3.20216334	-3.555225766	9.59E-06	4.38E-05	Down
AC006262.1	-3.20239194	-1.159185955	1.63E-09	1.36E-08	Down
AL592114.3	-3.203839706	-3.05382332	0.000157674	0.000561357	Down
PRRT2	-3.204879578	4.626691934	2.17E-21	6.74E-20	Down
RTN1	-3.206575727	7.195673104	1.70E-18	3.96E-17	Down
EXTL1	-3.206644589	3.201846484	1.79E-20	5.04E-19	Down
IL1RL2	-3.206829466	-1.396718849	7.52E-13	9.65E-12	Down
ST13P4	2.133094009	-0.480740221	1.99E-08	1.42E-07	Up
TYMS	2.13280918	5.723775402	1.46E-05	6.46E-05	Up
OR2L13	-3.208175559	-0.716989046	9.31E-08	6.03E-07	Down
TUBA4A	-3.20900565	5.263136703	1.07E-21	3.43E-20	Down
GINS4	2.130892449	2.851316851	1.08E-06	5.89E-06	Up
TRPM6	-3.210646694	0.436293751	1.12E-19	2.94E-18	Down
DARS-AS1	2.130698035	-0.016656636	5.83E-07	3.34E-06	Up
AC036214.3	2.129330525	-1.331754404	0.000103671	0.000384748	Up
AL031587.1	-3.210843271	-2.23497027	2.68E-10	2.50E-09	Down
AC082651.1	-3.211077089	-3.027853848	7.60E-13	9.73E-12	Down
PHETA2	2.127963265	3.332312018	2.94E-05	0.000122396	Up
AC007953.2	-3.211704509	-3.727582481	3.37E-06	1.68E-05	Down
TMEM253	2.127627367	-1.273113543	0.000149372	0.000534792	Up
INAVA	2.12711503	3.066724945	0.000680483	0.002091905	Up
LINC0131(	-3.211957217	-1.807234247	4.37E-13	5.77E-12	Down
MYCBP	2.126623149	3.830771769	3.32E-11	3.51E-10	Up
STMN2	-3.213497412	6.220337711	3.14E-08	2.18E-07	Down
ACAP2-IT1	-3.217087021	-1.702200781	2.85E-27	1.53E-25	Down
RPS10	2.125890593	7.743283916	1.34E-06	7.19E-06	Up

AC005865.1	-3.21766602	-1.964891141	2.77E-13	3.74E-12	Down
AC108734.3	2.1238833	-2.253407652	0.000116715	0.000427595	Up
CLSTN2	-3.220150311	5.150314896	4.52E-15	7.33E-14	Down
AL035409.1	-3.222318265	-3.034657564	3.05E-10	2.82E-09	Down
AC099792.1	2.123744809	1.736897224	0.000583196	0.001823176	Up
PRDM8	-3.223195959	2.993639047	1.53E-12	1.89E-11	Down
LINC0260[illegible]	2.122070761	-2.148772251	0.00060481	0.001882779	Up
AC093330.2	-3.223806657	-1.627747962	2.76E-17	5.69E-16	Down
ASPDH	-3.224093638	1.653699785	6.28E-19	1.52E-17	Down
KIF5C	-3.224351076	7.041755864	4.00E-33	3.78E-31	Down
GABBR2	-3.225270658	5.728758375	1.80E-11	1.97E-10	Down
MIR124-1H[illegible]	-3.226191423	2.599859243	4.83E-09	3.77E-08	Down
MIM10L2[illegible]	-3.227651645	3.993141721	1.39E-29	9.32E-28	Down
SYN3	-3.228429753	1.393153487	2.72E-17	5.62E-16	Down
AC098826.2	-3.22950404	-3.522657323	2.49E-07	1.51E-06	Down
TAGLN3	-3.231561682	5.758566055	3.47E-14	5.16E-13	Down
HNRNPA1P	2.121788217	-2.564466918	0.000586886	0.001833355	Up
GYPC	2.12140609	5.228048932	0.000210196	0.000728568	Up
HTR1B	-3.234186251	-2.164740814	5.67E-16	1.02E-14	Down
WNT5A	2.121040264	5.132006734	7.11E-06	3.34E-05	Up
HHIP	-3.234715197	3.390671211	2.82E-10	2.62E-09	Down
ST18	-3.235090891	3.042182773	9.97E-12	1.12E-10	Down
FER1L6	-3.237455509	-2.221982225	6.83E-11	6.94E-10	Down
TYMP	2.119527311	5.413447096	0.000264846	0.000898748	Up
CEACAM[illegible]	-3.238206443	-3.172591334	2.41E-05	0.000102031	Down
TNFSF8	2.118954192	1.931721116	0.00051801	0.001636437	Up
RPS26P3	2.117706958	-1.087734369	0.000291281	0.000977518	Up
RTN4RL2	-3.241637088	3.08584289	1.19E-18	2.81E-17	Down
GLRA3	-3.241809138	1.98138496	1.04E-05	4.74E-05	Down
TENM2	-3.242588842	3.55856625	1.97E-09	1.63E-08	Down
SLITRK1	-3.242627282	3.492948663	2.05E-09	1.69E-08	Down
MTND1P2[illegible]	-3.244267413	4.381837126	4.66E-07	2.71E-06	Down
KCNH8	-3.24452259	2.622388608	2.59E-11	2.77E-10	Down
AC145285.1	2.11632705	-1.65556744	1.17E-06	6.33E-06	Up
PAK1	-3.245667171	5.98749933	4.10E-66	1.31E-62	Down
CALB1	-3.245877243	2.143687975	1.46E-10	1.41E-09	Down
CNTN6	-3.246555811	0.30915609	2.30E-19	5.82E-18	Down
AC138305.3	2.115191716	-1.945936518	2.20E-05	9.36E-05	Up
DPRXP2	-3.247163114	-3.709772284	6.45E-12	7.43E-11	Down
AL592430.1	-3.247565258	-3.277479057	2.43E-15	4.06E-14	Down

KIF5A	-3.24768409	7.657177167	6.19E-12	7.16E-11	Down
AC012508.1	-3.249571983	-2.232499525	3.24E-18	7.30E-17	Down
AL391845.2	-3.249642966	-1.49000228	4.47E-14	6.56E-13	Down
LRGUK	2.114880694	0.838623138	0.000107601	0.000397896	Up
AP000763.2	2.113672864	0.562222387	0.00014036	0.000505353	Up
TUBBP5	-3.251338181	-2.27360589	2.55E-17	5.27E-16	Down
CELF3	-3.25315766	4.558351889	3.49E-11	3.69E-10	Down
CBLN4	-3.253226802	2.699668485	1.02E-08	7.59E-08	Down
AC060766.1	2.113526831	-0.412873856	0.000258642	0.00088039	Up
THEM5	-3.253852231	0.191373174	3.97E-16	7.34E-15	Down
AC243829.1	-3.255036053	-2.584436995	2.08E-09	1.72E-08	Down
MYC	2.113297197	5.601975931	1.62E-05	7.10E-05	Up
FBXO41	-3.256000022	4.930700356	8.54E-34	8.76E-32	Down
AL121749.1	-3.256893271	-2.783950668	3.87E-09	3.07E-08	Down
LINC01792	-3.260448173	-1.198027744	6.40E-09	4.91E-08	Down
STAT4	-3.260884348	0.972329235	6.14E-19	1.49E-17	Down
AC015540.1	-3.26580596	-1.613146297	9.23E-12	1.04E-10	Down
MCUB	2.112420336	5.448228316	1.97E-05	8.48E-05	Up
ATP2A1-AS	2.112366961	0.687087832	8.81E-06	4.06E-05	Up
PLEKHH1	-3.269000652	5.445991554	2.78E-23	1.04E-21	Down
SEPTIN4	-3.269091016	6.506339493	3.97E-25	1.77E-23	Down
FBXO2	-3.269975318	4.794321236	2.27E-17	4.73E-16	Down
NOS1	-3.26999155	2.122668781	5.67E-09	4.38E-08	Down
TRIM22	2.112013944	6.297246053	0.000130682	0.000473526	Up
C1RL	2.110457888	5.397437735	0.00013992	0.000503895	Up
C2CD4C	-3.270107914	2.814455003	4.66E-23	1.71E-21	Down
MYADML2	-3.270523444	-0.520949678	4.11E-28	2.36E-26	Down
AC134312.6	-3.270959852	-3.564966643	2.21E-09	1.82E-08	Down
CACNB1	-3.271594952	4.592906209	4.48E-45	1.37E-42	Down
HROB	2.109364348	2.287991144	6.90E-09	5.26E-08	Up
CLCA4	-3.272358976	0.402692252	5.28E-08	3.54E-07	Down
AC110998.1	2.109162215	-0.914681526	0.00016742	0.000592124	Up
TRIP6	2.109047621	6.790736912	1.70E-07	1.06E-06	Up
KIF1A	-3.272371165	8.252526401	3.52E-22	1.19E-20	Down
CBFA2T3	-3.274165308	1.565035727	2.65E-27	1.43E-25	Down
DSG1	-3.275133829	-3.577274489	2.48E-09	2.02E-08	Down
PABPC3	2.109020916	1.395184226	2.08E-09	1.71E-08	Up
CYP46A1	-3.27620451	4.403951495	1.37E-29	9.21E-28	Down
AC106881.1	-3.276947131	0.622745193	6.98E-14	1.00E-12	Down
ANO4	-3.28048823	2.057657496	1.00E-13	1.42E-12	Down

RPS2P32	2.106115607	1.501836264	9.43E-05	0.000353414	Up
AC091729.2	-3.280777585	-3.286639934	4.68E-16	8.56E-15	Down
NPY1R	-3.281000776	1.122563892	3.89E-11	4.09E-10	Down
DNM3	-3.282994354	4.822326042	1.71E-24	7.20E-23	Down
AC233699.1	2.10603731	-1.885124438	6.06E-05	0.000236049	Up
LINC0115(	2.105842354	-0.512890458	0.000423506	0.00136559	Up
AC103923.1	2.103289624	0.473254982	2.72E-05	0.000114116	Up
GAD1	-3.286494795	4.895541788	7.58E-16	1.35E-14	Down
KIAA1549I	-3.287102242	5.038869649	3.66E-38	5.70E-36	Down
AP000781.1	2.103200979	-2.177460923	0.000218021	0.000752703	Up
MAPK8IP2	-3.289436346	5.707360909	6.58E-28	3.71E-26	Down
TLE6	2.102681614	1.88263987	0.000518367	0.001637205	Up
NCS1	-3.290934577	7.049187635	8.29E-44	2.21E-41	Down
NKRD36B	-3.291511347	-0.83670369	1.20E-08	8.86E-08	Down
AL157871.3	2.102393597	-2.169728497	6.61E-05	0.000255874	Up
NRNPA1P	2.098601901	-2.766486948	0.000688066	0.002112062	Up
AC140847.2	-3.292557463	-3.219457789	6.53E-09	5.01E-08	Down
PPFIA3	-3.294912093	4.475021935	5.13E-43	1.27E-40	Down
SMYD1	-3.295064081	-1.065123263	2.86E-13	3.85E-12	Down
TGFBR3L	-3.295392818	0.170208267	9.07E-17	1.78E-15	Down
AC090912.1	2.097629764	-2.444603405	0.000630407	0.001953807	Up
TUBB4A	-3.296969057	7.739196542	2.44E-12	2.96E-11	Down
OPRD1	-3.297796095	1.727234905	1.08E-15	1.88E-14	Down
CDCA5	2.096947247	4.697431668	7.69E-06	3.58E-05	Up
LINC0092[illegible]	-3.298070293	-3.131227653	3.54E-09	2.82E-08	Down
PTPN3	-3.299285733	2.274791552	4.14E-12	4.90E-11	Down
AC084809.2	-3.299553255	-3.728895585	4.55E-08	3.09E-07	Down
FIRRE	2.09472565	0.769151168	5.53E-05	0.000217066	Up
CHRNB2	-3.302036457	3.802755785	1.73E-17	3.64E-16	Down
RESP18	-3.303299605	-2.728181066	1.27E-07	8.05E-07	Down
AC080128.1	-3.30503637	-2.302292431	1.59E-10	1.53E-09	Down
TES	2.093309162	3.273884799	6.03E-07	3.44E-06	Up
AP000866.5	-3.305602219	-3.269564315	7.31E-13	9.40E-12	Down
CARD6	2.093285534	3.243863809	3.63E-05	0.000147933	Up
HSPE1P3	2.092626409	-1.097311584	1.16E-08	8.54E-08	Up
MYRF	-3.306112473	6.101902102	2.62E-12	3.17E-11	Down
CD33	2.092321089	3.241305501	0.000158409	0.000563438	Up
TSPOAP1	-3.306939088	5.044148736	2.78E-23	1.04E-21	Down
VDAC1P6	2.091341614	-2.445753287	0.000118495	0.000433345	Up
AC138811.2	2.090406833	-1.213608968	0.000265592	0.000900962	Up

LINC0148	-3.308322878	-3.076968725	1.18E-13	1.66E-12	Down
NPY	-3.309043242	2.911801335	5.11E-10	4.56E-09	Down
ITPKA	-3.310764833	3.17925559	7.73E-31	5.83E-29	Down
KCNN1	-3.312819893	3.223433017	2.38E-21	7.34E-20	Down
RPS19	2.089525567	9.506230078	3.75E-07	2.22E-06	Up
GSTM2P1	2.089502447	-2.301316178	0.000579555	0.001813367	Up
RPL12P14	2.087545921	-1.7482788	5.92E-06	2.81E-05	Up
AC021074.2	-3.313821937	-3.770708082	3.23E-08	2.25E-07	Down
KCNC1	-3.315303558	3.796423355	5.64E-23	2.04E-21	Down
PPM1H	-3.319886453	3.988738792	1.43E-36	1.89E-34	Down
ASGRF2-A	-3.320833648	-2.473102947	1.25E-16	2.43E-15	Down
CHRM3	-3.322371481	3.585438051	4.82E-14	7.05E-13	Down
AC009560.1	2.08648417	-1.755813458	0.000701072	0.002147637	Up
SMAGP	2.085179824	2.238120852	0.000847445	0.002548882	Up
SGIP1	-3.32296126	3.940479644	4.65E-40	8.87E-38	Down
AC138028.1	-3.322981385	-2.60713281	3.21E-19	8.02E-18	Down
DOCK3	-3.323407074	4.873326278	2.03E-27	1.11E-25	Down
ANKRD33I	-3.327875538	2.748633522	6.06E-31	4.61E-29	Down
RPL22L1	2.084041108	4.810825886	0.000660612	0.002037341	Up
AC020765.2	2.083985753	-0.162980934	0.00040539	0.001313495	Up
PCP4L1	-3.329458749	2.132117516	2.24E-11	2.42E-10	Down
LONRF2	-3.330495676	5.751909055	5.33E-31	4.07E-29	Down
STUM	-3.33102658	4.848775409	3.35E-19	8.35E-18	Down
SETP3	2.082991311	-2.453843056	0.00011708	0.000428767	Up
AL139352.1	-3.332830923	-1.080371775	9.37E-19	2.23E-17	Down
FIBIN	2.082296139	6.478022104	1.57E-07	9.83E-07	Up
LORICRIN	-3.334989102	-3.579358813	3.35E-12	4.01E-11	Down
ZNF365	-3.335365784	4.325755326	5.34E-31	4.07E-29	Down
JPH4	-3.336312845	5.445991429	1.39E-16	2.68E-15	Down
BEX5	-3.336469063	3.169142674	6.55E-17	1.30E-15	Down
PRELID1P	2.081223411	-2.454396413	0.000146206	0.000524435	Up
CDH7	-3.338484064	0.435344835	3.16E-08	2.20E-07	Down
PPIAP34	2.080640454	-2.050070291	2.01E-05	8.65E-05	Up
HSP90AA2I	2.080306023	2.013458562	1.11E-08	8.20E-08	Up
NRG3	-3.340195676	2.69769817	1.44E-15	2.47E-14	Down
INSYN2B	-3.34283494	0.240717431	8.18E-15	1.29E-13	Down
CLDN16	-3.343463356	-2.967480121	1.27E-17	2.72E-16	Down
RAPGEF5	-3.344648454	5.240313571	2.12E-28	1.25E-26	Down
CALML3	-3.346747865	-3.885674265	3.83E-05	0.000155232	Down
DLGAP1	-3.347710457	5.178637745	2.83E-23	1.06E-21	Down

LINC00567	-3.348086845	-3.07680729	3.40E-18	7.63E-17	Down
ETV2	2.079706255	0.463366731	1.24E-05	5.58E-05	Up
ERP29P1	2.078439508	-1.353569952	2.41E-07	1.47E-06	Up
TMEM233	-3.348788091	1.59368562	2.48E-13	3.36E-12	Down
NRSN1	-3.349111471	4.033884716	7.98E-18	1.74E-16	Down
DRGX	-3.350277819	-2.576210205	4.97E-08	3.35E-07	Down
UBE2QL1	-3.351424037	4.074505352	2.82E-15	4.66E-14	Down
SDAD1P2	2.07809107	-2.970173903	0.000544077	0.001711093	Up
CNKSR2	-3.354520366	4.294015936	2.93E-44	8.32E-42	Down
NRNPA3I	2.076721133	1.362662554	7.17E-10	6.28E-09	Up
MAP1A	-3.355294644	7.945040889	7.96E-46	2.67E-43	Down
GJC2	-3.356307356	2.786109621	1.88E-18	4.34E-17	Down
PDIA2	-3.356850747	2.4550864	2.25E-14	3.40E-13	Down
YBX3	2.076547469	6.577575637	4.67E-07	2.71E-06	Up
KLK10	-3.35816742	-2.039277671	1.22E-14	1.89E-13	Down
CALB2	-3.361159177	2.911377653	5.21E-09	4.05E-08	Down
ERICH3	-3.361539522	3.364912913	3.54E-14	5.26E-13	Down
ADCY5	-3.363560761	4.400357132	1.44E-22	5.05E-21	Down
APGEF4-A	-3.364347798	-1.774964366	6.38E-20	1.71E-18	Down
IR4435-2H	2.074122728	5.23557243	0.000214671	0.000742651	Up
GYG2P1	-3.365105662	-2.746753425	4.67E-06	2.26E-05	Down
AIFM3	-3.366025602	3.252058466	5.69E-12	6.62E-11	Down
AC136632.1	2.073337242	3.266592251	3.09E-12	3.71E-11	Up
AC063926.3	-3.367959371	-3.323188519	6.25E-19	1.52E-17	Down
RAC2	2.070793291	4.15782671	5.48E-05	0.000215185	Up
ATRAID	2.070420452	7.214895807	2.66E-09	2.16E-08	Up
MOBP	-3.368484879	5.904186571	1.58E-08	1.15E-07	Down
MTND6P2	2.069573227	-1.056698209	0.000247905	0.000847448	Up
AC012593.1	-3.370007983	-2.801442803	5.70E-09	4.39E-08	Down
AC019171.1	-3.37024637	-1.465011388	1.39E-21	4.41E-20	Down
NPFFR1	-3.372002169	-0.711362184	6.23E-14	9.00E-13	Down
GPRASP1	-3.372070164	5.200378367	7.68E-45	2.30E-42	Down
PLOD2	2.068980719	6.702385868	1.90E-05	8.21E-05	Up
CORO6	-3.372304891	1.664756922	4.41E-21	1.32E-19	Down
FXYD7	-3.373037254	3.259055929	2.42E-11	2.60E-10	Down
C1QL2	-3.373917656	1.087795568	2.12E-09	1.74E-08	Down
EDIL3	-3.376132909	5.91558392	3.20E-16	5.99E-15	Down
OR2W3	-3.38507253	-2.039578339	1.97E-17	4.13E-16	Down
ABCC12	-3.38587764	-0.844762771	3.24E-08	2.25E-07	Down
RTL4	-3.38968082	-3.673805148	5.53E-08	3.70E-07	Down

TNFAIP8	2.068652132	2.877757027	0.000102464	0.000380759	Up
MACROD2	-3.390467101	2.05737307	2.76E-17	5.69E-16	Down
AC021613.1	-3.392739893	-2.187494136	5.70E-07	3.27E-06	Down
AC002351.1	-3.393584594	-1.205353305	1.03E-05	4.67E-05	Down
CEP170B	-3.394004991	5.127902658	5.15E-41	1.07E-38	Down
RPSAP69	-3.39601657	-1.178311883	1.74E-41	3.83E-39	Down
ENTPD3	-3.396271941	1.434744341	6.52E-14	9.41E-13	Down
RAB6C-AS1	-3.396859653	-0.805264919	8.24E-10	7.16E-09	Down
AACSP1	-3.396952301	-3.05708884	7.66E-14	1.10E-12	Down
BEND4	-3.399178038	-0.138884802	8.87E-08	5.76E-07	Down
BICDL1	-3.402089874	1.932831003	4.63E-18	1.03E-16	Down
ADAMTS7P	-3.402189481	-1.543042491	1.48E-21	4.69E-20	Down
STXBP5	-3.406060601	3.650972517	3.01E-52	1.70E-49	Down
SLCO1A2	-3.406956177	4.099171928	7.23E-16	1.29E-14	Down
OR2S1P	-3.406994721	-3.356187144	1.30E-12	1.62E-11	Down
AC020907.5	-3.409151512	-1.879443737	5.54E-16	1.00E-14	Down
ASPA	-3.411120116	3.505303492	5.36E-18	1.18E-16	Down
CASC18	-3.411394623	-1.320311652	3.91E-15	6.38E-14	Down
RAB1AP1	2.067222683	-2.96854301	0.000740116	0.002255255	Up
PDZD7	-3.412904826	1.404568112	9.23E-34	9.43E-32	Down
RPS18	2.064188903	10.1888759	2.02E-05	8.69E-05	Up
RAP1GAP2	-3.413281511	4.646719049	1.11E-23	4.37E-22	Down
PWWP3B	-3.415685037	1.108713864	5.76E-11	5.91E-10	Down
NAP1L1P3	2.063561817	-1.52689323	3.11E-06	1.56E-05	Up
CHRNA4	-3.4166895	2.58665401	1.00E-10	9.94E-10	Down
NOS1AP	-3.418508065	3.074711584	1.07E-36	1.43E-34	Down
C17orf107	-3.420833795	0.099183789	7.95E-48	3.14E-45	Down
AC007950.2	2.060107896	-2.127855768	0.000439635	0.001412696	Up
SLC38A8	-3.422008028	-2.107399342	1.55E-16	2.97E-15	Down
AC067956.1	-3.424662802	-3.391496373	4.79E-09	3.75E-08	Down
CYCSP45	2.058935128	-1.799474155	6.10E-05	0.000237473	Up
CHN1	-3.427375832	6.909021865	1.12E-46	4.09E-44	Down
OTOF	-3.428409734	0.500069142	7.56E-17	1.49E-15	Down
ARFGEF3	-3.428763579	4.866870399	8.03E-27	4.14E-25	Down
AL355578.1	-3.430466921	-2.78124635	1.52E-11	1.68E-10	Down
LYZL4	-3.431057312	-3.410722806	5.35E-09	4.15E-08	Down
TINCR	-3.431950668	1.446454477	3.34E-27	1.78E-25	Down
TEAD3	2.058448889	3.960126819	3.54E-05	0.000144568	Up
MAML2	2.056353753	5.81382443	8.20E-10	7.13E-09	Up
HNRNPA1P	2.053482794	-2.806008908	0.000318553	0.001058073	Up

AC011767.1	-3.432805684	-2.18599782	9.84E-12	1.11E-10	Down
SSTR2	-3.44011598	3.532169876	1.84E-19	4.70E-18	Down
S1PR5	-3.440240591	2.451231281	2.50E-18	5.69E-17	Down
CACYBPP2	2.053027523	0.202068098	2.91E-08	2.04E-07	Up
AC016717.2	-3.440548034	0.001907101	2.83E-10	2.63E-09	Down
PHLDA1	2.052646891	7.860591032	8.40E-06	3.89E-05	Up
SLCO4C1	-3.44115696	-2.899376676	4.85E-16	8.86E-15	Down
PRR18	-3.445377883	3.70617641	7.07E-26	3.35E-24	Down
PRSS3	-3.447838172	1.581817679	1.11E-19	2.91E-18	Down
AL162574.1	-3.449109273	-3.453183811	4.35E-11	4.55E-10	Down
CAMK2B	-3.449148497	6.011296161	5.25E-20	1.42E-18	Down
ROMO1	2.052508064	6.380286751	1.57E-06	8.37E-06	Up
LINC0029(	-3.451240555	-3.430756063	7.93E-08	5.19E-07	Down
RPL7AP14	2.051587476	-2.096238072	1.21E-05	5.45E-05	Up
RNF135	2.049528742	4.462706527	6.69E-09	5.11E-08	Up
AC015712.2	-3.45289319	0.631017756	5.38E-14	7.82E-13	Down
AC011504.1	-3.453242803	-0.57870613	6.12E-13	7.92E-12	Down
PGAP4	-3.455132548	4.090907	1.49E-15	2.56E-14	Down
AC007326.1	-3.455243047	-3.029450255	6.98E-16	1.25E-14	Down
RHO	-3.455810173	-2.367012463	2.37E-18	5.41E-17	Down
SLC22A6	-3.456262004	-0.706241427	1.73E-10	1.65E-09	Down
KCNQ3	-3.45727107	4.443159634	1.24E-37	1.80E-35	Down
HFE	2.049104465	3.707420513	6.37E-05	0.000247381	Up
LINC0125(	-3.458458035	-3.178024513	3.42E-14	5.09E-13	Down
DOC2B	-3.459064259	3.102854427	1.35E-15	2.33E-14	Down
ERI1	2.048528318	4.931039041	4.75E-10	4.26E-09	Up
RUNDC3A	-3.46109485	5.758129055	2.09E-21	6.52E-20	Down
SPOCK3	-3.461261715	4.536140817	3.20E-12	3.84E-11	Down
ANKS1B	-3.466339157	4.556411275	3.94E-20	1.08E-18	Down
KIRREL3	-3.466769191	3.688419448	1.90E-19	4.86E-18	Down
RPL14P3	2.048166528	-0.737502172	1.93E-06	1.00E-05	Up
AL355472.2	-3.469975305	-3.543600488	6.18E-07	3.52E-06	Down
MTMR7	-3.472311926	2.272469888	1.91E-34	2.09E-32	Down
NACAP8	2.047886556	-2.621847849	0.000460816	0.001474344	Up
DYNC1I1	-3.472788041	4.416687971	1.46E-14	2.25E-13	Down
LINC0247(	-3.472983308	-3.462090312	2.15E-07	1.32E-06	Down
FGD5P1	-3.474629912	-3.272299396	3.72E-16	6.91E-15	Down
FXYD4	-3.475876345	-2.660962599	6.01E-09	4.62E-08	Down
AC097639.1	-3.475902789	-1.058252304	1.57E-23	6.04E-22	Down
ABCA1	2.047614715	6.843848125	4.67E-06	2.26E-05	Up

STAR	-3.476272327	-0.275928443	2.34E-23	8.78E-22	Down
WNK2	-3.476523329	4.232600423	1.04E-13	1.47E-12	Down
NECAB1	-3.478906055	4.561885533	2.71E-39	4.88E-37	Down
MARCHF4	-3.479615693	1.956239651	1.53E-14	2.36E-13	Down
INRNPCP	2.046777702	-0.239577383	5.72E-07	3.27E-06	Up
SRP72P2	2.046664938	-2.085268732	0.000320837	0.001064923	Up
CORT	-3.48429233	-0.157379251	2.20E-25	1.00E-23	Down
CAMK1D	-3.485109562	5.554242765	3.63E-65	9.50E-62	Down
ENPP2	-3.486909126	5.845884056	1.72E-16	3.29E-15	Down
MCF2	-3.489306649	0.76259627	5.41E-14	7.86E-13	Down
NPHS1	-3.49053287	-2.277114814	2.26E-30	1.63E-28	Down
AC104260.2	-3.49059156	-2.327610735	6.36E-17	1.27E-15	Down
GPR68	-3.492618716	0.997894464	1.79E-22	6.23E-21	Down
NRNPA3P	2.04646846	-1.33075695	5.01E-07	2.89E-06	Up
RPL31P2	2.045856584	-1.984005454	0.000406206	0.001315844	Up
KCNQ5	-3.495865908	3.286389022	4.29E-16	7.89E-15	Down
BLM	2.045755402	4.63835601	2.06E-05	8.81E-05	Up
ATP6V0E1	2.044513488	7.562684237	3.15E-09	2.53E-08	Up
KCNAB1	-3.498386632	3.147221859	4.98E-47	1.84E-44	Down
AL162311.1	-3.499024797	-3.16577839	2.23E-18	5.10E-17	Down
L1CAM	-3.500641743	5.092129414	6.05E-12	7.01E-11	Down
PRC1	2.043600004	5.599645019	2.96E-06	1.49E-05	Up
GAPDHP4	2.043198838	-2.799926265	0.00084147	0.002534613	Up
RFPL2	-3.502144804	-1.034661501	4.39E-17	8.89E-16	Down
BNIP5	-3.502860112	-3.651172612	5.21E-13	6.81E-12	Down
ARPP21	-3.502984404	4.073188437	7.87E-24	3.13E-22	Down
C3orf80	-3.503935588	1.27902171	2.50E-21	7.68E-20	Down
AC008750.1	2.042515441	-0.986101556	0.000772727	0.002345203	Up
AL158166.2	2.041687231	-1.090618018	0.000102458	0.000380759	Up
GJD4	-3.50498138	-2.772502746	9.90E-15	1.55E-13	Down
RBM11	-3.506322457	-0.162452433	1.21E-13	1.70E-12	Down
LINC01771	-3.506851475	-2.955150193	4.60E-20	1.25E-18	Down
HTR4	-3.506933146	-0.956143797	5.98E-13	7.76E-12	Down
H3P13	2.041681691	-2.810266671	0.000558497	0.001753764	Up
HMGCLL1	-3.513157538	1.233879874	6.98E-13	8.99E-12	Down
BHD17AP	2.041620767	-1.45832855	5.71E-07	3.27E-06	Up
KIRREL1	2.040953462	5.471768032	5.05E-05	0.00019963	Up
ADGRL4	2.038858526	4.70096823	9.72E-07	5.34E-06	Up
ELAVL2	-3.514475136	3.20662687	2.34E-12	2.84E-11	Down
AC145207.1	-3.515281774	-2.801327991	2.05E-21	6.38E-20	Down

FKBP1C	2.03852486	1.518200896	2.83E-09	2.28E-08	Up
FAM201A	-3.521995291	-0.678106858	1.07E-12	1.35E-11	Down
P2RX2	-3.52716613	-1.697369368	3.30E-10	3.03E-09	Down
TMPOP2	2.037672164	-1.690635697	0.000561628	0.001762828	Up
NRNPA1P	2.037259403	-2.490121849	0.000177128	0.000623932	Up
AC063952.2	2.036466151	-1.798911136	6.44E-06	3.04E-05	Up
RPS7	2.036125798	8.988760526	2.79E-06	1.41E-05	Up
VSTM2B	-3.529132364	2.862036578	3.12E-11	3.31E-10	Down
AC098583.1	2.03401739	-1.048501485	1.70E-05	7.40E-05	Up
CRHR1	-3.529643362	1.159419753	9.36E-18	2.03E-16	Down
TCF7L1	2.0340124	4.941756889	5.75E-06	2.75E-05	Up
AC008708.2	-3.529827098	-2.311157	4.48E-08	3.04E-07	Down
PCSK2	-3.53142414	3.97764782	7.42E-10	6.50E-09	Down
MCF2L2	-3.532686339	2.547649464	6.09E-23	2.20E-21	Down
ATRNL1	-3.532732584	4.240487047	4.15E-20	1.13E-18	Down
MICAL2	-3.533812415	4.974710161	6.65E-30	4.58E-28	Down
TAFA4	-3.535826539	-0.882574839	8.84E-15	1.39E-13	Down
AL133387.1	-3.538902123	-3.598103125	1.15E-08	8.52E-08	Down
AC067969.1	-3.539125118	-2.853682145	1.24E-10	1.21E-09	Down
MAG	-3.539783872	6.316453523	1.67E-10	1.60E-09	Down
ZYX	2.033674493	8.57648142	6.00E-06	2.85E-05	Up
ZNF93	2.033426659	3.027546989	1.05E-07	6.72E-07	Up
LINC0263(	-3.541247991	-3.164344341	1.68E-10	1.61E-09	Down
OGDHL	-3.541509787	3.571198395	2.55E-18	5.79E-17	Down
LINC00621	-3.542068635	-2.59764537	7.33E-15	1.16E-13	Down
NDST3	-3.544584834	0.69343717	6.96E-09	5.30E-08	Down
KNDC1	-3.54475973	4.747019013	1.18E-24	5.03E-23	Down
SLC9A2	-3.545307046	-0.89875856	6.98E-14	1.00E-12	Down
RSPO3	-3.54576303	-0.027748156	4.67E-10	4.20E-09	Down
STIL	2.033109792	3.565031971	1.00E-07	6.48E-07	Up
ELFN2	-3.546649434	4.392869266	1.04E-11	1.17E-10	Down
RALYL	-3.548542677	2.728528764	3.96E-14	5.82E-13	Down
GRIP1	-3.548651514	0.311227481	3.69E-13	4.92E-12	Down
BTN3A3	2.031726792	5.192763217	1.47E-06	7.86E-06	Up
AMPH	-3.551129752	4.822954652	7.43E-27	3.84E-25	Down
KCNB1	-3.559618721	4.375720172	1.11E-18	2.63E-17	Down
ALOXE3	-3.560371379	-1.377078537	1.05E-19	2.77E-18	Down
AC066612.2	-3.562801872	-3.603630356	1.12E-09	9.64E-09	Down
ADCY1	-3.564253257	5.187205256	4.33E-23	1.59E-21	Down
DTX3L	2.031680251	5.84580145	1.05E-06	5.73E-06	Up

\C023301.:	-3.564505202	2.089416243	1.04E-10	1.02E-09	Down
\C008915.:	2.031558759	1.041351255	2.18E-07	1.33E-06	Up
ATP2B2	-3.565112184	5.630419725	1.15E-28	6.95E-27	Down
RTN4R	-3.566714283	2.97402876	6.19E-29	3.88E-27	Down
\C016597.:	-3.568891304	-0.324319791	1.17E-23	4.57E-22	Down
3TM2A-OT	-3.569646755	-0.661402257	1.54E-06	8.21E-06	Down
TXNP4	2.030600822	-2.496669648	0.000728354	0.002224118	Up
RNU6-353I	-3.56982797	-3.421285198	1.34E-12	1.66E-11	Down
PRKCE	-3.572118843	4.473693313	3.29E-70	3.16E-66	Down
RNF7P1	2.029946326	-2.638900685	0.000534871	0.001685079	Up
PRRT1	-3.572225614	4.080148089	2.85E-62	5.86E-59	Down
\L121835.2	-3.574022385	-3.280420803	9.74E-17	1.91E-15	Down
LINC0226:	-3.575025169	-3.451991046	6.29E-06	2.98E-05	Down
CLK3P2	-3.580636559	-3.152995888	9.37E-09	7.01E-08	Down
CLVS2	-3.581373371	2.99073665	2.94E-15	4.85E-14	Down
\L390726.4	-3.582075969	-1.283530772	1.03E-10	1.02E-09	Down
FA2H	-3.583944811	3.868688768	4.66E-13	6.13E-12	Down
LINC0101(	-3.590956891	-2.15572996	1.11E-15	1.94E-14	Down
CDK5R2	-3.597610883	3.72215249	1.71E-16	3.28E-15	Down
BRD7P2	2.029766743	0.034422975	1.46E-08	1.06E-07	Up
VIPR1-AS1	-3.599318115	-2.531402869	2.99E-22	1.02E-20	Down
\C138649.:	-3.601059444	0.349980007	5.11E-15	8.25E-14	Down
PRKCZ	-3.601654622	4.497643624	5.96E-24	2.40E-22	Down
OTUD7A	-3.602388723	3.257353853	2.84E-36	3.67E-34	Down
TCEAL6	-3.602858195	2.73987158	1.25E-14	1.94E-13	Down
THEMIS	-3.60342585	-0.42060393	1.27E-23	4.95E-22	Down
\P003119.2	2.029542089	-1.346554907	0.000285039	0.00095959	Up
PRRG3	-3.603669439	1.755841531	6.53E-12	7.52E-11	Down
FXYD5	2.029402284	5.335060133	1.03E-05	4.68E-05	Up
LINC01361	-3.6038359	-2.440110644	1.48E-09	1.24E-08	Down
\L353658.1	-3.603898587	-3.541216079	1.88E-11	2.05E-10	Down
ZCCHC12	-3.609855882	3.255799978	6.69E-19	1.62E-17	Down
KCNJ6	-3.612044571	2.342050119	2.68E-16	5.06E-15	Down
MAPT-IT1	-3.61286267	-0.775457585	8.34E-18	1.81E-16	Down
\C018685.2	-3.614343707	-3.77825802	2.62E-12	3.17E-11	Down
KCNK9	-3.61627887	1.140389676	5.83E-12	6.77E-11	Down
SLC6A20	-3.617773854	-0.337215883	8.89E-11	8.86E-10	Down
NSG2	-3.61802077	5.992592348	5.09E-11	5.26E-10	Down
CA11	-3.61803965	5.960584981	2.19E-33	2.12E-31	Down
AKAIN1	-3.621216395	-3.120245281	5.95E-11	6.08E-10	Down

AL023284.4	-3.622597503	-0.126325168	2.43E-28	1.43E-26	Down
MIR7-3HG	-3.62315906	0.623003637	8.94E-12	1.01E-10	Down
SERPIND1	-3.623993867	-0.868387259	1.37E-09	1.16E-08	Down
SEMA4A	-3.624206331	3.465793204	2.73E-52	1.57E-49	Down
FAIM2	-3.626217006	7.427697019	1.29E-43	3.36E-41	Down
STRCP1	-3.627054016	-1.570418655	4.21E-22	1.40E-20	Down
AC104129.1	-3.629858538	-3.065863249	2.37E-12	2.88E-11	Down
TYRP1	-3.629918064	-1.254991619	4.12E-11	4.32E-10	Down
ARHGEF19	2.026113021	2.005249479	0.000224749	0.000773982	Up
MYH7B	-3.636904865	1.55868234	1.29E-30	9.56E-29	Down
AC010931.1	-3.638182127	-0.604534784	8.10E-11	8.12E-10	Down
CYP26B1	-3.638244232	2.697725502	1.64E-18	3.82E-17	Down
BHMT	-3.639238967	-2.20106641	7.28E-16	1.30E-14	Down
SLC27A2	-3.639816307	-0.998000905	1.63E-15	2.78E-14	Down
MEM108-A	-3.641949559	-2.80380956	3.56E-12	4.25E-11	Down
SNX18P3	-3.642515264	-3.255846397	1.58E-13	2.18E-12	Down
PDE1B	-3.644833055	3.723967312	1.28E-35	1.53E-33	Down
NRIP3	-3.645459644	3.571885916	2.29E-29	1.48E-27	Down
ABCG4	-3.646316911	1.417567651	5.66E-30	3.92E-28	Down
PTPRN	-3.646822707	5.898339463	2.49E-16	4.71E-15	Down
SYNGR1	-3.650641886	6.189267358	1.85E-60	2.67E-57	Down
DARB2-AS	-3.655563248	-1.816219687	3.73E-07	2.20E-06	Down
CYP51A1P2	2.025989845	-0.475544952	1.35E-06	7.25E-06	Up
EXD1	-3.655608695	-2.998471183	2.31E-28	1.36E-26	Down
GPR62	-3.657290484	1.663409513	3.71E-16	6.90E-15	Down
AC022893.2	-3.661260452	-1.424237591	5.19E-08	3.49E-07	Down
ANKRD2	-3.6633845	-1.307364639	1.15E-19	3.01E-18	Down
HBQ1	-3.664051759	-1.721810244	3.14E-18	7.08E-17	Down
AC148477.4	-3.667264308	-3.228332697	1.86E-10	1.77E-09	Down
HLF	-3.668098275	4.19667439	6.75E-28	3.79E-26	Down
TUBA8	-3.668835765	-1.208897985	6.02E-38	9.17E-36	Down
ZNF536	-3.669568461	3.00516833	1.66E-17	3.51E-16	Down
LRP2	-3.673305408	2.894171442	5.55E-15	8.92E-14	Down
RTL1	-3.674776626	-1.063504427	5.92E-13	7.68E-12	Down
TRIM72	-3.674852772	-1.655021718	1.91E-39	3.46E-37	Down
LIMD1	2.024342722	5.323506065	3.13E-08	2.18E-07	Up
CYP4Z1	-3.676231882	-3.397088128	7.51E-11	7.57E-10	Down
AC090589.1	2.023991055	-1.080950446	7.04E-06	3.31E-05	Up
VIPR1	-3.685030705	2.064867483	8.21E-29	5.06E-27	Down
FGF13	-3.686188256	3.286269367	1.10E-15	1.92E-14	Down

IL1RAPL2	-3.687721395	-1.922860215	2.59E-10	2.42E-09	Down
LPAR3	-3.688009484	-1.53059264	3.02E-11	3.21E-10	Down
PCDH8P1	-3.689437031	-3.718999662	4.20E-09	3.31E-08	Down
RPL21P75	2.023675849	-0.94663455	3.02E-05	0.000125371	Up
TRIP13	2.022172659	3.749249003	2.08E-06	1.08E-05	Up
GJD2	-3.69012166	-0.827558959	3.77E-11	3.97E-10	Down
AL035252.2	-3.691138343	-3.180767347	1.13E-10	1.11E-09	Down
VSTM2L	-3.692454643	3.768128759	2.01E-18	4.63E-17	Down
ERMN	-3.694919373	5.922142712	1.28E-14	1.99E-13	Down
AC022960.2	-3.695175847	-2.740639618	7.02E-27	3.64E-25	Down
AC127496.2	-3.695465694	-2.804804919	9.36E-22	3.02E-20	Down
AP001972.5	-3.696597054	4.033584804	1.03E-35	1.24E-33	Down
FAR2P3	-3.6971918	-1.904059042	2.94E-12	3.54E-11	Down
CDH22	-3.698325593	2.859402738	5.64E-15	9.07E-14	Down
AC245164.1	-3.698347419	-3.853890105	8.45E-11	8.45E-10	Down
MAGEE1	-3.698509463	3.588845112	1.83E-32	1.60E-30	Down
BRINP1	-3.699109527	4.008632135	1.53E-17	3.24E-16	Down
SLC5A11	-3.703214228	2.383956586	1.74E-14	2.65E-13	Down
AC134312.4	-3.704909365	-2.139516719	7.38E-13	9.47E-12	Down
TMEM271	-3.705268748	-0.048349889	1.01E-14	1.58E-13	Down
KCNS1	-3.707419792	2.137716255	1.61E-15	2.74E-14	Down
NCDN	-3.709232741	7.022758733	1.60E-54	1.21E-51	Down
CASKIN1	-3.709771816	3.97126232	7.98E-33	7.28E-31	Down
AL445493.3	-3.717813684	-3.701290882	1.23E-11	1.37E-10	Down
TRIML2	-3.718074221	-2.882851718	1.20E-12	1.51E-11	Down
KCTD4	-3.719264479	1.624662355	4.65E-14	6.81E-13	Down
EPB41L4B	-3.720315539	2.908881202	3.94E-22	1.32E-20	Down
CNTN3	-3.721364246	1.863879525	1.77E-11	1.93E-10	Down
ANO6	2.021619961	6.622470959	3.17E-09	2.54E-08	Up
CCDC177	-3.721607289	0.077369207	2.94E-16	5.51E-15	Down
C1QTNF4	-3.722295698	2.008136012	2.59E-28	1.52E-26	Down
SLIT3	-3.724561488	3.242730018	2.18E-20	6.10E-19	Down
SPATA31C	-3.726522175	-3.541195733	3.86E-13	5.13E-12	Down
JAKMIP3	-3.728764472	1.906040024	1.15E-28	6.96E-27	Down
LINC01152	2.021208263	3.038638313	6.76E-06	3.18E-05	Up
AC015967.1	-3.729190157	-2.276480796	3.38E-26	1.66E-24	Down
PBX2P1	2.020108006	-1.07895516	2.06E-06	1.07E-05	Up
GABRA2	-3.730037546	3.706558025	1.59E-15	2.71E-14	Down
KRTAP5-AS	-3.733699382	-0.449488959	2.22E-18	5.08E-17	Down
DNAJC6	-3.73385623	5.829354075	1.36E-46	4.84E-44	Down

AP20DC-A	-3.736824704	-2.351854013	5.29E-09	4.11E-08	Down
ENC1	-3.736916394	7.752934871	5.17E-48	2.07E-45	Down
ADAP1	-3.738933136	3.380065605	8.10E-31	6.09E-29	Down
SRCIN1	-3.739737848	5.056261308	3.08E-27	1.66E-25	Down
AC138466.4	-3.741887694	-2.58307854	6.81E-15	1.09E-13	Down
NGB	-3.743075898	0.779936247	1.23E-10	1.20E-09	Down
NEFH	-3.747921393	3.138734395	1.38E-21	4.39E-20	Down
AC005089.1	-3.750240545	-3.459614566	9.83E-28	5.44E-26	Down
AC087457.1	-3.750876233	-2.898555558	3.45E-18	7.73E-17	Down
SLC1A6	-3.751376101	1.384638687	3.82E-10	3.48E-09	Down
STXBP5L	-3.754237664	2.958470646	5.63E-20	1.52E-18	Down
MAL	-3.757135	4.380363691	7.60E-16	1.35E-14	Down
AC009878.1	-3.763301641	-3.086836618	1.60E-18	3.75E-17	Down
AC015961.1	-3.764481337	-1.802963969	1.16E-16	2.25E-15	Down
SCN8A	-3.767008276	4.053699588	7.05E-55	6.35E-52	Down
NEDD1	2.019171213	4.53401444	5.84E-14	8.44E-13	Up
MTCO1P40	-3.76772045	3.759615445	5.10E-44	1.40E-41	Down
TMEM151A	-3.771312728	4.053514006	3.58E-18	8.03E-17	Down
ADRB3	-3.772585924	-2.834523079	3.22E-21	9.79E-20	Down
LINC02600	-3.773853486	-0.199074403	8.41E-14	1.20E-12	Down
MODL1-A:	-3.7744783	-1.58012607	2.91E-12	3.50E-11	Down
LINC01132	2.018576726	-0.239525999	0.000169347	0.000598573	Up
CNTN2	-3.776635074	6.48290509	2.62E-19	6.60E-18	Down
CKMT1B	-3.779498985	1.272970327	1.59E-14	2.43E-13	Down
GFOD1	-3.780018505	3.956695417	3.73E-58	4.29E-55	Down
GNG13	-3.786247489	-0.89581942	4.86E-19	1.20E-17	Down
CNTN4	-3.787485387	1.734219343	2.22E-20	6.20E-19	Down
AL136964.1	-3.788386976	-1.12940045	1.34E-16	2.58E-15	Down
CCBE1	-3.788710874	0.060617293	2.08E-09	1.71E-08	Down
SIRPB3P	-3.78992406	-3.453084948	6.98E-16	1.25E-14	Down
GALNT9	-3.791594906	3.234745399	7.65E-15	1.21E-13	Down
SNX18P7	-3.791708696	-3.968434933	2.01E-08	1.44E-07	Down
AL513175.1	2.017299102	-1.569225457	1.44E-06	7.72E-06	Up
CNTNAP4	-3.791754278	3.600182949	2.31E-16	4.38E-15	Down
MIR124-2HG	-3.795850581	1.183882302	6.61E-13	8.53E-12	Down
HAR1A	-3.797110126	-0.441903442	1.88E-18	4.34E-17	Down
HSPA12A	-3.797239033	4.673841585	4.04E-43	1.01E-40	Down
FTH1P7	2.016197383	2.670166117	1.92E-07	1.18E-06	Up
HMGA1P7	-3.799106634	-2.8333609	1.57E-09	1.32E-08	Down
PPP1R16B	-3.799322391	4.972367551	1.08E-20	3.11E-19	Down

NAP1L2	-3.803529571	3.771994428	1.54E-29	1.02E-27	Down
HTR3A	-3.805229068	-2.363806905	9.44E-13	1.20E-11	Down
LINC01721	-3.805586243	-3.672642286	1.16E-09	9.94E-09	Down
STXBP6	-3.806158218	3.233378132	4.98E-23	1.82E-21	Down
CELF5	-3.807788804	4.428068057	3.60E-16	6.71E-15	Down
NKAIN2	-3.810306619	3.181836766	2.91E-16	5.47E-15	Down
AL031598.1	-3.810311339	-3.369918554	2.13E-08	1.52E-07	Down
NKRD20A1	-3.810861204	-3.068758652	1.70E-10	1.63E-09	Down
TMEM255A	2.015632521	6.266813284	1.01E-05	4.59E-05	Up
AL133166.1	-3.811268024	-3.199865466	8.47E-11	8.46E-10	Down
AL121827.1	-3.811979144	-1.860623408	4.54E-13	5.98E-12	Down
AL139396.1	-3.812019959	-3.438714344	1.26E-13	1.76E-12	Down
NPTXR	-3.81385499	7.128030591	1.16E-39	2.15E-37	Down
ATP8A1	-3.816293521	5.161933301	1.81E-37	2.58E-35	Down
MIR657	-3.816908235	-3.331303802	9.73E-16	1.70E-14	Down
INA	-3.820348657	4.392232541	9.84E-13	1.24E-11	Down
SIAH3	-3.822227805	-0.025155916	2.63E-19	6.61E-18	Down
MGAT3-AS	-3.824420293	-3.203132515	3.40E-24	1.39E-22	Down
GPR78	-3.8250376	-2.769833402	2.48E-21	7.63E-20	Down
CCSER1	-3.82529463	-1.458422107	2.76E-25	1.25E-23	Down
AQP7	-3.826137702	-2.522653266	1.34E-18	3.15E-17	Down
AL137786.1	-3.827335757	-0.637597052	1.16E-13	1.63E-12	Down
KIAA0319	-3.827984438	2.877232349	1.03E-28	6.28E-27	Down
CKMT1A	-3.828365647	0.763921407	6.92E-16	1.24E-14	Down
AC233702.4	-3.82894516	-3.609346017	9.81E-11	9.73E-10	Down
DGKE	-3.829256068	2.248211249	1.35E-40	2.70E-38	Down
TRIP10	2.015028908	5.012437711	2.16E-10	2.04E-09	Up
ADRA1B	-3.83363281	1.723136872	1.43E-33	1.43E-31	Down
GRM3	-3.834357951	4.114436277	3.75E-28	2.16E-26	Down
C10orf82	-3.834849823	-2.286902451	1.08E-19	2.84E-18	Down
PNCK	-3.83643791	3.359808653	2.34E-32	2.05E-30	Down
UNC5A	-3.838035165	3.816934219	5.75E-21	1.70E-19	Down
RTBDN	-3.838273129	0.378623428	7.83E-21	2.29E-19	Down
LHX6	-3.840752294	1.880327575	7.73E-28	4.31E-26	Down
CRHBP	-3.840836678	1.368709543	1.91E-19	4.88E-18	Down
CACNA2D	-3.84133348	2.186985026	1.02E-24	4.39E-23	Down
AC021683.2	-3.841606154	-0.570499603	1.50E-19	3.85E-18	Down
CYP1A1	-3.843360802	-2.274478139	3.96E-22	1.33E-20	Down
PLCH2	-3.843912302	3.045432008	2.71E-19	6.79E-18	Down
AC092296.3	-3.844457635	-3.02670985	7.40E-15	1.17E-13	Down

LINC0225[illegible]	-3.84724156	-3.480995621	6.74E-11	6.85E-10	Down
AC019069.[illegible]	2.013952079	1.929293322	1.64E-05	7.15E-05	Up
BHLHE22	-3.848241068	0.927045759	2.57E-10	2.40E-09	Down
VWA5B2	-3.852253323	2.16832017	3.34E-40	6.51E-38	Down
MAST3	-3.852314048	5.421341927	1.02E-65	2.95E-62	Down
AC010973.[illegible]	2.012500423	0.566047257	3.54E-05	0.000144636	Up
ACP7	-3.852787173	-0.838787411	5.09E-13	6.66E-12	Down
CLEC4G	-3.856014875	-1.038729773	4.33E-12	5.11E-11	Down
HTR6	-3.857939881	-1.942100448	9.28E-16	1.63E-14	Down
AC148477.[illegible]	-3.858329407	-2.588417136	6.25E-10	5.52E-09	Down
CHGB	-3.858349886	4.876749413	7.71E-21	2.26E-19	Down
RIMS3	-3.858508265	5.143356795	2.04E-31	1.63E-29	Down
CPLX1	-3.859296963	4.347947544	1.19E-22	4.19E-21	Down
RAPGEF4	-3.861495088	5.158648794	6.21E-39	1.05E-36	Down
SIRPB2	2.011231804	3.018289788	0.000909505	0.002714854	Up
PAK3	-3.861734156	4.03797671	1.54E-23	5.94E-22	Down
SH3GL2	-3.863889492	4.92222975	2.80E-17	5.76E-16	Down
ATOH7	-3.866742089	-1.060959248	7.79E-32	6.41E-30	Down
DMTN	-3.867157486	5.62690018	3.36E-39	5.91E-37	Down
CHP2	-3.867512079	-3.078552958	1.96E-16	3.74E-15	Down
RGS7	-3.867741316	2.7038715	9.21E-24	3.63E-22	Down
SLC17A6	-3.868337936	1.423301375	8.15E-10	7.09E-09	Down
OPRK1	-3.868961825	0.367001816	6.20E-11	6.32E-10	Down
LINC0249[illegible]	-3.870193955	-0.334822907	1.92E-15	3.24E-14	Down
NGEF	-3.875298419	4.973021037	7.08E-20	1.89E-18	Down
KCNAB2	-3.876643959	5.77421758	7.16E-49	3.22E-46	Down
SELE	-3.878880362	-0.254107371	3.68E-14	5.45E-13	Down
COPZ2	2.010904152	4.565443524	0.000501873	0.001591391	Up
AC148477.[illegible]	-3.879595022	-2.806255081	1.74E-11	1.91E-10	Down
CPEB3	-3.882021744	2.887239156	3.93E-62	7.54E-59	Down
[illegible]NFRSF10[illegible]	2.010726856	5.59769698	4.37E-06	2.13E-05	Up
SLITRK4	-3.902083618	2.132304066	3.31E-14	4.93E-13	Down
CHRNB3	-3.905160728	-3.19098616	4.44E-10	4.00E-09	Down
PPFIA2	-3.910355928	3.311924524	5.25E-22	1.73E-20	Down
AC092720.[illegible]	-3.9107589	-0.752354708	1.39E-11	1.54E-10	Down
HNF4A	-3.912315907	-3.169476692	7.89E-11	7.92E-10	Down
AC010857.[illegible]	-3.91631896	-2.73231663	5.69E-14	8.25E-13	Down
TUNAR	-3.917194161	0.922162062	6.09E-13	7.88E-12	Down
AL020994.[illegible]	-3.918172861	-3.763627322	4.18E-10	3.78E-09	Down
NMNAT2	-3.921653113	4.813395681	3.57E-25	1.60E-23	Down

SPTBN2	-3.922990216	5.508523816	8.19E-36	1.02E-33	Down
KLHL34	-3.923303693	-1.553532305	1.64E-23	6.27E-22	Down
AL645940.1	2.009596592	-1.129302032	0.000830898	0.002505913	Up
SMPX	-3.925317736	-2.30050111	7.94E-10	6.92E-09	Down
RND2	2.009013064	7.525090658	2.42E-05	0.000102268	Up
RS1	-3.925762479	-1.527235019	2.13E-17	4.45E-16	Down
AP001972.3	-3.926343383	-2.728883541	8.89E-22	2.88E-20	Down
NF144A-A	-3.927005421	0.510281742	1.82E-19	4.66E-18	Down
KHDRBS2	-3.928754764	0.02623979	5.78E-15	9.26E-14	Down
AL139246.1	-3.92949719	-3.129703552	4.40E-14	6.46E-13	Down
CAPN13	-3.929739208	-1.727566293	3.82E-16	7.08E-15	Down
STXBP1	-3.930713964	6.884721179	1.14E-51	6.22E-49	Down
FEZF2	-3.933601345	0.795449295	9.74E-17	1.91E-15	Down
STX1A	-3.934380291	4.736566928	8.07E-46	2.67E-43	Down
SLC24A2	-3.935143824	4.636214687	1.16E-27	6.40E-26	Down
RAB11FIP	-3.936589279	5.059038051	2.05E-37	2.88E-35	Down
VWA7	-3.938368977	0.852773433	2.43E-38	3.87E-36	Down
SPPL2C	-3.938573459	-3.090366981	2.24E-21	6.93E-20	Down
AC134026.2	-3.939057169	-3.584258113	5.54E-12	6.46E-11	Down
AATK	-3.942838145	5.247767921	4.13E-27	2.18E-25	Down
AL139246.4	-3.947081578	-3.056914076	1.76E-14	2.69E-13	Down
AC005329.3	-3.949049162	-1.921000087	2.72E-33	2.62E-31	Down
CXCL16	2.008700967	6.608868576	7.21E-07	4.06E-06	Up
GCSHP5	2.008388088	0.122137947	5.71E-07	3.27E-06	Up
MAP3K9	-3.950650407	2.910703531	4.58E-29	2.90E-27	Down
CYP4X1	-3.950725728	0.978544931	2.77E-30	1.96E-28	Down
GNAL	-3.95250296	3.019903923	5.32E-35	6.06E-33	Down
AC097641.1	-3.954128655	-3.438040812	5.69E-15	9.13E-14	Down
AC126177.(	-3.957304842	-3.043959058	2.04E-26	1.01E-24	Down
SMIM32	-3.959912134	-1.289349284	4.38E-16	8.04E-15	Down
CES4A	-3.961175617	1.965320784	3.04E-31	2.41E-29	Down
CBLN2	-3.962899612	1.606897791	7.61E-13	9.75E-12	Down
SLC16A3	2.008299075	6.072563784	0.000376138	0.001228525	Up
LGI3	-3.963561315	4.971298412	1.76E-19	4.50E-18	Down
ABCG8	-3.964507605	-3.302283602	2.23E-18	5.10E-17	Down
CREB3L3	-3.966723706	-3.429076885	4.05E-14	5.95E-13	Down
ANKRD63	-3.968240222	-2.600375622	5.81E-20	1.56E-18	Down
KCNK4	-3.968428816	-2.315809803	1.41E-22	4.96E-21	Down
ARAP3	2.00761792	5.705666387	9.12E-05	0.000342509	Up
UNC13A	-3.972748791	5.394865676	1.23E-26	6.28E-25	Down

PRPH2	-3.9765901	0.01160559	6.50E-31	4.93E-29	Down
GLS2	-3.981832226	-0.911023273	4.30E-44	1.19E-41	Down
SLC26A8	-3.984837999	-0.779696824	4.85E-33	4.53E-31	Down
PSD	-3.989437699	5.44591049	3.83E-31	2.99E-29	Down
'ER1L6-AS	-3.994989541	-2.747775021	2.15E-25	9.80E-24	Down
CAMK4	-3.995473207	3.421478476	1.17E-37	1.70E-35	Down
EPHB6	-3.996431445	4.387314375	5.03E-28	2.86E-26	Down
LINC0089ε	-4.002799091	-3.053119818	2.34E-06	1.20E-05	Down
SGSM1	-4.008276744	2.956683747	3.79E-31	2.96E-29	Down
EPHA10	-4.009474173	1.812618394	4.35E-18	9.70E-17	Down
AC233702.:	-4.013866241	-3.68312775	7.61E-14	1.09E-12	Down
ERC2	-4.014631272	3.208181761	1.16E-42	2.82E-40	Down
PITPNM3	-4.014694142	3.976224354	2.84E-37	3.93E-35	Down
MED4-AS1	2.007243106	-2.136806593	1.84E-05	7.98E-05	Up
RBP4	-4.01694867	1.653323901	6.26E-22	2.06E-20	Down
CA7	-4.019925767	-0.232388499	2.08E-18	4.77E-17	Down
KCNK1	-4.021597796	3.524484706	1.19E-21	3.80E-20	Down
SPHKAP	-4.02190652	1.860734365	3.71E-16	6.90E-15	Down
CACNG8	-4.021939001	4.371326605	7.43E-24	2.96E-22	Down
HPCA	-4.022270784	4.573809844	5.98E-23	2.16E-21	Down
ATP1A3	-4.025132527	7.303747523	1.92E-20	5.40E-19	Down
SHANK2	-4.027470852	3.139987251	1.71E-20	4.84E-19	Down
TMEM151I	-4.030118519	3.885582294	5.48E-25	2.42E-23	Down
CEACAM5	-4.032902392	-3.52471141	1.64E-06	8.68E-06	Down
LINC0160ε	-4.034156218	-2.221563922	2.59E-13	3.50E-12	Down
GALNTL5	-4.037885581	-1.410265002	1.36E-11	1.51E-10	Down
MYOF	2.006320442	5.633797451	0.000107979	0.000399091	Up
GAPDHP4:	-4.03830348	-3.4358451	3.09E-27	1.66E-25	Down
KCNH3	-4.042937793	3.793236914	3.03E-46	1.06E-43	Down
SYT16	-4.045779423	3.424706561	5.20E-24	2.11E-22	Down
CR589904.:	-4.046630827	-3.908027365	9.22E-07	5.09E-06	Down
PLCXD3	-4.047131878	2.282682871	1.97E-17	4.13E-16	Down
AC117453.:	-4.050642646	-3.066954822	1.73E-20	4.90E-19	Down
PNMA3	-4.052537043	2.947187706	7.00E-24	2.80E-22	Down
PNMA8B	-4.053874818	3.955716038	1.54E-61	2.54E-58	Down
CDKL5	-4.055743218	4.072656642	2.10E-64	5.05E-61	Down
TMEM218	2.005311703	5.095107254	2.57E-10	2.40E-09	Up
NKRD20A1	-4.059210502	-3.592350098	3.90E-11	4.10E-10	Down
RAB3B	-4.059792619	2.954011304	1.49E-26	7.55E-25	Down
ARHGDIG	-4.063871013	3.699926018	3.91E-20	1.07E-18	Down

CAMKK1	-4.067062436	4.573449903	9.89E-42	2.21E-39	Down
SLC6A15	-4.070025628	2.52656357	4.25E-16	7.82E-15	Down
GABRB3	-4.07048132	4.371427756	3.89E-18	8.71E-17	Down
BGN	2.004888397	8.35128794	0.000104865	0.000388876	Up
AC011474.1	-4.077788755	-0.578828539	5.26E-14	7.65E-13	Down
GNG3	-4.078597408	4.018838886	6.87E-22	2.25E-20	Down
GRM7	-4.07921844	1.218678162	3.40E-17	6.94E-16	Down
ADARB2	-4.080973863	2.716591091	1.21E-22	4.26E-21	Down
SERPINI1	-4.081765939	4.822714485	8.89E-30	6.04E-28	Down
DNM1	-4.083487581	6.984298285	2.47E-42	5.79E-40	Down
CPNE7	-4.083723878	1.811062278	2.25E-20	6.27E-19	Down
CACNG2	-4.084860319	0.30538528	1.91E-13	2.62E-12	Down
AC007368.1	-4.085247258	-3.6029251	8.78E-13	1.12E-11	Down
CDKL2	-4.087503791	0.676270344	8.32E-27	4.27E-25	Down
DLG2	-4.089636763	4.830895215	3.96E-53	2.54E-50	Down
HECW1	-4.097668893	2.690680628	3.49E-21	1.06E-19	Down
KCNH1	-4.099265505	1.302795964	6.40E-21	1.88E-19	Down
CLEC4GP1	-4.099467539	-1.959761882	5.18E-11	5.35E-10	Down
LDB3	-4.101579275	2.462540641	1.43E-27	7.86E-26	Down
STX1B	-4.119055074	5.368211406	4.45E-41	9.36E-39	Down
AC135782.1	-4.121307344	-1.17844039	2.85E-24	1.17E-22	Down
ADAM11	-4.122697166	3.488500844	2.10E-45	6.64E-43	Down
PART1	-4.12323093	1.963408613	7.40E-36	9.24E-34	Down
PYDC1	-4.127551571	-2.171101783	1.49E-13	2.07E-12	Down
KCNK12	-4.128815134	1.232624134	6.65E-25	2.91E-23	Down
TNNT2	-4.129917131	-0.053283947	3.95E-13	5.25E-12	Down
RIMS2	-4.130408175	2.76243443	5.04E-18	1.12E-16	Down
JAKMIP1	-4.133066763	3.066480121	8.43E-26	3.98E-24	Down
AP003108.5	-4.133091909	-2.994399262	3.07E-17	6.30E-16	Down
LGAP1-AS	-4.137787962	-0.55143287	1.88E-18	4.35E-17	Down
REPS2	-4.13813628	3.900720567	8.39E-36	1.03E-33	Down
LAVL4-AS	-4.140507028	-3.492901557	1.30E-15	2.24E-14	Down
AC004925.1	-4.143141872	1.547437381	3.56E-32	3.07E-30	Down
KCNJ9	-4.145123938	4.159358959	7.85E-27	4.05E-25	Down
CRYGN	-4.145307902	-1.825432653	3.22E-38	5.04E-36	Down
GRM2	-4.149077085	1.669531916	3.25E-30	2.27E-28	Down
RASGRF2	-4.149290327	3.554728412	2.83E-67	1.02E-63	Down
CLEC2L	-4.149693553	1.348149276	1.89E-17	3.98E-16	Down
HCRTR2	-4.154216222	-3.445423778	3.57E-12	4.26E-11	Down
OPN4	-4.154222063	-2.170681043	2.69E-19	6.75E-18	Down

MATK	-4.154596312	2.232018102	1.33E-42	3.16E-40	Down
NRNPA1P	2.004489149	-2.516720305	0.000351838	0.001157422	Up
WIF1	-4.154911965	2.105206769	2.07E-10	1.96E-09	Down
NEGR1	-4.155922831	4.070352671	3.08E-24	1.27E-22	Down
CRH	-4.15673547	-0.787055918	1.83E-11	2.00E-10	Down
IGFBP5	2.004288402	10.20292319	0.000912514	0.002723269	Up
SDR16C5	-4.157641336	-0.654330791	9.88E-13	1.25E-11	Down
NECAB2	-4.162212355	3.605715736	3.35E-31	2.65E-29	Down
ERFL	-4.166072743	-0.930537103	8.72E-45	2.59E-42	Down
GABRD	-4.168537793	3.377682	2.09E-26	1.04E-24	Down
SYP	-4.168884611	6.665483748	1.50E-42	3.55E-40	Down
CD22	-4.173108124	2.203324958	8.50E-25	3.66E-23	Down
AC079296.1	-4.175587896	-2.951989432	1.65E-21	5.21E-20	Down
LINGO2	-4.175624241	-0.307240596	3.46E-14	5.14E-13	Down
SPTB	-4.17760778	2.308971729	6.62E-26	3.15E-24	Down
GVINP2	-4.177809019	-3.755065324	3.64E-21	1.10E-19	Down
CARMIL2	-4.178580877	1.83795585	8.09E-34	8.33E-32	Down
SCN2A	-4.18013858	4.624192763	1.59E-61	2.54E-58	Down
PDE1A	-4.183847649	3.373854494	9.53E-39	1.58E-36	Down
FBXL16	-4.188033224	6.250402501	1.25E-32	1.11E-30	Down
LINC0234	-4.18922816	-3.268542863	1.56E-22	5.45E-21	Down
LINC0229	-4.189653501	-3.696319677	4.98E-17	1.00E-15	Down
GABRG1	-4.189755626	2.725511936	1.83E-14	2.79E-13	Down
DOK6	-4.190372474	2.904740882	1.60E-26	8.05E-25	Down
LINC0219	-4.19039595	-2.637991075	7.47E-13	9.58E-12	Down
IPCEF1	-4.197311491	2.805847275	2.97E-47	1.11E-44	Down
ANK3	-4.199683869	4.550233653	5.83E-54	4.10E-51	Down
CDH12	-4.200765517	-0.187195529	2.86E-15	4.72E-14	Down
MIM10L2	-4.201325537	2.713756049	1.53E-53	1.00E-50	Down
PLAT	2.004242256	6.728856314	0.000443362	0.001423562	Up
CHGA	-4.20396424	4.476522039	5.06E-15	8.16E-14	Down
LINC0141	-4.205652731	-3.576245546	2.09E-13	2.84E-12	Down
LINC0125	-4.20696445	-2.305394372	9.88E-18	2.13E-16	Down
SYT7	-4.208807546	5.045094224	2.15E-28	1.27E-26	Down
PTPRT	-4.210320667	3.831438638	3.73E-21	1.12E-19	Down
MPP7	-4.210730941	0.644331381	1.30E-29	8.75E-28	Down
KCNA1	-4.215420372	2.031553581	1.84E-22	6.39E-21	Down
SLC5A5	-4.215637264	-1.89425258	9.48E-26	4.45E-24	Down
Z94160.1	-4.217227739	-3.454239045	3.22E-17	6.60E-16	Down
CDH18	-4.217581166	1.675568643	1.55E-14	2.38E-13	Down

NELL1	-4.217957457	2.317022177	5.03E-16	9.18E-15	Down
LINC0266[illegible]	-4.219398705	-3.15113397	3.46E-17	7.06E-16	Down
NPM2	-4.219647605	1.748862908	2.74E-26	1.35E-24	Down
AKAP5	-4.220431332	2.635231343	2.32E-69	1.11E-65	Down
AC148477.[illegible]	-4.221831291	-2.193545257	9.69E-13	1.23E-11	Down
LINC0285[illegible]	-4.224033442	-3.549687064	1.27E-15	2.19E-14	Down
GPR61	-4.224406038	0.376390899	4.51E-49	2.06E-46	Down
ADAD2	-4.226203103	0.672745201	1.55E-29	1.03E-27	Down
FNDC9	-4.232410759	1.648149308	7.93E-21	2.32E-19	Down
LINC0116[illegible]	-4.233153338	-1.746566763	2.19E-11	2.37E-10	Down
AC124804.[illegible]	-4.233655125	-3.065736809	7.25E-18	1.59E-16	Down
[illegible]LHL30-AS	-4.234749273	-3.232669399	1.35E-22	4.73E-21	Down
ACVR1C	-4.235489373	1.141621563	5.14E-25	2.28E-23	Down
LINC0129[illegible]	-4.235562539	-1.707509865	3.56E-13	4.76E-12	Down
MARCHF1[illegible]	-4.237022238	-1.722520419	1.24E-12	1.55E-11	Down
LYPD8	-4.238070135	-0.992884245	4.45E-14	6.53E-13	Down
SLC4A10	-4.238132458	3.95650049	1.84E-34	2.03E-32	Down
KCNJ12	-4.238226643	0.340055434	1.68E-23	6.39E-22	Down
SNCA	-4.238427591	4.816470531	3.06E-39	5.44E-37	Down
RN7SL471[illegible]	-4.239807031	-3.387907467	6.84E-10	6.01E-09	Down
AC090136.[illegible]	-4.240000052	-2.915127359	8.96E-12	1.01E-10	Down
MFSD4A	-4.244155555	3.863534937	2.40E-52	1.44E-49	Down
RIMS1	-4.252269243	2.879412212	6.14E-27	3.22E-25	Down
LRFN5	-4.253483035	1.861246586	2.73E-18	6.20E-17	Down
BASP1-AS1	-4.253517713	-1.272201773	6.40E-19	1.55E-17	Down
AIDAP2	-4.256448332	0.585895125	2.71E-29	1.75E-27	Down
FRRS1L	-4.257529247	4.161636858	1.60E-26	8.05E-25	Down
MTCO1P2	2.003930695	1.132710191	1.51E-05	6.64E-05	Up
CCDC85A	-4.261435405	0.686332745	3.92E-32	3.36E-30	Down
AC136759.[illegible]	-4.262773902	-1.151651866	1.27E-11	1.41E-10	Down
AC036111.[illegible]	-4.264015002	-3.665336117	3.63E-10	3.32E-09	Down
GJB6	-4.265753533	2.163808811	4.72E-11	4.91E-10	Down
KSR2	-4.266335973	1.943170339	4.46E-16	8.18E-15	Down
PCDH11Y	-4.26647858	-0.427441295	2.91E-11	3.11E-10	Down
AC122707.[illegible]	-4.269616209	-2.662631937	4.39E-17	8.89E-16	Down
PDYN	-4.272597214	3.527301685	1.37E-14	2.12E-13	Down
SYNGR3	-4.280489188	3.921593936	3.62E-28	2.08E-26	Down
TRPV6	-4.283635898	0.156593993	4.52E-27	2.38E-25	Down
AC062028.[illegible]	-4.284123407	-1.003548501	4.43E-29	2.81E-27	Down
SEC14L5	-4.285585428	3.123349986	5.53E-23	2.01E-21	Down

RHOQP3	2.002761258	-2.527163512	0.000327622	0.001085443	Up
AF106564.1	-4.286402173	-0.168987361	2.96E-15	4.87E-14	Down
AC020907.2	-4.292299902	-2.576496947	7.91E-17	1.56E-15	Down
AIRN	-4.297008553	-3.724409675	6.63E-20	1.77E-18	Down
C1orf115	-4.297778429	4.166631495	4.67E-48	1.89E-45	Down
AC226101.1	-4.299095027	-3.607468259	1.98E-24	8.29E-23	Down
PAK6	-4.299552255	-0.486349947	6.36E-45	1.93E-42	Down
CAMK1G	-4.30014101	2.677503407	5.59E-20	1.51E-18	Down
CABP1	-4.301607795	3.204609597	6.98E-27	3.63E-25	Down
CDH8	-4.306325477	2.35821011	3.99E-27	2.11E-25	Down
AC009084.2	-4.307194187	-0.564943529	2.48E-29	1.61E-27	Down
DACH2	-4.308093942	1.213861256	1.17E-18	2.78E-17	Down
SH3GL3	-4.308843975	3.049658642	3.90E-23	1.44E-21	Down
RAB3A	-4.309730005	5.070366076	8.67E-39	1.44E-36	Down
SOWAHA	-4.31145831	2.973929048	9.15E-38	1.36E-35	Down
AC103681.2	-4.311676536	-3.571220197	1.89E-26	9.44E-25	Down
AC010266.2	-4.313541399	-1.852539234	8.16E-20	2.16E-18	Down
SYT5	-4.313566131	3.981419778	8.92E-29	5.49E-27	Down
AC124312.2	-4.315066926	-0.551312351	6.46E-40	1.22E-37	Down
LINC0238[illegible]	-4.315471275	-1.458168705	5.16E-21	1.54E-19	Down
AC025253.1	-4.316318522	-3.407205884	8.86E-22	2.88E-20	Down
PABPC1L2A	-4.317629747	-1.269007776	1.35E-15	2.33E-14	Down
PDE2A	-4.319530095	5.023725372	2.38E-37	3.32E-35	Down
AC140125.2	-4.320666748	-2.612226673	3.87E-14	5.71E-13	Down
NEUROD2	-4.321108762	2.569334662	9.54E-18	2.06E-16	Down
PABPC1L2B	-4.322898458	-1.183168683	4.31E-15	7.01E-14	Down
AC104417.2	-4.324401075	-1.832966951	6.15E-13	7.96E-12	Down
GPR149	-4.330191316	-3.739932569	8.58E-18	1.87E-16	Down
CACNA1E	-4.330616581	3.801028001	1.93E-31	1.55E-29	Down
CCK	-4.33128327	3.776569947	4.41E-16	8.10E-15	Down
AL022313.4	-4.332092735	1.265351281	3.70E-15	6.05E-14	Down
SLC7A14	-4.334219596	3.75446323	2.12E-26	1.05E-24	Down
EPHA8	-4.334550871	-1.39896609	3.31E-30	2.31E-28	Down
NHBA-AS	-4.33960094	-1.046776065	9.60E-24	3.78E-22	Down
SLC35F3	-4.339660835	0.720130443	2.69E-24	1.11E-22	Down
HS3ST2	-4.348289382	3.227477337	1.08E-22	3.84E-21	Down
SYT4	-4.349799575	3.867277503	2.80E-18	6.35E-17	Down
TAFA2	-4.352360331	2.343207698	2.89E-39	5.17E-37	Down
NEURL1	-4.355970033	3.305069157	2.82E-36	3.66E-34	Down
LINC0111[illegible]	-4.359828336	-3.594351536	1.12E-09	9.59E-09	Down

AC011287.1	-4.360201548	-2.661313548	4.35E-13	5.76E-12	Down
FFAR1	-4.363338396	-2.471182135	3.14E-19	7.86E-18	Down
IL12RB2	-4.372067884	-1.098844023	7.74E-57	7.97E-54	Down
AC026790.1	-4.374132478	-3.009002997	1.21E-14	1.88E-13	Down
HS3ST4	-4.37528456	1.837509073	3.35E-21	1.01E-19	Down
SNCB	-4.375668634	5.201654753	3.62E-22	1.22E-20	Down
AC124303.2	-4.375776737	-2.054966261	1.06E-37	1.56E-35	Down
WNT1	-4.380747192	-2.644094667	1.25E-29	8.41E-28	Down
HDRBS2-O	-4.385041462	-3.090202733	7.59E-16	1.35E-14	Down
GABRA6	-4.386797994	-3.580490102	1.93E-15	3.25E-14	Down
KIAA0513	-4.389496945	5.849470075	6.54E-83	1.89E-78	Down
AP003559.1	-4.392315939	-2.173335722	7.94E-38	1.19E-35	Down
DLGAP3	-4.394352636	3.251173634	1.42E-34	1.58E-32	Down
AC104623.2	-4.39495445	-2.346893146	2.53E-35	2.94E-33	Down
ASIC2	-4.395032921	1.50917801	5.84E-19	1.42E-17	Down
RGS4	-4.396430405	4.97381496	1.16E-24	4.97E-23	Down
GREM2	-4.407807596	1.1211311	1.95E-25	8.93E-24	Down
AC121757.1	-4.40952893	-3.14402213	1.75E-16	3.35E-15	Down
TD-2297D1	-4.410286081	-2.966710723	3.49E-16	6.51E-15	Down
PEX5L	-4.410772722	3.942193038	2.79E-29	1.80E-27	Down
PTPRR	-4.414196747	1.772688895	4.83E-28	2.76E-26	Down
LCN15	-4.418638237	-1.599621314	1.64E-17	3.46E-16	Down
NAPB	-4.421332916	5.901529456	3.87E-58	4.29E-55	Down
HPCAL4	-4.422903395	5.481805883	2.49E-26	1.23E-24	Down
PTPN5	-4.426102416	3.880287696	9.40E-24	3.70E-22	Down
RBX1P2	2.001125576	0.063883544	5.00E-06	2.41E-05	Up
AC139491.1	-4.428177076	-1.67641395	2.41E-13	3.27E-12	Down
AC026585.1	-4.429990546	-3.470208478	5.04E-19	1.24E-17	Down
SNCG	-4.430163453	3.560547522	4.37E-31	3.37E-29	Down
ST8SIA3	-4.432945027	4.161592899	4.48E-19	1.11E-17	Down
TAFA1	-4.432951787	1.547502271	2.68E-21	8.18E-20	Down
AC021683.1	-4.438360531	-0.22827815	2.80E-25	1.27E-23	Down
CHRM2	-4.439853575	-0.401373791	7.71E-18	1.69E-16	Down
AJAP1	-4.440064927	2.929288161	8.29E-31	6.22E-29	Down
CYP4F26P	-4.443619885	-3.139992294	1.87E-12	2.29E-11	Down
AC104072.1	-4.445513601	0.956980818	3.90E-22	1.31E-20	Down
CNTNAP2	-4.446332713	4.263867371	9.98E-26	4.67E-24	Down
SLC6A5	-4.454368925	-1.609363887	2.09E-14	3.16E-13	Down
MT-TL1	-4.458166486	-3.044537239	1.76E-33	1.73E-31	Down
LRFN2	-4.459190171	1.233296045	1.25E-24	5.32E-23	Down

AC103563.2	-4.465034341	-3.287971262	8.19E-17	1.61E-15	Down
)CTN1-AS	-4.46516315	-0.664364855	3.18E-27	1.70E-25	Down
AP000843.1	-4.465400266	-1.8048951	1.25E-18	2.95E-17	Down
TMEM272	-4.468483526	-0.292411711	4.48E-29	2.84E-27	Down
SAMD12	-4.469622717	2.266000684	8.61E-64	1.91E-60	Down
TPPP	-4.471402929	6.210471483	3.22E-35	3.71E-33	Down
ODC1	2.000746802	7.772193087	1.85E-08	1.33E-07	Up
DOC2A	-4.472110181	3.528210938	1.00E-32	8.93E-31	Down
NYAP2	-4.472389549	-1.420647389	3.48E-17	7.10E-16	Down
CRYM	-4.474187337	3.882635192	1.37E-17	2.91E-16	Down
CAMKV	-4.4790411	4.698552194	1.01E-24	4.32E-23	Down
NRXN3	-4.482930557	3.937263991	5.83E-36	7.33E-34	Down
RGS7BP	-4.485908152	2.426834495	1.89E-45	6.04E-43	Down
LINC0112:	-4.490731542	-1.723573851	1.60E-15	2.73E-14	Down
IQSEC3	-4.49138956	3.565488429	3.98E-27	2.11E-25	Down
GRM1	-4.496845102	1.751175614	6.61E-28	3.72E-26	Down
THSD4-AS	-4.498449276	-2.105404778	1.99E-15	3.36E-14	Down
RELN	-4.505233362	2.756997649	1.11E-23	4.34E-22	Down
JPH3	-4.507599809	4.103649043	1.95E-21	6.10E-20	Down
SCN2B	-4.508770527	3.792918089	7.83E-44	2.11E-41	Down
ARHGAP4	-4.511341076	2.995084409	7.30E-44	1.99E-41	Down
ZNF831	-4.512325691	-0.593519209	1.96E-31	1.57E-29	Down
DNAJC5G	-4.514743428	-2.632392062	1.16E-20	3.32E-19	Down
TACR3	-4.515228415	-1.948126185	1.13E-25	5.26E-24	Down
AC120193.1	-4.516234149	-3.444215745	2.09E-16	3.96E-15	Down
TMEM215	-4.519826685	-1.753948863	7.08E-18	1.55E-16	Down
SYN1	-4.520317374	5.944688512	2.93E-30	2.06E-28	Down
GRM4	-4.524902942	0.387851339	2.66E-21	8.13E-20	Down
ANKRD30I	-4.534850528	-2.769281207	4.95E-22	1.64E-20	Down
AMER3	-4.535480815	1.45152999	1.14E-19	2.98E-18	Down
GALNT17	-4.537940018	4.29266459	1.85E-18	4.28E-17	Down
PSG8-AS1	-4.538854843	-2.254563144	2.42E-22	8.34E-21	Down
NRGN	-4.539602483	6.509827838	7.71E-33	7.07E-31	Down
KCTD16	-4.544476224	2.685335153	1.76E-32	1.55E-30	Down
ISLR2	-4.545406857	2.964994677	7.82E-26	3.70E-24	Down
VIP	-4.546180334	0.216736136	6.05E-28	3.42E-26	Down
CIDEA	-4.550691234	-2.410539465	1.63E-18	3.80E-17	Down
NEDD4	2.000731873	3.988935166	7.36E-11	7.43E-10	Up
FAM153B	-4.550786094	0.273437247	1.04E-16	2.03E-15	Down
PRKCB	-4.553866342	5.330630213	2.81E-45	8.81E-43	Down

C11orf87	-4.554796682	2.926537332	3.28E-27	1.75E-25	Down
CARNS1	-4.554804077	5.355199621	3.18E-22	1.08E-20	Down
PRMT8	-4.555109369	1.743944295	7.94E-33	7.26E-31	Down
PP12613	-4.558711476	-3.183368834	1.79E-21	5.59E-20	Down
AC125616.1	-4.561067067	-1.742910346	5.33E-21	1.59E-19	Down
KCNG3	-4.561460051	-1.581787388	5.64E-21	1.67E-19	Down
CPNE9	-4.564096388	0.721266969	2.34E-54	1.73E-51	Down
LRRC7	-4.566840549	2.542346211	2.41E-44	6.96E-42	Down
EGR4	-4.56880392	-0.301987125	1.33E-20	3.80E-19	Down
AC008164.1	-4.568922191	-3.774562267	4.28E-17	8.69E-16	Down
GABRG3	-4.569298308	0.928677347	8.81E-18	1.91E-16	Down
CRHR2	-4.572237195	-0.755403129	1.02E-28	6.24E-27	Down
SNAP91	-4.572537369	4.847227741	1.90E-24	7.98E-23	Down
LINCR-000	-4.572847921	-1.068037377	1.37E-15	2.35E-14	Down
LINC0116(	-4.578148415	-2.702171497	1.97E-25	8.99E-24	Down
AC003684.1	-4.578286298	-3.915770403	6.21E-11	6.33E-10	Down
CELF2-DT	-4.580842512	-2.490219645	2.79E-15	4.61E-14	Down
BSN	-4.59591905	5.311626053	1.01E-49	5.11E-47	Down
GLT1D1	-4.599037753	2.076877027	3.83E-33	3.63E-31	Down
KCNJ3	-4.602665775	2.506096003	1.62E-22	5.67E-21	Down
AL021395.1	-4.605539343	-0.13091111	7.93E-19	1.91E-17	Down
CHRNA2	-4.607676667	-0.572793569	1.02E-21	3.28E-20	Down
LINC0094(	-4.609949535	-1.238477672	3.18E-24	1.30E-22	Down
FAM153A	-4.610984527	-0.481694042	5.82E-20	1.57E-18	Down
AL049775.1	-4.616182329	-1.341486312	3.60E-28	2.08E-26	Down
CALN1	-4.616683221	3.320658322	4.07E-29	2.59E-27	Down
ALML3-AS	-4.619737756	-3.23301699	1.93E-17	4.06E-16	Down
SNAP25	-4.619847575	7.412669248	1.25E-28	7.54E-27	Down
KRT31	-4.621270116	-2.838277225	2.22E-13	3.02E-12	Down
NEFL	-4.62231472	5.8765641	2.51E-17	5.21E-16	Down
RFPL1S	-4.623342349	2.612516224	9.01E-33	8.08E-31	Down
SH2D5	-4.62591463	2.394010201	2.92E-41	6.25E-39	Down
AC004817.4	-4.626585833	-1.964875965	1.47E-17	3.13E-16	Down
KCNJ4	-4.627382402	2.96279652	3.03E-34	3.24E-32	Down
PNPLA5	-4.627588777	-2.178300375	1.48E-21	4.69E-20	Down
LHFPL5	-4.62886198	-1.985391837	1.66E-21	5.23E-20	Down
TPH2	-4.634289237	-2.767169601	1.86E-25	8.52E-24	Down
SCN3B	-4.634821506	5.181144181	1.30E-37	1.87E-35	Down
NPTX1	-4.641669334	5.839979263	1.09E-26	5.57E-25	Down
TMEM155	-4.643112495	1.851995648	2.36E-35	2.75E-33	Down

SHANK1	-4.644275479	3.92722357	1.12E-32	9.92E-31	Down
TCERG1L	-4.645280092	-0.262298961	1.44E-19	3.72E-18	Down
GPR22	-4.646106751	0.850448421	6.46E-22	2.12E-20	Down
PPP2R2C	-4.646871501	4.983471506	6.40E-30	4.41E-28	Down
AC004147.	-4.649516814	-3.750796733	2.65E-13	3.58E-12	Down
AC105219.	-4.650672839	-2.984168205	4.11E-26	2.00E-24	Down
LINC0178	-4.652700913	-2.278250396	5.96E-13	7.72E-12	Down
NKRD20A	-4.652856968	-2.527969199	1.09E-25	5.07E-24	Down
LINC0288	-4.654054528	-2.60644956	9.27E-19	2.21E-17	Down
FSTL5	-4.656256935	1.106842735	9.88E-25	4.25E-23	Down
KCNT1	-4.656995193	2.502672663	4.63E-24	1.88E-22	Down
HS6ST3	-4.65748605	3.122615164	4.92E-28	2.81E-26	Down
ANO3	-4.674134211	1.719893114	1.16E-33	1.16E-31	Down
CELF4	-4.675827068	4.14076739	5.12E-25	2.27E-23	Down
CALY	-4.682107139	3.263876591	5.20E-21	1.55E-19	Down
TRPC5	-4.68507475	-1.355110957	4.22E-26	2.04E-24	Down
SLC7A4	-4.689633579	0.001594669	9.28E-31	6.93E-29	Down
AL161734.	-4.696017762	-3.297055901	6.33E-22	2.08E-20	Down
FCER2	-4.696131185	-0.561926738	1.29E-17	2.75E-16	Down
RIMBP2	-4.697100295	3.144134261	3.38E-34	3.60E-32	Down
BHLHA9	-4.704189199	-3.258962694	7.81E-18	1.71E-16	Down
PTER	-4.708289269	3.186239628	2.90E-47	1.10E-44	Down
SYT13	-4.710407808	4.133237993	7.65E-23	2.74E-21	Down
HSD3BP4	-4.716448125	-3.743705234	2.79E-16	5.26E-15	Down
HRH3	-4.722583675	1.589299879	2.21E-20	6.17E-19	Down
AC110774.	-4.72263209	-2.990697313	1.30E-22	4.59E-21	Down
SOHLH1	-4.725953233	-0.357975197	5.10E-18	1.13E-16	Down
EMX1	-4.736138364	0.907695023	1.55E-26	7.80E-25	Down
TMEM235	-4.737021735	2.06785642	1.19E-21	3.80E-20	Down
AC016687.	-4.737189527	-4.009657342	7.06E-11	7.14E-10	Down
OLFM3	-4.739671743	1.690358136	1.58E-24	6.65E-23	Down
HTR5A-AS	-4.744008336	-0.382588241	3.87E-16	7.17E-15	Down
CNNM1	-4.749652354	1.685772373	4.27E-27	2.25E-25	Down
PTPRD-AS	-4.750108263	-3.274064467	2.46E-20	6.82E-19	Down
PHYHIP	-4.752027824	5.645554547	3.64E-36	4.66E-34	Down
SLC32A1	-4.75456102	2.285490993	2.63E-21	8.04E-20	Down
TMEM88B	-4.755103162	-0.532339246	2.29E-30	1.64E-28	Down
AL049651.	-4.758774835	-3.647045399	2.95E-15	4.86E-14	Down
FSTL4	-4.764177382	1.5584918	1.42E-30	1.04E-28	Down
HTR2A	-4.764736497	2.295272137	1.17E-30	8.70E-29	Down

FAM153CP	-4.767612083	-1.191713674	4.27E-20	1.16E-18	Down
KCNA4	-4.769813226	-0.060755542	1.21E-23	4.71E-22	Down
HOOK1	-4.774260542	0.708409868	8.99E-33	8.08E-31	Down
GAD2	-4.77722201	3.084960117	9.94E-23	3.54E-21	Down
SYNPR	-4.786820203	3.376824758	1.05E-20	3.04E-19	Down
LY86-AS1	-4.78942664	0.256807405	3.95E-22	1.32E-20	Down
KLK5	-4.795353463	-2.322443269	5.25E-13	6.86E-12	Down
HTR5A	-4.804282555	0.242172925	1.29E-19	3.35E-18	Down
AC004817.[illegible]	-4.806570462	-3.625136758	1.32E-20	3.77E-19	Down
CUX2	-4.80708439	2.186607506	7.67E-24	3.05E-22	Down
SYCE1	-4.809110934	-1.995188234	3.68E-25	1.65E-23	Down
SSTR4	-4.810910658	-3.123092451	5.90E-15	9.46E-14	Down
MIR770	-4.815988772	-0.550001726	3.69E-23	1.36E-21	Down
MAP7D2	-4.817983746	2.992077547	5.98E-34	6.26E-32	Down
U62631.1	-4.820442598	-2.677762495	4.10E-24	1.67E-22	Down
PCLO	-4.827776862	3.536879878	1.92E-34	2.09E-32	Down
AL591501.1	-4.82800814	-3.801008467	3.92E-15	6.40E-14	Down
AK5	-4.828315692	5.043296741	3.39E-31	2.67E-29	Down
GRIN3A	-4.830590426	1.548078823	1.01E-54	8.30E-52	Down
PTGER3	-4.836052672	-0.020726619	1.52E-28	9.14E-27	Down
RASGRF1	-4.836358033	3.543482471	3.66E-37	5.04E-35	Down
MTUS2	-4.838148911	0.819914091	1.17E-42	2.82E-40	Down
KCNS2	-4.840450412	0.663288811	7.53E-25	3.27E-23	Down
AL109946.1	-4.840972879	-1.887778192	3.55E-17	7.22E-16	Down
FAM163B	-4.845653672	3.017638968	3.71E-22	1.25E-20	Down
TBR1	-4.85430888	2.268658041	1.60E-29	1.06E-27	Down
UNC13C	-4.862174686	1.734908335	1.53E-24	6.48E-23	Down
LINC0110[illegible]	-4.873109288	-1.469395243	2.12E-24	8.83E-23	Down
LINC0137[illegible]	-4.876163951	-3.091363546	4.33E-14	6.37E-13	Down
GABRG2	-4.879023252	3.653675709	1.91E-20	5.37E-19	Down
GABRA4	-4.889780416	2.439024235	6.46E-26	3.09E-24	Down
KCNH5	-4.891408032	0.633480712	1.56E-24	6.60E-23	Down
RTN4RL1	-4.892416007	1.84345937	9.35E-36	1.15E-33	Down
AP005242.[illegible]	-4.893199914	-2.073638286	4.46E-12	5.25E-11	Down
GRIN2A	-4.895228106	3.762967843	3.07E-31	2.43E-29	Down
ICAM5	-4.897093014	3.445733601	1.41E-39	2.58E-37	Down
RAB3C	-4.901379076	4.214451245	4.72E-29	2.98E-27	Down
AC015712.[illegible]	-4.903194941	-2.995424544	1.16E-16	2.26E-15	Down
LINC0260[illegible]	-4.904092564	0.538354013	6.68E-18	1.47E-16	Down
GALNTL6	-4.92698609	-0.07333829	1.42E-48	6.10E-46	Down

LOXHD1	-4.927863815	-2.214977414	1.32E-44	3.87E-42	Down
GPR83	-4.929539415	0.988324452	2.28E-29	1.48E-27	Down
IRREL3-A:	-4.932381393	-3.519769887	3.17E-18	7.16E-17	Down
SLC22A10	-4.939304447	-3.362081199	1.02E-29	6.94E-28	Down
KCNB2	-4.940362452	-0.795314655	3.33E-18	7.48E-17	Down
LINC0050	-4.941177111	-0.169098782	2.23E-14	3.37E-13	Down
LINC0120	-4.943200106	-1.980960148	3.86E-12	4.58E-11	Down
CPNE6	-4.947488983	3.978237735	1.09E-25	5.07E-24	Down
CPLX2	-4.955031498	6.316000769	6.02E-25	2.65E-23	Down
RHDE-AS	-4.957268367	-1.018433626	1.13E-22	4.02E-21	Down
LINC0221	-4.961313496	-2.23379256	2.06E-19	5.23E-18	Down
EPHA6	-4.963656631	-0.819599201	1.98E-21	6.19E-20	Down
SHISAL1	-4.969225293	3.448716139	7.04E-30	4.82E-28	Down
AC096570.	-4.971174116	-4.030616986	6.47E-12	7.45E-11	Down
LINC01511	-4.978702645	-1.532022916	8.10E-19	1.94E-17	Down
LINC0128	-4.984260095	-3.899172439	1.93E-18	4.45E-17	Down
VSNL1	-4.98431803	5.790748818	2.43E-25	1.10E-23	Down
AC126177.	-4.991940779	-3.286450865	1.13E-17	2.44E-16	Down
GRM5	-4.992691251	1.0347223	1.55E-27	8.50E-26	Down
LINC0116	-4.995726974	-1.913419801	1.05E-41	2.33E-39	Down
SLC22A9	-4.997275907	-3.275661455	2.51E-20	6.95E-19	Down
FRMPD2B	-5.002118375	-1.883876362	8.71E-37	1.17E-34	Down
DRD1	-5.003124144	0.712153403	2.01E-30	1.45E-28	Down
PHF24	-5.003964264	4.162933502	1.97E-37	2.78E-35	Down
CCKBR	-5.019228946	1.350404754	2.01E-26	1.00E-24	Down
WNT10B	-5.019678517	1.108446701	5.70E-35	6.47E-33	Down
AC015819.	-5.020430376	-2.67343113	2.56E-15	4.25E-14	Down
AL035696.	-5.037634926	-2.677237218	1.11E-18	2.64E-17	Down
NPAS4	-5.041206843	-0.22040608	4.13E-31	3.21E-29	Down
SSTR1	-5.050055017	1.647519652	1.19E-22	4.21E-21	Down
AC037441.	-5.052800811	-2.00070486	2.56E-21	7.87E-20	Down
CACNA1I	-5.075018839	1.955476334	1.97E-38	3.19E-36	Down
AP003355.	-5.081386686	-0.103577606	5.25E-20	1.42E-18	Down
RBFOX3	-5.081995708	3.231116175	2.96E-29	1.90E-27	Down
AC132825.	-5.082372416	-1.346285947	1.19E-56	1.18E-53	Down
PPP4R4	-5.084071304	2.143975188	2.28E-44	6.63E-42	Down
FBXO40	-5.084231289	-2.042975093	1.28E-39	2.37E-37	Down
AC134312.	-5.086336489	-0.366212838	1.21E-36	1.60E-34	Down
MT-TF	-5.08997571	-1.977079661	2.62E-48	1.09E-45	Down
CHRM1	-5.093743583	3.000156726	1.85E-29	1.21E-27	Down

SYN2	-5.11032549	5.778770886	4.14E-39	7.23E-37	Down
MYT1L	-5.112651408	3.702696308	1.86E-26	9.33E-25	Down
TMEM130	-5.114697831	5.119165748	4.09E-33	3.85E-31	Down
EIF4E1B	-5.117191461	-1.456502435	4.11E-24	1.67E-22	Down
RXFP1	-5.12171524	0.626903672	1.04E-36	1.39E-34	Down
HTR1E	-5.124116994	-1.640821807	7.47E-19	1.80E-17	Down
LRTM2	-5.126411506	1.670094919	8.38E-25	3.61E-23	Down
SLC30A3	-5.143755388	2.885169973	3.71E-38	5.75E-36	Down
SYT1	-5.149402368	6.47551069	3.97E-32	3.38E-30	Down
LC26A4-AS	-5.152576539	2.035467958	6.98E-32	5.81E-30	Down
SLC17A7	-5.154031402	6.613836377	5.01E-23	1.83E-21	Down
AC134312.3	-5.156513219	-3.113646275	6.12E-26	2.93E-24	Down
AL162457.2	-5.157147178	-3.116237188	1.33E-18	3.14E-17	Down
AC116456.1	-5.1620349	-2.611033184	2.27E-23	8.54E-22	Down
NEFM	-5.174995483	4.320659544	3.62E-22	1.22E-20	Down
GDA	-5.180710145	4.116112427	9.23E-31	6.91E-29	Down
WSCD2	-5.180917825	2.018175578	8.23E-27	4.24E-25	Down
ANKRD34	-5.18360779	-1.810744598	2.50E-30	1.79E-28	Down
GABRB2	-5.194917144	3.505975462	4.83E-32	4.10E-30	Down
GPR26	-5.198367027	-0.049556822	6.33E-19	1.53E-17	Down
KRT222	-5.204898264	-0.095906148	1.60E-23	6.15E-22	Down
RBFOX1	-5.214846419	3.743090167	4.34E-31	3.36E-29	Down
SV2C	-5.215831378	2.085819797	1.31E-40	2.63E-38	Down
GABRA1	-5.236089867	3.840596964	3.02E-23	1.12E-21	Down
SULT4A1	-5.238668663	4.351071183	2.42E-31	1.93E-29	Down
KCNC2	-5.239674751	2.428335772	9.19E-26	4.32E-24	Down
STYK1	-5.258931157	0.140401172	1.49E-31	1.21E-29	Down
SLC12A5	-5.263854137	4.656518267	3.98E-54	2.87E-51	Down
LINC0039	-5.275759573	-3.462212788	2.56E-28	1.51E-26	Down
CAMK2A	-5.276418499	6.467662336	9.91E-34	1.01E-31	Down
AC099684.1	-5.277462438	-1.826212164	2.61E-21	8.00E-20	Down
OPALIN	-5.280378693	4.060175867	1.21E-19	3.14E-18	Down
LINC01821	-5.287653449	-3.392487669	2.75E-15	4.56E-14	Down
SERTM1	-5.288252353	1.283857659	3.22E-27	1.72E-25	Down
SLC6A17	-5.296426776	4.659861242	1.22E-34	1.37E-32	Down
SSTR3	-5.321075466	0.133782748	1.54E-35	1.82E-33	Down
GPR52	-5.349299606	-3.070472283	9.39E-28	5.21E-26	Down
TESPA1	-5.351125581	2.231157983	4.51E-39	7.68E-37	Down
CREG2	-5.35297313	4.192957328	3.12E-38	4.91E-36	Down
CALHM1	-5.359128541	-1.382459383	2.72E-43	6.88E-41	Down

MAL2	-5.367943933	2.493721899	3.71E-27	1.97E-25	Down
AL353746.1	-5.368154413	-0.419669724	2.73E-26	1.35E-24	Down
SLC8A2	-5.372488956	3.998013285	2.21E-37	3.09E-35	Down
C4orf50	-5.381251755	-0.261116619	6.20E-37	8.39E-35	Down
DRD5	-5.382005374	-1.447859293	2.21E-23	8.31E-22	Down
AC103855.2	-5.386157283	-2.741109846	4.01E-23	1.47E-21	Down
HIPK4	-5.390826764	0.127612055	7.86E-49	3.48E-46	Down
SST	-5.392545474	2.442275213	1.72E-32	1.51E-30	Down
HCN1	-5.393789857	1.704001706	9.80E-29	6.01E-27	Down
AGBL1	-5.39478796	-2.565864005	2.83E-28	1.65E-26	Down
FRMPD4	-5.397230876	1.928865501	4.73E-36	5.97E-34	Down
CACNA1B	-5.397462171	2.778882849	8.84E-33	7.98E-31	Down
LINC02346	-5.413559884	-2.772880938	1.91E-18	4.41E-17	Down
OPRM1	-5.422232898	-3.502687051	5.62E-32	4.73E-30	Down
DLGAP2	-5.426562803	1.625297867	5.54E-37	7.52E-35	Down
RASAL1	-5.428630017	2.925739407	1.64E-36	2.15E-34	Down
AC104024.2	-5.430971562	-0.012913518	8.87E-22	2.88E-20	Down
CDH9	-5.436591489	0.082419379	7.86E-29	4.87E-27	Down
PROKR2	-5.438822945	-2.949734534	2.04E-19	5.19E-18	Down
MPPED1	-5.443254376	2.703806243	2.24E-35	2.63E-33	Down
AC009487.3	-5.446377932	-3.641945086	1.43E-23	5.53E-22	Down
SVOP	-5.446465359	3.527475827	6.30E-33	5.86E-31	Down
SOWAHB	-5.450553454	-0.276272953	2.53E-52	1.49E-49	Down
ATP2B3	-5.457565219	2.76241938	6.34E-40	1.20E-37	Down
AC110491.1	-5.466219931	-0.627191364	1.01E-25	4.74E-24	Down
HTR1A	-5.473187733	-1.467790146	3.81E-19	9.45E-18	Down
PACSIN1	-5.474537439	5.185159535	7.43E-32	6.13E-30	Down
BX276092.7	-5.492371614	-3.318316299	1.45E-33	1.44E-31	Down
CACNG3	-5.495521324	2.541198792	6.58E-25	2.89E-23	Down
FADS6	-5.510542488	-1.152898415	1.65E-31	1.33E-29	Down
ATP8A2	-5.511481568	3.321607887	9.79E-42	2.20E-39	Down
GABRA5	-5.52563821	3.204068599	9.84E-29	6.02E-27	Down
LINC01331	-5.529035356	-3.435947824	2.46E-30	1.76E-28	Down
GPR6	-5.552520864	-1.524197112	4.91E-19	1.21E-17	Down
DDN	-5.554390189	4.41658985	1.48E-40	2.94E-38	Down
HTR2C	-5.596281636	-0.372586653	2.25E-30	1.62E-28	Down
KLHL1	-5.59703085	-1.093310466	3.53E-25	1.59E-23	Down
MAS1	-5.603602031	-0.763565188	3.90E-32	3.35E-30	Down
SLC6A7	-5.606352402	1.865036963	3.63E-29	2.31E-27	Down
CHD5	-5.61092967	4.39040832	7.91E-41	1.64E-38	Down

OR14I1	-5.611623987	-2.899880087	9.90E-20	2.61E-18	Down
AC008568.1	-5.612569039	-3.241741154	2.73E-28	1.60E-26	Down
AC018358.1	-5.625689369	-1.827650912	7.02E-24	2.81E-22	Down
AC009487.2	-5.637205061	-3.517887508	5.55E-25	2.45E-23	Down
TMEM132D	-5.642274227	1.587815034	1.27E-30	9.44E-29	Down
KIF12	-5.646770666	-1.621348718	5.87E-30	4.06E-28	Down
SV2B	-5.653671299	4.712160282	2.49E-34	2.70E-32	Down
HTR3B	-5.653875304	-2.083345481	3.10E-25	1.40E-23	Down
KCNV1	-5.661255128	1.440642799	7.97E-25	3.44E-23	Down
KLK7	-5.66281305	0.432066911	8.12E-20	2.15E-18	Down
TRHDE	-5.670329255	0.943727282	6.51E-34	6.80E-32	Down
GLP2R	-5.711889725	0.641805928	3.38E-45	1.05E-42	Down
AP001993.1	-5.724251455	-2.865524156	2.87E-21	8.72E-20	Down
KRD34C-A	-5.726704606	-2.774207431	7.22E-24	2.89E-22	Down
LINC0161(	-5.752583886	-1.479480874	3.49E-31	2.74E-29	Down
AC073525.1	-5.798348278	-2.584008593	4.38E-33	4.11E-31	Down
AP005901.3	-5.817025294	-2.778493746	1.63E-34	1.80E-32	Down
GRIN1	-5.827624466	5.308662833	5.44E-32	4.59E-30	Down
CD177P1	-5.830254006	-3.654852849	3.65E-21	1.10E-19	Down
CNGB1	-5.851426692	-0.084168777	6.66E-33	6.17E-31	Down
PNMA5	-5.884573362	0.66504665	8.41E-41	1.73E-38	Down
GRIN2B	-5.900702807	3.729928356	1.98E-50	1.02E-47	Down
C1QL3	-5.912076603	1.842633791	2.10E-38	3.37E-36	Down
NWD2	-5.928184507	0.325524308	4.41E-39	7.57E-37	Down
MCHR2	-5.971578335	-0.842436764	8.57E-24	3.39E-22	Down
AC011995.2	-5.972569291	-2.258958829	1.02E-40	2.08E-38	Down
RYR2	-5.977058817	2.8139244	8.41E-55	7.13E-52	Down
SYT10	-5.987570649	-2.856778311	2.37E-27	1.29E-25	Down
PRKCG	-5.998284004	3.456167929	1.26E-49	6.28E-47	Down
PNMA6F	-6.001892734	-0.164388656	3.93E-26	1.92E-24	Down
GSG1L2	-6.094759208	-3.001053212	1.65E-23	6.30E-22	Down
Z68323.1	-6.097618809	-3.178395994	9.18E-16	1.61E-14	Down
NEUROD(	-6.22888297	0.568970589	2.28E-33	2.20E-31	Down
AC007922.3	-6.350982893	-3.22615029	3.24E-17	6.64E-16	Down
LINC0147(	-6.394319746	-2.684982111	6.48E-26	3.09E-24	Down
SLC22A8	-6.746267689	-2.386283237	1.09E-35	1.31E-33	Down
LINC0100	-7.205387272	-1.065613571	6.91E-31	5.23E-29	Down

Gene_sym	adj.P.Val	P.Value	logFC
SPP1	4.66E-08	2.17E-12	1.07E+01
S100P	4.66E-08	2.19E-12	-9.9
MARC1	4.66E-08	2.79E-12	-8.95
WISP2	4.66E-08	4.23E-12	-9.01
UCHL1	4.66E-08	4.64E-12	1.06E+01
NEFL	4.66E-08	6.33E-12	9.21
CDKN2A	4.66E-08	8.40E-12	-8
STEAP4	4.66E-08	8.69E-12	-9.47
COL1A2	4.66E-08	9.54E-12	1.00E+01
MIF	4.66E-08	1.01E-11	-9.05
IL13RA2	4.66E-08	1.04E-11	8.38
KIF26A	4.66E-08	1.13E-11	-7.63
PRSS21	4.66E-08	1.27E-11	-7.3
MYPN	4.66E-08	1.28E-11	-7.87
MOCOS	4.66E-08	1.34E-11	-7.65
KYNU	4.66E-08	1.53E-11	-8.42
CAPS	4.66E-08	1.68E-11	8.27
SERPINB	4.66E-08	1.76E-11	-8.94
TENM2	4.66E-08	1.76E-11	9.54
C19orf33	4.66E-08	1.80E-11	-8.42
C11orf86	4.66E-08	1.80E-11	-7.44
ALPP	4.66E-08	1.94E-11	-8.9
C4BPB	4.66E-08	2.00E-11	-1.13E+01
CXorf61	4.66E-08	2.10E-11	-6.94
TRIM29	4.68E-08	2.37E-11	-7.1
SPARC	4.68E-08	2.41E-11	9.07
H19	4.68E-08	2.66E-11	-1.42E+01
DUSP23	4.68E-08	2.67E-11	-7.4
SEMA3A	4.68E-08	2.69E-11	9.07
SPAG17	4.68E-08	2.76E-11	7.58
PABPC4L	4.68E-08	2.80E-11	-6.7
SLC27A2	4.68E-08	2.81E-11	-8.06
CDKN2C	4.73E-08	3.09E-11	-1.03E+01
SLC43A3	4.73E-08	3.12E-11	-8.69
EPHA3	4.73E-08	3.17E-11	9.67
BMP2	4.73E-08	3.23E-11	-8.25
SLPI	4.73E-08	3.27E-11	-8.94
HSPA1A	5.31E-08	3.82E-11	-8.82
CNR1	5.31E-08	3.97E-11	7.4

PSCA	5.31E-08	4.08E-11	-6.66
BASP1	5.31E-08	4.26E-11	-7.5
C1S	5.31E-08	4.26E-11	-6.94
previous v	5.70E-08	4.67E-11	7.55
KRT17	5.73E-08	4.80E-11	-9.97
PLAU	5.77E-08	4.93E-11	9.56
GCHFR	6.09E-08	5.32E-11	-7.1
RBM24	6.13E-08	5.63E-11	8.65
NPTX1	6.13E-08	5.82E-11	-6.52
ANXA8|A	6.13E-08	5.90E-11	-6.28
RP3-428L1	6.13E-08	6.16E-11	7.25
CITED4	6.13E-08	6.18E-11	-5.99
COL5A2	6.13E-08	6.21E-11	7.35
SOX2	6.13E-08	6.34E-11	1.11E+01
RP11-119I	6.13E-08	6.40E-11	-6.62
SVEP1	6.13E-08	6.52E-11	-6.33
PRKCDBI	6.13E-08	6.62E-11	-6.33
LINC0046	6.13E-08	6.62E-11	8.91
HTATIP2	6.13E-08	6.70E-11	-8.28
FABP5	6.13E-08	6.84E-11	-7.47
DIAPH2	6.13E-08	7.01E-11	7.32
SCHIP1	6.13E-08	7.10E-11	6.79
MLLT11	6.13E-08	7.43E-11	7.45
HDAC9	6.13E-08	7.53E-11	8
NA	6.13E-08	7.58E-11	-5.88
WFDC1	6.13E-08	7.59E-11	-6.72
SYT11	6.13E-08	7.71E-11	7.72
ADD2	6.43E-08	8.20E-11	6.85
RAB17	6.47E-08	8.58E-11	-7.46
ZYG11A	6.47E-08	8.72E-11	-6.14
MAGEB2	6.47E-08	8.79E-11	-5.98
STK11	6.47E-08	8.92E-11	7.03
TES	6.47E-08	9.01E-11	-8.37
GDA	6.47E-08	9.18E-11	-6.66
CNN3	6.47E-08	9.29E-11	9.16
GLIS3	6.47E-08	9.30E-11	7.81
SOX2-OT	6.47E-08	9.31E-11	6.81
NRN1	6.66E-08	9.91E-11	9.37
CDH4	6.66E-08	9.94E-11	6.93
EFNB2	6.66E-08	9.94E-11	7.55

AQP3	6.68E-08	1.01E-10	-6.9
SERPINB	6.72E-08	1.04E-10	-8.16
LINC0047	6.76E-08	1.08E-10	-7.17
CYP11A1	6.76E-08	1.08E-10	-6.07
RPP25	6.76E-08	1.10E-10	-6.32
DCN	6.76E-08	1.11E-10	-7.91
SFN	6.76E-08	1.11E-10	-5.9
AUTS2	6.76E-08	1.12E-10	9.38
LUM	6.76E-08	1.13E-10	-8.09
MDK	6.76E-08	1.15E-10	5.47
FGF12	7.00E-08	1.21E-10	6.08
IGF2BP1	7.00E-08	1.25E-10	-6.41
PAGE1	7.00E-08	1.25E-10	-6.03
KIR2DL2	7.00E-08	1.25E-10	-6.71
PLEKHG	7.00E-08	1.26E-10	9.03
IGFBP2	7.00E-08	1.26E-10	9.83
HENMT1	7.29E-08	1.33E-10	-7.31
SOX9	7.59E-08	1.40E-10	6.9
STAT6	7.63E-08	1.42E-10	-5.83
RARRES2	7.63E-08	1.43E-10	-5.48
GFPT2	7.79E-08	1.48E-10	6.49
CHCHD1(	8.11E-08	1.56E-10	-9.24
GAL	8.18E-08	1.60E-10	-6.01
GLIPR1	8.18E-08	1.61E-10	6.53
TMPRSS3	8.21E-08	1.64E-10	-5.38
C3	8.21E-08	1.66E-10	-5.79
FABP7	8.21E-08	1.66E-10	8.35
SLC38A5	8.27E-08	1.69E-10	-6.1
SLC16A2	8.27E-08	1.70E-10	5.57
TINCR	8.60E-08	1.82E-10	-5.41
CT45A2|(	8.60E-08	1.84E-10	8.19
TMEM15‹	8.60E-08	1.85E-10	6.3
EPB41L3	8.63E-08	1.87E-10	-5.94
GPR87	8.74E-08	1.92E-10	-5.2
EPS8L2	8.74E-08	1.93E-10	-6.55
GALM	8.86E-08	1.97E-10	-6.42
VWA5A	8.90E-08	1.99E-10	-5.22
ZNF518B	9.03E-08	2.07E-10	6.15
C3orf52	9.03E-08	2.11E-10	-5.52
PSTPIP2	9.03E-08	2.14E-10	-5.51

KCTD14	9.03E-08	2.15E-10	-6.01
NMRAL1	9.03E-08	2.15E-10	-5.77
DMKN	9.03E-08	2.16E-10	-6.09
PTGES	9.03E-08	2.20E-10	-5.17
CPNE8	9.03E-08	2.21E-10	-6.39
NPNT	9.03E-08	2.23E-10	-7.26
PTN	9.03E-08	2.27E-10	9.15
ENOSF1	9.03E-08	2.28E-10	-6.51
NRP2	9.03E-08	2.29E-10	5.24
DPYSL3	9.23E-08	2.35E-10	5.33
VCAN	9.24E-08	2.38E-10	8.46
KIR3DL2	9.24E-08	2.40E-10	-5.66
TESC	9.24E-08	2.41E-10	-5.35
KIR2DL2	9.38E-08	2.46E-10	-5.29
PDE4DIP	9.38E-08	2.48E-10	5.92
MOXD1	9.52E-08	2.53E-10	7.09
THY1	9.73E-08	2.61E-10	5.82
TMSB15B	9.79E-08	2.64E-10	7.65
GPR37	9.83E-08	2.67E-10	-5.29
FAM198B	9.83E-08	2.68E-10	6.34
KIR2DL5	9.84E-08	2.73E-10	-7.52
SOCS2	9.84E-08	2.76E-10	7.85
TPD52L1	9.84E-08	2.78E-10	-4.95
LINC0097	9.84E-08	2.80E-10	7.5
ZFHX4	9.84E-08	2.82E-10	6.91
PDE1C	9.84E-08	2.83E-10	5.49
DENND2	9.84E-08	2.85E-10	7.45
PRRX1	9.84E-08	2.85E-10	5.99
IFITM1	9.98E-08	2.91E-10	-5.52
GLIPR2	1.01E-07	2.99E-10	5.93
DACT1	1.01E-07	2.99E-10	5.83
AMPD1	1.01E-07	3.02E-10	6.64
FILIP1	1.01E-07	3.04E-10	5.68
CH17-360	1.01E-07	3.05E-10	-5.87
ENPP4	1.01E-07	3.08E-10	7.78
FAM24B	1.01E-07	3.09E-10	-5.2
TNC	1.01E-07	3.11E-10	8.32
PLAC8	1.01E-07	3.11E-10	-6.57
GRAMD1	1.01E-07	3.12E-10	-5
STON1-G	1.02E-07	3.20E-10	6.67

MAGED4	1.03E-07	3.27E-10	6.59
NES	1.03E-07	3.27E-10	7.8
RHOJ	1.04E-07	3.33E-10	6.93
CDA	1.04E-07	3.37E-10	-9.9
EPGN	1.06E-07	3.44E-10	-5.63
COL6A2	1.07E-07	3.52E-10	6.1
C7orf29	1.07E-07	3.53E-10	-5.94
GJA1	1.07E-07	3.55E-10	1.06E+01
FAP	1.07E-07	3.57E-10	-6.56
PTPN3	1.12E-07	3.75E-10	-5.6
KIR2DL5	1.12E-07	3.77E-10	-6.14
PCSK9	1.13E-07	3.84E-10	-5.75
NLGN1	1.13E-07	3.85E-10	7.92
NPY1R	1.14E-07	3.89E-10	8.19
PDGFC	1.15E-07	3.97E-10	6.55
POU3F2	1.15E-07	3.99E-10	6.04
ME1	1.17E-07	4.10E-10	-5.25
DIRAS3	1.18E-07	4.18E-10	6.16
PAQR5	1.18E-07	4.19E-10	-6.5
KIR2DL3	1.20E-07	4.29E-10	-5.16
PDE4DIP	1.21E-07	4.32E-10	5.28
LXN	1.22E-07	4.41E-10	-5.95
SIGIRR	1.25E-07	4.53E-10	-6.76
ZNF22	1.25E-07	4.56E-10	8.12
LYPD1	1.25E-07	4.58E-10	9.9
DYNC1I1	1.27E-07	4.73E-10	6.73
RUNX2	1.29E-07	4.80E-10	5.19
ITGA4	1.29E-07	4.89E-10	5.34
ZC4H2	1.29E-07	4.91E-10	5.1
MFAP5	1.29E-07	4.92E-10	-6.91
GFAP	1.29E-07	4.93E-10	8.9
MSL3P1	1.29E-07	4.95E-10	-5.16
ZNF831	1.29E-07	4.97E-10	-4.72
EREG	1.30E-07	5.09E-10	-8.69
JAM3	1.30E-07	5.15E-10	8.61
B4GALNT	1.30E-07	5.15E-10	4.69
GPC5	1.30E-07	5.17E-10	-4.65
C1orf51	1.30E-07	5.21E-10	5.87
OAS1	1.33E-07	5.34E-10	-4.87
PTPRG	1.34E-07	5.41E-10	6.69

MUC3A	1.35E-07	5.49E-10	-5.79
SRPX	1.40E-07	5.71E-10	6.33
NCAM1	1.40E-07	5.76E-10	7.23
LINC0102	1.40E-07	5.80E-10	-5.58
KRT7	1.41E-07	5.84E-10	-7.62
MSLN	1.43E-07	5.95E-10	-5.41
L1CAM	1.44E-07	6.06E-10	-5.38
SV2A	1.44E-07	6.13E-10	6.34
MAPK4	1.44E-07	6.14E-10	-5.01
AMOT	1.44E-07	6.15E-10	7.03
OLR1	1.45E-07	6.20E-10	-7.09
KIR2DL2	1.46E-07	6.26E-10	-4.54
RARB	1.49E-07	6.41E-10	9.44
FBXO17	1.49E-07	6.49E-10	5.26
SFRP1	1.49E-07	6.50E-10	5.5
FOXG1	1.49E-07	6.55E-10	5.22
PCDH20	1.49E-07	6.56E-10	5.25
PKDCC	1.49E-07	6.58E-10	-4.63
CDH11	1.51E-07	6.70E-10	7.11
CADM1	1.54E-07	6.87E-10	6.25
RP11-49I1	1.54E-07	6.88E-10	-6.09
MKX	1.57E-07	7.05E-10	-6.46
IDS	1.57E-07	7.05E-10	4.94
MGMT	1.57E-07	7.12E-10	-6.66
RP11-1149	1.57E-07	7.13E-10	-4.84
BASP1P1	1.57E-07	7.14E-10	-5.76
CSGALNA	1.57E-07	7.19E-10	-4.73
PTHLH	1.60E-07	7.40E-10	6.43
CT45A2|C	1.60E-07	7.43E-10	7.61
MAGI2-A	1.60E-07	7.50E-10	4.97
KRT8	1.60E-07	7.51E-10	-4.92
PDGFD	1.62E-07	7.67E-10	5.81
TMEM161	1.62E-07	7.70E-10	5.21
WNT7B	1.62E-07	7.71E-10	-5.33
PBDC1	1.62E-07	7.71E-10	7.71
TNS4	1.62E-07	7.78E-10	-4.56
LOC10100	1.70E-07	8.22E-10	4.71
CPS1	1.70E-07	8.22E-10	-6.54
COL11A1	1.71E-07	8.26E-10	5.39
C1QTNF6	1.73E-07	8.41E-10	-5.36

C1R	1.73E-07	8.44E-10	-5.56
FILIP1L	1.73E-07	8.46E-10	5.06
TNS1	1.73E-07	8.49E-10	-5.22
TNFRSF1	1.73E-07	8.53E-10	-4.5
CTNND2	1.74E-07	8.61E-10	5.12
CPA6	1.74E-07	8.64E-10	5.09
UNC13D	1.74E-07	8.68E-10	-4.43
BOLA1	1.75E-07	8.77E-10	-6.12
LINC0102	1.75E-07	8.85E-10	-4.58
IFI27	1.77E-07	8.94E-10	6.02
RP11-328(	1.77E-07	8.99E-10	6.89
CXCL14	1.78E-07	9.06E-10	8.75
PCDH17	1.80E-07	9.21E-10	7.73
ASS1	1.81E-07	9.34E-10	-5.7
DACH1	1.81E-07	9.34E-10	7.13
KIR2DS2|	1.82E-07	9.39E-10	-5.04
TIAM1	1.82E-07	9.42E-10	6.19
CFI	1.83E-07	9.51E-10	4.92
PDE8B	1.83E-07	9.58E-10	-5.25
LOC1010(	1.83E-07	9.67E-10	-4.72
ZNF268	1.83E-07	9.67E-10	7.14
ZIC1	1.84E-07	9.77E-10	6.71
EPHA5	1.85E-07	9.84E-10	5.98
AGPAT9	1.87E-07	9.99E-10	6.65
TNFSF10	1.87E-07	1.00E-09	6.48
SEMA6B	1.88E-07	1.02E-09	5.2
NDP	1.88E-07	1.02E-09	4.86
DTX4	1.88E-07	1.02E-09	-4.13
LINC0095	1.88E-07	1.03E-09	-6.02
C1orf114	1.88E-07	1.03E-09	5.39
CTD-2319	1.88E-07	1.03E-09	-4.5
COL15A1	1.90E-07	1.05E-09	-8.39
NRG4	1.92E-07	1.06E-09	-4.94
SOX3	1.92E-07	1.08E-09	8.13
ADAM19	1.92E-07	1.08E-09	5.22
CLDN7	1.92E-07	1.08E-09	-5.12
ZNF569	1.92E-07	1.08E-09	5.67
GEM	1.94E-07	1.10E-09	5.11
SMO	1.94E-07	1.10E-09	5.35
PDGFRA	1.94E-07	1.10E-09	5.66

FBXO32	1.94E-07	1.10E-09	5.77
CNRIP1	1.98E-07	1.13E-09	6.44
RP13-726I	2.00E-07	1.15E-09	-4.23
AFP	2.01E-07	1.16E-09	5.84
RARRES1	2.03E-07	1.17E-09	-5.05
SULF2	2.03E-07	1.18E-09	6.2
PIK3CG	2.04E-07	1.18E-09	4.44
C14orf169	2.04E-07	1.19E-09	-7.75
LINC0096	2.04E-07	1.19E-09	-4.29
CYFIP2	2.04E-07	1.19E-09	5.67
BVES	2.05E-07	1.20E-09	6.87
SOCS2-AS	2.06E-07	1.22E-09	5.7
RP11-119I	2.06E-07	1.22E-09	-4.46
CDKN1C	2.06E-07	1.22E-09	-4.36
KIR2DL1	2.06E-07	1.23E-09	-4.54
PIK3C2B	2.09E-07	1.25E-09	-4.41
GIPC3	2.10E-07	1.26E-09	-4.75
APBA2	2.11E-07	1.27E-09	6.75
RP11-119I	2.11E-07	1.27E-09	-5.91
LOC10012	2.11E-07	1.28E-09	-4.28
CXCR4	2.12E-07	1.29E-09	-5.37
RP11-542I	2.12E-07	1.29E-09	-4.05
ZP3	2.12E-07	1.31E-09	-4.34
PRKAG2	2.12E-07	1.31E-09	4.16
NFIX	2.12E-07	1.31E-09	7.01
EHBP1L1	2.13E-07	1.32E-09	-5.02
RHOF	2.13E-07	1.33E-09	-5.04
IQCG	2.14E-07	1.34E-09	6.37
CMTM3	2.16E-07	1.35E-09	4.27
WBSCR17	2.17E-07	1.36E-09	4.11
MAP1LC3	2.17E-07	1.36E-09	6.35
CNN1	2.17E-07	1.37E-09	5.81
SERPINE	2.19E-07	1.39E-09	6.5
PCDHGA	2.20E-07	1.40E-09	6.81
RP11-706(	2.20E-07	1.41E-09	-5.95
RP5-875H	2.20E-07	1.41E-09	7.14
NRP1	2.24E-07	1.43E-09	6.82
MPZL2	2.24E-07	1.45E-09	-4.15
TUBA1A	2.24E-07	1.45E-09	6.26
NRCAM	2.26E-07	1.46E-09	7.68

SMOC1	2.26E-07	1.47E-09	-4.72
QKI	2.27E-07	1.49E-09	5.48
C1QTNF1	2.28E-07	1.50E-09	-4.64
TIMP3	2.29E-07	1.50E-09	-6.01
MEIS2	2.29E-07	1.52E-09	5.36
LINC0046	2.30E-07	1.54E-09	4.52
ZNF697	2.30E-07	1.54E-09	5.41
LAMA4	2.30E-07	1.54E-09	8.16
BHLHE41	2.30E-07	1.55E-09	5.98
PRSS35	2.30E-07	1.55E-09	9.01
ZNF382	2.30E-07	1.55E-09	4.22
SOX2-OT	2.30E-07	1.56E-09	4.84
PRAME	2.30E-07	1.56E-09	-6.52
PTPRM	2.32E-07	1.58E-09	-6.23
TNFSF9	2.32E-07	1.59E-09	-4.56
RAB42	2.36E-07	1.61E-09	-4.4
C14orf23	2.38E-07	1.64E-09	7.65
AKT3	2.38E-07	1.64E-09	5.57
GJB3	2.38E-07	1.64E-09	-4.76
KIR3DL2	2.38E-07	1.65E-09	-4.47
LRCH2	2.38E-07	1.65E-09	7.97
EEF1A2	2.40E-07	1.67E-09	4.27
RP11-119I	2.42E-07	1.69E-09	6.48
SLC16A6	2.45E-07	1.73E-09	-4.62
NR2E1	2.45E-07	1.73E-09	6.06
PLEKHA5	2.46E-07	1.75E-09	-4.47
MYL9	2.47E-07	1.75E-09	-8.05
IFI30	2.48E-07	1.77E-09	-4.57
LYPD3	2.48E-07	1.77E-09	-4.69
CYBA	2.49E-07	1.78E-09	-4.74
ERAP2	2.50E-07	1.80E-09	5.51
PCLO	2.51E-07	1.81E-09	6.85
ACSS3	2.52E-07	1.83E-09	5.3
ENPP6	2.57E-07	1.87E-09	5.58
CAPG	2.57E-07	1.88E-09	-3.88
IGFBP3	2.61E-07	1.91E-09	-6.22
CDKN2B	2.62E-07	1.92E-09	-6.81
TLL1	2.62E-07	1.93E-09	-4.81
SLC12A3	2.63E-07	1.94E-09	-4.41
ENDOD1	2.65E-07	1.96E-09	6.42

APOBEC3	2.66E-07	1.97E-09	4.46
LINC0046	2.66E-07	1.97E-09	4.78
GMPR	2.66E-07	1.98E-09	4.33
KIR2DS2 |	2.68E-07	2.01E-09	-4.05
CTA-941F	2.68E-07	2.01E-09	-4.09
FOXL2	2.69E-07	2.01E-09	-4.67
COL6A1	2.76E-07	2.08E-09	4.67
KIF5C	2.76E-07	2.08E-09	5.52
EFNB3	2.76E-07	2.09E-09	4.44
PTPN6	2.78E-07	2.11E-09	-4.14
RP11-445I	2.78E-07	2.12E-09	6.12
KIR3DS1 |	2.78E-07	2.12E-09	-4.4
ERRFI1	2.79E-07	2.14E-09	5.3
TUBA3D |	2.79E-07	2.14E-09	-3.87
RERG	2.79E-07	2.14E-09	-6.39
RP11-90D	2.80E-07	2.16E-09	-5.44
POPDC3	2.81E-07	2.18E-09	7.13
RNF157	2.82E-07	2.19E-09	5.82
FSD1	2.85E-07	2.22E-09	4.76
TRIB2	2.86E-07	2.25E-09	4.93
CCL26	2.86E-07	2.25E-09	5.96
PION	2.86E-07	2.25E-09	5.44
H2AFJ	2.86E-07	2.26E-09	-6.58
TPM2	2.86E-07	2.26E-09	-3.96
TSPAN5	2.86E-07	2.27E-09	7.53
LOC10012	2.86E-07	2.27E-09	6.35
ADAMTS	2.86E-07	2.28E-09	4.49
LPL	2.88E-07	2.29E-09	-4.02
GFRA1	2.90E-07	2.32E-09	5.23
CXCR7	2.90E-07	2.32E-09	-4.39
FAM213A	2.90E-07	2.33E-09	-7.86
RP11-384	2.90E-07	2.33E-09	4.77
S100A2	2.90E-07	2.34E-09	6.03
DNAJA4	2.90E-07	2.35E-09	-3.83
AC159540	2.93E-07	2.37E-09	3.74
LOC40004	2.93E-07	2.39E-09	6
TMEM47	2.93E-07	2.39E-09	4.95
GBP2	3.00E-07	2.45E-09	5.3
LOXL1	3.01E-07	2.47E-09	5.24
KRT15	3.03E-07	2.50E-09	8.04

CASP10	3.03E-07	2.50E-09	-3.75
DMRTA1	3.03E-07	2.50E-09	4.64
EN1	3.09E-07	2.55E-09	5.5
NID1	3.11E-07	2.58E-09	4.09
TCF4	3.13E-07	2.61E-09	3.84
CAMTA1	3.14E-07	2.63E-09	5.66
LOC28488	3.14E-07	2.63E-09	-5.02
GRB10	3.15E-07	2.65E-09	4.64
PIPOX	3.18E-07	2.67E-09	4.57
SLC2A12	3.18E-07	2.68E-09	3.93
CD70	3.18E-07	2.69E-09	4.71
TMC5	3.18E-07	2.69E-09	-5.35
PDLIM1	3.18E-07	2.70E-09	-5.14
ADORA2I	3.24E-07	2.76E-09	4.69
MAFB	3.25E-07	2.79E-09	-5.4
CASC9|C.	3.25E-07	2.79E-09	-3.95
NEURL1I	3.25E-07	2.79E-09	-4.95
MB21D1	3.25E-07	2.79E-09	-4.4
PAX6	3.25E-07	2.80E-09	5.41
DIP2C	3.26E-07	2.82E-09	5.17
PPAPDC1	3.30E-07	2.86E-09	4.43
MYLK3	3.30E-07	2.86E-09	-3.89
CPEB1	3.30E-07	2.88E-09	5.69
LTBP3	3.31E-07	2.90E-09	3.89
LEPREL2	3.31E-07	2.90E-09	5.81
PKIA	3.32E-07	2.91E-09	5.57
ZCCHC5	3.32E-07	2.92E-09	5.47
FAM129A	3.38E-07	2.98E-09	4.49
GPC4	3.38E-07	2.99E-09	5.74
IRX1	3.38E-07	3.00E-09	9.94
TMC6	3.39E-07	3.01E-09	-3.64
GPM6A	3.40E-07	3.03E-09	9.24
ALDH2	3.40E-07	3.04E-09	-4.51
PDE1A	3.44E-07	3.08E-09	-6.54
MLPH	3.46E-07	3.11E-09	8.25
LDHA	3.48E-07	3.14E-09	-5.11
MCTP2	3.55E-07	3.21E-09	-4.85
MAGI2-A	3.55E-07	3.23E-09	3.66
CDCP1	3.55E-07	3.23E-09	-4.89
PERP	3.58E-07	3.26E-09	-4.04

GALT	3.59E-07	3.27E-09	-4.4
SIM2	3.63E-07	3.31E-09	-3.89
RP11-15H	3.64E-07	3.33E-09	4.7
RP11-298/	3.64E-07	3.34E-09	-3.8
FBLN5	3.67E-07	3.37E-09	-5.66
UNC5C	3.69E-07	3.40E-09	6
DZIP1	3.70E-07	3.42E-09	5.35
GUCY1B3	3.71E-07	3.43E-09	5.87
C6orf141	3.73E-07	3.46E-09	4.13
GPR39	3.74E-07	3.47E-09	4.87
MPP7	3.75E-07	3.50E-09	-4.45
GPC6	3.76E-07	3.51E-09	7.65
FAF1	3.77E-07	3.53E-09	-9.12
AKAP12	3.77E-07	3.54E-09	4.65
P2RX5|P2	3.77E-07	3.54E-09	-5.86
SAMD9L	3.78E-07	3.56E-09	4.53
HHEX	3.80E-07	3.58E-09	-5.59
VIT	3.82E-07	3.61E-09	-3.65
VSNL1	3.84E-07	3.63E-09	5.85
QPRT	3.85E-07	3.65E-09	-5.16
SUSD2	3.85E-07	3.66E-09	-4.05
B3GALNT	3.86E-07	3.68E-09	6.13
AHRR	3.88E-07	3.70E-09	-4.62
RP11-337N	3.90E-07	3.74E-09	-4.49
UPP1	3.90E-07	3.74E-09	3.73
RP11-115I	3.90E-07	3.74E-09	-4.62
PNMA2	3.90E-07	3.75E-09	3.62
LINC0070	3.92E-07	3.78E-09	4.64
CTD-2620	3.96E-07	3.82E-09	6.96
MGC1291	4.00E-07	3.88E-09	5.36
FAM26E	4.00E-07	3.89E-09	4.74
CHMP4C	4.00E-07	3.89E-09	-6.1
LINC0096	4.02E-07	3.92E-09	-4.7
FAM221A	4.02E-07	3.93E-09	6.08
NTN1	4.09E-07	4.01E-09	5.44
MUC3A|I	4.09E-07	4.02E-09	-5.38
ARHGEF	4.09E-07	4.05E-09	-5.21
ARHGAP	4.09E-07	4.05E-09	5.13
SEC14L4	4.13E-07	4.11E-09	-3.57
FBLN1	4.14E-07	4.12E-09	-3.58

CPT1C	4.15E-07	4.15E-09	4.85
DDX26B	4.15E-07	4.17E-09	3.52
PKP3	4.15E-07	4.17E-09	-5.27
CLIP2	4.17E-07	4.20E-09	4.16
DKK1	4.23E-07	4.27E-09	3.84
LINGO2	4.23E-07	4.27E-09	5.8
RP11-354I	4.23E-07	4.29E-09	-4.62
AASS	4.24E-07	4.32E-09	5.46
TBX1	4.24E-07	4.33E-09	-5.69
CTD-3049	4.24E-07	4.35E-09	4.18
CLDN2	4.24E-07	4.36E-09	-3.94
LAMA1	4.25E-07	4.38E-09	-4.84
SLC22A23	4.27E-07	4.41E-09	4.71
TMEM21(	4.29E-07	4.44E-09	-3.78
NETO2	4.30E-07	4.46E-09	-5.43
PPARG	4.32E-07	4.49E-09	-5.27
IFI27L2	4.32E-07	4.49E-09	3.55
S100A4	4.32E-07	4.49E-09	-5.27
FEZ1	4.32E-07	4.50E-09	5.36
DNAH14	4.35E-07	4.54E-09	-4.97
RP11-436I	4.37E-07	4.58E-09	4.01
RP11-66B:	4.37E-07	4.59E-09	-5.47
BNC2	4.37E-07	4.59E-09	4.68
CTD-3018	4.38E-07	4.61E-09	4.7
LRRC8C	4.38E-07	4.62E-09	6.27
ENG	4.42E-07	4.66E-09	-3.56
FAM13C	4.44E-07	4.70E-09	4.97
LOC1010(	4.46E-07	4.73E-09	-3.8
CLIP3	4.46E-07	4.73E-09	3.39
ADAM12	4.47E-07	4.76E-09	4.11
TP73	4.48E-07	4.77E-09	4.49
COBL	4.49E-07	4.80E-09	-4.23
SLIT2	4.50E-07	4.83E-09	6.32
PHGDH	4.50E-07	4.83E-09	3.94
TUBB2A	4.50E-07	4.84E-09	5.71
HMGA2	4.50E-07	4.87E-09	7.52
IGSF1	4.50E-07	4.87E-09	4.17
FAM69A	4.52E-07	4.89E-09	4.67
LEF1	4.53E-07	4.91E-09	4.33
RP11-706(	4.53E-07	4.92E-09	-4.68

EFEMP2	4.53E-07	4.94E-09	4.22
PTPRR	4.55E-07	4.99E-09	5.99
FOXD1	4.55E-07	4.99E-09	-3.6
PRSS16	4.55E-07	4.99E-09	-3.84
IFFO1	4.58E-07	5.04E-09	5.11
ACOT4|L	4.58E-07	5.06E-09	-3.69
LPHN2	4.58E-07	5.07E-09	6.78
RP3-523E	4.60E-07	5.09E-09	-7.54
CADPS2	4.68E-07	5.21E-09	5.14
IL20RB	4.71E-07	5.27E-09	-4.36
HTRA1	4.71E-07	5.28E-09	-3.98
HOXA11-	4.71E-07	5.28E-09	-4.14
CTGF	4.74E-07	5.33E-09	5.35
TMEM13	4.75E-07	5.35E-09	-3.99
PDE3A	4.78E-07	5.39E-09	-5.97
LHFPL3-	4.78E-07	5.41E-09	-3.57
GSTO2	4.84E-07	5.49E-09	-4.44
LINC0005	4.87E-07	5.53E-09	4.65
TRABD2	4.89E-07	5.56E-09	-5.37
CYB5A	4.89E-07	5.58E-09	4
IL11	4.96E-07	5.67E-09	5.58
LINC0034	4.96E-07	5.68E-09	3.59
CD274	4.96E-07	5.68E-09	7.57
DCLK1	4.96E-07	5.70E-09	5.67
HOXA10	4.97E-07	5.71E-09	-4.2
ARL14EP	4.98E-07	5.77E-09	4.15
CERS4	4.98E-07	5.78E-09	4.41
HOXA11-	5.08E-07	5.91E-09	-3.73
SNHG23|	5.08E-07	5.93E-09	4.38
ELOVL4	5.17E-07	6.05E-09	4.8
RAC2	5.17E-07	6.05E-09	-4.29
RP4-639F	5.18E-07	6.08E-09	3.79
B3GNT5	5.22E-07	6.13E-09	4.31
RP11-96D	5.25E-07	6.18E-09	-3.7
ZNF608	5.28E-07	6.23E-09	5.15
DCBLD2	5.28E-07	6.23E-09	5.24
LOC1009	5.28E-07	6.25E-09	-3.63
MAOB	5.30E-07	6.29E-09	-9.49
FNDC4	5.30E-07	6.31E-09	4.5
AC009229	5.31E-07	6.33E-09	-4

ZNF83	5.32E-07	6.35E-09	4.62
S1PR1	5.32E-07	6.38E-09	5.63
ZFHX2	5.32E-07	6.39E-09	4.45
HCG11	5.35E-07	6.44E-09	-3.47
ULBP2	5.35E-07	6.44E-09	4.63
FUOM	5.35E-07	6.47E-09	-4.88
ITGB4	5.35E-07	6.47E-09	3.64
FAM105A	5.40E-07	6.55E-09	-3.69
ARHGEF	5.41E-07	6.57E-09	3.74
TRIM22	5.43E-07	6.61E-09	4.52
ZNF577	5.43E-07	6.64E-09	4.96
IGFBP6	5.43E-07	6.64E-09	3.9
ZNF667-A	5.43E-07	6.64E-09	5.37
ELOVL2	5.45E-07	6.67E-09	5.71
PLAT	5.45E-07	6.67E-09	4.01
SLITRK2	5.51E-07	6.77E-09	4.95
LY6K	5.51E-07	6.78E-09	-4.14
RAB11FII	5.52E-07	6.81E-09	-3.24
SORBS1	5.52E-07	6.82E-09	4.32
CSPG4	5.52E-07	6.83E-09	-3.46
TP53INP1	5.53E-07	6.84E-09	5.71
SH3PXD2	5.53E-07	6.85E-09	-3.67
PGAP2	5.53E-07	6.87E-09	-3.82
AGMAT	5.57E-07	6.93E-09	-3.81
LINC0111	5.60E-07	6.97E-09	5.89
SOX21	5.60E-07	6.99E-09	8.04
ATOH8	5.60E-07	7.00E-09	-3.28
PCDH7	5.62E-07	7.03E-09	5.38
RP11-443A	5.63E-07	7.06E-09	3.71
TNFRSF2	5.65E-07	7.10E-09	5.26
ZNF551	5.66E-07	7.12E-09	4.72
IFITM4P	5.66E-07	7.13E-09	-3.84
ADAMTS	5.72E-07	7.22E-09	-3.44
REEP2	5.72E-07	7.24E-09	3.61
SLC2A6	5.74E-07	7.27E-09	4.61
AC006116	5.75E-07	7.31E-09	4.21
SERPINB	5.78E-07	7.37E-09	-3.95
ETNK2	5.82E-07	7.42E-09	4.24
ABCC2	5.82E-07	7.45E-09	-3.81
ATP8B2	5.82E-07	7.46E-09	5.37

IFFO2	5.84E-07	7.50E-09	3.5
SGCE	5.86E-07	7.53E-09	3.53
PROCR	5.86E-07	7.54E-09	3.1
ITGA7	5.92E-07	7.63E-09	5.34
RP11-132/	5.93E-07	7.65E-09	-4.84
MAL2	5.98E-07	7.74E-09	-5.65
ENC1	5.98E-07	7.76E-09	5.32
LINC0016	6.01E-07	7.81E-09	-8.04
ADAMTS:	6.02E-07	7.83E-09	-4.14
ZNF256	6.11E-07	7.97E-09	4.35
APOBEC:	6.13E-07	8.01E-09	-3.35
KCNK15-	6.13E-07	8.03E-09	-3.62
HYLS1	6.16E-07	8.07E-09	5.05
ARSJ	6.16E-07	8.08E-09	4.24
TRIM6	6.21E-07	8.17E-09	4.45
P2RX5	6.21E-07	8.19E-09	-4.76
HDX	6.21E-07	8.22E-09	3.59
GAP43	6.21E-07	8.22E-09	4.43
PDE3B	6.21E-07	8.22E-09	-5.53
ANK2	6.25E-07	8.29E-09	5.19
TRIM9	6.26E-07	8.30E-09	4.3
NKX1-2	6.26E-07	8.32E-09	3.21
CRMP1	6.31E-07	8.40E-09	3.85
GAMT	6.32E-07	8.43E-09	3.32
HSD17B8	6.38E-07	8.51E-09	-5.09
PER3	6.38E-07	8.53E-09	4.73
MAP3K4	6.38E-07	8.55E-09	4.07
RP11-834(	6.38E-07	8.56E-09	6.58
RP11-509J	6.39E-07	8.59E-09	5.29
PCDHGC	6.43E-07	8.65E-09	3.62
LOC1010(	6.44E-07	8.68E-09	4.01
MAP7	6.45E-07	8.70E-09	-3.27
RASIP1	6.47E-07	8.75E-09	-6.07
TFAP2A	6.50E-07	8.80E-09	-3.14
PLCE1	6.50E-07	8.81E-09	3.32
APBB2	6.57E-07	8.93E-09	3.59
VEGFC	6.57E-07	8.95E-09	4.05
KDR	6.57E-07	8.97E-09	6.02
HIP1	6.58E-07	8.99E-09	3.52
SALL1	6.59E-07	9.01E-09	7.83

SCOC-AS1	6.73E-07	9.23E-09	3.86
ZNF529	6.74E-07	9.26E-09	5.42
CXorf57	6.74E-07	9.27E-09	4.76
MB	6.74E-07	9.29E-09	-4.07
SLIT3	6.75E-07	9.32E-09	4.97
ARMCX5-	6.76E-07	9.36E-09	4.68
KCNQ5	6.77E-07	9.38E-09	6.1
CDS1	6.80E-07	9.43E-09	3.57
HTR7	6.81E-07	9.47E-09	3.36
SYNJ2	6.81E-07	9.48E-09	3.58
OSBP2	6.82E-07	9.50E-09	-4.48
ZNF365	6.91E-07	9.64E-09	6.3
HIPK2	6.91E-07	9.65E-09	3.46
HDGFRP.	6.93E-07	9.71E-09	3.5
PDE4DIP	6.93E-07	9.71E-09	6.97
MCOLN3	6.93E-07	9.74E-09	-3.66
RP11-15H	6.96E-07	9.81E-09	3.52
PRG4	6.99E-07	9.86E-09	-3.86
CLIC3	7.01E-07	9.90E-09	-4.34
TGM2	7.06E-07	9.99E-09	-4.15
PRTFDC1	7.08E-07	1.00E-08	4.62
GSPT2	7.10E-07	1.01E-08	4.93
VAT1L	7.10E-07	1.01E-08	5.84
GALNTL(	7.12E-07	1.02E-08	-4.38
RP11-11N	7.13E-07	1.02E-08	5.95
AC159540	7.15E-07	1.02E-08	3.64
RP11-65J2	7.16E-07	1.02E-08	-3.3
ZNF436	7.17E-07	1.03E-08	3.04
WASF3	7.17E-07	1.03E-08	5.97
IGF2BP2	7.17E-07	1.03E-08	4.41
ZNF667-A	7.18E-07	1.03E-08	4.05
FAM196B	7.21E-07	1.04E-08	3.61
GALNT3	7.22E-07	1.04E-08	-3.56
MYZAP	7.27E-07	1.05E-08	-3.67
HMCN1	7.28E-07	1.05E-08	5.23
MAP9	7.29E-07	1.06E-08	3.94
PARM1	7.35E-07	1.07E-08	3.61
ROR2	7.39E-07	1.07E-08	-4.24
COL3A1	7.39E-07	1.08E-08	-5.93
LINC0034	7.41E-07	1.08E-08	3.31

FAM211A	7.41E-07	1.08E-08	4.04
FZD10	7.44E-07	1.09E-08	-4.32
SLC16A5	7.48E-07	1.10E-08	-6.22
BLACAT1	7.49E-07	1.10E-08	3.49
FHOD3	7.49E-07	1.11E-08	4.55
FLNC	7.51E-07	1.11E-08	-4.43
HSPB7	7.51E-07	1.11E-08	-4.57
RASSF4	7.56E-07	1.12E-08	4.11
FAM201A	7.58E-07	1.13E-08	-3.83
DSP	7.60E-07	1.13E-08	-3.76
JAKMIP2	7.70E-07	1.15E-08	5.34
HOXB3	7.70E-07	1.15E-08	4.44
AVPI1	7.74E-07	1.16E-08	-4.08
P2RY6	7.78E-07	1.16E-08	-3.02
AATK	7.79E-07	1.17E-08	-3.85
SELENBI	7.90E-07	1.19E-08	-3.55
PAPLN	7.94E-07	1.19E-08	-2.93
FENDRR	7.94E-07	1.19E-08	-3.95
SERINC5	8.00E-07	1.21E-08	4.2
TRHDE	8.02E-07	1.21E-08	5.14
LRAT	8.02E-07	1.21E-08	5.41
ALPK1	8.02E-07	1.21E-08	3.37
THBS2	8.08E-07	1.22E-08	5.88
FRMD5	8.08E-07	1.23E-08	4.43
LHX1	8.16E-07	1.24E-08	4.68
OLFML1	8.18E-07	1.24E-08	-4.63
RP11-820I	8.19E-07	1.25E-08	-3.99
LPAR6	8.21E-07	1.25E-08	3.5
ANO4	8.26E-07	1.26E-08	-3.1
TUBA4A	8.31E-07	1.27E-08	-4.83
LIMS2	8.42E-07	1.29E-08	-3.23
IFI44	8.43E-07	1.29E-08	3.72
KCNJ18	8.44E-07	1.30E-08	-3.72
PNPLA3	8.44E-07	1.30E-08	-3.27
CEP112	8.44E-07	1.30E-08	4.23
KRT18P5	8.45E-07	1.30E-08	-3.45
SLC2A10	8.49E-07	1.31E-08	4.53
DAPK1	8.49E-07	1.31E-08	-3.29
RTN1	8.54E-07	1.32E-08	4.02
HR	8.54E-07	1.32E-08	-3.15

GLP2R	8.54E-07	1.32E-08	-5.4
LINC0063	8.55E-07	1.33E-08	4.41
ZNF667	8.60E-07	1.34E-08	5.74
ARMCX2	8.66E-07	1.35E-08	3.47
C19orf21	8.66E-07	1.35E-08	-3.17
RP11-106I	8.70E-07	1.36E-08	-3.31
WDR17	8.72E-07	1.36E-08	4.96
EPHX4	8.81E-07	1.38E-08	3.68
KIR2DL2	9.04E-07	1.42E-08	-3.58
PCYT1B	9.05E-07	1.42E-08	4.78
CPQ	9.05E-07	1.43E-08	4.08
MEF2C	9.05E-07	1.43E-08	4.61
JAKMIP3	9.07E-07	1.44E-08	6.05
UNC5D	9.10E-07	1.44E-08	4.23
TRAM1L1	9.10E-07	1.44E-08	3.57
KCNS3	9.10E-07	1.45E-08	5.59
KIAA1324	9.10E-07	1.45E-08	-3.09
KLHL35	9.15E-07	1.46E-08	-4.35
CCNA1	9.15E-07	1.46E-08	6.57
PLEK2	9.23E-07	1.48E-08	-4.52
SGCB	9.25E-07	1.48E-08	3.73
VLDLR	9.31E-07	1.49E-08	3.08
NPR3	9.35E-07	1.50E-08	-3.01
PDE4DIP	9.38E-07	1.51E-08	4.25
RGS3	9.42E-07	1.52E-08	3.82
LOC28458	9.42E-07	1.52E-08	3.14
TNFSF4	9.52E-07	1.54E-08	5.23
ANKRD1!	9.52E-07	1.54E-08	-3.05
SH3TC1	9.52E-07	1.54E-08	-3.23
TRAP1	9.52E-07	1.55E-08	-2.98
LOC1005(	9.53E-07	1.55E-08	-5.01
IL7R	9.55E-07	1.55E-08	3.69
RNASE13	9.58E-07	1.56E-08	-3.67
AHR	9.58E-07	1.56E-08	-6.9
PHLDA1	9.59E-07	1.57E-08	3.76
SLC6A15	9.60E-07	1.57E-08	-3.74
LRRC61	9.60E-07	1.57E-08	-4.92
TSPAN33	9.60E-07	1.58E-08	3.05
XKR6	9.72E-07	1.60E-08	4.32
RASSF5	9.75E-07	1.61E-08	-3.22

SLC46A3	9.81E-07	1.62E-08	4.49
C14orf159	9.85E-07	1.63E-08	3.63
ZNF347	9.90E-07	1.64E-08	4.95
OCIAD2	9.91E-07	1.64E-08	4.23
AQP11	9.93E-07	1.65E-08	3.24
FAT1	9.94E-07	1.65E-08	3.72
RAET1E	9.94E-07	1.66E-08	-3.53
ALDOC	9.99E-07	1.67E-08	-3.92
LINC0155	9.99E-07	1.67E-08	5.81
CAMK4	9.99E-07	1.67E-08	4.03
CDKN2D	9.99E-07	1.68E-08	2.95
ZNF85	1.00E-06	1.68E-08	3.16
PDE7B	1.00E-06	1.69E-08	-4.23
NMU	1.00E-06	1.69E-08	4.33
VPS53	1.01E-06	1.71E-08	3.49
RPL21P44	1.01E-06	1.71E-08	3.49
RUFY3	1.02E-06	1.73E-08	4.04
TENM4	1.02E-06	1.74E-08	4.16
PARP8	1.03E-06	1.76E-08	-3.38
F2R	1.05E-06	1.79E-08	4.85
PAQR8	1.05E-06	1.80E-08	3.75
SP110	1.05E-06	1.80E-08	3.6
INSL4	1.06E-06	1.82E-08	-3.17
TOM1L2	1.08E-06	1.85E-08	3.38
LRRK1	1.08E-06	1.87E-08	-4.12
CMTM7	1.08E-06	1.88E-08	6.56
BRSK1	1.09E-06	1.89E-08	4.3
CFH	1.09E-06	1.90E-08	2.9
MGLL	1.10E-06	1.90E-08	-5.02
HPD	1.10E-06	1.91E-08	-3.33
NCF2	1.10E-06	1.91E-08	-3.04
GKN1	1.10E-06	1.92E-08	-4.58
CLGN	1.11E-06	1.94E-08	3.37
MMP28	1.13E-06	1.98E-08	-2.93
HTR1D	1.13E-06	1.98E-08	-3.9
ZNF671	1.13E-06	1.98E-08	4.08
HEBP2	1.13E-06	1.99E-08	3.1
CHST10	1.14E-06	2.02E-08	4.26
TSPAN12	1.15E-06	2.03E-08	-3.23
AMPH	1.16E-06	2.05E-08	5.21

TMEM71	1.16E-06	2.05E-08	5.14
HOOK1	1.16E-06	2.06E-08	-4.03
TRIM36	1.16E-06	2.06E-08	2.92
PPL	1.16E-06	2.06E-08	-3.25
RAPGEF5	1.16E-06	2.07E-08	4.14
CA13	1.18E-06	2.11E-08	4.45
ZNF503	1.18E-06	2.11E-08	3.5
LCA5	1.18E-06	2.11E-08	3.53
VASN	1.18E-06	2.11E-08	-3.38
ZNF606	1.18E-06	2.12E-08	3.41
APBB1	1.18E-06	2.12E-08	3.39
SRPX2	1.18E-06	2.12E-08	4.08
PIK3CD	1.19E-06	2.14E-08	3.62
JAG1	1.19E-06	2.15E-08	5.48
ALDH3A1	1.19E-06	2.16E-08	-2.91
ADRBK2	1.19E-06	2.16E-08	4.27
ARL4C	1.19E-06	2.16E-08	5.47
KCNK5	1.19E-06	2.17E-08	-3.61
PARVB	1.19E-06	2.17E-08	-5.58
HOXA9|I	1.20E-06	2.19E-08	-3.99
TMOD2	1.20E-06	2.20E-08	3.32
AKR1C3|	1.21E-06	2.22E-08	-3.5
SESN3	1.21E-06	2.22E-08	3.04
DKK3	1.21E-06	2.24E-08	4.89
AKTIP	1.22E-06	2.24E-08	2.79
C3orf70	1.22E-06	2.25E-08	4.37
MMP14	1.23E-06	2.28E-08	4.76
MYEF2	1.24E-06	2.29E-08	4.06
GPR85	1.24E-06	2.29E-08	3.81
PELI2	1.24E-06	2.30E-08	2.8
APOBEC3	1.24E-06	2.30E-08	-4.07
CASP1	1.24E-06	2.30E-08	-2.98
G6PD	1.24E-06	2.30E-08	3
SH3BGRL	1.25E-06	2.32E-08	2.95
NAPEPLD	1.25E-06	2.33E-08	3.25
NXNL2	1.26E-06	2.36E-08	-4
ANTXR2	1.27E-06	2.37E-08	3.98
HOXD11	1.28E-06	2.40E-08	3.58
ZNF525	1.28E-06	2.40E-08	4.29
GAS1	1.30E-06	2.43E-08	-3.12

VASH2	1.30E-06	2.44E-08	-3.09
PNMAL1	1.30E-06	2.44E-08	7.22
SLC4A8	1.30E-06	2.46E-08	3.8
OSTM1	1.31E-06	2.47E-08	3.65
B4GALNT	1.31E-06	2.48E-08	-3.3
ZFP3	1.31E-06	2.48E-08	5.2
TACSTD2	1.32E-06	2.50E-08	-3.29
CACNG4	1.33E-06	2.53E-08	5.05
TNIK	1.33E-06	2.53E-08	5.47
NCCRP1	1.34E-06	2.54E-08	-3.6
NTNG1	1.34E-06	2.55E-08	5.85
NFE2L3	1.34E-06	2.55E-08	5.33
IFITM2	1.34E-06	2.57E-08	-2.97
HSD17B1	1.34E-06	2.57E-08	2.89
USB1	1.35E-06	2.59E-08	3.3
CSF2RA	1.35E-06	2.59E-08	-2.91
FAM209B	1.35E-06	2.59E-08	3.56
SLAIN1	1.35E-06	2.60E-08	4.91
LOC3386	1.35E-06	2.60E-08	-3.36
NRG2	1.35E-06	2.60E-08	5.25
ITGBL1	1.35E-06	2.61E-08	6.05
IL8	1.36E-06	2.63E-08	7.17
SLC35D2	1.37E-06	2.66E-08	3.53
CRIP1	1.37E-06	2.66E-08	-2.99
CLCN6	1.37E-06	2.67E-08	3.72
HLA-DQI	1.38E-06	2.68E-08	-3.57
RNF43	1.38E-06	2.69E-08	-2.88
PKIB	1.39E-06	2.73E-08	4.07
LRRC20	1.41E-06	2.77E-08	3.48
RUNX2|F	1.42E-06	2.78E-08	2.82
ZBTB18	1.43E-06	2.82E-08	5.2
ABCB4	1.44E-06	2.84E-08	4.64
SHOX2	1.45E-06	2.86E-08	3.53
PAPSS2	1.46E-06	2.90E-08	-3.13
CNIH2	1.47E-06	2.92E-08	2.74
LOC1010	1.47E-06	2.93E-08	-3.41
RGS4	1.48E-06	2.94E-08	6.71
OASL	1.48E-06	2.95E-08	-4.23
JUP	1.49E-06	2.98E-08	-3.86
CCIN	1.49E-06	2.98E-08	4.39

FRMD4A	1.50E-06	2.99E-08	5.02
GSC	1.51E-06	3.02E-08	3.58
ITGB8	1.51E-06	3.03E-08	3.9
ZNF415	1.51E-06	3.04E-08	4.82
POU5F1B	1.51E-06	3.04E-08	-3.13
JUN	1.52E-06	3.06E-08	4
CT45A2|(	1.52E-06	3.07E-08	7.94
DLC1	1.52E-06	3.07E-08	2.75
FLI1	1.52E-06	3.07E-08	7.3
HOXD3	1.52E-06	3.08E-08	3.26
ZNF788	1.52E-06	3.08E-08	4.76
AKR1C1|	1.53E-06	3.10E-08	-3.44
LIMS3|LI	1.53E-06	3.10E-08	3.37
KCNE4	1.53E-06	3.10E-08	5.61
FGF13	1.53E-06	3.12E-08	7.98
MECOM	1.55E-06	3.15E-08	3.34
EPPK1	1.55E-06	3.17E-08	-3.77
GLDC	1.57E-06	3.20E-08	2.59
C8orf42	1.57E-06	3.21E-08	3.41
SULT1A1	1.58E-06	3.23E-08	-2.89
ARHGEF	1.59E-06	3.26E-08	-3.26
SYTL5	1.61E-06	3.32E-08	4.83
ZNF772	1.62E-06	3.33E-08	5
DNMT3L	1.62E-06	3.33E-08	-3.71
TLR4	1.63E-06	3.36E-08	3.1
SNX10	1.64E-06	3.38E-08	2.82
TMEM25	1.64E-06	3.39E-08	5.11
FAM219A	1.64E-06	3.39E-08	3.85
MXRA7	1.66E-06	3.43E-08	3.36
SYT12	1.67E-06	3.46E-08	-2.85
LOC1002	1.68E-06	3.49E-08	-3.61
RIMS3	1.69E-06	3.52E-08	4.43
MYO1D	1.69E-06	3.53E-08	3.05
SLC27A3	1.70E-06	3.54E-08	3.42
PEA15	1.70E-06	3.55E-08	3.23
SERAC1	1.70E-06	3.55E-08	2.89
SLC44A5	1.70E-06	3.56E-08	7.65
STAC	1.70E-06	3.56E-08	3.41
MAP2	1.71E-06	3.58E-08	5.58
LOC1010(	1.71E-06	3.58E-08	4.1

ZNF425	1.71E-06	3.58E-08	2.91
ROBO2	1.73E-06	3.65E-08	4.67
SPATA6	1.73E-06	3.65E-08	4.27
ADA	1.73E-06	3.65E-08	-2.67
TNNC1	1.73E-06	3.66E-08	-4.21
RIMKLB	1.74E-06	3.68E-08	-2.62
RIN3	1.78E-06	3.77E-08	3.03
SALL2	1.79E-06	3.80E-08	4.79
MICAL1	1.79E-06	3.81E-08	2.74
NEURL	1.79E-06	3.81E-08	-3.03
MST1R	1.79E-06	3.82E-08	-3.9
CREB5	1.79E-06	3.83E-08	5.06
C9orf64	1.79E-06	3.84E-08	-7.62
EXOC1	1.79E-06	3.84E-08	3.31
CLOCK	1.80E-06	3.86E-08	4.36
FUT8	1.81E-06	3.89E-08	3.49
HOXD10	1.81E-06	3.89E-08	3.7
KAT2B	1.81E-06	3.89E-08	3.08
PGM2L1	1.81E-06	3.91E-08	3.39
ZNF880	1.82E-06	3.93E-08	4.22
PTPRN2	1.82E-06	3.93E-08	3.85
LPHN1	1.83E-06	3.95E-08	3.04
GALNT1(	1.83E-06	3.97E-08	2.76
FAM83A	1.83E-06	3.98E-08	-6.5
TMEFF1|	1.83E-06	3.98E-08	2.6
PRKD1	1.84E-06	4.00E-08	3.46
ADSSL1	1.84E-06	4.00E-08	-2.6
GAS6	1.85E-06	4.02E-08	3.78
RIBC2	1.86E-06	4.06E-08	-2.85
LOXL4	1.87E-06	4.08E-08	4.01
MUC1	1.87E-06	4.10E-08	-2.8
ZNF404	1.87E-06	4.10E-08	3.37
NF2	1.87E-06	4.10E-08	3.76
C10orf90	1.88E-06	4.12E-08	2.75
FEZF1-AS	1.88E-06	4.13E-08	5.04
DUSP9	1.88E-06	4.13E-08	-2.61
MFSD2A	1.89E-06	4.14E-08	3.8
PANX1	1.89E-06	4.15E-08	2.98
TUBB3	1.89E-06	4.16E-08	4.18
ZNF559-Z	1.89E-06	4.16E-08	3.32

PCSK6	1.90E-06	4.19E-08	-3.18
PDCD4	1.90E-06	4.20E-08	-2.67
SNX18	1.90E-06	4.21E-08	2.66
RPA4	1.91E-06	4.22E-08	3.46
SIRPA	1.91E-06	4.23E-08	3.36
PRICKLE	1.91E-06	4.24E-08	6.7
TBC1D8B	1.92E-06	4.26E-08	3.53
PRICKLE	1.92E-06	4.28E-08	3.85
NXPE3	1.93E-06	4.30E-08	3.35
GBP1	1.93E-06	4.30E-08	2.69
TCN2	1.96E-06	4.38E-08	2.97
ISL1	1.97E-06	4.40E-08	-4.63
HS3ST1	2.00E-06	4.47E-08	3.16
PCDHB16	2.00E-06	4.48E-08	3.81
PLCB4	2.00E-06	4.49E-08	-3.28
ANKRD2	2.00E-06	4.49E-08	-2.97
AIM1	2.00E-06	4.50E-08	4.09
GRIP1	2.01E-06	4.51E-08	3.22
GREB1L	2.01E-06	4.52E-08	-3.58
NINL	2.01E-06	4.53E-08	-3.44
S100B	2.01E-06	4.53E-08	3.32
KRT18|K	2.01E-06	4.53E-08	-2.95
IGSF3	2.02E-06	4.57E-08	2.74
HIST2H2I	2.02E-06	4.58E-08	-2.5
PRKCQ	2.02E-06	4.59E-08	3.04
CPZ	2.03E-06	4.62E-08	-2.95
SRGN	2.04E-06	4.64E-08	-3.44
HIST1H2	2.05E-06	4.67E-08	-2.74
TNK1	2.11E-06	4.82E-08	-3.21
CORO2B	2.12E-06	4.84E-08	4.46
PRUNE2	2.12E-06	4.85E-08	4.24
PDLIM4	2.12E-06	4.85E-08	4.8
CLIP4	2.12E-06	4.87E-08	3.55
NRG1	2.12E-06	4.87E-08	3.68
CNEP1R1	2.12E-06	4.89E-08	2.52
TMEM121	2.13E-06	4.92E-08	4.38
TTLL7	2.14E-06	4.95E-08	2.8
UGT1A1|	2.16E-06	4.98E-08	-3.95
LOC1005(	2.16E-06	5.01E-08	-2.98
NPFFR2	2.16E-06	5.02E-08	-5.11

IL32	2.16E-06	5.03E-08	3.15
CLMP	2.16E-06	5.03E-08	-2.63
CCDC74B	2.18E-06	5.08E-08	3.38
ONECUT	2.19E-06	5.10E-08	-2.6
TMEM20(	2.19E-06	5.11E-08	5.35
EPHB3	2.21E-06	5.16E-08	2.91
COL6A3	2.21E-06	5.16E-08	4.2
FGFR1	2.21E-06	5.18E-08	4.21
CRABP2	2.23E-06	5.22E-08	3.65
CCDC80	2.23E-06	5.24E-08	3.67
HOXA1	2.23E-06	5.24E-08	3.61
PSD3	2.23E-06	5.25E-08	2.8
NAAA	2.23E-06	5.26E-08	3.7
FBXO2	2.23E-06	5.26E-08	-2.91
RAB3B	2.24E-06	5.28E-08	3.57
AMIGO2	2.25E-06	5.32E-08	3.39
MYO7B	2.25E-06	5.33E-08	-3.3
ADCY10P	2.25E-06	5.34E-08	3.78
RRP7A	2.27E-06	5.40E-08	-2.68
TMSB15A	2.28E-06	5.41E-08	4.96
LRIG1	2.28E-06	5.43E-08	2.76
RAB15	2.29E-06	5.46E-08	3.4
BMF	2.29E-06	5.48E-08	-3.17
FOXQ1	2.30E-06	5.49E-08	-2.8
VANGL1	2.31E-06	5.52E-08	2.92
SEPT8	2.31E-06	5.54E-08	3.3
TTC39B	2.31E-06	5.56E-08	3.35
FBXL7	2.32E-06	5.57E-08	2.95
C7orf13	2.34E-06	5.63E-08	3.03
RHOD	2.35E-06	5.67E-08	-3.17
MB21D2	2.36E-06	5.72E-08	4.17
CGNL1	2.39E-06	5.80E-08	4.41
LNX1	2.40E-06	5.82E-08	3.55
TGFB2	2.42E-06	5.86E-08	2.81
SIK1	2.42E-06	5.87E-08	-3.48
ZNF208	2.42E-06	5.88E-08	2.6
FAM184A	2.42E-06	5.88E-08	4.72
CCDC88C	2.42E-06	5.91E-08	-3.08
FAM72A|	2.43E-06	5.92E-08	3.56
EGF	2.43E-06	5.95E-08	2.44

SSTR1	2.43E-06	5.95E-08	-5.41
IL11RA	2.44E-06	5.98E-08	-2.85
DNER	2.44E-06	5.98E-08	4.34
EBF1	2.45E-06	6.00E-08	2.99
TRABD2I	2.48E-06	6.09E-08	-4.45
TRPM2	2.49E-06	6.10E-08	-4.31
GBP3	2.49E-06	6.11E-08	2.81
PBX3	2.51E-06	6.18E-08	3.33
ARMCX1	2.51E-06	6.19E-08	5.55
TRO	2.51E-06	6.20E-08	4.17
ZNF461	2.52E-06	6.22E-08	4.58
RLN1	2.52E-06	6.22E-08	2.56
PPYR1|L(	2.52E-06	6.23E-08	-3.15
RASGRP3	2.52E-06	6.23E-08	5.12
CALCRL	2.53E-06	6.26E-08	3.06
DGKG	2.53E-06	6.27E-08	4.48
PCDHGB	2.53E-06	6.28E-08	3.44
GSTM2	2.53E-06	6.31E-08	3.1
GPM6B	2.54E-06	6.34E-08	3.1
PRKCB	2.54E-06	6.34E-08	-3.04
CD55	2.55E-06	6.38E-08	-2.81
HRCT1	2.55E-06	6.38E-08	3.14
C6orf223	2.59E-06	6.49E-08	-2.76
FAM171A	2.61E-06	6.54E-08	4.09
CTNND1	2.61E-06	6.55E-08	2.65
OSBPL5	2.62E-06	6.57E-08	-2.45
TCEAL3|	2.62E-06	6.59E-08	3.19
RHBDF1	2.62E-06	6.60E-08	2.79
FAM131A	2.63E-06	6.62E-08	2.92
KHDRBS:	2.63E-06	6.64E-08	9.64
PDP1	2.64E-06	6.67E-08	3.81
CNTN1	2.64E-06	6.68E-08	3.26
BCHE	2.64E-06	6.68E-08	9.29
RARG	2.64E-06	6.70E-08	-2.75
HLA-F	2.65E-06	6.72E-08	4.02
KHDC1	2.65E-06	6.72E-08	3.34
ME3	2.65E-06	6.73E-08	-3.18
HCG4	2.65E-06	6.76E-08	3.61
STK32A	2.65E-06	6.76E-08	2.99
TVP23A	2.65E-06	6.77E-08	-3.37

ZNF701	2.66E-06	6.78E-08	5.8
C3orf18	2.66E-06	6.78E-08	2.7
FMN2	2.67E-06	6.82E-08	3.8
PRLR	2.69E-06	6.89E-08	-2.52
SLC4A4	2.71E-06	6.94E-08	4.31
C4orf32	2.73E-06	7.01E-08	-5.18
PRDM2	2.76E-06	7.08E-08	3.31
JAM2	2.77E-06	7.13E-08	4
PNKD	2.77E-06	7.14E-08	-2.8
HIVEP3|1	2.78E-06	7.17E-08	4.38
SPOCD1	2.78E-06	7.18E-08	3.82
FAM83H	2.80E-06	7.22E-08	-2.52
DDTL	2.80E-06	7.24E-08	-2.65
RASA1	2.81E-06	7.27E-08	2.89
AGXT2L1	2.81E-06	7.28E-08	-2.35
GLYATL2	2.81E-06	7.28E-08	4.44
PPM1H	2.82E-06	7.30E-08	-2.43
SYT17	2.82E-06	7.32E-08	-3.11
SYT1	2.83E-06	7.34E-08	4.52
GATM	2.85E-06	7.41E-08	5.51
ERCC6L	2.86E-06	7.46E-08	2.96
TLR1	2.86E-06	7.47E-08	3.24
FAM195B	2.88E-06	7.51E-08	3.71
WNT5A	2.89E-06	7.54E-08	-3.62
THBS1	2.90E-06	7.57E-08	3.16
SDK1	2.90E-06	7.58E-08	4.2
CCL2	2.91E-06	7.62E-08	4.71
LRP3	2.91E-06	7.65E-08	-2.4
FAM117A	2.91E-06	7.65E-08	2.77
MAGEA1	2.93E-06	7.70E-08	-2.56
AKR1C3	2.93E-06	7.70E-08	-2.61
WSCD1	2.93E-06	7.71E-08	4.83
DPY19L2	2.98E-06	7.86E-08	4.96
SRGAP2C	2.98E-06	7.88E-08	2.4
ZNF649	2.99E-06	7.92E-08	3.66
PPP1R14C	3.00E-06	7.95E-08	2.75
ZNF883	3.00E-06	7.96E-08	3.8
ADARB2	3.00E-06	7.98E-08	-2.5
TRIM4	3.00E-06	7.99E-08	3.09
GCNT1	3.00E-06	7.99E-08	3.6

ANXA3	3.00E-06	7.99E-08	-5.27
CUEDC1	3.02E-06	8.05E-08	2.88
RGL1	3.03E-06	8.09E-08	3.28
GKN2	3.04E-06	8.11E-08	-2.66
IRX5	3.05E-06	8.16E-08	-3.41
PARP14	3.07E-06	8.22E-08	3.3
C3orf67	3.08E-06	8.25E-08	2.43
SEMA4F	3.08E-06	8.26E-08	2.57
ACBD7	3.08E-06	8.27E-08	3.98
ZNF423	3.08E-06	8.29E-08	4.03
KIAA1377	3.09E-06	8.32E-08	-2.77
GPRIN2	3.10E-06	8.36E-08	-3.57
LOC1510(	3.12E-06	8.42E-08	-2.89
NR4A3	3.12E-06	8.43E-08	-2.77
KIAA1244	3.12E-06	8.44E-08	3.23
USO1	3.13E-06	8.47E-08	2.54
ITGA3	3.14E-06	8.50E-08	3.27
PAX7	3.14E-06	8.51E-08	4.48
CHFR	3.15E-06	8.53E-08	3.05
ZNF568	3.15E-06	8.54E-08	5.63
NMT2	3.17E-06	8.59E-08	4.19
RGS17	3.19E-06	8.67E-08	4.55
MPP1	3.22E-06	8.79E-08	2.98
SH2B3	3.24E-06	8.83E-08	3.98
SCD5	3.25E-06	8.86E-08	3.02
LOC6535(	3.25E-06	8.86E-08	2.62
SSPN	3.25E-06	8.88E-08	3.63
CRYBB2P	3.25E-06	8.90E-08	2.43
GULP1	3.28E-06	8.99E-08	2.49
NACAD	3.28E-06	9.00E-08	2.64
GBP5	3.29E-06	9.03E-08	3.41
SUSD5	3.29E-06	9.04E-08	-3.35
IRF7	3.29E-06	9.05E-08	-2.74
TAB2	3.30E-06	9.08E-08	2.44
MTMR11	3.34E-06	9.24E-08	2.85
LY96	3.35E-06	9.25E-08	8.9
LARP6	3.35E-06	9.26E-08	3.83
IFRD1	3.35E-06	9.28E-08	3.25
CAMK2D	3.36E-06	9.30E-08	2.48
RELL2	3.36E-06	9.32E-08	2.71

MAP2K6	3.39E-06	9.41E-08	-2.56
HS3ST3A1	3.39E-06	9.44E-08	3.76
LDOC1	3.40E-06	9.45E-08	2.33
CTSZ	3.42E-06	9.52E-08	-3.06
TMEM44	3.42E-06	9.54E-08	2.61
MAB21L1	3.43E-06	9.57E-08	4.1
ATP11A	3.43E-06	9.60E-08	-2.33
NFIA	3.44E-06	9.64E-08	-2.67
CTSB	3.49E-06	9.79E-08	3.64
RECK	3.49E-06	9.79E-08	3.03
TSHZ3	3.50E-06	9.82E-08	3.85
IGFBP5	3.51E-06	9.87E-08	4.49
TNNT1	3.51E-06	9.89E-08	2.36
LAMP2	3.51E-06	9.90E-08	3.1
FHL1	3.52E-06	9.93E-08	2.47
KIAA0226	3.54E-06	9.98E-08	2.69
PLEKHG	3.54E-06	9.98E-08	2.44
HIST1H3I	3.54E-06	1.00E-07	-2.56
PCDHB10	3.55E-06	1.00E-07	3.43
KRTCAP3	3.57E-06	1.01E-07	2.77
LOC10013	3.60E-06	1.02E-07	-2.94
HIST1H2I	3.61E-06	1.02E-07	-2.63
OSBPL1A	3.64E-06	1.04E-07	-2.58
ABI3BP	3.65E-06	1.04E-07	3.3
C10orf10	3.67E-06	1.05E-07	3.34
TECPR1	3.67E-06	1.05E-07	2.34
SP8	3.70E-06	1.06E-07	3.35
CTSH	3.70E-06	1.06E-07	2.66
CAPS2	3.73E-06	1.07E-07	2.78
STX11	3.74E-06	1.07E-07	2.84
TTC39C	3.75E-06	1.08E-07	-3.3
HMGCS1	3.75E-06	1.08E-07	2.52
PRSS8	3.75E-06	1.08E-07	-2.55
KIF26B	3.76E-06	1.08E-07	2.3
MAGI2-A	3.76E-06	1.08E-07	2.77
TRPC1	3.81E-06	1.10E-07	3.87
MAP6D1	3.81E-06	1.10E-07	2.76
STOM	3.86E-06	1.11E-07	-2.38
NOVA1	3.87E-06	1.12E-07	3.17
PPAPDC2	3.88E-06	1.12E-07	2.59

EFR3B	3.88E-06	1.12E-07	3.08
LAMC3	3.89E-06	1.13E-07	-2.17
KIAA145(	3.89E-06	1.13E-07	-2.28
C6orf120	3.89E-06	1.13E-07	2.42
CABLES1	3.91E-06	1.14E-07	-2.35
FBXL16	3.92E-06	1.14E-07	-4.61
AGPAT4	3.92E-06	1.14E-07	2.89
NAPRT1	3.92E-06	1.14E-07	-4.24
PCDHGA	3.92E-06	1.14E-07	2.47
NEDD9	3.92E-06	1.14E-07	2.5
SOX17	3.96E-06	1.15E-07	-3.06
HAS3	4.00E-06	1.17E-07	3.63
XKR4	4.00E-06	1.17E-07	3.94
CFD	4.02E-06	1.18E-07	-3.52
DTNA	4.05E-06	1.19E-07	2.73
HOXD8	4.06E-06	1.19E-07	2.76
RASSF2	4.06E-06	1.19E-07	2.92
RNASET2	4.06E-06	1.19E-07	2.56
FCHSD1	4.07E-06	1.20E-07	2.66
HIST1H1(	4.08E-06	1.20E-07	-3.2
EPHA4	4.08E-06	1.21E-07	6.4
LOC6535(	4.08E-06	1.21E-07	2.46
TP53TG1	4.08E-06	1.21E-07	2.81
CSAG1|C	4.09E-06	1.21E-07	3.23
VGLL3	4.14E-06	1.23E-07	-3.79
IQGAP3	4.14E-06	1.23E-07	3.42
PTER	4.14E-06	1.23E-07	-4.85
ARHGAP.	4.16E-06	1.23E-07	-4.11
C10orf116	4.17E-06	1.24E-07	-2.53
EPB41L5	4.17E-06	1.24E-07	3.28
ZNF681	4.18E-06	1.25E-07	6.29
ZNF542	4.19E-06	1.25E-07	4.35
TRAPPC1	4.19E-06	1.25E-07	2.55
ZNF14	4.20E-06	1.25E-07	2.57
THEM4	4.21E-06	1.26E-07	-2.36
PARP9	4.21E-06	1.26E-07	2.24
GPR63	4.21E-06	1.26E-07	3.54
MFI2	4.21E-06	1.26E-07	-2.21
COPG2IT	4.22E-06	1.26E-07	2.38
KCNQ5-I'	4.23E-06	1.27E-07	3.67

TSPAN8	4.23E-06	1.27E-07	-5.57
TUBB2B	4.24E-06	1.27E-07	3.06
ARHGEF	4.24E-06	1.27E-07	3.97
DHRS12	4.25E-06	1.28E-07	2.48
ADAMTS	4.25E-06	1.28E-07	3.58
FGFBP1	4.26E-06	1.28E-07	-3.61
ZNF320	4.28E-06	1.29E-07	5.7
ATP6V0E	4.31E-06	1.30E-07	2.56
FAM101B	4.33E-06	1.31E-07	4.42
CCBL2	4.34E-06	1.31E-07	2.46
TLE2	4.35E-06	1.32E-07	-2.45
ADAMTS	4.37E-06	1.33E-07	5.84
ZNF583	4.37E-06	1.33E-07	4.13
OLFM1	4.37E-06	1.33E-07	-3.03
LTBP1	4.37E-06	1.33E-07	2.6
SCOC	4.37E-06	1.33E-07	3.08
FAM92A1	4.38E-06	1.34E-07	2.57
IQGAP2	4.38E-06	1.34E-07	3.92
ZNF470	4.38E-06	1.34E-07	2.29
C19orf25	4.40E-06	1.34E-07	2.87
GPRASP1	4.40E-06	1.35E-07	2.76
SPATS2L	4.40E-06	1.35E-07	4
PGF	4.41E-06	1.35E-07	3.16
CHGB	4.41E-06	1.35E-07	3.69
GALNT5	4.42E-06	1.36E-07	2.26
SLC25A21	4.45E-06	1.37E-07	-3.66
LY6E	4.47E-06	1.38E-07	-2.53
CHD9	4.47E-06	1.38E-07	3.01
PAG1	4.47E-06	1.38E-07	2.68
SYTL2	4.47E-06	1.38E-07	2.73
DPYSL4	4.47E-06	1.38E-07	3.02
SNX7	4.47E-06	1.38E-07	2.6
SYT14	4.47E-06	1.38E-07	2.64
GNG2	4.48E-06	1.38E-07	4.46
LOC10012	4.48E-06	1.38E-07	2.62
SEPP1	4.48E-06	1.38E-07	-2.41
NMB	4.48E-06	1.39E-07	2.96
SCARF2	4.48E-06	1.39E-07	2.31
ZNF655	4.48E-06	1.39E-07	2.95
CHRDL1	4.50E-06	1.40E-07	2.97

PRSS12	4.51E-06	1.40E-07	2.39
FAR2	4.52E-06	1.40E-07	2.69
ARSG	4.53E-06	1.41E-07	-2.56
TICAM2|'	4.53E-06	1.41E-07	3.11
MUM1L1	4.53E-06	1.41E-07	2.28
DOCK4	4.54E-06	1.42E-07	2.69
ZNF775	4.55E-06	1.42E-07	2.84
DIO2	4.55E-06	1.42E-07	-3.01
LRRC66	4.55E-06	1.42E-07	2.47
ASMTL	4.55E-06	1.42E-07	-2.52
GUF1	4.56E-06	1.43E-07	2.6
ROBO3	4.57E-06	1.43E-07	4.64
SNN	4.62E-06	1.45E-07	2.44
VAMP8	4.66E-06	1.46E-07	-4.1
RHOG	4.67E-06	1.47E-07	-2.29
LACC1	4.71E-06	1.49E-07	-2.73
COL21A1	4.72E-06	1.49E-07	5.03
CNKSR2	4.74E-06	1.50E-07	4.96
IMPA2	4.74E-06	1.50E-07	-3
FAM155A	4.75E-06	1.50E-07	4.06
FREM2	4.76E-06	1.51E-07	3.46
IRX3	4.80E-06	1.52E-07	-2.33
SFI1	4.80E-06	1.53E-07	-2.32
PIP5K1B	4.80E-06	1.53E-07	-3.27
CDH10	4.81E-06	1.53E-07	3.96
ANXA1	4.81E-06	1.53E-07	2.65
HOPX	4.81E-06	1.53E-07	2.2
HIST1H2I	4.81E-06	1.53E-07	-2.53
MGP	4.82E-06	1.54E-07	3.67
SDR16C5	4.82E-06	1.54E-07	-2.3
BEX1	4.82E-06	1.54E-07	3.87
IGFBP4	4.82E-06	1.54E-07	2.81
ZNF879	4.82E-06	1.54E-07	3.33
KTN1-AS'	4.82E-06	1.55E-07	-2.47
LPCAT2	4.85E-06	1.56E-07	3.08
UG0898H	4.90E-06	1.57E-07	3.04
LOC1001:	4.90E-06	1.58E-07	-3.29
TRADD	4.94E-06	1.59E-07	2.51
PDGFRB	4.96E-06	1.60E-07	-4.15
STRA6	4.96E-06	1.60E-07	2.68

UTRN	4.97E-06	1.61E-07	2.91
PDGFRL	4.97E-06	1.61E-07	3.13
C5	4.97E-06	1.61E-07	2.72
LHFP	4.99E-06	1.62E-07	3.66
TRIM2	5.05E-06	1.64E-07	3.44
FAM84A	5.05E-06	1.64E-07	3.54
NDRG1	5.05E-06	1.64E-07	-3.08
VPS41	5.07E-06	1.65E-07	2.8
SORL1	5.07E-06	1.65E-07	3.58
LOC72839	5.07E-06	1.66E-07	2.56
CAPN5	5.07E-06	1.66E-07	2.52
DPYD	5.09E-06	1.66E-07	2.47
SKIDA1	5.10E-06	1.67E-07	2.69
NCEH1	5.10E-06	1.67E-07	4.09
PCDHGB	5.10E-06	1.67E-07	2.47
EFNA1	5.11E-06	1.68E-07	-2.35
STC1	5.11E-06	1.68E-07	3.52
MPP4	5.12E-06	1.68E-07	3.24
MFSD6	5.12E-06	1.68E-07	3.03
GGT7	5.14E-06	1.69E-07	3.55
PTK7	5.14E-06	1.69E-07	2.67
PAK3	5.16E-06	1.70E-07	4.11
NAV3	5.18E-06	1.71E-07	4.02
ZFP28	5.18E-06	1.71E-07	4.93
FCHO1	5.18E-06	1.71E-07	-2.31
CACNA2I	5.20E-06	1.72E-07	-2.33
ARHGEF	5.20E-06	1.72E-07	2.16
RIMS2	5.22E-06	1.73E-07	2.61
ABCA3	5.26E-06	1.74E-07	3.35
BAG1	5.27E-06	1.74E-07	-2.39
ZNF71	5.27E-06	1.75E-07	3.87
FBN2	5.32E-06	1.77E-07	-3.34
CFHR1|C	5.33E-06	1.77E-07	2.52
PRRG4	5.33E-06	1.78E-07	-2.12
ARHGAP	5.34E-06	1.78E-07	2.67
ACVR1	5.35E-06	1.79E-07	2.6
CUL4B	5.35E-06	1.79E-07	2.76
ZNF682	5.35E-06	1.79E-07	3.19
DNM3	5.42E-06	1.81E-07	2.98
GJB2	5.44E-06	1.82E-07	3.05

STXBP2	5.44E-06	1.83E-07	-2.16
SLC45A1	5.48E-06	1.84E-07	3.26
LGSN	5.49E-06	1.85E-07	-2.97
TMEM8B	5.51E-06	1.85E-07	2.81
ITGB1BP	5.52E-06	1.86E-07	3.22
RNF165	5.52E-06	1.86E-07	-2.54
NUMBL	5.52E-06	1.87E-07	2.97
PLGRKT	5.52E-06	1.87E-07	2.14
SLC29A2	5.55E-06	1.88E-07	-2.58
ZBED1	5.55E-06	1.88E-07	-2.57
TMEM24(	5.59E-06	1.90E-07	-2.4
MAGED1	5.61E-06	1.90E-07	2.76
TNS3	5.62E-06	1.91E-07	2.45
GDI1	5.62E-06	1.91E-07	2.94
MYOM2	5.66E-06	1.93E-07	3.27
RGMB	5.69E-06	1.94E-07	3.12
HOTAIR	5.69E-06	1.94E-07	-2.3
MPC1	5.70E-06	1.94E-07	2.54
GUCA1B	5.71E-06	1.95E-07	3.06
NT5DC3	5.71E-06	1.95E-07	2.14
SARM1	5.72E-06	1.95E-07	2.77
CROT	5.72E-06	1.96E-07	2.49
NHLRC3	5.74E-06	1.96E-07	2.34
C4orf19	5.74E-06	1.97E-07	2.44
C8orf4	5.74E-06	1.97E-07	-2.31
SRC	5.75E-06	1.97E-07	2.51
NELL2	5.76E-06	1.98E-07	2.03
DLX4	5.76E-06	1.98E-07	-2.36
SLC39A13	5.78E-06	1.99E-07	2.55
ABR	5.82E-06	2.00E-07	2.34
CD24	5.85E-06	2.02E-07	-2.76
INPP5F	5.85E-06	2.02E-07	2.88
PEG10	5.86E-06	2.02E-07	2.45
TRPS1	5.90E-06	2.03E-07	2.24
SORBS2	5.93E-06	2.05E-07	2.47
TBX2	5.93E-06	2.05E-07	2.41
RGS12	5.93E-06	2.05E-07	2.09
KCNK1	5.94E-06	2.06E-07	-2.42
TFAP2C	5.94E-06	2.06E-07	4.05
GDF15	5.95E-06	2.06E-07	2.13

MAGEH1	5.98E-06	2.08E-07	3.53
CMTM8	5.99E-06	2.08E-07	2.83
HIST1H2l	6.00E-06	2.09E-07	-2.12
POLD4	6.01E-06	2.09E-07	2.08
CYP4V2	6.02E-06	2.10E-07	4.35
SYNGR3	6.06E-06	2.11E-07	3.52
APLP1	6.08E-06	2.12E-07	2.79
HOXB5	6.08E-06	2.12E-07	-3.39
TPSAB1|'	6.08E-06	2.12E-07	-2.14
PLXNB3	6.08E-06	2.12E-07	2.09
GRB7	6.09E-06	2.13E-07	-2.83
METTL7I	6.11E-06	2.14E-07	3.64
AK3	6.13E-06	2.14E-07	2.32
TENM3	6.15E-06	2.15E-07	3.14
SHB	6.15E-06	2.16E-07	2.63
RPRM	6.15E-06	2.16E-07	3.16
EGFEM1I	6.19E-06	2.18E-07	-2.65
TMEM37	6.22E-06	2.19E-07	-2.16
RAB36	6.23E-06	2.19E-07	2.74
C10orf11	6.25E-06	2.20E-07	2.77
CRISPLD'	6.25E-06	2.20E-07	4.18
RHOC	6.26E-06	2.21E-07	2.37
RCHY1	6.26E-06	2.21E-07	3.04
ITFG1	6.31E-06	2.23E-07	2.2
ZNF91	6.32E-06	2.24E-07	3.72
PLD5	6.32E-06	2.24E-07	2.73
GPR160	6.32E-06	2.24E-07	3.25
TEAD4	6.33E-06	2.24E-07	-2.27
RNASEK	6.33E-06	2.25E-07	2.78
SKAP2	6.34E-06	2.25E-07	2.45
FBXO25	6.34E-06	2.26E-07	2.73
MCOLN2	6.36E-06	2.26E-07	3.08
HIST1H2l	6.39E-06	2.27E-07	-3.46
TFR2	6.39E-06	2.27E-07	-2.77
ANKS1B	6.41E-06	2.28E-07	2.37
TET1	6.42E-06	2.29E-07	4.26
OR6N2	6.46E-06	2.31E-07	-3.03
C11orf63	6.47E-06	2.31E-07	3.4
DNAJB2	6.47E-06	2.31E-07	2.15
EFHA2	6.48E-06	2.32E-07	2.98

PSTK	6.48E-06	2.32E-07	2.42
LRRC49	6.48E-06	2.32E-07	2.67
ZMAT1	6.49E-06	2.32E-07	3.18
ESRP2	6.49E-06	2.33E-07	-4.46
FAM122B	6.50E-06	2.33E-07	2.38
ICA1L	6.50E-06	2.33E-07	2.44
NPAS2	6.50E-06	2.33E-07	-2.81
RNF38	6.55E-06	2.36E-07	2.65
GXYLT2	6.56E-06	2.36E-07	3.5
CTSO	6.57E-06	2.37E-07	3.46
BCAS4	6.59E-06	2.37E-07	2.44
CA2	6.59E-06	2.38E-07	2.24
CYB561D	6.59E-06	2.38E-07	2.51
EVPL	6.60E-06	2.38E-07	-2.58
DNPH1	6.60E-06	2.38E-07	-2.44
APC2	6.65E-06	2.40E-07	4.24
PDGFA	6.67E-06	2.41E-07	3.32
ARHGAP	6.69E-06	2.42E-07	3.8
IFT74	6.69E-06	2.42E-07	2.78
HEATR4	6.70E-06	2.43E-07	-4.05
ANGPT1	6.71E-06	2.43E-07	-3.02
ELAVL2	6.71E-06	2.44E-07	2.87
PAQR3	6.73E-06	2.44E-07	2.62
FAM50B	6.73E-06	2.44E-07	-2.56
LYPD6	6.73E-06	2.45E-07	2.22
FOXD4L1	6.73E-06	2.45E-07	3.16
NMNAT2	6.75E-06	2.46E-07	2.65
FBXL2	6.78E-06	2.47E-07	2.36
ITGA1	6.78E-06	2.47E-07	-2.29
ARNTL2	6.79E-06	2.48E-07	2.09
PIGZ	6.80E-06	2.48E-07	2.21
ATP11B	6.81E-06	2.49E-07	2.26
TSPAN15	6.82E-06	2.49E-07	2.48
SEC22B	6.83E-06	2.50E-07	3.02
GDF6	6.84E-06	2.50E-07	4.23
DOCK3	6.85E-06	2.50E-07	2.89
CPED1	6.89E-06	2.52E-07	-2.43
UBE2S	6.89E-06	2.52E-07	2.65
ATF7IP2	6.90E-06	2.53E-07	-2.61
BCAS2	6.94E-06	2.55E-07	2.66

EIF5A2	6.94E-06	2.55E-07	3.08
NT5E	6.95E-06	2.56E-07	2.56
TUBA3FP	6.98E-06	2.57E-07	2.01
RTCA	6.98E-06	2.57E-07	2.19
PTPRB	6.99E-06	2.58E-07	2.36
SH3BP1	7.03E-06	2.60E-07	2.12
PRTG	7.03E-06	2.60E-07	-2.51
CYP2J2	7.03E-06	2.60E-07	2.63
NBEA	7.03E-06	2.60E-07	2.25
GAGE2D	7.04E-06	2.61E-07	-2.66
SHC2	7.09E-06	2.63E-07	4.36
ICAM4	7.10E-06	2.64E-07	3.88
TRAF1	7.16E-06	2.66E-07	2.14
CHST1	7.16E-06	2.66E-07	3.89
CHURC1	7.19E-06	2.67E-07	-2.33
ZSCAN12	7.21E-06	2.68E-07	-4.3
DNAJC22	7.21E-06	2.68E-07	2.22
NIPAL2	7.21E-06	2.68E-07	-2.08
ABCG1	7.22E-06	2.69E-07	-3.26
C7orf57	7.22E-06	2.69E-07	2.85
ARL2BP	7.22E-06	2.70E-07	2.43
CIRBP	7.23E-06	2.70E-07	2.84
LOC10013	7.23E-06	2.70E-07	3.63
SPATA13	7.27E-06	2.72E-07	3.62
MICALL2	7.27E-06	2.72E-07	2.54
RNF122	7.27E-06	2.72E-07	-2.33
AR	7.27E-06	2.72E-07	4.18
WTAP	7.38E-06	2.77E-07	2.87
TIGD7	7.39E-06	2.77E-07	3
AKR1C1|	7.40E-06	2.78E-07	-3.27
ACSS1	7.41E-06	2.78E-07	2.08
CA5B	7.41E-06	2.79E-07	-3.16
ATP11C	7.41E-06	2.79E-07	2.23
LONRF2	7.45E-06	2.80E-07	3.33
FHDC1	7.45E-06	2.81E-07	2.78
C10orf35	7.48E-06	2.82E-07	2.96
LMNB2	7.51E-06	2.83E-07	3
CCDC68	7.51E-06	2.83E-07	-3.01
RPS6KA2	7.51E-06	2.84E-07	2.55
SMAD7	7.51E-06	2.84E-07	-3.21

DRD2	7.53E-06	2.85E-07	4.45
KIF3C	7.54E-06	2.85E-07	2.25
ZNF468	7.54E-06	2.86E-07	3.4
ADCK3	7.54E-06	2.86E-07	-1.95
UBA6	7.56E-06	2.87E-07	2.5
CBR3	7.58E-06	2.88E-07	2.93
PC	7.61E-06	2.89E-07	-1.94
FGFR2	7.61E-06	2.89E-07	2.71
ESRRG	7.63E-06	2.91E-07	2.97
IFITM3	7.67E-06	2.93E-07	-2.29
FOLR1	7.67E-06	2.93E-07	-2.19
ATP5G1	7.67E-06	2.93E-07	-2.6
CYP4X1	7.69E-06	2.94E-07	-2.08
MAP4K4	7.72E-06	2.95E-07	2.63
C3AR1	7.72E-06	2.96E-07	2.87
STARD3N	7.73E-06	2.96E-07	2.79
ANKRA2	7.73E-06	2.97E-07	2.15
DCTD	7.76E-06	2.98E-07	2.34
ASAP3	7.78E-06	2.98E-07	1.97
ANKRD6	7.81E-06	3.00E-07	2.47
SRD5A1	7.82E-06	3.00E-07	-1.94
ICK	7.85E-06	3.02E-07	2.18
SCRN1	7.91E-06	3.04E-07	2.36
NUDT11	7.91E-06	3.05E-07	4.57
ZNF264	7.91E-06	3.05E-07	3.09
TMEM24	7.92E-06	3.05E-07	3.07
PARP11	7.94E-06	3.07E-07	2.1
RERE	7.94E-06	3.07E-07	2.32
PRKCD	7.96E-06	3.08E-07	2.83
PRRT3	7.96E-06	3.08E-07	2.94
SMAD3	7.97E-06	3.09E-07	2.14
SPATA6L	7.97E-06	3.09E-07	3.03
PROSER2	7.97E-06	3.09E-07	-2.46
CD109	7.97E-06	3.09E-07	2.32
GLI3	7.98E-06	3.10E-07	2.74
ZDHHC1	8.00E-06	3.11E-07	2.17
ASPHD2	8.02E-06	3.12E-07	3.38
SLC39A3	8.02E-06	3.12E-07	2.79
TNFRSF1	8.04E-06	3.13E-07	2.38
ELOVL3	8.05E-06	3.13E-07	-2.12

ZNF567	8.06E-06	3.14E-07	2.51
LAYN	8.06E-06	3.14E-07	6.63
C3orf72	8.06E-06	3.14E-07	-5.45
OSBPL7	8.06E-06	3.14E-07	2.42
NMI	8.07E-06	3.15E-07	2.26
GCA	8.11E-06	3.17E-07	3.24
ANKRD1	8.11E-06	3.17E-07	3.62
NHSL1	8.11E-06	3.17E-07	2.64
LRP12	8.12E-06	3.18E-07	2.18
TMEM15[illegible]	8.16E-06	3.19E-07	3.92
ATF1	8.24E-06	3.23E-07	-1.89
MAPRE2	8.25E-06	3.24E-07	2.2
FYN	8.27E-06	3.25E-07	2.22
ZNF670-Z	8.33E-06	3.27E-07	-2.01
ZNF808	8.35E-06	3.28E-07	2.04
EVI2B	8.35E-06	3.29E-07	3.28
GPR162	8.35E-06	3.29E-07	3.17
CUL9	8.35E-06	3.29E-07	2.19
OGDHL	8.35E-06	3.29E-07	2.38
LOC1010[illegible]	8.35E-06	3.30E-07	-2.39
CLEC11A	8.38E-06	3.31E-07	3.05
RAB4B|M	8.39E-06	3.32E-07	2.1
USP46	8.45E-06	3.35E-07	2.06
EHD4	8.45E-06	3.35E-07	2.59
CEP135	8.46E-06	3.35E-07	3.02
WNT10B	8.46E-06	3.35E-07	-2.41
TM4SF1	8.46E-06	3.36E-07	-3.06
FHL1|LO	8.49E-06	3.37E-07	2.82
FAM20C	8.50E-06	3.37E-07	2.89
OPTN	8.57E-06	3.41E-07	2.61
PDK4	8.64E-06	3.44E-07	-2.48
ITPRIPL1	8.65E-06	3.45E-07	-3.48
TMEM55[illegible]	8.65E-06	3.45E-07	2.6
RBM20	8.66E-06	3.45E-07	4.14
IRS1	8.66E-06	3.45E-07	3.04
BAIAP2L[illegible]	8.67E-06	3.46E-07	-1.92
HIST1H2[illegible]	8.68E-06	3.46E-07	-2.24
CCDC120	8.68E-06	3.47E-07	-1.99
MLLT4	8.69E-06	3.47E-07	2.57
C2CD4C	8.69E-06	3.47E-07	3.33

PHTF1	8.69E-06	3.47E-07	2.5
MCC	8.70E-06	3.48E-07	2.71
GTF2H5	8.72E-06	3.49E-07	2.61
LCTL	8.73E-06	3.50E-07	2.95
IQCJ-SCH	8.78E-06	3.52E-07	2.06
GGT1|G(	8.78E-06	3.53E-07	-2.31
MLF1IP	8.78E-06	3.53E-07	2.61
FAM209A	8.80E-06	3.54E-07	2.57
DIRC3	8.80E-06	3.54E-07	2.55
DMD	8.82E-06	3.55E-07	-2.35
TRERF1	8.82E-06	3.55E-07	3.01
CBFA2T2	8.83E-06	3.56E-07	2.09
LOC1010:	8.84E-06	3.57E-07	3.49
CAMKK2	8.85E-06	3.57E-07	-2.37
SORT1	8.86E-06	3.58E-07	2.31
NINJ2	8.88E-06	3.59E-07	-2.54
STARD5	8.90E-06	3.60E-07	2.43
TENM1	8.94E-06	3.62E-07	3.39
DOCK11	8.99E-06	3.65E-07	2.27
TMEM16:	9.00E-06	3.65E-07	2.31
RASL10B	9.01E-06	3.66E-07	-2.93
FAM149A	9.04E-06	3.67E-07	2.83
ACOT7	9.04E-06	3.68E-07	2.36
FIGN	9.04E-06	3.68E-07	2.16
CREB3	9.05E-06	3.68E-07	2.87
LOC1002{	9.06E-06	3.69E-07	2.56
TMEM80	9.06E-06	3.69E-07	2.13
ILDR2	9.08E-06	3.70E-07	1.97
LOC1005(	9.09E-06	3.71E-07	2.24
MSMO1	9.10E-06	3.72E-07	2.76
HPS1	9.10E-06	3.72E-07	2.84
ZNF285	9.11E-06	3.72E-07	3.34
STMN3	9.12E-06	3.73E-07	3.67
KLHDC9	9.13E-06	3.74E-07	-1.89
LOC3449(	9.19E-06	3.77E-07	2.05
PIK3R1	9.22E-06	3.79E-07	2.6
KRT8P41	9.26E-06	3.81E-07	-2.12
CRHR1-IT	9.27E-06	3.82E-07	2.33
FOXO3|F	9.28E-06	3.82E-07	2.68
BDKRB2	9.28E-06	3.83E-07	-4.26

HLA-H	9.28E-06	3.83E-07	2.21
SH3BP4	9.28E-06	3.83E-07	2.44
PCDH9	9.29E-06	3.84E-07	-3.28
MTAP	9.34E-06	3.86E-07	3.08
GBA2	9.36E-06	3.87E-07	2.16
USP43	9.38E-06	3.88E-07	-2.3
IMPACT	9.40E-06	3.90E-07	-2.25
SPANXD	9.42E-06	3.91E-07	3.87
RADIL	9.45E-06	3.92E-07	4.09
GPT2	9.45E-06	3.93E-07	2.91
TGFBR3	9.47E-06	3.94E-07	-2.35
KLHL9	9.47E-06	3.95E-07	2.49
PNPLA4	9.51E-06	3.97E-07	-2.3
CEBPD	9.51E-06	3.97E-07	-2.43
KIF1B	9.52E-06	3.97E-07	2.46
ZNF25	9.54E-06	3.98E-07	2.03
PRKAR2B	9.56E-06	4.00E-07	2.68
LOC1005(	9.62E-06	4.03E-07	-2.73
IER3	9.64E-06	4.04E-07	-3.18
TBX19	9.65E-06	4.04E-07	2.51
CERS6	9.65E-06	4.04E-07	2.49
NTM|LO	9.69E-06	4.06E-07	7.45
CCDC50	9.69E-06	4.06E-07	3.3
FAM174B	9.71E-06	4.08E-07	2.43
FRYL	9.82E-06	4.13E-07	2.16
PPAP2B	9.84E-06	4.14E-07	-2.37
NYAP2	9.84E-06	4.15E-07	-2.52
FLT3LG	9.84E-06	4.15E-07	3.05
PARD6A	9.87E-06	4.17E-07	2.7
POFUT2	9.87E-06	4.17E-07	2.57
ZNF521	9.89E-06	4.18E-07	-4.37
CD302|LY	9.94E-06	4.21E-07	6.53
SEC14L2	1.00E-05	4.24E-07	3.72
KBTBD8	1.00E-05	4.25E-07	3.32
CORO6	1.00E-05	4.26E-07	-2.37
NAV1	1.00E-05	4.26E-07	1.93
TGM1	1.00E-05	4.26E-07	-2.1
NBPF4|N	1.00E-05	4.26E-07	-2.48
SMYD3	1.00E-05	4.27E-07	1.84
CDKN2B-	1.00E-05	4.28E-07	-4

LOC10013	1.00E-05	4.28E-07	-2.47
FZD7	1.00E-05	4.28E-07	2.06
DOK7	1.01E-05	4.29E-07	-2.05
RPS23	1.01E-05	4.32E-07	2.7
BAI3	1.01E-05	4.32E-07	2.44
GPX8	1.01E-05	4.34E-07	2.6
MTCP1	1.02E-05	4.34E-07	1.85
ALCAM	1.02E-05	4.35E-07	3.05
SPRY4	1.03E-05	4.41E-07	3.23
PPP2R3A	1.03E-05	4.41E-07	2.53
CD83	1.04E-05	4.47E-07	-2.42
FAM126A	1.04E-05	4.48E-07	2.96
AIG1	1.05E-05	4.50E-07	2.7
LGALS3	1.05E-05	4.51E-07	-1.93
SYBU	1.05E-05	4.52E-07	-2.63
C15orf52	1.05E-05	4.52E-07	2.01
NR0B1	1.05E-05	4.54E-07	2.25
HK1	1.05E-05	4.54E-07	2.63
TM7SF2	1.06E-05	4.57E-07	2.7
FGFRL1	1.06E-05	4.57E-07	2.23
PFKFB4	1.06E-05	4.58E-07	2.57
DRAP1	1.06E-05	4.59E-07	2.14
LOXL2	1.06E-05	4.59E-07	2.28
CYB5R4	1.06E-05	4.61E-07	2.03
TMEM179	1.06E-05	4.62E-07	2.75
VPS13D	1.07E-05	4.63E-07	2.27
NDRG4	1.07E-05	4.64E-07	2.29
FUT4	1.07E-05	4.65E-07	3.1
CBFB	1.07E-05	4.65E-07	2.07
MN1	1.07E-05	4.66E-07	-2.42
NR1D2	1.07E-05	4.68E-07	2.34
TLN1	1.08E-05	4.69E-07	2.47
C1orf63	1.08E-05	4.70E-07	1.93
BTBD9	1.08E-05	4.71E-07	2.6
BTBD11	1.08E-05	4.71E-07	4.87
C12orf75	1.08E-05	4.72E-07	2.23
ZNF419	1.08E-05	4.73E-07	2.9
VRK2	1.08E-05	4.73E-07	-2.93
SLC6A17	1.09E-05	4.75E-07	-1.87
KIAA0825	1.09E-05	4.77E-07	-1.83

LRRC43	1.09E-05	4.77E-07	-2.03
EDARAD	1.10E-05	4.80E-07	2.08
CCDC89	1.10E-05	4.83E-07	2.66
HIVEP3	1.10E-05	4.85E-07	2.15
DLG1	1.11E-05	4.87E-07	2.86
PIK3AP1	1.11E-05	4.89E-07	2.78
VPS37D	1.11E-05	4.89E-07	2.54
HOXA5	1.11E-05	4.90E-07	2.79
KLHL4	1.11E-05	4.90E-07	2.11
ZNF275	1.11E-05	4.90E-07	1.99
ALG1L9P	1.12E-05	4.92E-07	-2.13
LAT2	1.12E-05	4.93E-07	2.04
FAM3C	1.12E-05	4.93E-07	2.69
C4BPA	1.12E-05	4.93E-07	-2.58
PPP3CB	1.12E-05	4.94E-07	2.87
PRKAB2	1.12E-05	4.95E-07	2.14
CCDC172	1.12E-05	4.95E-07	-2.35
SLC7A5	1.12E-05	4.97E-07	-2.74
MOB3B	1.12E-05	4.97E-07	-2.52
N4BP1	1.12E-05	4.97E-07	1.84
ASZ1	1.13E-05	4.99E-07	-4.4
NAT14	1.13E-05	4.99E-07	2.38
ZDHHC2	1.13E-05	5.00E-07	2.43
GUSBP2	1.13E-05	5.01E-07	-1.87
F12	1.13E-05	5.03E-07	1.97
EIF4A2	1.14E-05	5.04E-07	2.25
IL1R1	1.15E-05	5.10E-07	2.33
HIST1H2I	1.15E-05	5.12E-07	-2.3
LIMK1	1.15E-05	5.13E-07	2.25
ABRACL	1.15E-05	5.14E-07	3.13
TSPAN3	1.16E-05	5.16E-07	2.1
PPP1CB	1.16E-05	5.19E-07	1.92
BEND5	1.16E-05	5.20E-07	3.8
ARHGEF	1.16E-05	5.21E-07	-2.29
CCDC146	1.17E-05	5.22E-07	3.83
DHCR24	1.17E-05	5.24E-07	1.91
ZFP90	1.17E-05	5.25E-07	2.13
PFKFB3	1.17E-05	5.25E-07	2.67
ARHGAP.	1.17E-05	5.25E-07	3.71
SLC25A20	1.17E-05	5.25E-07	2.1

THAP6	1.17E-05	5.26E-07	2.57
EVI2A	1.17E-05	5.27E-07	5.33
PMS2P5	1.17E-05	5.28E-07	2.45
TP53INP2	1.18E-05	5.29E-07	3.3
HSBP1L1	1.18E-05	5.31E-07	-2.72
RAB9A	1.18E-05	5.33E-07	-1.87
HRASLS	1.19E-05	5.35E-07	7.24
ABHD11	1.19E-05	5.36E-07	2.67
SCARB1	1.19E-05	5.38E-07	-2.29
ID2	1.20E-05	5.40E-07	2.85
CEP120	1.20E-05	5.43E-07	1.96
CAV1	1.20E-05	5.43E-07	2.73
HIST2H2I	1.21E-05	5.45E-07	-2.05
EGFR	1.21E-05	5.45E-07	-2.86
KLF4	1.21E-05	5.48E-07	-2.69
AP1S2	1.21E-05	5.49E-07	2.79
VAMP5	1.21E-05	5.50E-07	-3.43
DGKQ	1.22E-05	5.52E-07	2.02
SIKE1	1.22E-05	5.55E-07	2.51
C6orf147	1.23E-05	5.57E-07	3.02
PLEKHA4	1.23E-05	5.57E-07	2.12
ZNF486	1.23E-05	5.59E-07	2.31
CHIC2	1.23E-05	5.60E-07	2.19
KCNQ1	1.23E-05	5.60E-07	-2.06
RCAN2	1.23E-05	5.61E-07	3.42
CHST7	1.23E-05	5.62E-07	2.55
ERCC8	1.24E-05	5.63E-07	2.75
ULBP1	1.24E-05	5.64E-07	2.72
GPR137B	1.24E-05	5.64E-07	2.34
ZNF549	1.24E-05	5.65E-07	3.87
AP3S1	1.24E-05	5.66E-07	1.77
C2orf74	1.26E-05	5.76E-07	2.19
SMU1	1.26E-05	5.76E-07	2.11
PLEKHA6	1.26E-05	5.78E-07	-2.39
CLTA	1.27E-05	5.80E-07	2.36
CFB	1.27E-05	5.82E-07	-2.16
DCAF7	1.27E-05	5.84E-07	1.8
FGF18	1.28E-05	5.86E-07	2.03
TMEFF2	1.28E-05	5.87E-07	3.58
ZNF385B	1.28E-05	5.87E-07	1.86

ONECUT	1.28E-05	5.89E-07	-2.33
CAB39L	1.28E-05	5.89E-07	2.4
KCNJ2	1.28E-05	5.89E-07	3.23
CGB|CGI	1.28E-05	5.89E-07	2.55
ELAVL1	1.28E-05	5.90E-07	2.51
CHST2	1.28E-05	5.91E-07	3.18
CITED2	1.29E-05	5.93E-07	2.19
SEC62	1.29E-05	5.96E-07	1.94
SLC2A1	1.29E-05	5.98E-07	2.1
SMIM3	1.29E-05	5.98E-07	2.5
SACS	1.30E-05	5.98E-07	2.72
NOG	1.30E-05	5.99E-07	-2.31
TXK	1.30E-05	5.99E-07	2.75
ASNS	1.30E-05	6.00E-07	2.61
PPEF1	1.30E-05	6.00E-07	-2.33
DLG3	1.30E-05	6.01E-07	2.38
NAT1	1.30E-05	6.01E-07	2.53
OCLN	1.30E-05	6.02E-07	2.81
ZNF69	1.30E-05	6.02E-07	2.1
CDH13	1.31E-05	6.10E-07	3.04
DAB2	1.32E-05	6.11E-07	-2.44
RALGPS1	1.32E-05	6.13E-07	1.86
SCAMP4	1.33E-05	6.17E-07	2.1
DCP2	1.34E-05	6.24E-07	2.85
LTBP4	1.34E-05	6.25E-07	2.04
HS6ST3	1.34E-05	6.26E-07	2.33
DEPDC1I	1.34E-05	6.27E-07	3.04
PLEKHH	1.34E-05	6.28E-07	2.75
GGT1|G(	1.35E-05	6.30E-07	-1.86
JPH3	1.35E-05	6.31E-07	-1.85
PXK	1.35E-05	6.31E-07	1.87
CHN1	1.36E-05	6.35E-07	2.33
ZDHHC3	1.36E-05	6.37E-07	2.06
GLRB	1.36E-05	6.39E-07	3.05
ADAM32	1.36E-05	6.39E-07	2.98
ZDHHC1	1.37E-05	6.40E-07	1.88
CCDC109	1.37E-05	6.42E-07	2.4
AGTR1	1.37E-05	6.44E-07	3.07
CD44	1.37E-05	6.45E-07	2.66
ZSWIM6	1.38E-05	6.47E-07	1.78

STK40	1.38E-05	6.48E-07	2.29
RFX3	1.38E-05	6.49E-07	1.85
HNMT	1.38E-05	6.49E-07	1.9
SPTSSB	1.38E-05	6.49E-07	1.93
CCDC107	1.38E-05	6.49E-07	2.4
HIST1H3(	1.38E-05	6.50E-07	-1.8
FOXF2	1.38E-05	6.51E-07	-2.41
SNX19	1.38E-05	6.51E-07	2.24
METRN	1.38E-05	6.54E-07	2.2
NHS	1.39E-05	6.56E-07	-3.64
PZP	1.39E-05	6.56E-07	-2.66
MOB1B	1.39E-05	6.57E-07	2.25
TNXB|L(	1.39E-05	6.57E-07	-2.06
LOC1005(	1.39E-05	6.57E-07	2.16
PROSC	1.39E-05	6.59E-07	2.95
F8	1.40E-05	6.62E-07	2.03
SEMA3B	1.40E-05	6.64E-07	-2.24
GNRH1	1.40E-05	6.66E-07	2.42
VGLL2	1.41E-05	6.69E-07	3.19
ABCA5	1.41E-05	6.70E-07	-2.32
AIFM2	1.41E-05	6.72E-07	2.14
LDLR	1.42E-05	6.74E-07	2.55
FOXD4L3	1.42E-05	6.75E-07	2.58
SPANXB1	1.42E-05	6.78E-07	3.03
CHAC1	1.42E-05	6.78E-07	2.71
BAI2	1.43E-05	6.80E-07	2.03
CNNM1	1.43E-05	6.82E-07	-3.2
OSBPL3	1.44E-05	6.87E-07	2.15
SNX8	1.44E-05	6.87E-07	2.7
LRRN1	1.44E-05	6.89E-07	4.2
DDX58	1.44E-05	6.90E-07	3.24
PCYOX1I	1.44E-05	6.90E-07	2.07
KLF2	1.44E-05	6.90E-07	3.87
POLR2B	1.45E-05	6.93E-07	2.01
KCTD15	1.45E-05	6.96E-07	-1.9
TSPAN14	1.45E-05	6.97E-07	2.15
FRZB	1.47E-05	7.05E-07	1.91
NPAS3	1.47E-05	7.05E-07	2.18
GPRC5B	1.47E-05	7.09E-07	1.81
COL5A1	1.47E-05	7.09E-07	-1.75

SPDYA	1.48E-05	7.11E-07	2.12
RPGRIP1]	1.48E-05	7.12E-07	2.01
ZFP82	1.48E-05	7.14E-07	2.78
NUPR1	1.48E-05	7.15E-07	-1.72
LOC64785	1.49E-05	7.17E-07	2.82
BST2	1.49E-05	7.19E-07	-1.99
LRIG3	1.49E-05	7.20E-07	2.08
FAM96B	1.49E-05	7.23E-07	2.15
FKBP1B	1.50E-05	7.24E-07	2.8
WNT5B	1.50E-05	7.28E-07	2.92
GSTA4	1.50E-05	7.29E-07	2.29
MAN1A1	1.50E-05	7.30E-07	1.85
SCN9A	1.51E-05	7.33E-07	2.39
RSPRY1	1.51E-05	7.34E-07	2.47
NR4A1	1.51E-05	7.35E-07	-1.77
GM2A	1.51E-05	7.36E-07	2.01
RLN2	1.52E-05	7.40E-07	3.38
PHF7	1.52E-05	7.40E-07	2.48
FLRT2	1.52E-05	7.40E-07	3.09
STEAP2	1.52E-05	7.40E-07	1.98
RASSF9	1.52E-05	7.41E-07	-1.86
SAMD13	1.53E-05	7.46E-07	2.48
MFAP3L	1.53E-05	7.46E-07	2.53
KAZALD	1.53E-05	7.46E-07	-2.43
FAM72A|	1.53E-05	7.47E-07	2.94
COL17A1	1.53E-05	7.48E-07	3.02
C17orf100	1.53E-05	7.49E-07	1.83
FAT3	1.55E-05	7.57E-07	2.74
ATP6V0A	1.55E-05	7.58E-07	-3.23
NTRK3	1.55E-05	7.60E-07	1.99
DBF4	1.55E-05	7.61E-07	1.88
NUP43	1.55E-05	7.62E-07	2.24
PTPRJ	1.56E-05	7.65E-07	2.52
MACF1	1.56E-05	7.66E-07	2.26
C10orf55	1.56E-05	7.68E-07	1.96
S100A3	1.56E-05	7.68E-07	2.72
CMTM6	1.57E-05	7.72E-07	2.11

miRNA_I[illegible]	adj.P.Val	logFC	Up/Down
hsa-miR-1	0.000136	-1.0344307	Down
hsa-miR-1	0.000136	-1.2138132	Down
hsa-miR-9	0.000169	2.0888741	Up
hsa-miR-9	0.000179	-1.0486019	Down
hsa-miR-2	0.000198	-1.1735643	Down
hsa-miR-3	0.000198	-1.1558731	Down
hsa-miR-2	0.000236	-1.2446829	Down
hsa-miR-3	0.000466	-1.0359961	Down
hsa-miR-1	0.000518	-1.6447052	Down
hsa-miR-1	0.000518	-1.6197975	Down
hsa-miR-3	0.000634	-1.1038569	Down
hsa-miR-4	0.000999	-1.0019685	Down
hsa-miR-9	0.001384	-1.4485846	Down
hsa-miR-1	0.002031	-1.4158903	Down
hsa-miR-2	0.002271	-1.367624	Down
hsa-miR-4	0.002271	1.4713474	Up
hsa-miR-6	0.002271	-1.0597579	Down
hsa-miR-9	0.002316	-1.0427309	Down
hsa-miR-1	0.00465	-1.2956839	Down
hsa-miR-1	0.00465	-1.0568608	Down
hsa-miR-2	0.004936	-1.3058275	Down
hsa-miR-5	0.004936	-1.0993551	Down
hsa-miR-1	0.006619	-1.7032207	Down
hsa-miR-3	0.007286	-1.1248264	Down
hsa-miR-6	0.008164	-1.3360606	Down
hsa-miR-6	0.009177	-1.2606091	Down
hsa-miR-1	0.010126	-1.3133811	Down
hsa-miR-3	0.022019	-1.2792063	Down
hsa-miR-2	0.036102	-1.1117024	Down

Gene_symbol	adj.P.Val	logFC	Up/Down
SOX2-OT	0.00000216	9.3	Up
LINC00461	6.13E-08	8.91	Up
LINC00973	9.84E-08	7.5	Up
RP3-428L16.2	6.13E-08	7.25	Up
RP5-875H18.9	0.00000022	7.14	Up
CTD-2620I22.7	0.000000396	6.96	Up
RP11-328C8.5	0.000000177	6.89	Up
RP11-834C11.4	0.000000638	6.58	Up
RP11-119F7.5	0.000000242	6.48	Up
RP11-445F12.1	0.000000278	6.12	Up
LOC400043	0.000000293	6	Up
RP11-11N9.4	0.000000713	5.95	Up
LINC01116	0.00000056	5.89	Up
LINC01551	0.000000999	5.81	Up
SOCS2-AS1	0.000000206	5.7	Up
SFTA1P	0.000003	5.62	Up
RP11-509J21.1	0.000000741	5.46	Up
ZNF529	0.000000674	5.42	Up
ZNF667-AS1	0.000000543	5.37	Up
MGC12916	0.0000004	5.36	Up
TMEM161B-AS1	0.000000162	5.21	Up
LINC00460	0.00000367	5.05	Up
FEZF1-AS1	0.00000188	5.04	Up
PRKCQ-AS1	0.00000119	4.98	Up
MAGI2-AS3	0.00000016	4.97	Up
ZFHX4-AS1	0.00000404	4.78	Up
RP11-384O8.1	0.00000029	4.77	Up
RP11-15H20.6	0.000000364	4.7	Up
CTD-3018O17.3	0.000000438	4.7	Up
LINC00632	0.00000134	4.68	Up
LINC00052	0.000000487	4.65	Up
LINC00707	0.000000392	4.64	Up
LINC00704	0.00000103	4.55	Up
RP11-32B5.8	0.00000189	4.51	Up
LINC00511	0.00000229	4.44	Up
GLIPR2	0.000000183	4.39	Up
SNHG23	0.000000508	4.38	Up
HIVEP3	0.00000278	4.38	Up
ZNF542	0.00000419	4.35	Up

RP11-404J23.1	0.00000112	4.31	Up
AC006116.21	0.000000575	4.21	Up
CTD-3049M7.1	0.000000424	4.18	Up
RP11-677M24.1	0.000000906	4.16	Up
RP11-436H11.5	0.000000437	4.01	Up
RP11-346D6.6	0.00000436	3.99	Up
RP11-324L17.1	0.00000496	3.98	Up
RP11-232D9.3	0.000000905	3.95	Up
SCOC-AS1	0.000000673	3.86	Up
RP4-639F20.1	0.000000518	3.79	Up
ADCY10P1	0.00000225	3.78	Up
AC159540.1	0.000000293	3.74	Up
RP11-734I18.1	0.00000171	3.74	Up
LINC01279	0.00000457	3.72	Up
RP11-443A13.5	0.000000563	3.71	Up
KCNQ5-IT1	0.00000423	3.67	Up
HCG4	0.00000265	3.61	Up
LINC01268	0.00000482	3.61	Up
LINC00342	0.000000496	3.59	Up
BLACAT1	0.000000749	3.49	Up
RPL21P44	0.00000101	3.49	Up
LINC00622	0.00000415	3.46	Up
RP11-128A17.2	0.00000401	3.45	Up
APCDD1L-AS1	0.00000265	3.37	Up
RP11-418J17.1	0.0000033	3.36	Up
TP53TG1	0.00000146	3.26	Up
LOC284581	0.000000942	3.14	Up
MGC45800	0.00000307	3.14	Up
LOXL1-AS1	0.00000297	3.11	Up
LINC00639	0.00000216	3.09	Up
RP11-320M16.2	0.00000419	3.07	Up
UG0898H09	0.0000049	3.04	Up
RP11-473M20.9	0.000000975	3.02	Up
LINC00601	0.00000135	3	Up
RP1-290I10.5	0.00000106	2.96	Up
HOTAIRM1	0.00000212	2.93	Up
RP11-981G7.6	0.00000447	2.87	Up
RP11-384L8.1	0.00000441	2.86	Up
AP000473.8	0.00000291	2.85	Up
RUNX2	0.00000142	2.82	Up

LINC00702	0.00000228	2.8	Up
RP5-1086K13.1	0.00000231	2.79	Up
RP4-782G3.1	0.00000502	2.79	Up
RP11-15A1.3	0.00000244	2.7	Up
FAM95B1	0.00000291	2.7	Up
TNKS2-AS1	0.00000483	2.58	Up
RP11-474O21.5	0.00000213	2.52	Up
RP11-460B17.2	0.00000364	2.52	Up
RP11-320N7.2	0.00000481	2.47	Up
LINC00312	0.00000408	2.46	Up
CRYBB2P1	0.00000325	2.43	Up
COPG2IT1	0.00000422	2.38	Up
RP11-60L3.1	0.00000482	2.21	Up
MIR4435-1HG	0.00000466	2.18	Up
RP11-21L23.3	0.00000462	-2.18	Down
RP11-63P12.7	0.00000322	-2.39	Down
KTN1-AS1	0.00000437	-2.4	Down
VPS9D1-AS1	0.00000291	-2.41	Down
LINC00323	0.00000351	-2.45	Down
RP11-806O11.1	0.00000342	-2.46	Down
RP4-781B1.5	0.00000373	-2.5	Down
A2M-AS1	0.000003	-2.56	Down
LINC01033	0.00000313	-2.64	Down
LINC00518	0.00000229	-2.65	Down
PRRT3-AS1	0.00000284	-2.65	Down
AC009229.6	0.00000196	-2.67	Down
FAM230C	0.00000411	-2.67	Down
LINC00964	0.00000371	-2.71	Down
RP4-799P18.4	0.00000265	-2.72	Down
CTD-2291D10.4	0.0000027	-2.73	Down
RP11-49I11.4	0.00000179	-2.79	Down
FOXP4-AS1	0.00000293	-2.81	Down
LINC00963	0.00000336	-2.82	Down
CERS6-AS1	0.00000147	-2.84	Down
RP11-383J24.1	0.00000108	-2.87	Down
RP11-706O15.3	0.00000264	-2.88	Down
LOC151009	0.00000312	-2.89	Down
RP3-406A7.7	0.00000105	-2.9	Down
RP11-90K6.1	0.0000048	-2.9	Down
AC009299.3	0.00000361	-2.94	Down

AP001059.5	0.00000223	-2.95	Down
LINC00313	0.00000456	-2.95	Down
CTD-2561J22.5	0.0000016	-2.97	Down
RP1-27K12.2	0.00000218	-2.97	Down
LOC100506737	0.00000216	-2.98	Down
RP11-392P7.6	0.00000282	-2.99	Down
CDKN2B-AS1	0.00000319	-3.01	Down
ANKRD19P	0.000000952	-3.05	Down
RP11-496N12.6	0.00000154	-3.08	Down
DNAH17-AS1	0.00000227	-3.17	Down
RP11-78A19.4	0.00000262	-3.18	Down
STOM	0.00000427	-3.19	Down
RP11-398H6.1	0.00000142	-3.22	Down
ARHGEF34P	0.00000159	-3.26	Down
AC009336.24	0.00000261	-3.26	Down
RP11-65J21.3	0.000000716	-3.3	Down
RP11-106D4.2	0.00000087	-3.31	Down
RP11-167J8.3	0.000001	-3.31	Down
LOC101060264	0.00000147	-3.41	Down
KRT18P55	0.000000845	-3.45	Down
RP11-392E22.10	0.00000473	-3.46	Down
HCG11	0.000000535	-3.47	Down
LHFPL3-AS1	0.000000478	-3.57	Down
AC006262.5	0.00000421	-3.58	Down
KCNK15-AS1	0.000000613	-3.62	Down
LOC100996579	0.000000528	-3.63	Down
HOTAIR	0.000000958	-3.65	Down
RP5-843L14.1	0.00000242	-3.65	Down
SERPINB1	0.00000113	-3.66	Down
RP11-96D1.11	0.000000525	-3.7	Down
AP001505.9	0.00000096	-3.79	Down
RP1-80N2.2	0.00000103	-3.79	Down
RP11-298A8.2	0.000000364	-3.8	Down
LINC01106	0.00000343	-3.8	Down
LINC00648	0.00000322	-3.81	Down
PGAP2	0.000000553	-3.82	Down
FAM201A	0.000000758	-3.83	Down
IFITM4P	0.000000566	-3.84	Down
CH17-360D5.2	0.00000207	-3.84	Down
CASC9	0.000000325	-3.95	Down

FENDRR	0.000000794	-3.95	Down
RP11-190J1.3	0.000000975	-3.96	Down
RP11-820L6.1	0.000000819	-3.99	Down
AC009229.5	0.000000531	-4	Down
RP11-542M13.3	0.000000212	-4.05	Down
LINC01139	0.00000355	-4.06	Down
CYP4F26P	0.00000236	-4.08	Down
CTA-941F9.9	0.000000268	-4.09	Down
HOXA11-AS	0.000000471	-4.14	Down
RP11-30J20.1	0.00000168	-4.2	Down
RP13-726E6.1	0.0000002	-4.23	Down
LINC00969	0.000000204	-4.29	Down
RP11-416N2.4	0.00000145	-4.35	Down
RP11-314B1.2	0.00000124	-4.47	Down
RP11-337N6.1	0.00000039	-4.49	Down
CTD-2319I12.1	0.000000188	-4.5	Down
LINC01352	0.00000264	-4.57	Down
HOTTIP	0.00000144	-4.61	Down
RP11-354K1.1	0.000000423	-4.62	Down
C1QTNF1-AS1	0.000000228	-4.64	Down
RP11-706O15.7	0.000000453	-4.68	Down
LINC00968	0.000000402	-4.7	Down
RP11-1149O23.3	0.000000157	-4.84	Down
RP11-132A1.4	0.000000593	-4.84	Down
RP4-584D14.7	0.00000139	-4.85	Down
RP11-736N17.10	0.00000343	-5.29	Down
TINCR	0.000000086	-5.41	Down
RP11-90D4.3	0.00000028	-5.44	Down
RP11-66B24.7	0.000000437	-5.47	Down
RP11-115D19.1	0.000000758	-5.49	Down
LINC01021	0.00000014	-5.58	Down
CASC19	0.00000381	-5.58	Down
BASP1P1	0.000000157	-5.76	Down
LINC00987	0.00000342	-5.82	Down
CH17-360D5.3	0.000000101	-5.87	Down
RP11-706O15.5	0.00000022	-5.95	Down
LINC00958	0.000000188	-6.02	Down
RP11-49I11.1	0.000000154	-6.09	Down
MSL3P1	0.00000178	-6.11	Down
RP11-119D9.1	6.13E-08	-6.62	Down

LINC00473	6.76E-08	-7.17	Down
RP3-523E19.2	0.00000046	-7.54	Down
LINC00162	0.000000601	-8.04	Down
H19	4.68E-08	-14.2	Down

Ferrotosis	Name
ACSL4	acyl-CoA synthetase long-chain family member 4
AKR1C1	aldo-keto reductase family 1 member C1
AKR1C2	aldo-keto reductase family 1 member C2
AKR1C3	aldo-keto reductase family 1 member C3
ALOX15	arachidonate 15-lipoxygenase
ALOX5	arachidonate 5-lipoxygenase
ALOX12	arachidonate 12-lipoxygenase
ATP5MC3	ATP synthase membrane subunit c locus 3
CARS	cysteinyl tRNA synthetase
CBS	cystathion ine beta synthase
CD44	CD44 molecule
CHAC1	ChaC glutathione- specific gamma-glutamyl cyclotransferase 1
CISD1	CDGSH iron sulfur domain 1
CS	citrate synthase
DPP4	dipeptidyl-dippeptidase-4
FANCD2	Fanconi anemia comple mentation group D2
GCLC	glutamate-cysteine ligase catalytic subunit
GCLM	glutamate-cysteine ligase modifier subunit
GLS2	glutaminase 2
GPX4	glutathio ne peroxidase 4
GSS	glutathione synthetase
HMGCR	3-hydroxy-3- methylglutaryl-CoA reductase
HSPB1	heat shock protein beta 1
CRYAB	heat shock protein beta 5
LPCAT3	lysophosp hatidylcholine acyltransferase 3
MT1G	metallothionein-1G
NCOA4	nuclear receptor coactiva tor 4
PTGS2	prostagla ndin-endoperoxide synthase 2
RPL8	ribosomal protein L8
SAT1	spermidine/spermine N1-acetyltra nsferase 1
SLC7A11	solute carrier family 7 member 11
FDFT1	farnesyl-diphosphate farnesyltransferase 1
TFRC	transferrin receptor
TP53	tumor protein 53
EMC2	ER membrane protein complex subunit 2
AIFM2	apoptosis inducing factor mitochondria associated 2
PHKG2	phospho rylase kinase ,g2
HSBP1	heat-shock 27-k Da protein 1
ACO1	aconitase 1

FTH1	ferritin heavy chain 1
STEAP3	six-transm embrane epithelial antigen of prostate 3
NFS1	cysteine desulfurase
ACSL3	acyl-CoA synthetase long-chain family member 3
ACACA	Acetyl-CoA carboxylase alpha
PEBP1	phosphatidy lethanolamine-binding protein 1
ZEB1	zinc finger E-box-binding homeobox 1
SQLE	squalene monooxygenase
FADS2	fatty acid desaturase 2/acyl-CoA 6-desaturase
NFE2L2	nuclear factor, erythroid 2 like 2
KEAP1	kelch-like ECH- associated protein 1
NQO1	quinone oxidoreductas e-1
NOX1	NADPH oxidase 1
ABCC1	ATP binding cassette subfamily C member 1
SLC1A5	solute carrier family 1 member 5
GOT1	glutamic-oxa loacetic transaminase 1
G6PD	glucose-6-phosphate dehydrogenas e
PGD	phosphoglycerate dehydrogenas e
IREB2	iron response element-binding protein 2
HMOX1	heme oxygenase 1
ACSF2	acyl-CoA synthetase family member 2
