# Supplementary material for: Over-expression of lncRNA TMEM161B-AS1 promotes the malignant biological behavior of glioma cells and the resistance to temozolomide via up-regulating the expression of multiple ferroptosis-related genes by sponging hsa-miR-27a-3p
Source: Cell Death Discov. 2021 Oct 23;7:311. doi: 10.1038/s41420-021-00709-4 (PMC8542043; doi:10.1038/s41420-021-00709-4)
Supplement: Supplementary file 5 — Supplemental Table S1 [file 41420_2021_709_MOESM5_ESM.docx]

**Supplemental Table S1 primer sequence for qRT-PCR**

| RNA | Sequence (5’ to 3’) |
| --- | --- |
| *FANCD2* mRNA | Forward sequence：CCGGAATATTGGATTCTCACAT  Reverse sequence：GAACTTTCACTCCTGGTCCATC |
| *CD44* mRNA | Forward sequence：ACATCAGTCACAGACCTGCCC  Reverse sequence：ATCCATGAGTGGTATGGGAC |
| TMEM161B-AS1 | Forward sequence：TTGCGAGGACCCTGAGGTAG  Reverse sequence：AATTGTCACGCTGTTCGTCC |
| hsa-miR-27a-3p | Forward sequence：TGAGGAGCAGGGCTTAGCTG  Reverse sequence：GCGGAACTTAGCCACTGTGA |
| GAPDH | Forward sequence：GCATGGCCTTCCGTGTTC  Reverse sequence：ATGTCATCATACTTGGCAGGT |
| U6 | Forward sequence：ATTGGAACGATACAGAGAAGATT  Reverse sequence：GGAACGCTTCACGAATTTG |

FANCD2, Fanconi anemia complementation group D2. TMEM161B-AS1, transmembrane protein 161B antisense RNA 1. GAPDH, Glyceraldehyde 3-phosphate dehydrogenase. U6, U6 small nuclear RNA.
