# Supplementary material for: Over-expression of lncRNA TMEM161B-AS1 promotes the malignant biological behavior of glioma cells and the resistance to temozolomide via up-regulating the expression of multiple ferroptosis-related genes by sponging hsa-miR-27a-3p
Source: Cell Death Discov. 2021 Oct 23;7:311. doi: 10.1038/s41420-021-00709-4 (PMC8542043; doi:10.1038/s41420-021-00709-4)

All of the co-authors’ email responses as follows:

Qiudan Chen:


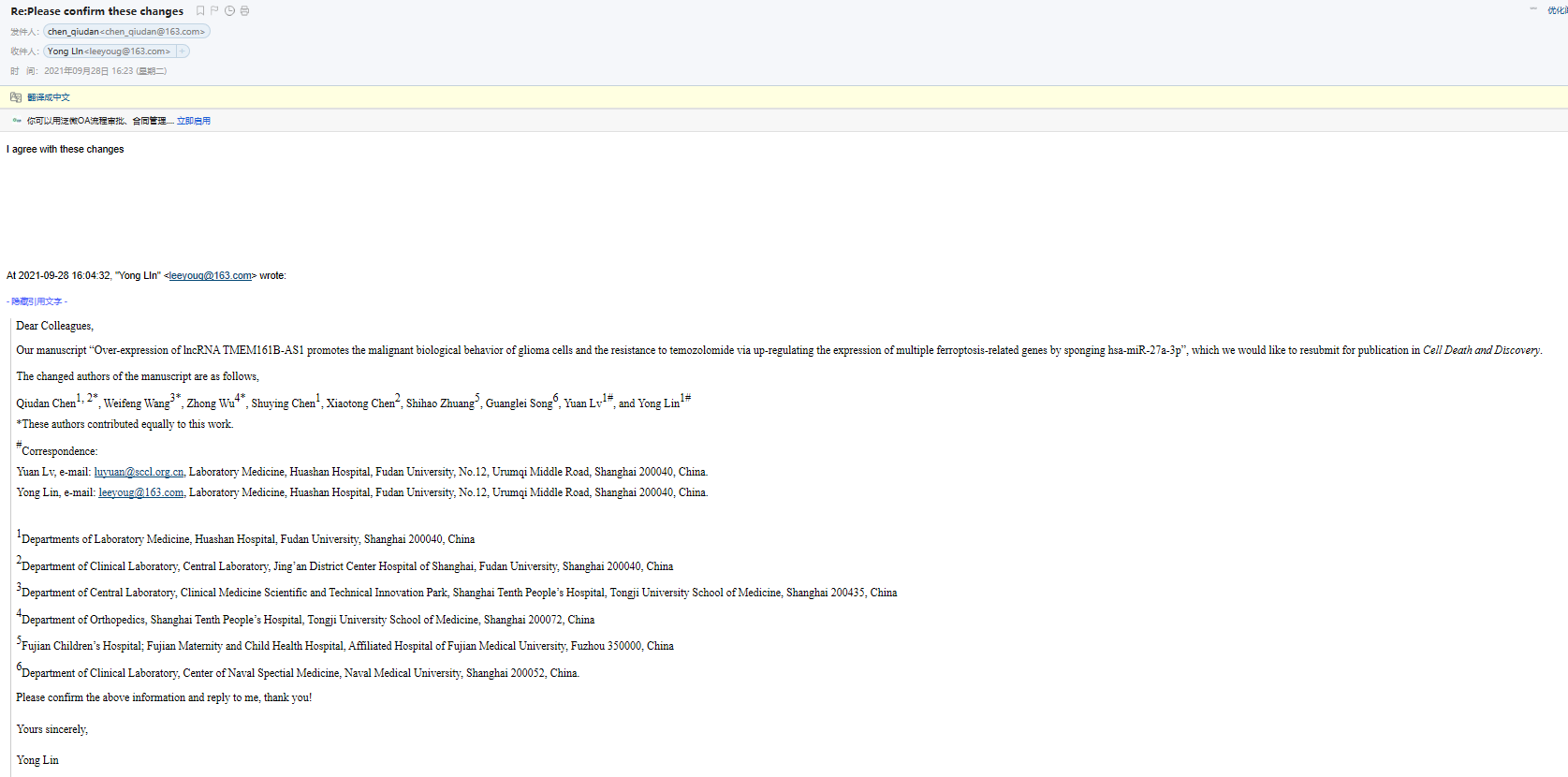


Weifeng Wang:


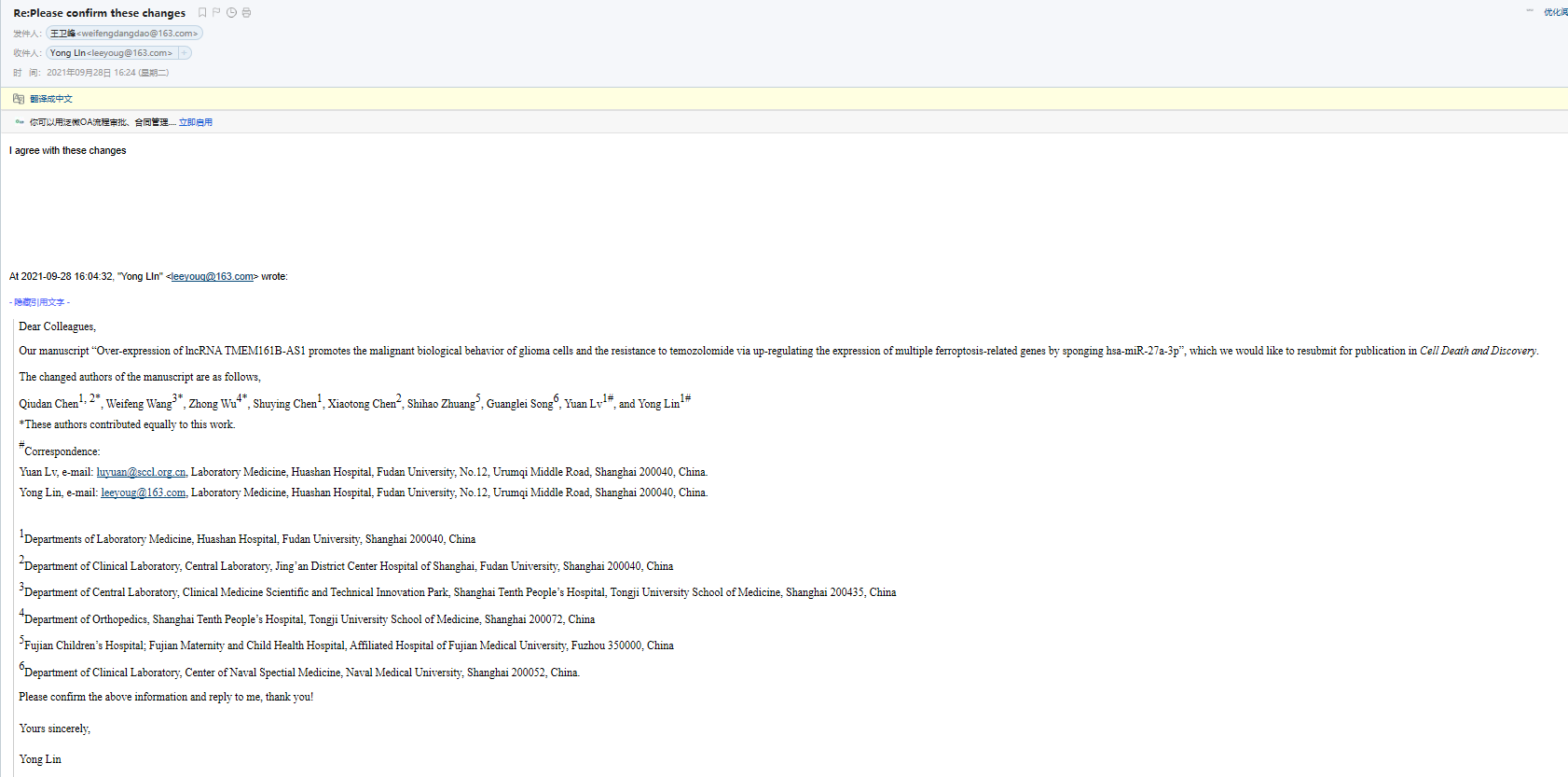


Zhong Wu:


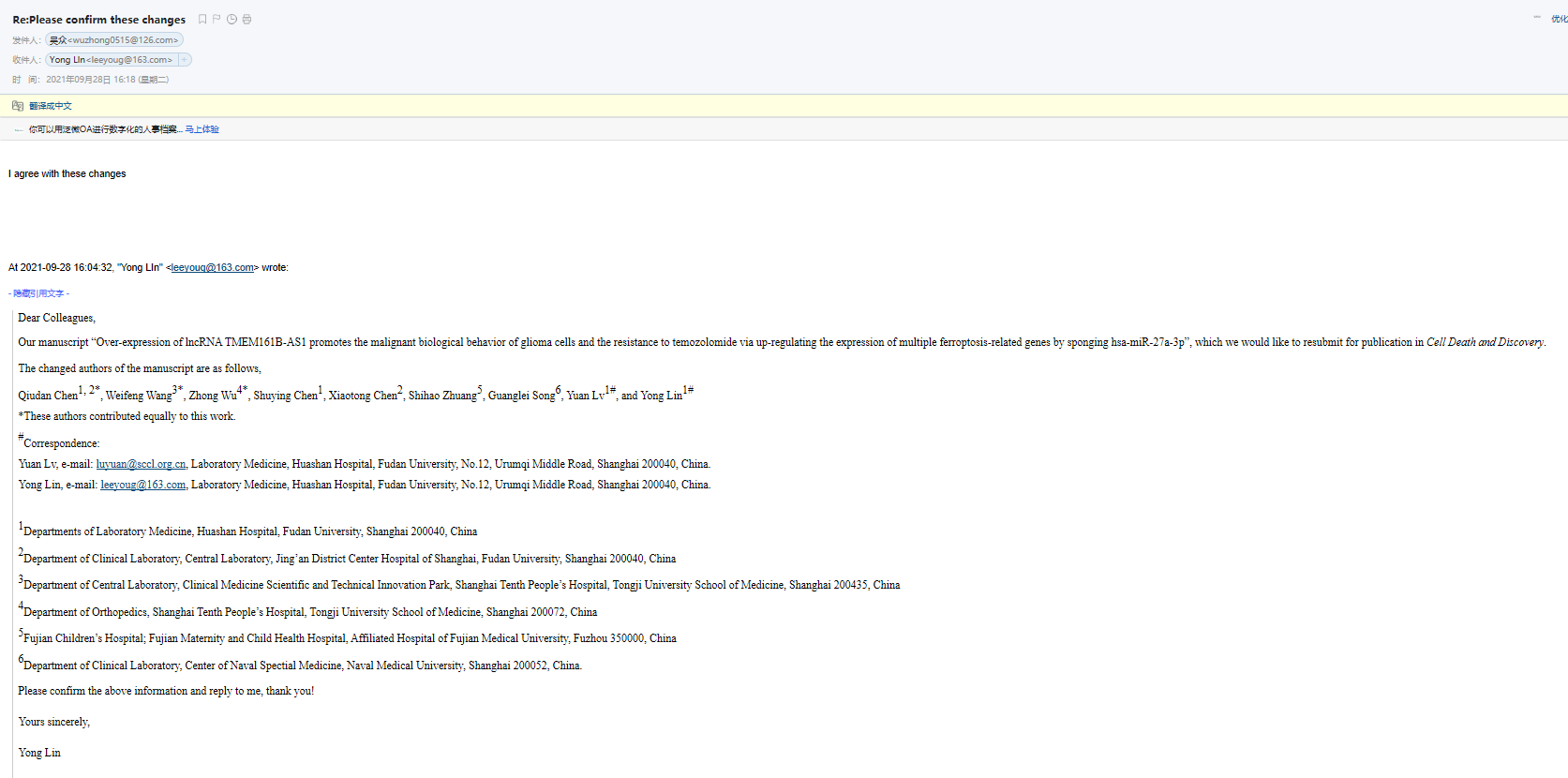


Shuying Chen:


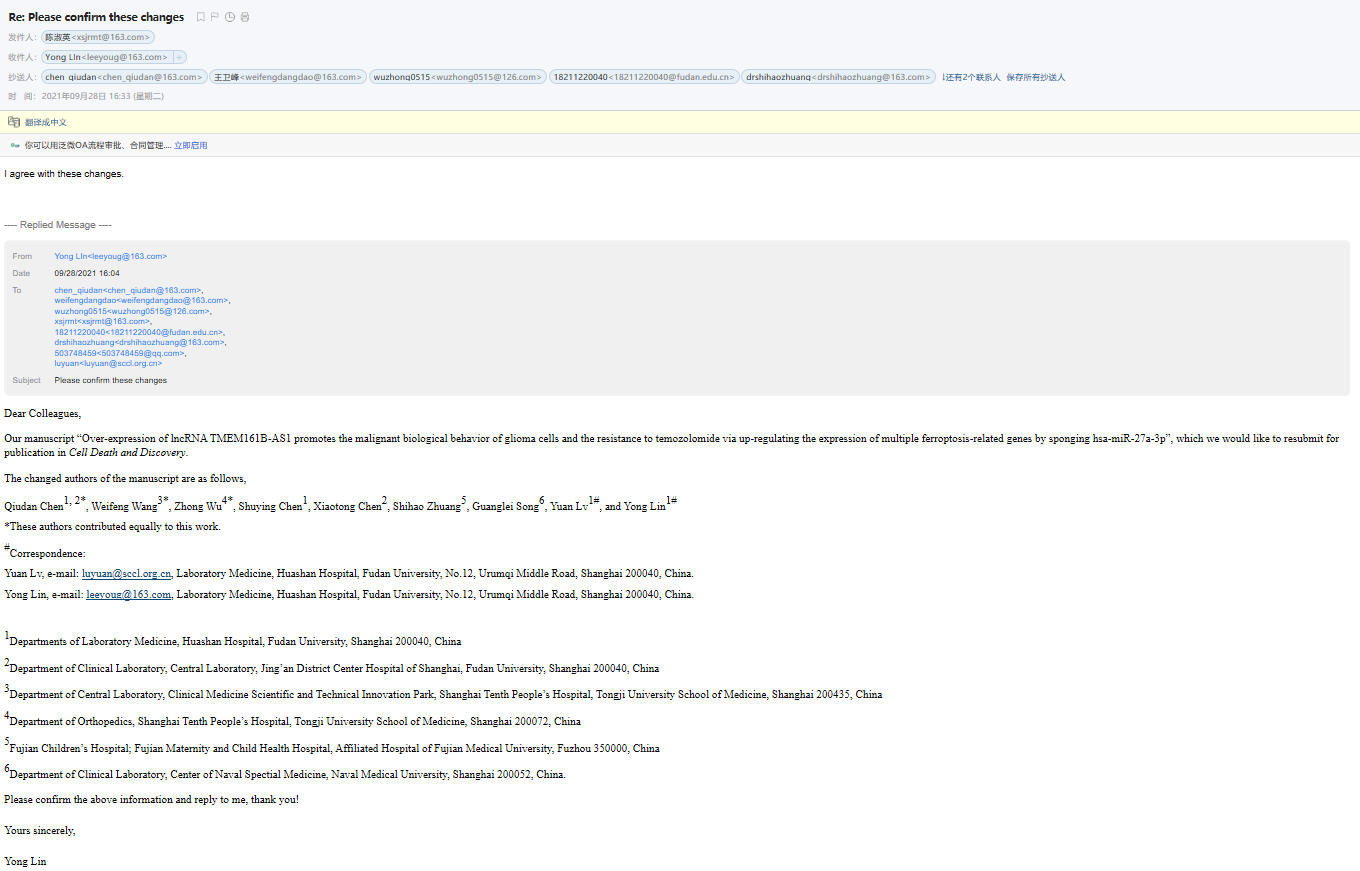


Xiaotong Chen:


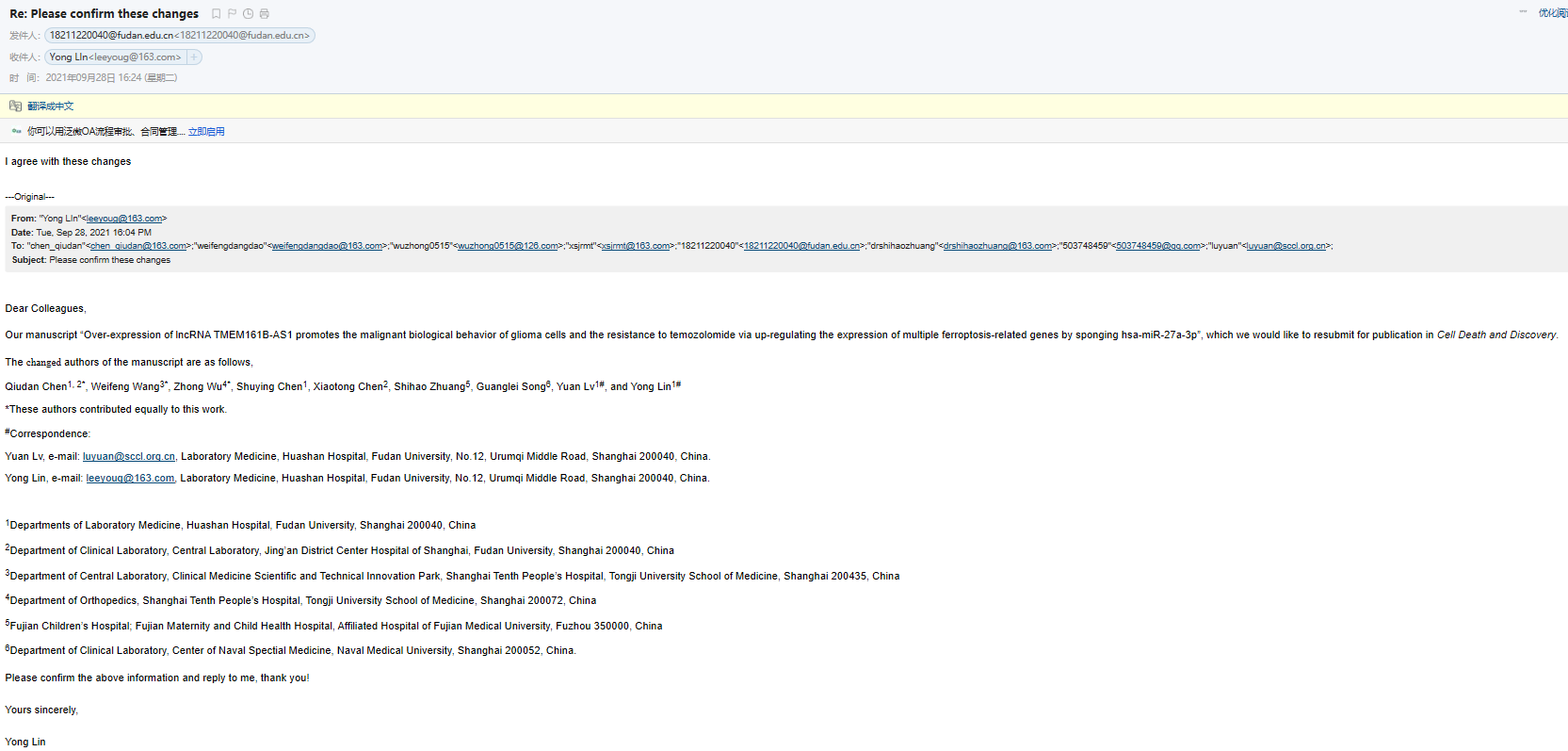


Shihao Zhuang:


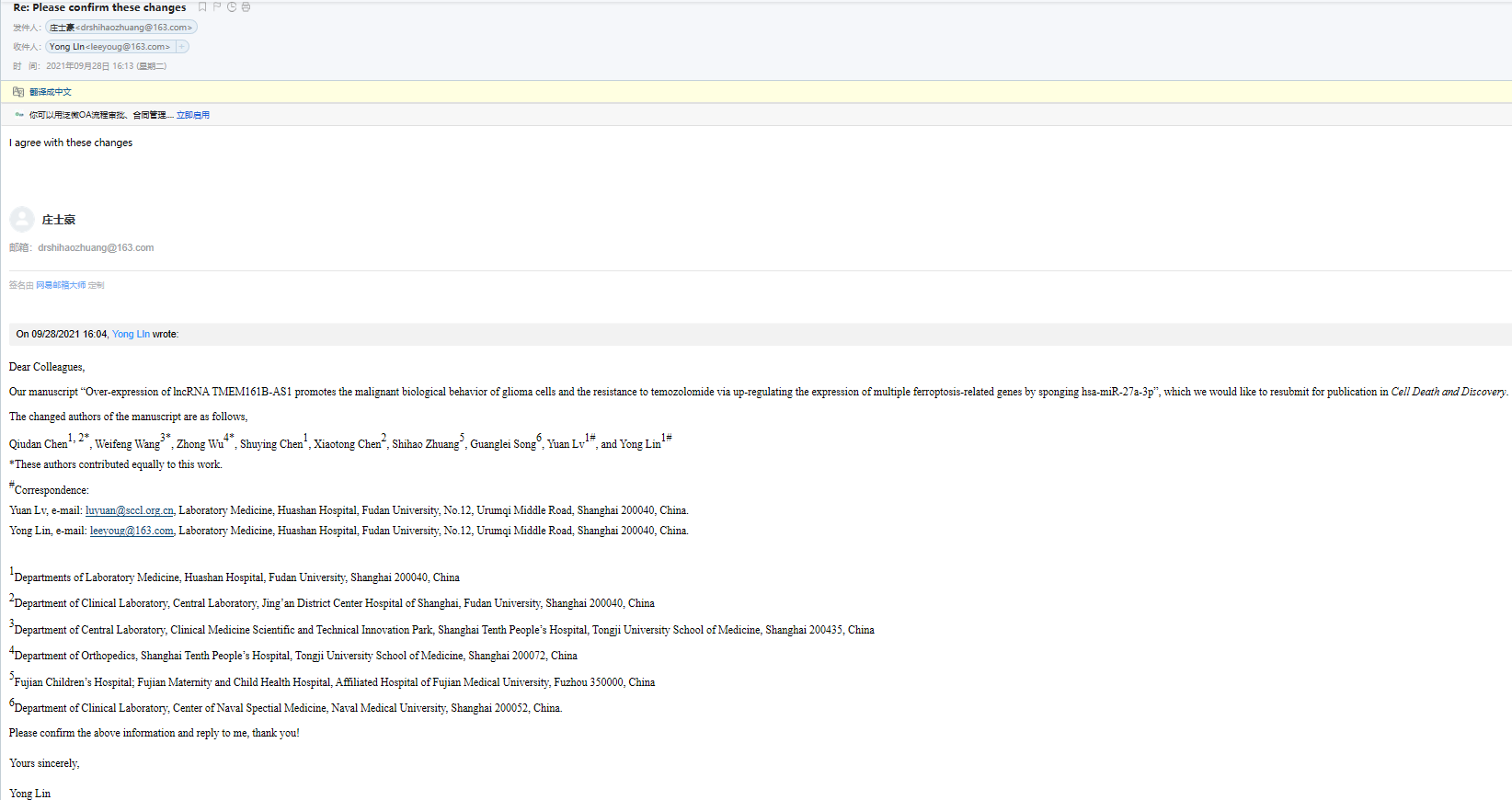


Guanglei Song:


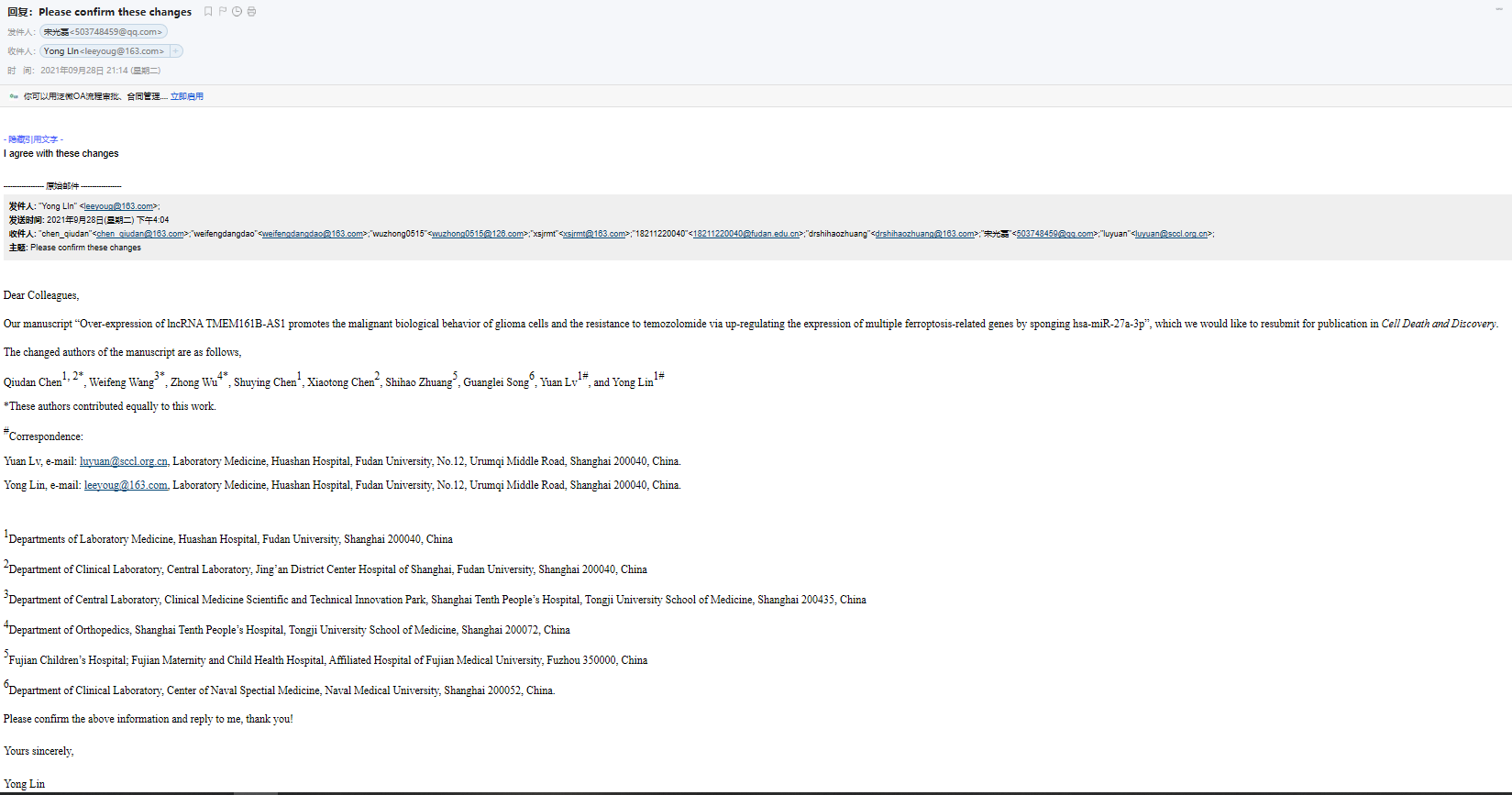


Yuan Lv:


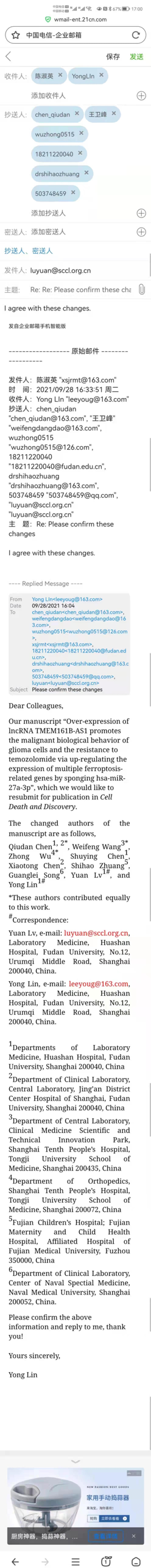

Supplement: Supplementary file 6 — All authors responded to the email confirming their agreement to these changes [file 41420_2021_709_MOESM6_ESM.doc]
